# Supplementary material for: FLIM Playground: An interactive, end-to-end graphical user interface for analyzing single cells with fluorescence lifetime imaging microscopy
Source: Cell Rep Methods. 2026 Jun 10;6(8):101484. doi: 10.1016/j.crmeth.2026.101484 (PMC13494553; doi:10.1016/j.crmeth.2026.101484)
Supplement: Supplementary file 2 [file mmc5.pdf]

Cell Reports
Methods

Article

FLIM Playground: An interactive, end-to-end graphical user interface for analyzing single cells with fluorescence lifetime imaging microscopy

Graphical abstract

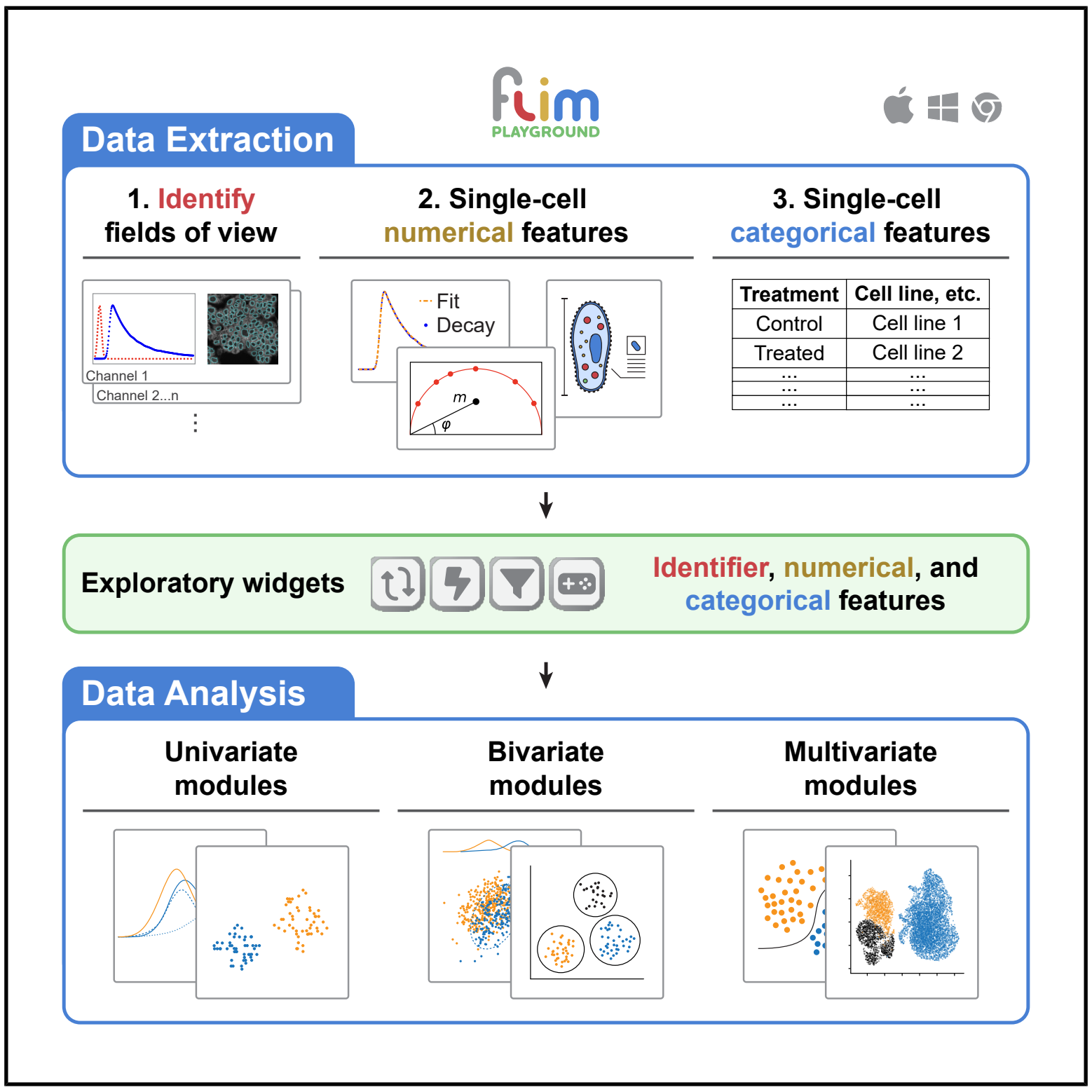

Highlights

- Interactive platform unifies single-cell fluorescence lifetime imaging analysis
- Extendable cell phenotyping with morphology, texture, and metadata
- Encourages fast, iterative exploration through data visualization and analysis
- Demonstrated on FLIM imaging of cancer cells treated with metabolic inhibitors

Authors

Wenxuan Zhao (赵文轩), Kayvan Samimi, Melissa C. Skala, Rupsa Datta

Correspondence

rdatta@morgridge.org

In brief

Analysis of cellular-resolution fluorescence lifetime imaging microscopy (FLIM) remains fragmented across siloed tools requiring custom code. Zhao et al. present FLIM Playground for unified feature extraction, visualization, and statistical modeling in an interactive, code-free platform with modular design for adapting to new imaging modalities and methods.

Zhao et al., 2026, Cell Reports Methods 6, 101484
August 17, 2026 © 2026 The Authors. Published by Elsevier Inc.
https://doi.org/10.1016/j.crmeth.2026.101484

CellPress

Cell Reports Methods

CellPress
OPEN ACCESS

Article

FLIM Playground: An interactive, end-to-end graphical user interface for analyzing single cells with fluorescence lifetime imaging microscopy

Wenxuan Zhao (赵文轩),[1] Kayvan Samimi,[1] Melissa C. Skala,[1,2] and Rupsa Datta[1,3,*]
[1]Morgridge Institute for Research, Madison, WI, USA
[2]Department of Biomedical Engineering, University of Wisconsin, Madison, WI, USA
[3]Lead contact
*Correspondence: rdatta@morgridge.org
https://doi.org/10.1016/j.crmeth.2026.101484

MOTIVATION Turning raw photon decays from fluorescence lifetime imaging microscopy (FLIM) into biological insight remains a fragmented process. Existing tools each cover only part of the pipeline, which spans extraction and analysis across multiple data levels—experiment, field of view, channel, region of interest, and pixel—forcing users to stitch them together with custom code that reformats inputs, processes outputs for downstream tasks, and bridges alternative methods. This patchwork hinders reproducibility and slows hypothesis-driven discovery. We address this with a unified, interactive, code-free graphical interface that spans the full FLIM pipeline and a modular architecture that adapts to new imaging modalities, feature descriptors, and analysis methods.

SUMMARY

Fluorescence lifetime imaging microscopy (FLIM) is sensitive to molecular environments and enables high-resolution mapping of cellular heterogeneity. Yet, the journey from raw photon decays to biological insight remains fragmented by multi-step data extraction and siloed analyses, creating burdens for experts and non-experts alike. This work presents FLIM Playground, the first interactive graphical platform that unifies single-cell FLIM workflows. Modularly designed to encompass data extraction (if desired) and data analysis, FLIM Playground can check field-of-view metadata, calibrate and extract fluorescence lifetime features per region of interest along with morphology and texture features across channels, merge multiple datasets, and provide real-time visual analytic modules. Lifetime extraction was validated against commercial software and published results, and both data extraction and analysis were demonstrated on a FLIM dataset of cancer cell lines. By adopting best practices and offering interactivity, FLIM Playground promotes reproducibility, allows for expansion to new imaging modalities, and accelerates hypothesis-driven discovery.

INTRODUCTION

Fluorescence lifetime imaging microscopy (FLIM) is a popular molecular imaging tool across diverse fields such as cell biology, biophysics, and biomedical research.[1–5] FLIM is sensitive to changes in fluorophore microenvironment including temperature, pH, conformational changes with protein binding, and the presence of quenchers.[2,4] Advancements in instrumentation, detection technologies, acquisition speed, and automated cell-segmentation algorithms[6–9] have greatly expanded its capabilities, making FLIM more powerful and widely adopted. Together, these developments make single-cell analyses possible that reveal biological heterogeneity[10] and underscore the need for commensurate progress in analysis tools.[11]

A typical FLIM experiment spans multiple imaging sessions, each containing multiple multi-channel fields of view (FOVs). Each channel—spectral, acquisition, or color—contains multiple cell-level regions of interest (ROIs), defined by ROI masks and composed of pixel sets. At the pixel level, fluorescence decays can be fitted[12–14] or transformed into phasor features.[15–17] ROI masks or pre-aggregated cell-level decays[18] enable per-ROI mapping of heterogeneity through fit,[19] phasor,[20] morphology, and texture[21] extraction. While the most common use case is single-cell analysis, ROIs can represent any user-defined spatial units including subcellular compartments such as nuclei, cytoplasm, or mitochondria; whole cells; organoids; or binary-mask foregrounds. The channel level introduces fluorophore-specific inputs, enabling per-channel extraction of single-cell numerical features. The FOV level aggregates information across channels,

This is an open access article under the CC BY license (http://creativecommons.org/licenses/by/4.0/).

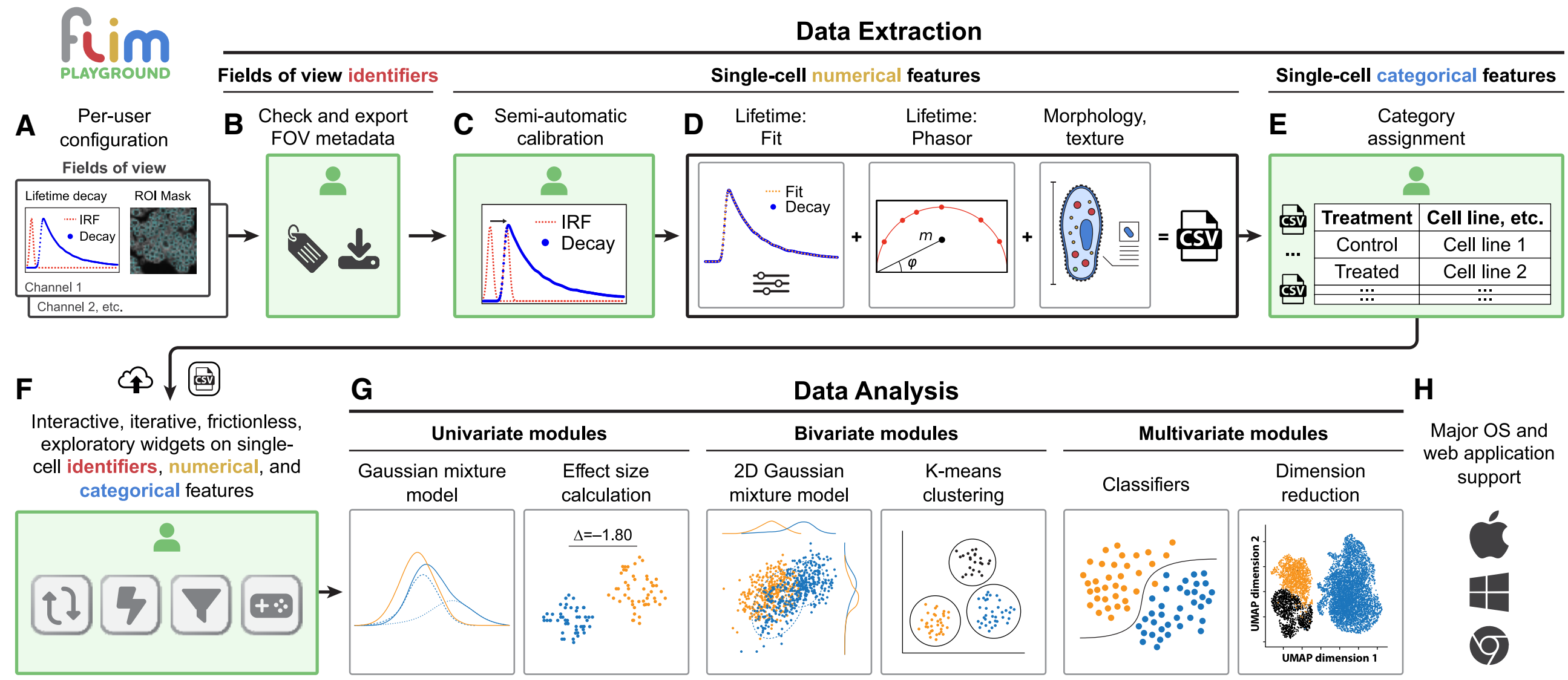

Figure 1. System overview of FLIM Playground

(A–D) (A) After loading a saved configuration, (B) Data Extraction collects and validates channel inputs across all fields of view, (C) calibrates for IRF shifts or uses fluorescence lifetime standards, and (D) extracts single-cell lifetime (fit and phasor), morphology, and texture features in one step.

(E) To bridge Data Extraction with downstream analysis, FLIM Playground combines multiple single-cell datasets and assists users with assigning category labels to each cell.

(F) The three feature classes—identifiers (red), numerical features (yellow), and categorical features (blue)—whether extracted through Data Extraction or from user-provided datasets, are transformed into interactive widgets shared by all analysis methods. Icons denote interface attributes: iterative, frictionless, exploratory, and intuitive.

(G) Data Analysis includes univariate, bivariate, and multivariate modules.

(H) FLIM Playground is available as a ready-to-run executable for major operating systems and as a web application. Interactive interfaces are indicated by a green background.

allowing unique single-cell identifiers to be linked with their imaging context. Finally, the experiment level takes qualitative category labels of FOVs and incorporates them into downstream analysis methods to gain biological insights. Together, pixels, cell ROIs, channels, FOVs, and experiments constitute the hierarchical data levels in FLIM analysis, each representing a distinct unit of information with progressively richer biological semantics.

While a diverse set of tools—both open-source and commercial, ranging from libraries to graphical user interfaces (GUIs)—offers alternative methods and flexibility for FLIM analysis, they typically address only subsets of the data levels. As a result, users often write custom code to reformat inputs, process outputs, and explore alternative methods, leading to a fragmented landscape of FLIM analysis.

FLIM Playground provides a unified framework for integrating diverse methods and input types across data levels, accessible through a cross-platform, interactive, and code-free GUI. It spans the full pipeline: from FOV metadata organization; through calibration with instrument response functions (IRF) or fluorescence lifetime standards; to extraction of single-cell identifiers, numerical features (lifetime fits and phasor, morphology, and texture), and categorical features; followed by visualization and statistical modeling. Embedded validation checks guide users at every step, while interactive widgets and a built-in repertoire of visual-analytic modules encourage hypothesis-driven and iterative exploration of large datasets, including user-provided datasets.

FLIM Playground's modular architecture can incorporate new imaging modalities, feature extraction methods, and analysis modules as they evolve, and because it is open-source and Python-based, other laboratories can easily adopt and extend the platform and leverage the rich Python package ecosystem.

To ensure reliability, we compared FLIM Playground's lifetime fits against the widely used commercial SPCImage software[14] using two-photon autofluorescence FLIM of cells and assessed analysis consistency across multiple hardware configurations. We further illustrate the capabilities of FLIM Playground on autofluorescence FLIM of cancer cell lines (PANC-1 and MCF7) treated with metabolic inhibitors and on a fluorescence lifetime flow cytometry[18] dataset of primary human T cells.

RESULTS

Framework design

FLIM Playground is organized into two main sections: Data Extraction and Data Analysis. Data Extraction derives single-cell features from raw FLIM inputs, while Data Analysis can process these outputs or directly operate on user-provided datasets for data visualization and statistical analysis. Figure 1 provides an overview of the software.

Both Data Extraction and Data Analysis are designed to focus on three classes of single-cell features in the imaging data: categorical features (discrete cell groups such as treatment and cell line), identifiers (FOV identifiers and unique cell identifiers), and

CellPress
OPEN ACCESS

Table 1. FLIM Playground processes at each data level to extract single-cell features from three feature classes

Data levels	Data processing	Feature classes
Experiment	Assign category labels (e.g., treatment, cell line, etc.) to each cell; perform analysis on labeled cell groups.	**Categorical features**
Field of view	Assign FOV identifier to each cell; obtain unique cell identifiers.	**Identifiers**
Channel	Apply per-channel ROI mask, calibration, and extraction methods to cell-level signals.	**Numerical features**
Cell ROI	Aggregate pixel-level decays to obtain ROI-level signals.	N/A
Pixel	Read pixel-level decays encoded in different formats.	N/A

Each data level in the hierarchy is composed of a set of instances from the level below (e.g., a cell is composed of a set of pixels). FLIM Playground manages this complexity by reading pixel-level decays encoded in vendor-specific formats (e.g., Becker & Hickl and PicoQuant) and aggregates the decays that belong to the same cell ROI to obtain the cell-level signals (i.e., cell-level decay and cell intensity image). In this way, we can achieve single-cell resolution at all feature classes (in bold). For each acquisition channel, FLIM Playground flexibly handles the per-channel masks, calibration procedures, and methods (e.g., fit, phasor, texture, and morphology) to extract numerical features. FLIM Playground organizes metadata for FOVs and assigns to cells the FOV they come from and their unique identifiers. Category labels such as treatment and time points are biologically meaningful, and FLIM Playground can assist the user in assigning those category labels to each cell and using them to perform data analysis.

numerical features (quantitative measures such as lifetime and morphology features that capture differences or similarities between groups) (Table 1). At each Data Extraction stage, FLIM Playground targets one feature class and provides interactive interfaces with validation checks and status feedback. Data Analysis builds dedicated interactive components for each feature class, which are shared across the analysis modules it provides, while module-specific components further support a variety of statistical modeling approaches (Table 2).

FLIM Playground employs the interactive GUI library Streamlit,[22] which supports both local and web deployment. PyInstaller[23] bundles the software into executables that run locally on major operating systems.

Data Extraction produces single-cell features using a modularized framework

The modularized Data Extraction framework in FLIM Playground is *channel-centric*, which enables per-channel customization of masks that might focus on different cell ROIs, calibration procedures, and feature extraction methods (Table 1). These assignments are managed through a configuration interface, so the configuration can be set up once and reused across future analyses or altered as needed. After users specify the number of channels, the interface renders a column for configuring settings within each channel. Then users can assign a name (e.g., fluorophore label), an imaging modality, and a list of numerical feature extractors to each channel.

Two imaging modalities are currently supported. In *FLIM mode*, the signal is time-resolved and corresponds to one of the decay types described below. In *intensity-only mode*, the signal is a spatial intensity map without a lifetime axis (e.g., TIFF/TIF format), enabling mixed-modality analyses within the same FOV and supporting analysis of non-FLIM images.

Each channel can be associated with a subset of feature extractors rather than a fixed choice across all channels. Lifetime-based extractors, fit or phasor, can be applied to FLIM channels, while morphology- and texture-based extractors are available for all imaging modalities. These extractors will be introduced in detail in later sections. This modular design also allows future integration of additional imaging modalities and corresponding feature extractors.

Based on the selected imaging modality and feature extractors, the interface prompts the user to specify file suffixes for each required input file type (e.g., raw decay, ROI mask, and calibration file). These are later used during the FOV identification stage. Channels can either share a common ROI mask or use channel-specific masks to target distinct compartments within a cell.

Beyond channel-specific settings, several parameters apply across all channels. Three decay types are supported: (1) 3D/4D decay, consisting of spatial dimensions and the fluorescence decay axis, with an optional spectral channel dimension capturing multiple fluorophores (time-lapse acquisitions are not yet supported); these image data are stored in vendor-specific formats, and FLIM Playground supports those from Becker & Hickl and PicoQuant; (2) 2D decay, where each row of a tabular datasheet corresponds to the fluorescence decay of a single cell and each column represents a time bin along the decay curve—an example of such data is obtained from fluorescence lifetime flow cytometry[18]; and (3) 3D/4D pixel-prefit decay, representing pixel-level lifetime features pre-fit by open-source or commercial software such as SPCImage. Two calibration methods are available for phasor analysis: either by shifting the IRF or by using a fluorescence lifetime standard with known lifetime.[15] Users may also specify a list of categorical features for extraction, which will later be recognized in the Data Analysis section. A few additional settings are described in the Methods S1, Chapter 3; the most up-to-date version of the FLIM Playground manual is also available online.[24]

The next steps after specifying the reusable configuration are the three stages of Data Extraction, each focusing on one feature

Table 2. Modules offered in FLIM Playground Data Analysis

Analysis class & numerical feature selection	Modules	Filter by	Color by	Point-based	Built-in modeling methods
Univariate—select *one* feature from a feature group.	Feature Comparison	✔	✔	✔	Effect size calculation & statistical tests
	Feature Histogram	✔	✔	✘	Gaussian mixture model & heterogeneity index
Bivariate—two univariate selectors, or choose a channel and a harmonic to select phasor coordinates.	Feature Distribution	✔	✔	✔	2D Gaussian mixture model & linear regression
	Phasor Analysis	✔	✔	✔	K-means clustering
Multivariate—select multiple features from each feature group.	Dimension Reduction	✔	✔	✔	PCA, t-SNE, UMAP with user-adjustable hyperparameters
	Classification	✔	✔*	✘	Random Forest, SVM, Logistic Regression, Gradient Boosting

Modules are grouped into three analysis classes—Univariate, Bivariate, and Multivariate—based on the number of numerical features required, with custom feature-selection widgets implemented for each class. Each module pairs an interactive visualization with a statistical modeling method. *Filter by* and *Color by* are interactive widgets shared by all modules (*the Classification module renames *Color by* to *Classify by* to form classification groups). For modules that come with point-based visualizations (e.g., scatterplot), hover-based interactions that show cell identifiers and visual channel widgets derived from categorical features are implemented. Each module also comes with built-in modeling methods, supported by custom interactive widgets.

class (Figure 2). Video S1 shows an example run of the Data Extraction pipeline.

Identify fields of view

Imaging sessions consist of multiple FOVs, each containing input files associated with potentially multiple channels (Figure 2A; Methods S1, Chapter 4). Per-channel and cross-channel settings are loaded from the configuration, and most can be updated at this stage, including the file suffixes for each input file type of each channel. FLIM Playground searches the user-specified folder to locate all input files for each FOV across channels based on user-configured file suffixes and reports a per-FOV status indicating whether all required files were found, missing, or duplicated. It then reads decay file metadata and verifies consistency across channels and with calibration files and ROI masks, including temporal (pulse interval and time bins) and spatial dimensions (STAR Methods). When a decay file contains multiple non-empty channels, the user is prompted to select the appropriate one. The resulting metadata contains one row per FOV, with columns recording file paths, channel numbers, spatial dimensions, pulse interval, time bins, imaging modality, and assigned feature extractors. It is previewed, cached for the next stage, and exported as a comma-separated values (CSV) file.

Extract single-cell numerical features

Using either the cached metadata file from the previous step or a newly uploaded file, FLIM Playground initiates numerical feature extraction as a two-step process: calibration followed by feature extraction (Figure 2B). Calibration is required whenever lifetime

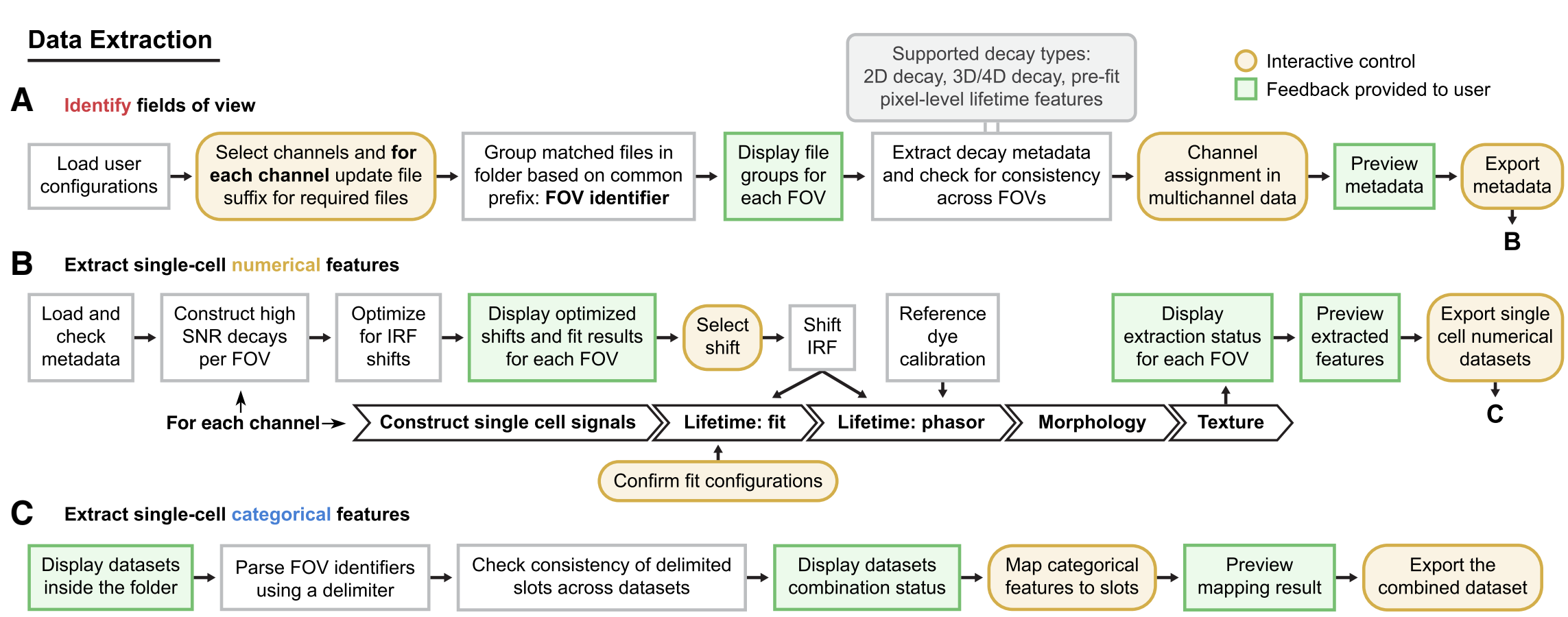

Figure 2. A high-level overview of the Data Extraction workflow

This section is divided into three stages (A, B, C). A short description of steps involved in each stage is shown here, with detailed descriptions provided in the following sections. The white boxes are internal processing steps not shown to the user. The yellow oval-shaped boxes are interactive controls the user can change. Green boxes are interfaces that provide feedback to the user.

fit or phasor analysis is selected, since the measured fluorescence decay is the convolution product of the underlying fluorescence decay with the IRF. Calibration is carried out separately for each channel.

Fit calibration: IRF time shifts are estimated through reconvolution fitting, where the shift is treated as a free parameter optimized jointly with lifetimes and amplitudes using high signal-to-noise (SNR) decay curves from each FOV (Methods S1, Chapter 5). For each FOV, high SNR decays are constructed by summing the decay curves of all non-background pixels defined by the ROI mask. The brightest cells are selected as high-SNR decays if the decay type is 2D decay. Reconvolution fitting models the measured fluorescence decay as a sum of exponential functions—reflecting the characteristic lifetimes of fluorophores—and fits this model after convolution with the IRF to account for temporal broadening introduced during acquisition (STAR Methods).

FLIM Playground supports fit models with up to three components. For multi-component models, users may optionally fix one or more lifetime parameters (τ_i), excluding them from optimization. This allows the incorporation of prior knowledge about specific decay components into the fitting procedure. Parameter optimization is performed by minimizing the difference between the fitted curve and the measured curve, quantified by either maximum likelihood estimation (MLE) or least squares as a *cost metric*.[14] The fitting procedure also allows users to specify *time gates*, restricting the optimization to a selected temporal window of the decay. FLIM Playground provides two *optimization modes* that balance speed against robustness to local minima. Local optimization applies the Levenberg-Marquardt[25] algorithm when the chosen metric is least squares, or the Nelder-Mead[26] algorithm otherwise. Hybrid optimization first applies the differential evolution algorithm,[27] a derivative-free, population-based global optimizer, to generate an initial guess, followed by local refinement. Fits are parallelized across CPU cores to accelerate processing.

For each high-SNR decay curve, a shift value is optimized and displayed as a point in an interactive scatterplot. By clicking on a point, the user can inspect the measured decay alongside the fitted curve, together with the fit metric and estimated parameters. This interface allows users to evaluate the fits, select a fixed shift to apply across all FOVs, or adjust the time gates to trigger re-optimization. Once the user confirms the *number of components* of the exponential model, *cost metric*, *fitting mode*, *time gates*, and *shifts* for all channels, these settings are stored in the metadata file and applied to extracting fit features for the entire dataset.

Phasor calibration: this step can be performed using either the IRF or a fluorescence lifetime standard. In IRF-based calibration, the temporal shift for each high-SNR decay is estimated by maximizing the cross-correlation between the IRF and the corresponding decay. Similar to fit-based calibration, all estimated shifts are plotted, and the user can select a fixed shift to apply across all decays. In addition to shifting the IRF by the chosen value, each decay curve is corrected by subtracting an offset—defined as the mean of the last 10% of the decay tail—and clipped to zero if negative values occur after subtraction. The raw phasor of the decay is divided by the phasor of the shifted IRF using complex division to obtain the calibrated phasor.

When using a fluorescence lifetime standard for calibration, the offset is subtracted from both the standard fluorophore decay and the decay curve, and their raw phasors are calculated. Phasor calibration is then performed using the PhasorPy[17] library, which applies to the raw decay phasor the same polar rotation and scaling transform that moves the raw standard fluorophore phasor to its correct location, yielding its calibrated phasor.

Feature extraction: for each channel, the selected feature extractors specified in the previous stage are applied to each cell ROI. A single-cell decay curve summed from all the decays of its pixels[19] can be fit with an exponential model, transformed into phasor coordinates, or both. Additionally, morphological and texture features can be computed from single-cell masks and intensity images.

The *Lifetime Fit* extractor (Methods S1, Chapter 6) applies reconvolution fitting to the decay curve of each cell using the shifted IRF, with fit settings specified by the user in the calibration step. In addition to saving the absolute amplitudes, it normalizes these amplitudes into fractions (α_i) and computes the mean lifetime (τ_m) (STAR Methods). It can also compute cell-level lifetime fit features when pre-fit pixel-level results are available, by averaging the pixel-level features within each cell ROI.

The *Lifetime Phasor* extractor (Methods S1, Chapter 7) provides a fast, model-free view of lifetimes by transforming fluorescence decays into phasor coordinates through a discrete Fourier transform. For each cell, raw phasors are computed from the fluorescence decay for the first and second harmonics of the laser pulse repetition rate, and they are calibrated using the procedures described under the heading *Phasor calibration* to obtain calibrated phasor coordinates for the first harmonic (g,s) and second harmonic (g_2, s_2). Phasor-derived lifetime features are also computed, including phase (ϕ), modulation (m), tau phase (τ_ϕ), and tau modulation (τ_M).[4,15,28]

The *Morphology* extractor (Methods S1, Chapter 8) computes single-cell morphological features of the cell ROI mask, including *area*, *perimeter*, *solidity*, *eccentricity*, *major axis length*, *minor axis length*, and *circularity*. The list can be readily extended.

The *Texture* extractor (Methods S1, Chapter 9) uses the single-cell ROI intensity image to extract texture features that can be readily extended. For example, *granularity*$_n$ is the percentage of intensity contributed by bright objects of n pixels in radius. It is computed by performing a morphological opening with a disk of radius n on the cell intensity image and measuring the intensity lost as a percentage of the total intensity of the original cell ROI. Five granularity-based features are calculated: n = 1, 3, 5, 7, 9. *Mass displacement* calculates the displacement, as Euclidean distance in pixels, of the intensity-weighted cell ROI centroid from the geometric centroid of the cell ROI. To calculate *radial distribution*, each cell intensity image is partitioned into four concentric rings, and the intensity fraction over the total intensity for each ring is calculated.

Each cell is assigned a unique identifier by concatenating its FOV identifier with the corresponding label from the ROI mask, and this identifier is used as the row index. Each row then aggregates features extracted from every channel, produced by their

respective feature extractors. The extraction status for each FOV is tracked with a progress bar, and the results are displayed for preview before being exported.

Extract single-cell categorical features

In this stage, FLIM Playground merges cell-level datasets produced by the numerical feature extraction stage into a unified table and assigns categorical features to each cell (Figure 2C; Methods S1, Chapter 10). FLIM Playground searches the user-designated folder for CSV files and verifies the uniqueness of cell identifiers. Datasets are then merged by aligning shared columns and concatenating rows. FOV identifiers are assumed to consist of segments delimited in a consistent manner, where each segment or combination of segments encodes a categorical feature (e.g., treatment and time point). Under this assumption, FLIM Playground parses the segments and presents them to the user, who interactively assigns them to categorical features with a live preview of the results. The resulting merged dataset, enriched with categorical features, can be exported for downstream analysis.

Data Analysis provides a shared interactive interface for diverse analysis methods

After single-cell datasets are generated—either through Data Extraction or by the user's own methods—the Data Analysis section provides a suite of analysis methods (Table 2) to help the user gain insights. To ensure users can seamlessly apply alternative analysis methods across datasets with varying features, Data Analysis offers a unified interface built on a library of interactive widgets shared across its analysis modules, allowing new modules to be added easily by reusing existing components. Video S2 shows a user iteratively exploring a dataset by switching among modules within the shared interface.

The shared interface

The shared interface supports common analysis tasks and is composed of interactive widgets built to operate on the three feature classes—identifiers, numerical features, and categorical features (Methods S1, Chapter 11). This class-based design also allows users to map features from external datasets (i.e., datasets not output by Data Extraction) to these classes in the analysis configuration interface, enabling their analysis within FLIM Playground (Video S3, Methods S1, Chapter 12).

Numerical feature selection widgets

Numerical features are grouped either by channel-extractor pairs or by custom groupings specified in the analysis configuration, making it faster to locate and select features. Univariate methods present one single-select widget per group; choosing a feature in any group clears the selections in the others. Bivariate methods present two such panels, with the first chosen feature excluded from the second panel. Multivariate methods present one multi-select widget per group, with an *All* option to quickly include every feature in that group.

Filter widgets

Categorical filter widgets, each linked to a categorical feature, allow users to select one or more labels to find data of interest, with an *All* option available to include every label in that feature. Users may also refine the dataset through numerical filter widgets. Each numerical filter is expressed as a condition composed of a selected feature, a comparison operator, and a threshold value. The permissible range of threshold values is displayed for reference and updates automatically in response to any filters already applied, ensuring that the user inputs values consistent with the currently visible subset of the data. Users may add multiple numerical conditions, each of which is evaluated conjunctively, allowing for compound constraints on continuous measurements. After both categorical and numerical filters are applied, the resulting subset of data is passed to all analysis modules.

Visual channel widgets

Visual channel widgets map categorical features to colors, shapes, and opacities in the visualizations. Users can select multiple categorical features in a *Color by* widget; each unique label combination partitions the filtered dataset, with a distinct color assigned to each partition. For example, if the filtered dataset contains three treatments and two cell lines, and both features are selected in the *Color by* widget, six non-overlapping treatment-cell line partitions will be created. If no feature is selected, all data are grouped into a single partition and displayed in the same color. In point-based visualizations, *Opacity by* and *Shape by* widgets allow a single categorical feature to be mapped so that each label is represented with a distinct opacity or shape. Interactive visualizations are implemented in Plotly,[29] enabling users to hover over individual points (cells) to view their cell and FOV identifiers.

Plot configuration widgets

Widgets are also provided to allow interactive adjustment of key visualization parameters, including point size in point-based visualizations, axis label size, legend size, and the colormap applied to distinguish groups.

Module-specific statistical methods

Custom widgets are implemented to support module-specific modeling, and adjustments to them trigger real-time reanalysis. All models are applied separately to each partition created by the Cartesian product of the categorical features selected in *Color by*. These partitions will hereafter be referred to as color groups. Library dependencies are listed in STAR Methods; full implementation details are provided in the referenced Methods S1 chapters.

Feature Comparison (Methods S1, Chapter 13): users can choose to compute effect sizes and/or perform statistical tests for each comparison pair on the selected feature and annotate the results, quantifying the difference and testing its significance. FLIM Playground offers two effect size calculation methods: Glass' delta[30] and Cohen's d,[31] and two statistical test options, Student's t test and Welch's t test.[32]

Available comparison pairs include all pairwise combinations of color groups. Because the number of pairs can grow combinatorially and many may be uninformative, users can restrict the annotations displayed in the visualization either by deselecting them in a selection widget or by setting a numerical threshold to show only annotations above a chosen value. The accompanying visualization comprises Sina plots for each color group, revealing single-cell heterogeneity and group-level distributions of the selected numerical feature, with filtered effect size

CellPress
OPEN ACCESS

annotations overlaid. Users can designate a categorical feature in the *Separate by* widget to generate sub-panels corresponding to the labels of the selected feature. The *Color by* widget then creates color groups, and comparisons are performed between pairs in each panel.

Feature Histogram (Methods S1, Chapter 14): the selected numerical feature is visualized on the *x*-axis, and the *y*-axis represents either the counts or densities estimated by the Gaussian mixture model[33] (GMM) of each color group. The counts are determined by the user-adjustable bin width of the histograms. The default bin width is determined automatically as the minimum of the Sturges'[34] and Freedman-Diaconis[35] estimators.

As an alternative to histograms, GMMs can capture multimodal distributions by representing a dataset as a weighted sum of Gaussian components, each with its component weights, means, and variances. FLIM Playground fits all GMMs up to the user-controlled maximum number of components, retains only those in which all components exceed the user-specified minimum weight, and selects the model with the lowest Bayesian information criterion[36] to prevent overfitting. To quantify the subpopulation structure of each color group from the GMM fit, a weighted entropy-based heterogeneity index can be computed and displayed.[37,38]

Each point can be assigned to a GMM component, which can then be exported and used as a categorical feature. In *Hard Assignment*, the posterior probability (responsibility) of each component is computed, and the data points are assigned to the component with the highest responsibility. Alternatively, *Intersection Thresholding* finds intersection points of adjacent component distributions using Brent's method[39] and assigns each point to the component whose region (bounded by these intersections) it falls into.

Feature Distribution (Methods S1, Chapter 15): the first selected feature is visualized on the *x* axis and the second on the *y* axis as a scatterplot. A two-dimensional GMM can be fit to each color group, following the same procedure as in *Feature Histogram*. The correlation coefficient and its *p* value are calculated, and a linear regression line can be fit using scikit-learn and displayed.

Phasor Analysis (Methods S1, Chapter 16): the phasor coordinates are plotted into the universal semicircle.[28] K-means clustering[40] is performed with a user-specified number of clusters, after standardizing the phasor coordinates since *g* (real part) and *s* (imaginary part) may differ in scale. A convex hull, a polygon that contains all the points of each cluster, is drawn for each cluster, and the cluster centroid is marked with its coordinates displayed.

Dimension Reduction (Methods S1, Chapter 17): selected numerical features are standardized and projected into two dimensions for visualization. Three algorithms are provided: principal-component analysis (PCA[41]), t-distributed stochastic neighbor embedding (t-SNE[42]), and uniform manifold approximation and projection (UMAP[43]). Since hyperparameters affect projection results, users can adjust perplexity and early exaggeration for t-SNE, and the number of neighbors and minimum distance for UMAP, while all other parameters are left at library defaults.

Classification (Methods S1, Chapter 18): users can select a classifier and specify the proportion of data allocated to training and testing. The visual channel widget *Color by* is renamed to *Classify by* and is used to form classes based on selected categorical features. Both binary and multi-class classification are supported, including one-versus-rest schemes for multi-class tasks. Machine learning classifiers, including Random Forest, Support Vector Machine, Logistic Regression, and Gradient Boosting, are provided. A dedicated hyperparameter control panel allows users to configure key hyperparameters for each classifier—covering model capacity, regularization, and optimization—directly in the GUI. Their performance is evaluated using accuracy, precision, recall, F1 score, and visual diagnostics including confusion matrices and receiver operating characteristic (ROC) curves with corresponding area under the curve (AUC). For decision tree-based models (Random Forest and Gradient Boosting), feature importance plots are also generated to rank the contribution of each feature.

To address class imbalance, three strategies are provided, all applied to the training set only to prevent data leakage. First, three sampling options are available: by default, stratified sampling that preserves the original class distribution is applied; alternatively, undersampling[44] reduces all classes to the size of the smallest class, and oversampling increases all classes to the size of the largest class by randomly duplicating samples from minority classes. Second, a balanced class weighting option assigns weights inversely proportional to class size, available for all classifiers except Gradient Boosting. Third, threshold tuning adjusts the post-training decision boundary to maximize either balanced accuracy or F1 score via stratified 5-fold cross-validation without altering the learned model.

Reproducible script export

A dedicated export button allows users to generate a standalone Python script that reproduces the current analysis end to end—from dataset loading through numerical feature selection, filtering, module-specific modeling, and visualization configurations—enabling reproducibility outside of the graphical interface and providing a starting point for further customization.

Fit validation

To validate the *Lifetime Fit* extractor in FLIM Playground, 25 NAD(P)H FLIM images of PANC-1 cancer cells[19] were fit using SPCImage (STAR Methods) and FLIM Playground. SPCImage yielded 6.40 time bins (or 307 ps) as the IRF shift, while the FLIM Playground produced 6.53 time bins (or 313 ps). The cost metric (MLE), number of exponential components (2), and time gates were matched between the two methods to ensure a fair comparison. The average photon count across all 505 cells was 4,626. Lifetimes and fractions of each component were obtained from fits performed with these two methods.

The comparisons of two representative lifetime features (τ_m, α_1) are shown in Figure 3. Although the mean of τ_m and α_1 calculated by FLIM Playground deviated from the SPCImage fit (Figures 3B and 3E) on average by −15.4 picoseconds (ps) (95% confidence interval [CI]: −16.7 ps, −14.2 ps) and 0.18% (95% CI: 0.1%, 0.2%), respectively, the effect sizes were small (Figures 3A and 3D), and the FLIM Playground values correlated consistently with the SPCImage values ($r = 0.99$ and $p < 0.001$). A regression line was fit for each of the two features,

whose 95% confidence interval for the slope included 1, indicating no evidence of proportional bias (Figures 3C and 3F).

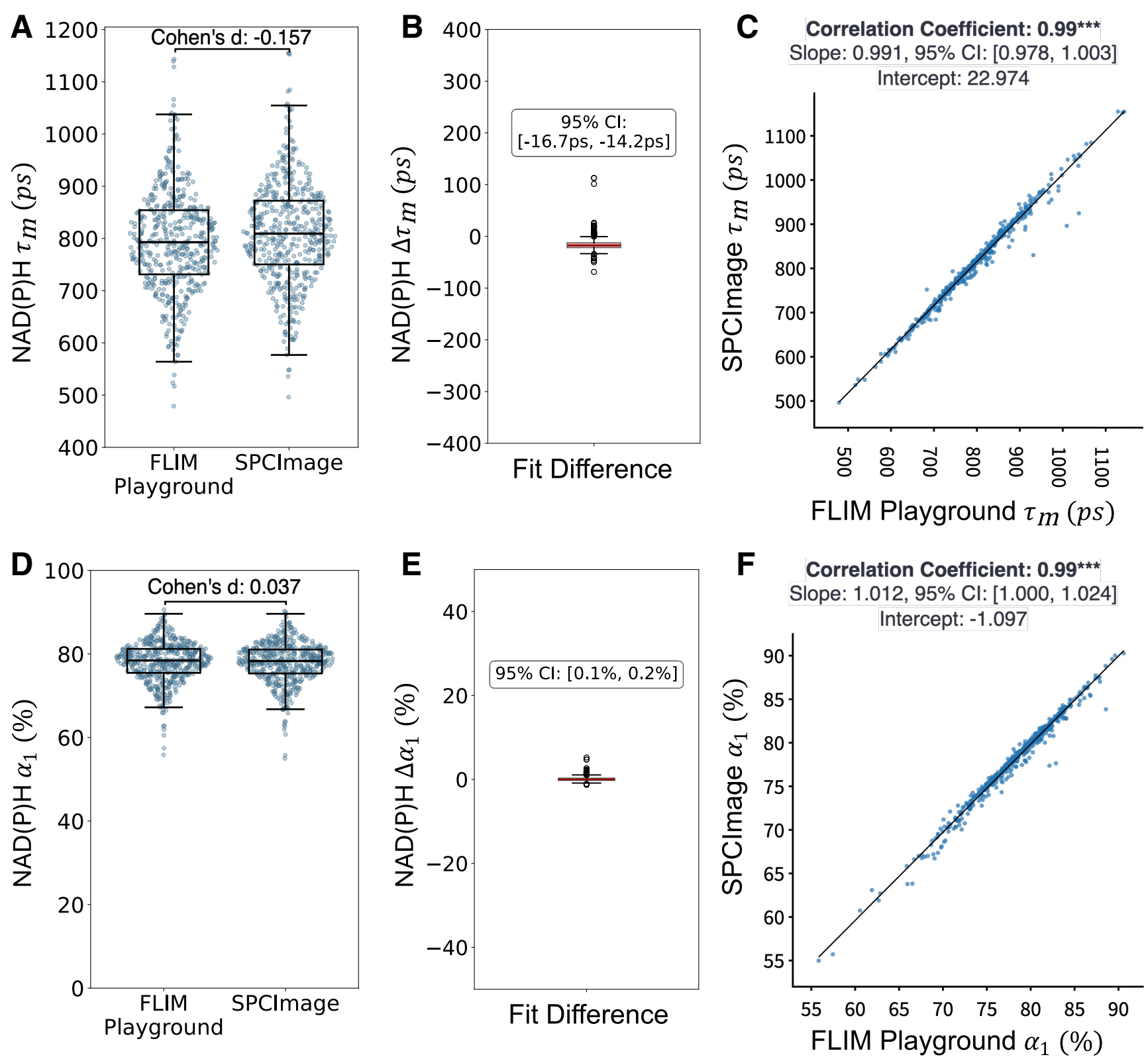

Figure 3. Fit comparison between FLIM Playground and commercial software (SPCImage)

The first row shows the comparison of the NAD(P)H mean lifetime (τ_m) calculated from FLIM Playground and SPCImage.

(A) Distributions of τ_m are shown for both methods, with Cohen's d calculated between their means.

(B) $\Delta\tau_m$ distribution with the 95% confidence interval (CI) annotated.

(C) A scatterplot annotated with the correlation coefficient between the τ_m values obtained from the two fit methods ($p < 0.001$), exported from the Feature Distribution module. A regression line (in black) was fit and plotted. Its slope, confidence interval, and intercepts are reported.

(D–F) The same visualizations and statistics for comparing the proportion of free NAD(P)H (α_1).

Discriminating inhibitor treatments in PANC-1 and MCF7 cells using FLIM Playground

To demonstrate the integrated workflow of FLIM Playground, the following FLIM dataset was acquired: three metabolic inhibitors—sodium cyanide (cyanide), 2-deoxy-*d*-glucose (2-DG), and iodoacetic acid (IAA)—were added to each of the two cell lines (PANC-1 human pancreatic and MCF7 human mammary gland adenocarcinoma), alongside a control condition, and NAD(P)H and FAD channel FLIM images were acquired along with IRFs. Whole-cell masks were made using CellProfiler[21] based on the NAD(P)H intensity images (STAR Methods). Analysis was performed in FLIM Playground, including extracting single-cell features and visualizing and applying models to those features.

The settings were specified using the configuration interface (Figure S1) and were kept consistent across subsequent stages. The FOV identification (Figure S2) and IRF calibration (Figure S3) stages were repeated for each day. Fits were performed in Local mode, and a bi-exponential decay model was applied to both channels with the same cost metric (MLE) and the same time gates ([49, 240]). The fit lifetime features, phasor features, morphology, and texture features were extracted for both channels (Figure S4). All extracted features are shown in Table S1.

The Data Extraction section was concluded by the categorical feature extraction stage, which merged the four single-cell numerical datasets, and assisted in assigning categories (cell line, treatment, and dish number) interactively to cells (Figure S5).

The combined dataset was uploaded to the Data Analysis section, and a subset of modules was applied to gain biological insights (Figures 4 and 5). In the *Feature Comparison* module, through the shared interactive interface, the dataset was filtered based on categories of interest and a numerical threshold (Figures 4A and 4B), and categorical features were assigned to visual channels (Figure 4C). Differences between groups were quantified using effect size calculations, where Glass' delta was calculated for selected comparison pairs (Figure 4D), and an effect size threshold was chosen as 0.7 (Figure 4E). This produced a visualization showing the distributions of NAD(P)H τ_m and τ_ϕ for different inhibitors across two cell lines, with replicates encoded in different opacities. The large overlap indicated that technical-replicate variability was relatively small. As expected, IAA increased the NAD(P)H τ_m and τ_ϕ compared to the control, whereas inhibition of oxidative phosphorylation by cyanide decreased NAD(P)H τ_m and τ_ϕ for both cell lines (Figures 4F and 4G).

The PCA from the *Dimension Reduction* module was demonstrated on MCF7 cells, using all features except single-cell intensity sum to ensure independence from acquisition-related intensity differences (Figure 5A). The separation between control and 2-DG was less distinct, consistent with the small effect size observed between these groups for both τ_m and τ_ϕ. UMAP provided a clearer separation between treatments in PANC-1 cells using the same feature set (Figures 5B and 5C): the two glycolysis inhibitors (2-DG and IAA) clustered on one side, while the oxidative phosphorylation inhibitor (cyanide) localized to the opposite region of the embedding space, and the control lay in the middle.

Whereas dimension reduction methods provided a qualitative view of treatment separation, the *Classification* module was used to quantify the discriminative power of the features. To discriminate between cyanide, the oxidative phosphorylation inhibitor, and glycolysis inhibitors (2-DG and IAA), "cyanide versus the rest" was selected in the classification options list

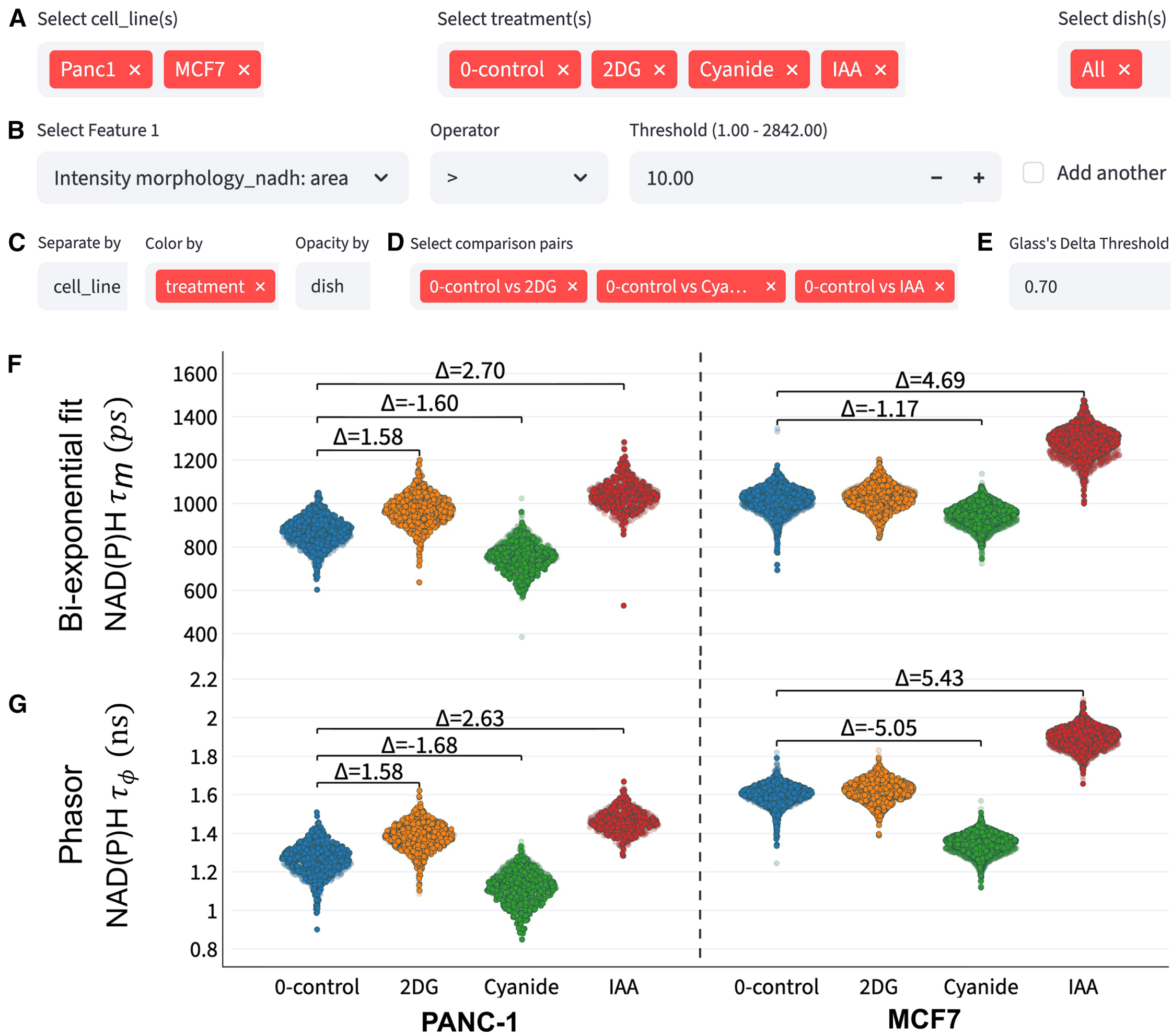

Figure 4. Applying the Feature Comparison module to compare features across categories

(A) Filter widgets were rendered for each categorical feature. Two cell lines, three inhibitors plus the control, and both dishes were selected.

(B) A lower-bound threshold (10 pixels) was applied to cell area, a numerical feature.

(C) Data separated by cell line, each generating one sub-panel; within each panel, treatments were color coded. Different opacities indicate variability between dishes.

(D and E) (D) Three comparison pairs, control versus each inhibitor, were selected, and (E) only effect sizes, Δ (Glass' delta) exceeding the user-specified threshold (0.7) were shown.

(F) NAD(P)H τ_m was compared between the inhibitors and the control, separated by cell line (left: PANC-1, right: MCF7). Cell counts for PANC-1: control, n = 889; 2-DG, n = 950; cyanide, n = 974; IAA, n = 645. For MCF7: control, n = 1,689; 2-DG, n = 1,572; cyanide, n = 2,551; IAA, n = 1,705.

(G) Same comparisons plotted for NAD(P)H tau phase (τ_ϕ), a phasor-derived lifetime feature.

(Figure 5D). The train-test split was 70% and 30% (Figure 5E). Since the classes (cyanide versus 2-DG + IAA) were not substantially imbalanced, the default stratified sampling was used. All classifiers achieved an AUC of 1.0 on the ROC curves (Figure 5F), with the near-diagonal confusion matrix (of the Random Forest classifier, Figure 5G), indicating strong discriminative ability of cyanide versus the glycolysis inhibitors (2-DG and IAA).

Analysis of fluorescence lifetime flow cytometry data using FLIM Playground

To validate FLIM Playground, we reanalyzed a previously published dataset of primary human T cells acquired by fluorescence lifetime flow cytometry.[18] This is an example of a dataset without imaging, but with single-cell decays. The data comprise two-dimensional decays in a tabular format: rows are individual cells and columns are successive time bins of the decay. Consistent with the reported results, activated T cells exhibited shorter mean NAD(P)H lifetimes (τ_m) and a higher fractional contribution of the short component (α_1) compared to quiescent cells. The phasor distribution reproduced the clear separation between activation states, and UMAP embeddings of lifetime fit and phasor features likewise resolved two distinct clusters. Random Forest classification based on FLIM Playground-extracted features achieved accuracy and AUC values comparable to those previously

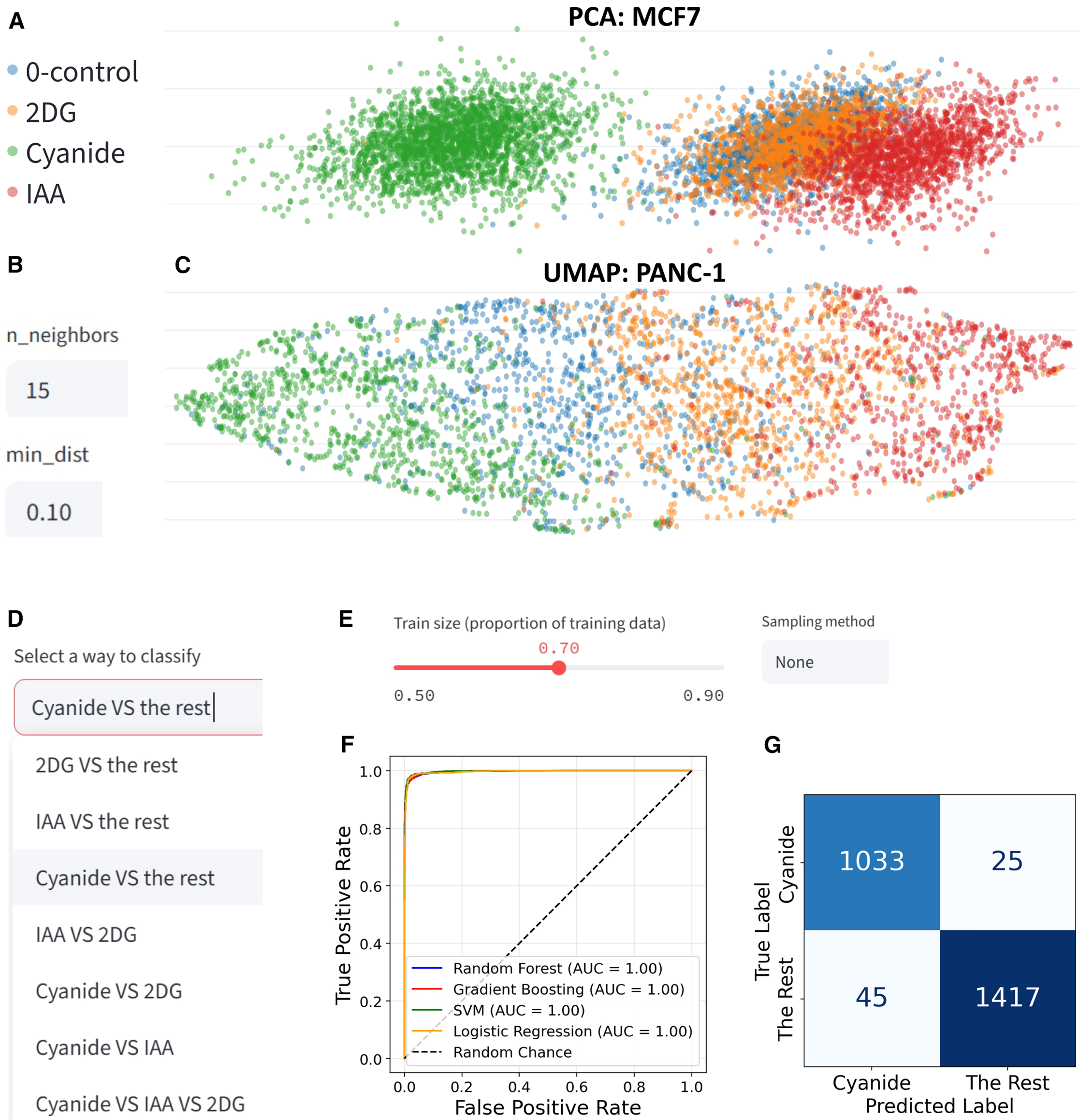

Figure 5. Applying the Dimension Reduction module and the Classification module to differentiate inhibitor treatments

(A) PCA was performed on MCF7 cells (axes were truncated).

(B and C) (B) Interactive widgets for specifying UMAP hyperparameters (*n_neighbors* = 15, *min_dist* = 0.1); (C) UMAP of PANC-1 cells.

(D) List of all classification options; cyanide versus the rest (two glycolysis inhibitors: 2-DG and IAA) was selected.

(E) Train-test split: 70% (5,877 cells) training and 30% (2,520 cells) testing and the sampling method used (None: default stratified sampling).

(F) ROC curves generated from FLIM Playground classifiers. All classifiers were trained on the same numerical feature set used for UMAP.

(G) Confusion matrix of the Random Forest classifier evaluated on unseen data, showing the number of cells within true-positive (upper left), false-negative (upper right), false-positive (lower left), and true-negative (lower right) categories.

reported, confirming that the framework faithfully reproduces published lifetime fit, phasor, and analysis results (Figure S6).

Cross-system validation of lifetime analysis

To assess lifetime analysis consistency across hardware configurations, we imaged a lifetime standard green fluorescent slide (FLIM Labs, Rome, Italy) on three system configurations in two laboratories (Table S2). Decay curves were fit in both FLIM Playground and the corresponding commercial software, with results shown in Figure S7.

DISCUSSION

FLIM Playground is an open-source, interactive platform that unifies metadata organization, calibration, single-cell feature extraction, visualization, and statistical modeling through an intuitive and code-free graphical interface. In current practice,

CellPress
OPEN ACCESS

FLIM analysis involves switching between a fragmented set of tools and processing their outputs to obtain biologically interpretable features and insights, often requiring custom code and expertise. FLIM Playground overcomes this limitation by mapping the hierarchical levels of FLIM data to three semantically meaningful feature classes—identifiers, numerical features, and categorical features (Table 1).

The Data Extraction section adopts a channel-centric framework, in which each channel can be assigned its own extraction methods. Notably, extraction is not limited to lifetime features: morphological and texture descriptors are also supported, enabling a richer characterization of cellular phenotypes than is currently available in existing tools and extending feature extraction to non-FLIM imaging modalities. In addition to single-cell numerical features, identifiers and categorical features are also incorporated into a seamless three-stage extraction interface. Beyond single cells, this framework accommodates any segmented ROI—for instance, organoids—whose boundaries are defined by ROI masks and labeled with unique ROI identifiers.

The Data Analysis section then transforms these three feature classes of single-cell datasets—whether produced by Data Extraction or imported as user-provided data (including from other fields)—into a unified interface shared across visualization and analysis methods built on the Python ecosystem. This interface allows users to explore datasets from multiple perspectives—inspecting individual cells, filtering data of interest, and visualizing features through visual encodings that leverage the human visual system's capacity for rapid, parallel detection of patterns and trends, revealing insights that raw numbers or text may obscure.[45] The interface further couples with module-specific analysis methods and their corresponding interactive components, enabling specialized statistical modeling to reveal biological insights.

To ensure reliability, we benchmarked lifetime fits in FLIM Playground against the widely used commercial package SPCImage and demonstrated that the two yielded consistently correlated lifetimes with small differences. We further assessed analysis consistency across multiple hardware configurations. Additionally, to demonstrate the integration and its capabilities, we used FLIM Playground to run the complete FLIM analysis pipeline—from feature extraction through analysis—on representative two-photon NAD(P)H and FAD FLIM images of cancer cell lines treated with metabolic inhibitors. FLIM Playground was also demonstrated on a fluorescence lifetime flow cytometry dataset of primary human T cells composed of 2D decays (i.e., non-imaging measurements). FLIM Playground successfully discriminated between different inhibitors and activation states, respectively.

FLIM Playground makes advanced FLIM analysis accessible to non-computational practitioners through its intuitive interface, while its modular design facilitates improvements by specialists. The platform can be expanded to incorporate additional acquisition modalities, feature descriptors, fit-free methods, and analysis modules, supporting a range of applications in quantitative imaging. By unifying extraction, visualization, and analysis in a single platform, FLIM Playground supports biologically meaningful discoveries at the single-cell level and at scale.

Limitations of the study

At present, the software is designed for time-correlated single-photon counting-acquired data; however, support for other time-domain and frequency-domain acquisition modalities could be implemented in future versions. Similarly, additional fit-free methods, such as Laguerre deconvolution,[4] may be incorporated to broaden the feature extraction repertoire. Fitting is currently limited to the ROI level due to computational speed constraints, making pixel-level fitting impractical, although FLIM Playground can accept pixel-level prefit lifetime features or allow users to perform cell-level fitting, which improves accuracy and precision for cell-level analysis.[19] Although pixel-level phasor calculation, visualization, and texture descriptors of phasor feature maps are technically feasible, they have not yet been implemented. The extraction stage can also be expanded to include a wider range of morphological and texture feature descriptors. Finally, the current version does not support time-lapse or volumetric FLIM or object tracking. Numerical features that capture relationships across time, such as velocity, and their correlation with lifetime features will provide valuable insights and will be considered in future developments. Planned extensions to Data Analysis include hierarchical clustering for exploratory grouping, linear mixed-effects models to account for biological and technical variability, and modality alignment methods that integrate FLIM-derived numerical features with data from other omics or imaging modalities.

RESOURCE AVAILABILITY

Lead contact

Requests for further information and resources should be directed to and will be fulfilled by the lead contact, Rupsa Datta (rdatta@morgridge.org).

Materials availability

- This study did not generate new unique reagents.

Data and code availability

- All raw data necessary to reanalyze the data reported the "Results" section has been deposited at Zenodo at doi:10.5281/zenodo.19774942.
- All original code has been deposited at Zenodo at doi:10.5281/zenodo.19744706 and is also publicly available at https://github.com/skalalab/flim_playground.
- Any additional information is available from the lead contact upon request.

ACKNOWLEDGMENTS

The authors would like to thank all members of the Skala lab for their suggestions, as well as those who kindly proofread the online manual. We are also grateful to Matthew Stefely for designing and creating the FLIM Playground logo and editing figures, Alicia Williams for editing the manuscript, and Dr. Jenu Chacko and Dr. Kevin Eliceiri for providing access to instrument systems at the Eliceiri lab (Laboratory for Optical and Computational Instrumentation, UW-Madison) for cross-system fluorescence lifetime measurements. Funding: this work was funded by NIH R01 CA278051, NIH R01 CA272855, NIH R01 HL165726, NIH R37 CA226526, and NSF 2426316.

AUTHOR CONTRIBUTIONS

Conceptualization, W.Z., K.S., M.C.S., and R.D.; methodology, W.Z. and K.S.; investigation, W.Z. and R.D.; formal analysis, W.Z. and K.S.; funding acquisition, M.C.S.; writing – original draft preparation, W.Z. and R.D.; writing – review & editing, W.Z., K.S., M.C.S., and R.D.

DECLARATION OF INTERESTS

M.C.S. is an adviser to Elephas Biosciences. All other authors declare they have no competing interests.

STAR★METHODS

Detailed methods are provided in the online version of this paper and include the following:

- KEY RESOURCES TABLE
- EXPERIMENTAL MODEL AND STUDY PARTICIPANT DETAILS
- METHOD DETAILS
  - Identify fields of view
  - Reconvolution fitting
  - Lifetime fit extractor
  - Two-photon fluorescence lifetime imaging microscopy of cells
  - Single-cell segmentation
  - SPCImage analysis
- QUANTIFICATION AND STATISTICAL ANALYSIS
  - Module-specific statistical methods

SUPPLEMENTAL INFORMATION

Supplemental information can be found online at https://doi.org/10.1016/j.crmeth.2026.101484.

Received: December 19, 2025
Revised: April 1, 2026
Accepted: May 12, 2026
Published: June 10, 2026

REFERENCES

1. Bastiaens, P.I., and Squire, A. (1999). Fluorescence lifetime imaging microscopy: spatial resolution of biochemical processes in the cell. Trends Cell Biol. *9*, 48–52. https://doi.org/10.1016/S0962-8924(98)01410-X.
2. J.R. Lakowicz, ed. (2006). Principles of Fluorescence Spectroscopy (Springer US). https://doi.org/10.1007/978-0-387-46312-4.
3. Chang, C., Sud, D., and Mycek, M. (2007). Fluorescence Lifetime Imaging Microscopy. In Methods in Cell Biology Digital Microscopy, 3rd Edition (Academic Press), pp. 495–524. https://doi.org/10.1016/S0091-679X(06)81024-1.
4. Datta, R., Heaster, T.M., Sharick, J.T., Gillette, A.A., and Skala, M.C. (2020). Fluorescence lifetime imaging microscopy: fundamentals and advances in instrumentation, analysis, and applications. JBO *25*, 1–43. https://doi.org/10.1117/1.JBO.25.7.071203.
5. Torrado, B., Pannunzio, B., Malacrida, L., and Digman, M.A. (2024). Fluorescence lifetime imaging microscopy. Nat. Rev. Methods Primers *4*, 80. https://doi.org/10.1038/s43586-024-00358-8.
6. Schmidt, U., Weigert, M., Broaddus, C., and Myers, G. (2018). Cell Detection with Star-Convex Polygons. In Medical Image Computing and Computer Assisted Intervention – MICCAI 2018 (Cham: Springer), pp. 265–273. https://doi.org/10.1007/978-3-030-00934-2_30.
7. Stringer, C., and Pachitariu, M. (2025). Cellpose3: one-click image restoration for improved cellular segmentation. Nat. Methods *22*, 592–599. https://doi.org/10.1038/s41592-025-02595-5.
8. Sofroniew, N., Lambert, T., Bokota, G., Nunez-Iglesias, J., Sobolewski, P., Sweet, A., Gaifas, L., Evans, K., Burt, A., Doncila Pop, D., et al. (2025). Napari: A Multi-Dimensional Image Viewer for Python (Zenodo). https://doi.org/10.5281/zenodo.16883660.
9. Riendeau, J.M., Gillette, A.A., Guzman, E.C., Cruz, M.C., Kralovec, A., Udgata, S., Schmitz, A., Deming, D.A., Cimini, B.A., and Skala, M.C. (2025). Cellpose as a reliable method for single-cell segmentation of autofluorescence microscopy images. Sci. Rep. *15*, 5548. https://doi.org/10.1038/s41598-024-82639-6.
10. Shirshin, E.A., Shirmanova, M.V., Gayer, A.V., Lukina, M.M., Nikonova, E.E., Yakimov, B.P., Budylin, G.S., Dudenkova, V.V., Ignatova, N.I., Komarov, D.V., et al. (2022). Label-free sensing of cells with fluorescence lifetime imaging: The quest for metabolic heterogeneity. Proc. Natl. Acad. Sci. USA *119*, e2118241119. https://doi.org/10.1073/pnas.2118241119.
11. Pham, D.L., Gillette, A.A., Riendeau, J., Wiech, K., Guzman, E.C., Datta, R., and Skala, M.C. (2025). Perspectives on label-free microscopy of heterogeneous and dynamic biological systems. JBO *29*, S22702. https://doi.org/10.1117/1.JBO.29.S2.S22702.
12. Warren, S.C., Margineanu, A., Alibhai, D., Kelly, D.J., Talbot, C., Alexandrov, Y., Munro, I., Katan, M., Dunsby, C., and French, P.M.W. (2013). Rapid Global Fitting of Large Fluorescence Lifetime Imaging Microscopy Datasets. PLOS ONE *8*, e70687. https://doi.org/10.1371/journal.pone.0070687.
13. Gao, D., Barber, P.R., Chacko, J.V., Kader Sagar, M.A., Rueden, C.T., Grislis, A.R., Hiner, M.C., and Eliceiri, K.W. (2020). FLIMJ: An open-source ImageJ toolkit for fluorescence lifetime image data analysis. PLoS One *15*, e0238327. https://doi.org/10.1371/journal.pone.0238327.
14. Becker, W. (2026). The Bh TCSPC Handbook, 11th ed. (Becker & Hickl GmbH). https://www.becker-hickl.com/literature/documents/flim/the-bh-tcspc-handbook-11th-edition-2026/.
15. Ranjit, S., Malacrida, L., Jameson, D.M., and Gratton, E. (2018). Fit-free analysis of fluorescence lifetime imaging data using the phasor approach. Nat. Protoc. *13*, 1979–2004. https://doi.org/10.1038/s41596-018-0026-5.
16. Gottlieb, D., Asadipour, B., Kostina, P., Ung, T.P.L., and Stringari, C. (2023). FLUTE: A Python GUI for interactive phasor analysis of FLIM data. Biol. Imaging *3*, e21. https://doi.org/10.1017/S2633903X23000211.
17. Gohlke, C., Pannunzio, B., Schüty, B., and Blanco, R. (2025). phasorpy/phasorpy: v0.7. (Zenodo). https://doi.org/10.5281/zenodo.16923774.
18. Samimi, K., Pasachhe, O., Guzman, E.C., Riendeau, J., Gillette, A.A., Pham, D.L., Wiech, K.J., Moore, D.L., and Skala, M.C. (2024). Autofluorescence lifetime flow cytometry with time-correlated single photon counting. Cytometry. A. *105*, 607–620. https://doi.org/10.1002/cyto.a.24883.
19. Samimi, K., Desa, D.E., Zhang, X., Pham, D.L., Datta, R., and Skala, M.C. (2025). Segmentation-guided photon pooling enables robust single cell analysis and fast fluorescence lifetime imaging microscopy. bioRxiv. 2025.09.30.679660. https://doi.org/10.1101/2025.09.30.679660.
20. Kapsiani, S., Läubli, N.F., Ward, E.N., Shehata, M., Kaminski, C.F., and Kaminski Schierle, G.S. (2025). FLIMPA: A Versatile Software for Fluorescence Lifetime Imaging Microscopy Phasor Analysis. Anal. Chem. *97*, 11382–11387. https://doi.org/10.1021/acs.analchem.5c00495.
21. Stirling, D.R., Swain-Bowden, M.J., Lucas, A.M., Carpenter, A.E., Cimini, B.A., and Goodman, A. (2021). CellProfiler 4: improvements in speed, utility and usability. BMC Bioinf. *22*, 433. https://doi.org/10.1186/s12859-021-04344-9.
22. Streamlit Inc. (2019). Streamlit: A faster way to build and share data apps. https://streamlit.io/.
23. PyInstaller Development Team (2024). PyInstaller: Freeze (package) Python programs into stand-alone executables. https://github.com/pyinstaller/pyinstaller.
24. Zhao, W. (2025). FLIM Playground. https://skalalab.github.io/flim_playground_doc/.
25. Marquardt, D.W. (1963). An Algorithm for Least-Squares Estimation of Nonlinear Parameters. Journal of the Society for Industrial and Applied Mathematics *11*, 431–441.
26. Nelder, J.A., and Mead, R. (1965). A Simplex Method for Function Minimization. Comput J *7*, 308–313. https://doi.org/10.1093/comjnl/7.4.308.
27. Storn, R., and Price, K. (1997). Differential Evolution – A Simple and Efficient Heuristic for global Optimization over Continuous Spaces. J. Global Optim. *11*, 341–359. https://doi.org/10.1023/A:1008202821328.

CellPress
OPEN ACCESS

28. Digman, M.A., Caiolfa, V.R., Zamai, M., and Gratton, E. (2008). The Phasor Approach to Fluorescence Lifetime Imaging Analysis. Biophys. J. *94*, L14–L16. https://doi.org/10.1529/biophysj.107.120154.
29. Plotly Technologies Inc. (2015). Collaborative data science. https://plot.ly.
30. Glass, G.V. (1976). Primary, Secondary, and Meta-Analysis of Research. Educ. Res. *5*, 3–8. https://doi.org/10.2307/1174772.
31. Cohen, J. (2013). Statistical Power Analysis for the Behavioral Sciences, 2nd ed. (Routledge). https://doi.org/10.4324/9780203771587.
32. Welch, B.L. (1947). The Generalization of 'Student's' Problem when Several Different Population Variances are Involved. Biometrika *34*, 28–35. https://doi.org/10.2307/2332510.
33. Dempster, A.P., Laird, N.M., and Rubin, D.B. (1977). Maximum Likelihood from Incomplete Data via the EM Algorithm. J. Roy. Stat. Soc. B *39*, 1–22.
34. Sturges, H.A. (1926). The Choice of a Class Interval. J. Am. Stat. Assoc. *21*, 65–66. https://doi.org/10.1080/01621459.1926.10502161.
35. Freedman, D., and Diaconis, P. (1981). On the histogram as a density estimator: L2 theory. Z. Wahrscheinlichkeitstheorie verw. Gebiete *57*, 453–476. https://doi.org/10.1007/BF01025868.
36. Schwarz, G. (1978). Estimating the Dimension of a Model. Ann. Statist. *6*, 461–464. https://doi.org/10.1214/aos/1176344136.
37. Sharick, J.T., Walsh, C.M., Sprackling, C.M., Pasch, C.A., Pham, D.L., Esbona, K., Choudhary, A., Garcia-Valera, R., Burkard, M.E., McGregor, S.M., et al. (2020). Metabolic Heterogeneity in Patient Tumor-Derived Organoids by Primary Site and Drug Treatment. Front. Oncol. *10*, 553. https://doi.org/10.3389/fonc.2020.00553.
38. Datta, R., Miskolci, V., Gallego-López, G.M., Britt, E., Gillette, A., Kralovec, A., Giese, M.A., Qian, T., Votava, J., Zhao, W., et al. (2025). Single cell autofluorescence imaging reveals immediate metabolic shifts of neutrophils with activation across biological systems. Front. Immunol. *16*. https://doi.org/10.3389/fimmu.2025.1617993.
39. Brent, R.P. (2013). Algorithms for Minimization without Derivatives. (Courier Corporation).
40. Lloyd, S. (1982). Least squares quantization in PCM. IEEE Trans. Inf. Theor. *28*, 129–137. https://doi.org/10.1109/TIT.1982.1056489.
41. Pearson, K. (1901). LIII. On lines and planes of closest fit to systems of points in space. London, Edinburgh Dublin Phil. Mag. J. Sci. *2*, 559–572. https://doi.org/10.1080/14786440109462720.
42. Maaten, L. van der, and Hinton, G. (2008). Visualizing Data using t-SNE. J. Mach. Learn. Res. *9*, 2579–2605.
43. McInnes, L., Healy, J., Saul, N., and Großberger, L. (2018). UMAP: Uniform Manifold Approximation and Projection. J. Open Source Softw. *3*, 861. https://doi.org/10.21105/joss.00861.
44. Lemaître, G., Nogueira, F., and Aridas, C.K. (2017). Imbalanced-learn: A Python Toolbox to Tackle the Curse of Imbalanced Datasets in Machine Learning. J. Mach. Learn. Res. *18*, 1–5.
45. Cleveland, W.S., and McGill, R. (1984). Graphical Perception: Theory, Experimentation, and Application to the Development of Graphical Methods. J. Am. Stat. Assoc. *79*, 531–554. https://doi.org/10.1080/01621459.1984.10478080.
46. Newville, M., Otten, R., Nelson, A., Stensitzki, T., Ingargiola, A., Allan, D., Fox, A., Carter, F., and Rawlik, M. (2025). LMFIT: Non-linear Least-Squares Minimization and Curve-Fitting for Python (Zenodo). https://doi.org/10.5281/zenodo.16175987.
47. Gohlke, C. (2025). Cgohlke/Sdtfile: v2025.3.25 (Zenodo). https://doi.org/10.5281/zenodo.15086209.
48. Gohlke, C. (2025). Cgohlke/Ptufile: v2025.5.10 (Zenodo). https://doi.org/10.5281/zenodo.15380998.
49. Virtanen, P., Gommers, R., Oliphant, T.E., Haberland, M., Reddy, T., Cournapeau, D., Burovski, E., Peterson, P., Weckesser, W., Bright, J., et al. (2020). SciPy 1.0: fundamental algorithms for scientific computing in Python. Nat. Methods *17*, 261–272. https://doi.org/10.1038/s41592-019-0686-2.
50. Harris, C.R., Millman, K.J., van der Walt, S.J., Gommers, R., Virtanen, P., Cournapeau, D., Wieser, E., Taylor, J., Berg, S., Smith, N.J., et al. (2020). Array programming with NumPy. Nature *585*, 357–362. https://doi.org/10.1038/s41586-020-2649-2.
51. Pedregosa, F., Varoquaux, G., Gramfort, A., Michel, V., Thirion, B., Grisel, O., Blondel, M., Prettenhofer, P., Weiss, R., Dubourg, V., et al. (2011). Scikit-learn: Machine Learning in Python. J. Mach. Learn. Res. *12*, 2825–2830.
52. Jones, T.R., Carpenter, A., and Golland, P. (2005). Voronoi-Based Segmentation of Cells on Image Manifolds. In Computer Vision for Biomedical Image Applications, Y. Liu, T. Jiang, and C. Zhang, eds. (Springer), pp. 535–543. https://doi.org/10.1007/11569541_54.
53. Walsh, A.J., and Skala, M.C. (2014). An automated image processing routine for segmentation of cell cytoplasms in high-resolution autofluorescence images. In Multiphoton Microscopy in the Biomedical Sciences XIV (SPIE), pp. 161–166. https://doi.org/10.1117/12.2040644.

STAR★METHODS

KEY RESOURCES TABLE

REAGENT or RESOURCE	SOURCE	IDENTIFIER
Chemicals, peptides, and recombinant proteins		
Sodium cyanide (NaCN)	Fisher Scientific	Cat# AC370310050
2-Deoxy-*d*-glucose (2-DG)	Sigma-Aldrich	Cat# 154-17-6
Iodoacetic acid (IAA)	Sigma-Aldrich	Cat# I4386
Urea crystals (used to generate IRF via SHG)	Sigma-Aldrich	Cat# U4883
Deposited data		
Raw FLIM data of PANC-1 and MCF7 cells with metabolic inhibitors	This paper; Zenodo	https://doi.org/10.5281/zenodo.19774942
Cross-system green slide FLIM measurements	This paper; Zenodo	https://doi.org/10.5281/zenodo.19774942
Fluorescence lifetime flow cytometry dataset of primary human T cells	Samimi et al.[18]; Zenodo	https://doi.org/10.5281/zenodo.19774942
NAD(P)H FLIM images of PANC-1 cells	Samimi et al.[19]; Zenodo	https://doi.org/10.5281/zenodo.19774942
Experimental models: Cell lines		
Human: PANC-1 (pancreatic carcinoma)	ATCC	RRID: CVCL_0480
Human: MCF7 (mammary gland adenocarcinoma)	ATCC	RRID: CVCL_0031
Software and algorithms		
FLIM Playground v1.6.0	This paper	https://github.com/skalalab/flim_playground; https://doi.org/10.5281/zenodo.19744707
FLIM Playground online manual	Zhao[25]	https://skalalab.github.io/flim_playground_doc/
SPCImage 8.9.0	Becker & Hickl GmbH[14]	https://www.becker-hickl.com/products/spcimage/
Streamlit v1.54.0	Streamlit Inc.[22]	https://pypi.org/project/streamlit/1.54.0/
PyInstaller v6.14.2	PyInstaller Development Team[23]	https://pypi.org/project/pyinstaller/6.14.2/
Plotly v6.2.0	Plotly Technologies Inc.[29]	https://pypi.org/project/plotly/6.2.0/
lmfit v1.3.4	Newville et al.[46]	https://doi.org/10.5281/zenodo.16175987
PhasorPy v0.7	Gohlke et al.[17]	https://doi.org/10.5281/zenodo.16923774
sdtfile v2025.3.25	Gohlke[47]	https://doi.org/10.5281/zenodo.15086209
ptufile v2025.9.9	Gohlke[48]	https://doi.org/10.5281/zenodo.17089195
CellProfiler 4	Stirling et al.[21]	https://cellprofiler.org/releases
Cellpose v3.0.11	Stringer and Pachitariu[7]	https://github.com/MouseLand/cellpose
Napari v0.5.5	Sofroniew et al.[8]	https://zenodo.org/records/14427406
SciPy v1.15.2	Virtanen et al.[49]	https://doi.org/10.5281/zenodo.14880408
NumPy v2.2.4	Harris et al.[50]	https://pypi.org/project/numpy/2.2.4/
scikit-learn v1.8.0	Pedregosa et al.[51]	https://doi.org/10.5281/zenodo.17880109
umap-learn v0.5.11	McInnes et al.[43]	https://pypi.org/project/umap-learn/0.5.11/
imbalanced-learn v0.14.1	Lemaître et al.[44]	https://pypi.org/project/imbalanced-learn/0.14.1/
Python $\geq$3.11	Python Software Foundation	https://www.python.org
Other		
Standard green fluorescent slide (cross-system validation)	FLIM Labs	https://www.flimlabs.com/

EXPERIMENTAL MODEL AND STUDY PARTICIPANT DETAILS

PANC-1 human pancreatic (ATCC, RRID: CVCL_0480) and MCF7 human mammary gland adenocarcinoma (ATCC, RRID: CVCL_0031) cells were cultured in high-glucose Dulbecco's modified Eagle's medium supplemented with 10% fetal bovine serum (Gibco) and 1% penicillin/streptomycin (Gibco). Cell lines were regularly tested for mycoplasma contamination. Cells were maintained at 37°C and 5% CO_2.

METHOD DETAILS

Identify fields of view

FLIM Playground recursively searches the user-specified folder for files ending in the first suffix of the first channel. Matched filenames, with this suffix removed, serve as FOV identifiers, under the assumption that *all files of a FOV share the same prefix*. All other files across channels are then located by appending each suffix to the FOV identifier. The only exception is calibration files, which are not FOV-specific and are therefore searched based solely on their file suffixes. For each FOV, the search status is reported. A status of *Success* indicates that all required files were located, and the FOV is then recorded into a metadata file together with the paths to its files. If a required file type is missing, its type and expected filename are displayed. A status of *Duplicate* is shown when multiple files with the same name are detected.

FLIM Playground then reads the metadata of all decay files. The temporal laser pulse interval and the number of time bins per pulse interval are cross-checked across all channels for consistency. The time bins are also validated against the lifetime axis of the calibration files. In addition, the spatial dimensions of the decay files are cross-checked with the ROI masks to ensure consistency. For each acquisition channel, if the corresponding decay file contains more than one non-empty channel, the user is prompted to select the appropriate channel number. The sdtfile[47] and ptufile[48] Python libraries are used to read decay data from Becker & Hickl and PicoQuant files.

Reconvolution fitting

The time axis (t) of the exponential function is defined by time bin width (Δt), which is the ratio between the laser pulse period (T) and the number of time bins (N) (Equation 1).

$$t = [0, \Delta t, 2\Delta t, (N-1)\Delta t] = T/N. \qquad \text{(Equation 1)}$$

A linear convolution (*) is performed between the multi-exponential model (A_i are amplitudes, τ_i are lifetimes of each component) and the shifted IRF (IRF_s) with truncation (0:N) (Equation 2). The constant offset parameter (Z) that represents a time-independent background is also optimized.[12] Together, this yields the fit curve ($\hat{\mathbf{y}}$).

$$\hat{\mathbf{y}} = \left(\sum_{i=1}^{n} A_i\, e^{-\mathbf{t}/\tau_i} * \mathbf{IRF}_s\right)_{0:N} + Z \qquad \text{(Equation 2)}$$

The cost metric chosen by the user (MLE or LS) is fed into lmfit,[46] a generic Python curve-fitting library, to be minimized (maximizing MLE is mathematically equivalent to minimizing the negative log likelihood function).

Lifetime fit extractor

In addition to saving the absolute amplitudes (A_i), it normalizes these amplitudes into fractions (α_i) (Equation 3) and computes the mean lifetime (τ_m) (Equation 4):

$$\alpha_i = \frac{A_i}{\sum_{j=1}^{n} A_j} \qquad \text{(Equation 3)}$$

$$\tau_m = \sum_{i=1}^{n} \alpha_i \tau_i \qquad \text{(Equation 4)}$$

Two-photon fluorescence lifetime imaging microscopy of cells

2×10^5 cells were plated on 35 mm glass-bottom dishes (MatTek) 48 h prior to imaging. The following metabolic inhibitors were added to the dish as indicated prior to imaging—sodium cyanide (NaCN; inhibits oxidative phosphorylation) (Fisher Scientific): 4 mM for 15 min; 2-Deoxy-*d*-glucose (2-DG; inhibits glycolysis) (Sigma-Aldrich): 10 mM for 2 h; iodoacetic acid (IAA; inhibits glycolysis) (Sigma-Aldrich): 1.5 mM for 30 min. FLIM was performed on a custom-made Ultima Multiphoton Imaging System (Bruker) that consists of an inverted microscope (TI-E, Nikon). The system is coupled to an ultrafast tunable laser source (Insight DS+, Spectra Physics Inc). The fluorescence lifetime images were acquired using time-correlated single-photon counting (TCSPC) electronics (SPC-150,

Becker & Hickl GmbH) and imaging was performed using Prairie View Software (Bruker). NAD(P)H and FAD were sequentially excited using excitation wavelengths of 750 nm and 890 nm respectively with the laser power <10 mW at the sample. The samples were illuminated using a 40× objective lens (W.I./1.15 NA/Nikon PlanApo) with a pixel dwell time of 4.8 μs and frame integration of 60 s at 256 × 256 pixels. The photon count rates were maintained at 1–5 × 10^5 photons/second to ensure adequate photon detection for accurate lifetime decay fits. A 720 nm dichroic mirror and bandpass filters separated fluorescence signals from the excitation laser. Emission filters used were bandpass 460/80 nm for NAD(P)H and 500/100 nm for FAD. Fluorescence signals were collected on GaAsP photomultiplier tubes (H7422P-40, Hamamatsu, Japan). The IRF was collected each day by recording the second harmonic generation signal of urea crystals (Sigma-Aldrich) excited at 890 nm. For each condition, data were acquired from two replicates, and three to four images were acquired per replicate per condition.

Single-cell segmentation

Single-cell segmentation of whole-cell masks was performed using either a customized CellProfiler pipeline or the Cellpose algorithm applied to NAD(P)H fluorescence intensity images. The CellProfiler pipeline, previously validated for both 2D cell and 3D organoid images, began with the manual identification of nuclei as primary objects, which served as seed regions for segmentation. Subsequently, whole-cell boundaries were delineated automatically using a Voronoi-based segmentation approach.[52,53] Alternatively, automated whole-cell masks were generated using Cellpose[7] (model = cyto3, radius = 30). The resulting masks were then visually inspected and manually refined in Napari[8] to ensure accurate delineation of individual cells.

SPCImage analysis

To enable fitting using the SPCImage software (Becker & Hickl GmbH) at the cell level, preprocessing was required: for each cell, the decays of all pixels within the same cell were summed. The resulting cell-level decay was then propagated back to replace the decays of all pixels belonging to that cell, so that the pixel-fitting routine by SPCImage could be applied to fit cell-level decays.[19,47] Because all pixels of a given cell now shared the same decay curve, the binning factor was set to zero. The IRF time-shift was selected manually from one FOV by clicking on representative pixels containing cell decays; upon selection, SPCImage fits the decay and outputs the corresponding shift value. The median shift across those pixels was chosen and applied to all FOVs.

QUANTIFICATION AND STATISTICAL ANALYSIS

Module-specific statistical methods

Feature Comparison

Statistical tests are implemented using the *scipy.stats.ttest_ind* function from SciPy. Student's *t*-test assumes equal variances between the two groups, while Welch's *t*-test generalizes the test to unequal variances (specified via equal_var=False).

Feature Histogram

The default histogram bin width is computed with *numpy.histogram* function from NumPy. GMM fitting is performed with scikit-learn's *sklearn.mixture.GaussianMixture* class, using its *fit* method to estimate model parameters with the expectation maximization algorithm and its *bic* method to compute the Bayesian Information Criterion for model selection. For Hard Assignment, posterior probabilities are computed using the *predict_proba* method, and each data point is assigned to the component with the highest posterior probability. For Intersection Thresholding, intersection points between adjacent component distributions are located using SciPy's scipy.optimize.brentq function.

Feature Distribution

Two-dimensional GMM fitting uses scikit-learn's *sklearn.mixture.GaussianMixture* class, as in Feature Histogram. The correlation coefficient and its *p*-value are computed with SciPy's *scipy.stats.pearsonr*. Linear regression is fit using scikit-learn's *sklearn.linear_model.LinearRegression*.

Phasor Analysis

Phasor coordinates are standardized with scikit-learn's *sklearn.preprocessing.StandardScaler* prior to clustering, because g and s may differ in scale. K-means clustering is performed using scikit-learn's *sklearn.cluster.KMeans* with the user-specified number of clusters. Convex hulls are computed with SciPy's *scipy.spatial.ConvexHull*.

Dimension Reduction

Features are standardized with scikit-learn's *sklearn.preprocessing.StandardScaler*. PCA and t-SNE are implemented with scikit-learn's *sklearn.decomposition.PCA* and *sklearn.manifold.TSNE*. UMAP is implemented with the umap-learn library (*umap.UMAP*). User-adjustable hyperparameters are perplexity and early exaggeration for t-SNE, and the number of neighbors and minimum distance for UMAP; non-adjustable parameters use scikit-learn and umap-learn defaults, respectively.

Classification

Classifiers are implemented with scikit-learn: Random Forest (*sklearn.ensemble.RandomForestClassifier*), Gradient Boosting (*sklearn.ensemble.GradientBoostingClassifier*), Logistic Regression (*sklearn.linear_model.LogisticRegression*), and Support Vector Machine (*sklearn.svm.SVC* with a linear kernel and probability=True). Logistic Regression and SVM are wrapped in a pipeline with *sklearn.preprocessing.StandardScaler* so that features are standardized before model fitting. Logistic Regression supports L1, L2, elasticnet, and unregularized configurations, selected via the l1_ratio and inverse-regularization parameter C. Stratified train–test

CellPress
OPEN ACCESS

splitting is performed with *sklearn.model_selection.train_test_split*. Oversampling and undersampling are implemented with *imblearn.over_sampling.RandomOverSampler* and *imblearn.under_sampling.RandomUnderSampler* from the imbalanced-learn library. Balanced class weighting is implemented using the *class_weight='balanced'* option of Random Forest, Logistic Regression, and SVM, which assigns weights inversely proportional to class frequencies; Gradient Boosting does not support this option. Threshold tuning is implemented as a custom generalization of scikit-learn's *TunedThresholdClassifierCV* that supports both binary and multi-class classification: for binary problems a single decision threshold is tuned, and for multi-class problems per-class thresholds are tuned jointly, with each class's probability normalized by its threshold and the predicted class taken as the argmax of the normalized probabilities. Thresholds are optimized using the Nelder–Mead algorithm (*scipy.optimize.minimize*) with bounds [0, 1], maximizing either balanced accuracy or macro-averaged F1 score via 5-fold stratified cross-validation (*sklearn.model_selection.StratifiedKFold*). ROC curves and AUC are computed with *sklearn.metrics.roc_curve* and *sklearn.metrics.auc*; for multi-class problems, one-vs-rest binarization is performed with *sklearn.preprocessing.label_binarize*. Confusion matrices are computed with *sklearn.metrics.confusion_matrix*; precision, recall, specificity, Youden's J, F1 score, accuracy, and balanced accuracy are computed from the confusion matrix. Feature importances for tree-based models are obtained from the classifier's *feature_importances_* attribute. A fixed random seed is used throughout to ensure reproducibility.

Cell Reports Methods, Volume *6*

Supplemental information

FLIM Playground: An interactive, end-to-end graphical user interface for analyzing single cells with fluorescence lifetime imaging microscopy

Wenxuan Zhao (赵文轩), Kayvan Samimi, Melissa C. Skala, and Rupsa Datta

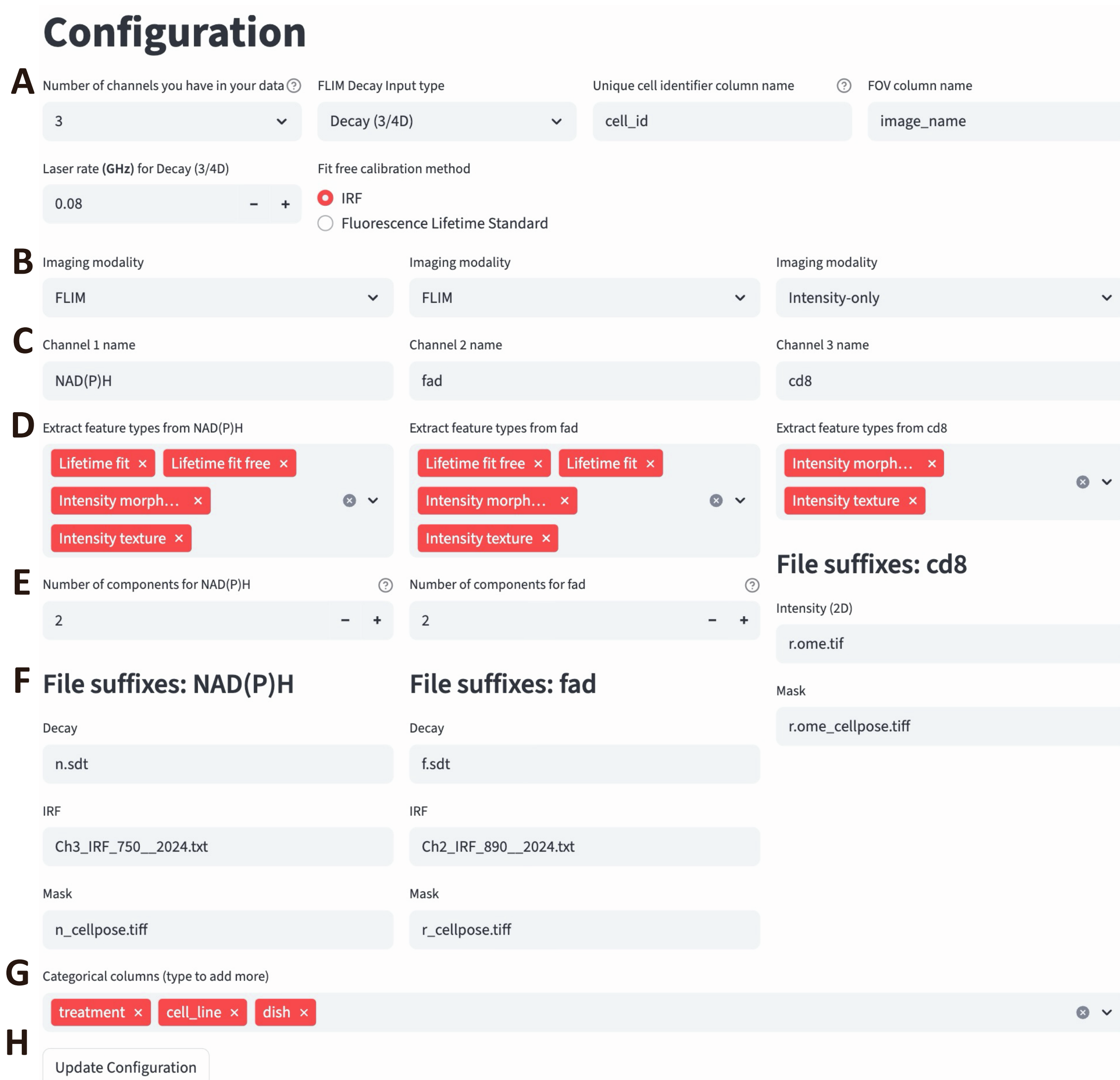

Figure S1. An example of the configuration interface, related to Figure 2. FLIM Playground loads the previously saved configurations and displays them for users to update. **(A)** The first two rows show configurations applied to all acquisition channels: number of channels, decay type, names of the columns that store the unique cell identifiers and field-of-view identifiers, laser frequency, and calibration method for extracting phasor features. Based on the specified number of channels, one column is rendered for each channel that stores channel-level configurations: **(B)** imaging modality, **(C)** channel name, **(D)** feature extractors, **(E)** number of exponential components if Lifetime Fit extractor is selected, and **(F)** file suffixes for each required input file type. Different channels can apply different feature extractors and focus on different cell ROIs. **(G)** Categorical features to be extracted are specified here. **(H)** The configuration can be saved for future use by clicking the update button.

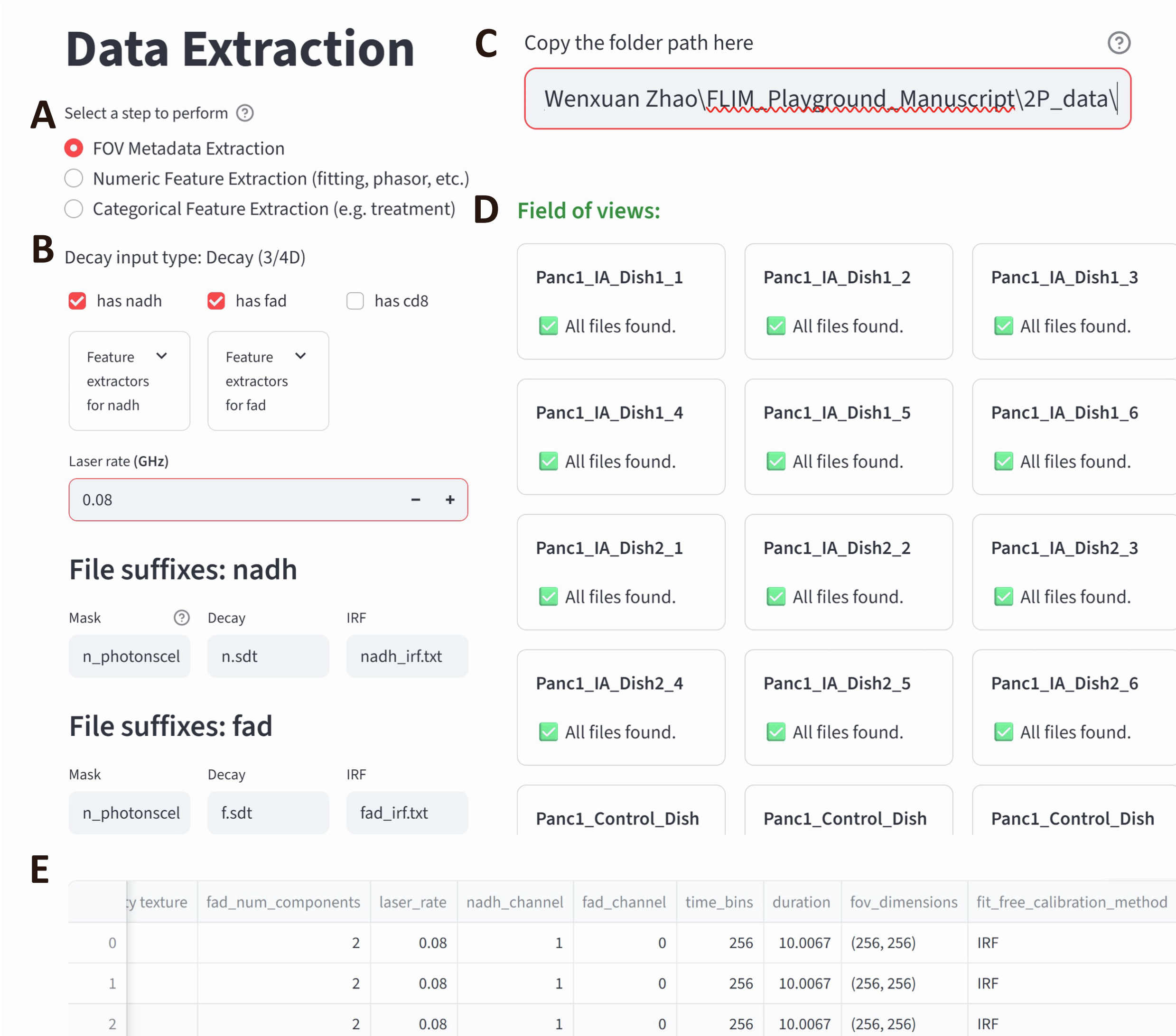

	...y texture	fad_num_components	laser_rate	nadh_channel	fad_channel	time_bins	duration	fov_dimensions	fit_free_calibration_method
0		2	0.08	1	0	256	10.0067	(256, 256)	IRF
1		2	0.08	1	0	256	10.0067	(256, 256)	IRF
2		2	0.08	1	0	256	10.0067	(256, 256)	IRF

Figure S2. Identify FOVs of inhibitor-treated PANC-1 cells, related to Figure 2. (A) This was the first of the three stages of the Data Extraction section. **(B)** The parameters were retrieved from a saved file updated by the configuration interface (Figure S1), and the third acquisition channel was deselected. **(C)** After the user specified the folder path containing all the required files, **(D)** a widget card was rendered for each FOV showing the file lookup status (some FOVs were cut off due to space limitations). **(E)** In addition to the file paths, file metadata and other configurations were checked and saved.

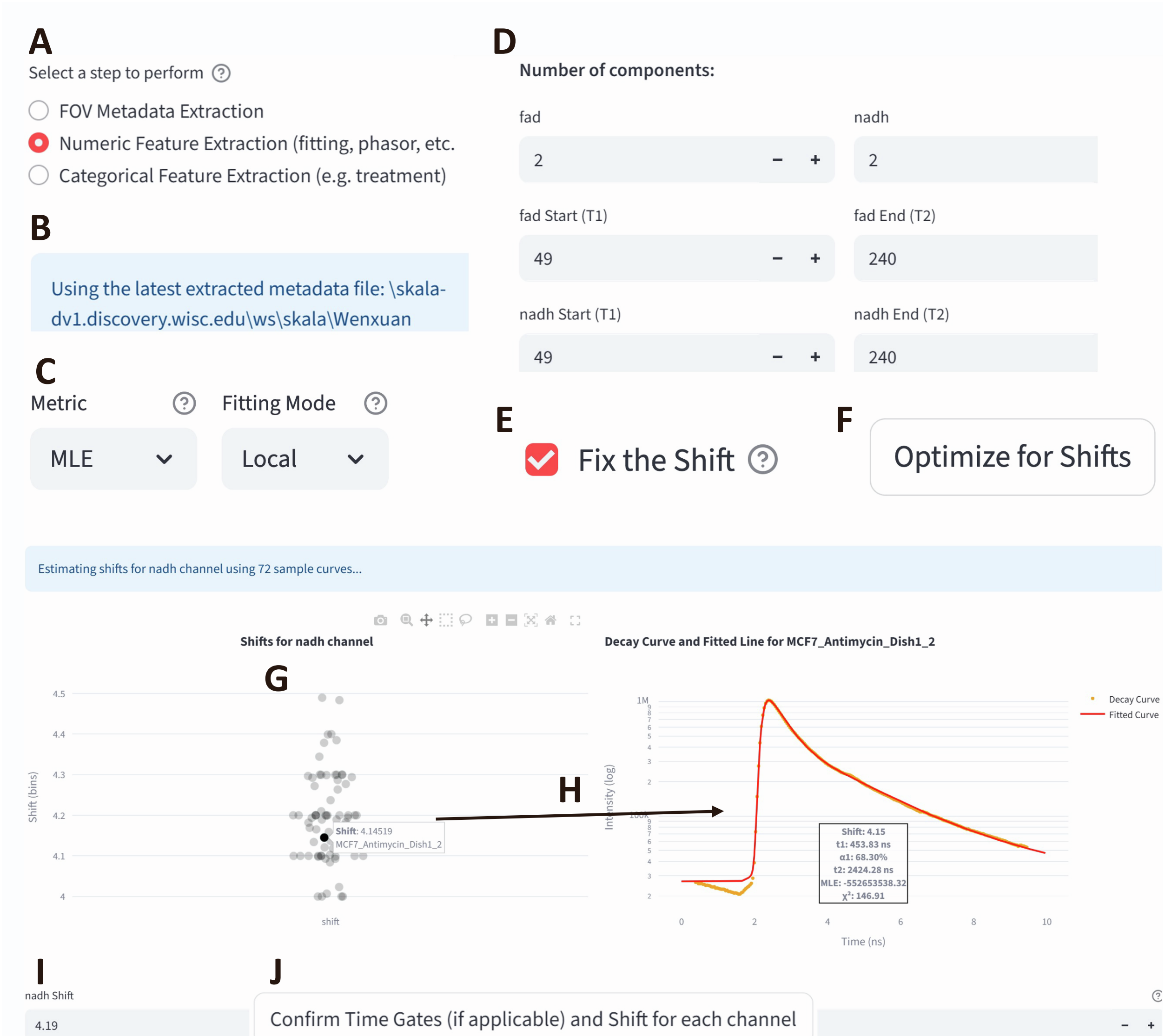

Figure S3. IRF calibration on FOVs of inhibitor-treated PANC-1 and MCF7 cells, related to Figure 2. A calibration interface was displayed for the first step of the **(A)** numerical feature extraction stage. **(B)** It loaded the latest extracted data automatically from the cache. **(C)** The chosen cost metric and the fitting mode, **(D)** number of exponential components, and the time gates of the reconvolution fitting were displayed. **(E)** *Fix the Shift* was chosen, so the selected shifts were applied to all FOVs. Once all configurations were confirmed, the user started fitting for shifts on high-SNR decays by clicking the **(F)** *Optimize for shifts* button. **(G)** The distribution of optimized shifts was plotted, allowing the user to hover and click on individual points to view **(H)** the fitted curve alongside the decay curve with diagnostic plots. Users could hover over the decay curve to display the corresponding time bin number, which helped in selecting appropriate time gates. **(I)** The shift selector was automatically populated with the median shift of the distribution, which was selected in this case. Steps **(G–I)** were repeated for the FAD channel as well. The shifts, time gates, and other fitting settings were confirmed by clicking the **(J)** *confirm* button.

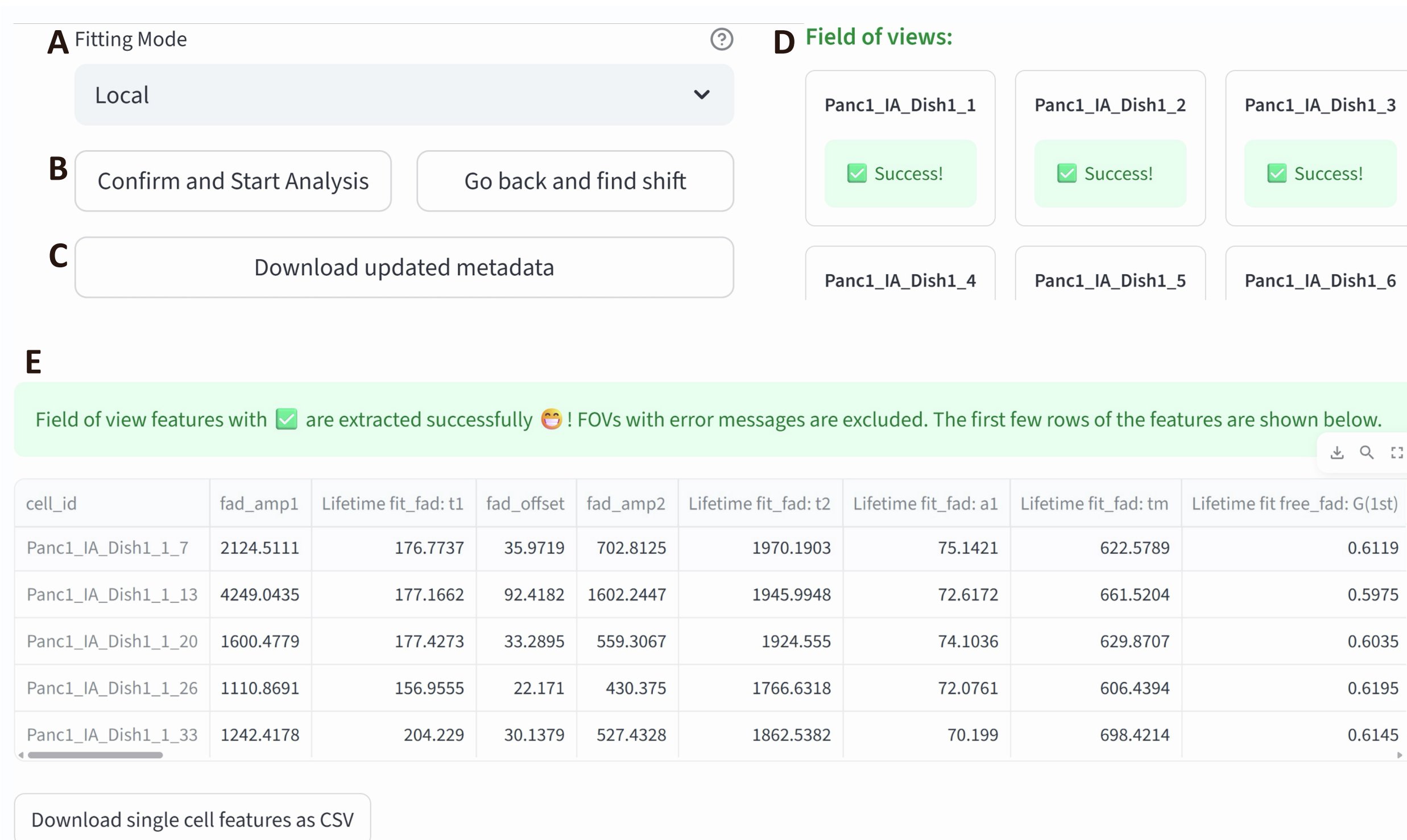

Figure S4. Numerical feature extraction for FOVs of inhibitor-treated PANC-1 cells, related to Figure 2. (A) Users could choose a different fitting mode after IRF calibration. **(B)** They could either confirm the shifts and fitting configurations or choose to go back and recalibrate. **(C)** They could update the FOV metadata file with the configuration values. **(D)** After starting the analysis, the selected feature extractors of each acquisition channel were applied to each FOV. The extraction status was displayed, **(E)** and the preview of the extracted dataset was shown.

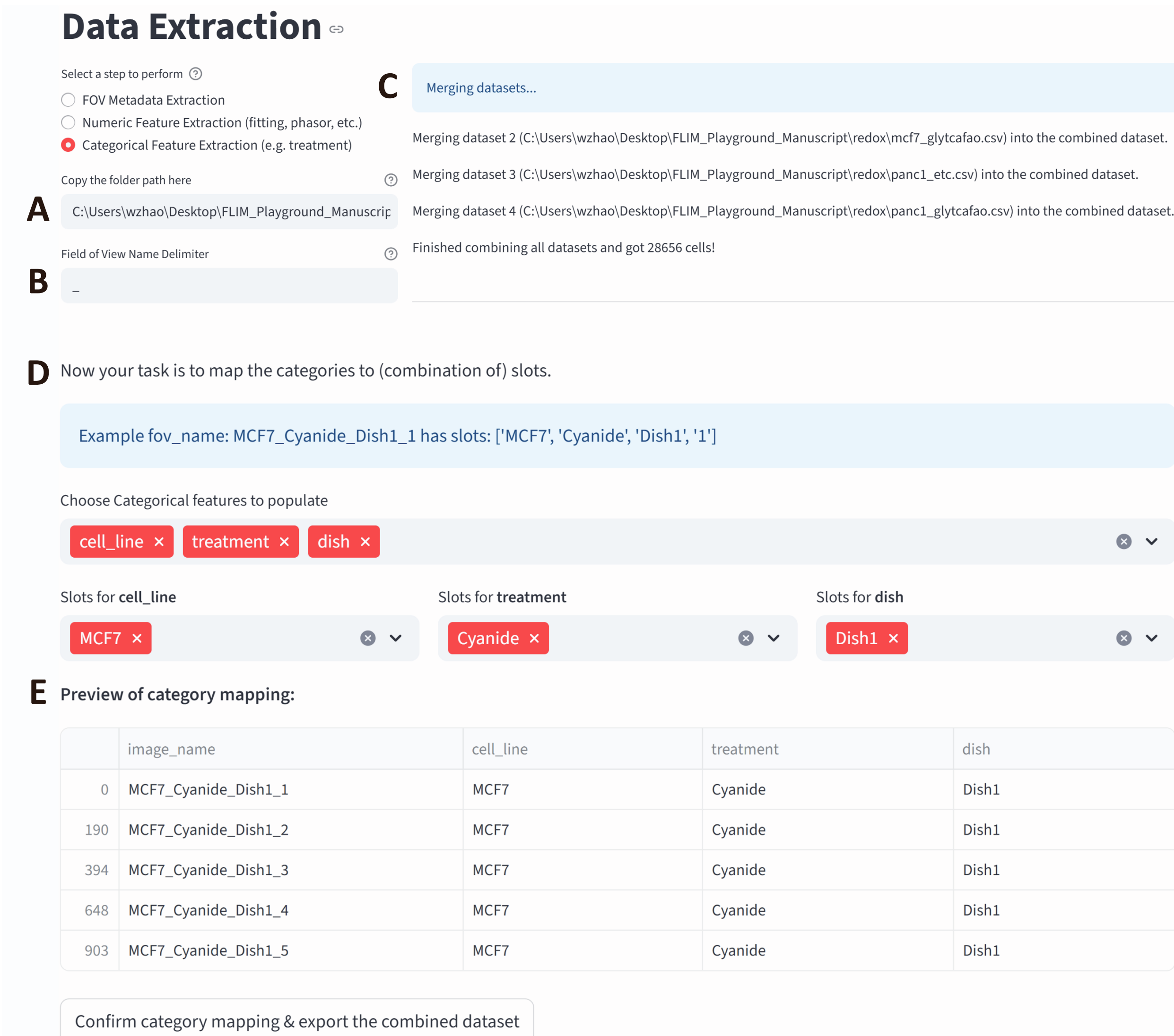

Figure S5. Categorical feature extraction of PANC-1 and MCF7 single-cell numerical datasets, related to Figure 2. (A) The folder path containing single-cell numerical datasets extracted by the previous stages of Data Extraction was pasted into the text box. **(B)** The FOV identifiers were segmented by the user-chosen delimiter. **(C)** Datasets were merged and the merging status was displayed. **(D)** FLIM Playground parsed the FOV identifiers and interactively assisted the user in assigning categorical features to each cell. **(E)** A live preview of such assignment was displayed, and the merged dataset, annotated with categorical features, was exported.

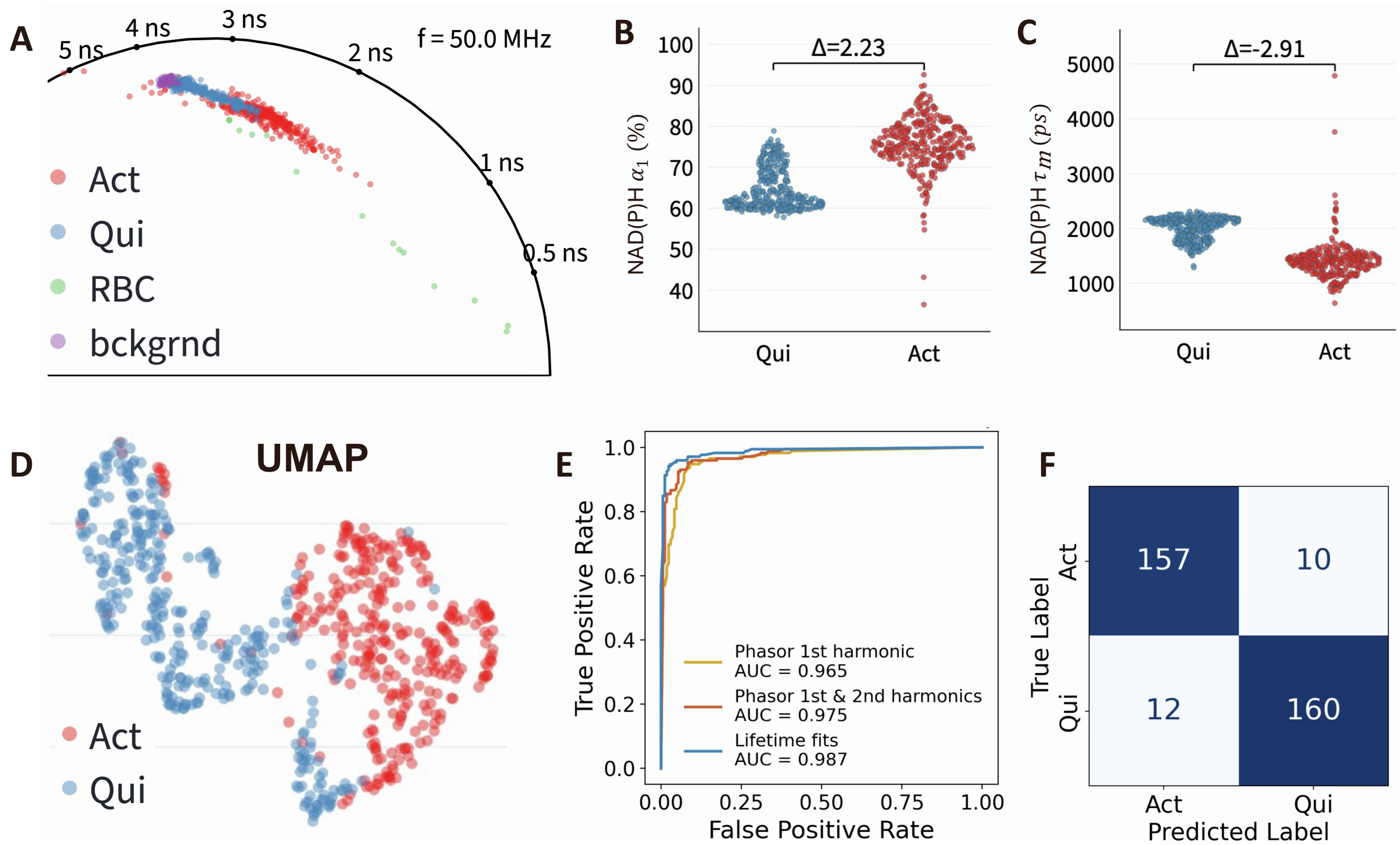

Figure S6. Discriminating quiescent versus activated primary human T cells, related to Table 2. (A) Phasor representation of NAD(P)H lifetime from the primary human T cells measured in flow. The activated cells cluster separately from the control quiescent cells and present with a shorter fluorescence lifetime. Red blood cells (RBCs) have shorter fluorescence lifetimes, and background (bckgrnd) has a longer fluorescence lifetime. Activated cells have an **(B)** increased proportion of free (α_1) and **(C)** shorter NAD(P)H mean lifetime (τ_m). **(D)** UMAP projection of the NAD(P)H lifetime fit parameters (τ_m, τ_1, τ_2, α_1) and phasors (1st, 2nd harmonics) shows separate clustering of the quiescent and activated T cells. **(E)** Random Forest classifiers trained on NAD(P)H phasor or lifetime parameters (50% training; 50% testing split) can predict the activation state of the T cells with good accuracy as demonstrated by the area under the curve (AUC) of the receiver operating characteristic (ROC) curves. **(F)** Confusion matrix for the classifier based on NAD(P)H phasors.

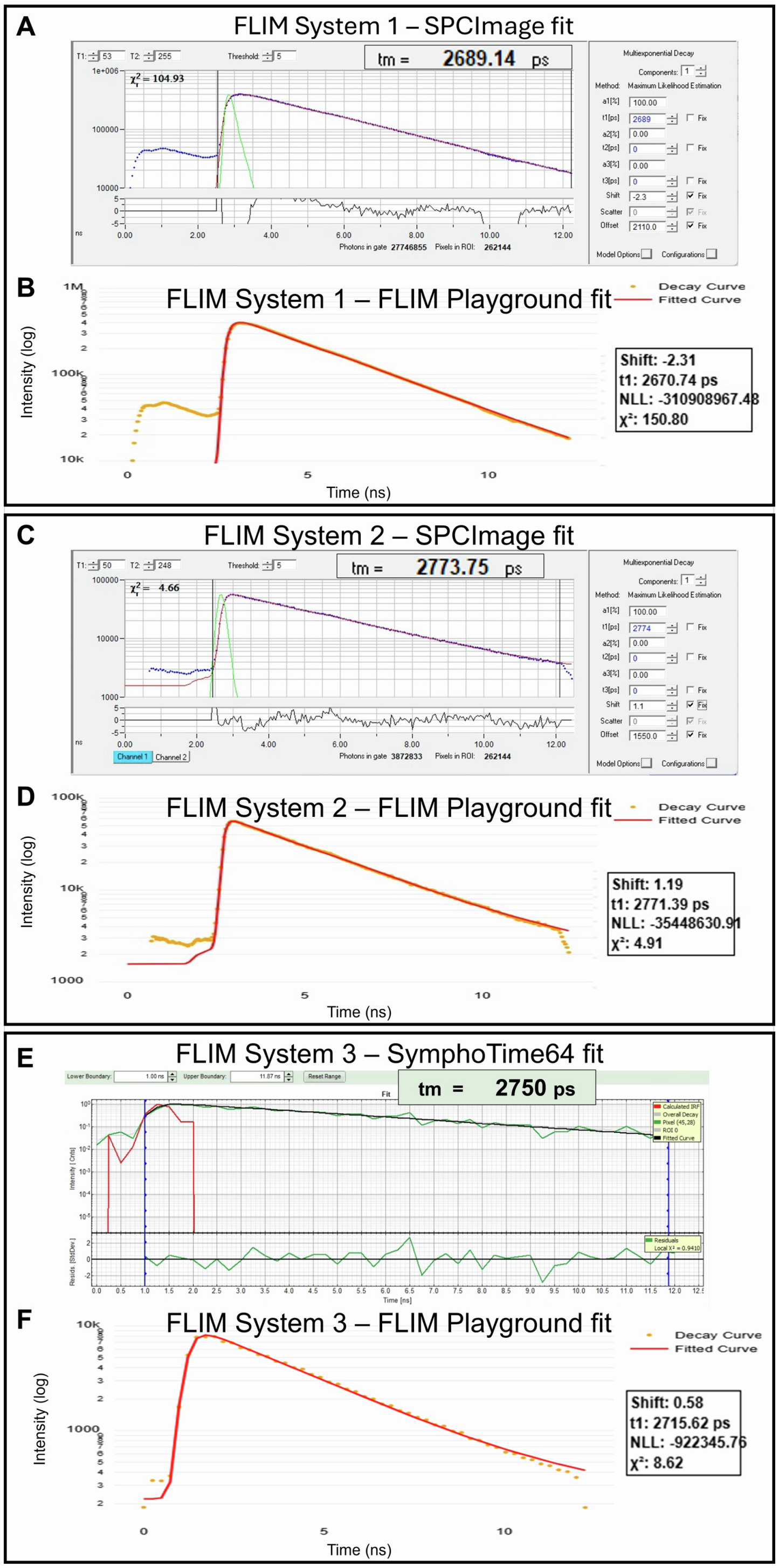

Figure S7. Cross-system multiphoton lifetime measurements of a green fluorescent slide, fit in FLIM Playground and commercial software, related to Figure 3. (A–B) The first system used Swabian Time Tagger Ultra, and the mean lifetime was fit as **(A)** 2689 picoseconds (ps) in SPCImage and **(B)** 2671 ps in FLIM Playground. **(C–D)** For system 2 using Becker & Hickl SPC-180NX, the fit mean lifetime was **(C)** 2774 ps in SPCImage and **(D)** 2771 ps in FLIM Playground. **(E–F)** For system 3 using PicoQuant TimeHarp260N, the fit mean lifetime was **(E)** 2750 ps in SymphoTime64 and **(F)** 2716 ps in FLIM Playground. The fit configurations (time gates, mono-exponential, metric) were kept the same when fit was performed in either platform.

Table S1. Extracted single-cell numerical features from PANC-1 and MCF7 cells across all treatments, computed separately for NAD(P)H and FAD channels, related to Figure 4–5. The features were grouped by selected feature extractors: Lifetime Fit, Lifetime Phasor, Morphology, and Texture. For the FAD channel, the same feature set as NAD(P)H was used, with fit and phasor features derived from FAD-specific decay files and texture descriptors from single-cell FAD intensity images. Since both channels shared the same ROI masks, morphology features were identical across channels and therefore omitted for FAD.

Acquisition Channels	Lifetime Fit	Lifetime Phasor	Morphology	Texture
NAD(P)H	α_1, τ_1, τ_2, τ_m	g, s, g_2, s_2, m, ϕ, τ_ϕ, τ_m	area, perimeter, solidity, eccentricity, major_axis_length, minor_axis_length, circularity	Granularity: 1,3,5,7,9, Radial Distribution: 1,2,3,4, Mass Displacement, Intensity Sum
FAD	α_1, τ_1, τ_2, τ_m	g, s, g_2, s_2, m, ϕ, τ_ϕ, τ_m	None	Granularity: 1,3,5,7,9, Radial Distribution: 1,2,3,4, Mass Displacement, Intensity Sum

Table S2. Summary of FLIM system configurations, including laser source, objective lens, detector, timing electronics, and analysis software, across the three systems used in this study, related to Figure 3.

System	Center	Laser	Objective Lens	Detector	Timing Electronics	Analysis Software
**1**	1	Spectra Physics Insight DS+ (750 nm)	Nikon 40x WI 1.15 NA	Hamamatsu H16722 GaAsP PMT	Swabian Time Tagger Ultra	SPCImage NG v8.9
**2**	2	Mai Tai DeepSee Ti:sapphire (750 nm)	Nikon 20x air 0.75 NA	Hamamatsu H7422P-40 GaAsP PMT	Becker & Hickl SPC-180NX	SPCImage NG v8.9
**3**	2	Mai Tai DeepSee Ti:sapphire (750 nm)	Nikon 20x air 0.75 NA	Hamamatsu H7422P-40 GaAsP PMT	PicoQuant TimeHarp260N	SymphoTime64

Methods S1: FLIM Playground Manual

(related to STAR Methods)

Table of contents

1 Quick Start 7
1.1 Installation . . . 8
1.1.1 Download . . . 8
1.1.2 Build it yourself . . . 8
1.2 Introduction . . . 8
1.3 Challenges . . . 9
1.3.1 Data Levels . . . 9
1.4 Method . . . 10
1.4.1 Feature Classes . . . 10
1.4.2 Design . . . 11
1.4.3 Summary . . . 13
1.4.4 Citation . . . 13

I Data Extraction 14

2 Overview 15
2.1 Stages . . . 15
2.2 Input File Types . . . 16
2.2.1 Decay . . . 16
2.2.2 Mask . . . 17
2.2.3 IRF . . . 17
2.2.4 SPCImage t1 . . . 17
2.2.5 Fluorescence Lifetime Standard . . . 17
2.2.6 Intensity (2D) . . . 18
2.3 Limitations . . . 18

3 Configuration 19
3.1 Cross-Channel Configurations . . . 20
3.1.1 Categorical Features . . . 20
3.1.2 Decay Types . . . 20
3.1.3 Identifiers . . . 21
3.1.4 Decay Info . . . 21
3.1.5 Calibration Method . . . 22
3.1.6 Number of Channels . . . 22

3.2 Per-Channel Configurations . . . 22
3.2.1 Channel Name . . . 22
3.2.2 Imaging Modality . . . 22
3.2.3 Feature Extractor . . . 23
3.2.4 Number of Components . . . 24
3.2.5 Fixed Lifetimes . . . 24
3.2.6 File Suffix . . . 24
3.3 Save . . . 25

4 Field of View Metadata **26**
4.1 Metadata . . . 26
4.1.1 Decay Type . . . 27
4.1.2 Channel Names . . . 27
4.1.3 Feature Extractors . . . 27
4.1.4 Decay Info . . . 27
4.1.5 File Suffix . . . 28
4.1.6 Fluorescence Lifetime Standard . . . 28
4.1.7 Folder Path . . . 28
4.2 Metadata Extraction . . . 29
4.2.1 FOV File Paths . . . 29
4.2.2 Decay Info . . . 30
4.2.3 Result Preview and Export . . . 31

5 Numerical Feature Extraction **33**
5.1 Input . . . 33
5.2 Calibration . . . 34
5.2.1 Fit Calibration . . . 34
5.2.2 Fit Free Calibration . . . 39
5.2.3 Confirm Calibration . . . 40
5.3 Feature Extraction . . . 40
5.4 Save Results . . . 41

6 Lifetime Features (Fit) **42**
6.1 Fitting . . . 42
6.1.1 Preprocessing . . . 43
6.1.2 Fraction of components . . . 43
6.1.3 Mean lifetime . . . 43
6.2 Pixel pre-fit . . . 43

7 Lifetime Features (Fit-Free) **44**
7.1 Preprocessing . . . 44
7.2 Phasor features . . . 44
7.2.1 Step 1: raw phasor coordinates . . . 44

7.2.2 Step 2: calibrated phasor coordinates 45
7.2.3 Step 3: phasor-derived lifetime 46

8 Morphological Features 47

9 Textural Features 48
9.1 Granularity 48
9.2 Radial Distribution 48
9.3 Mass Displacement 48
9.4 Intensity Sum 49

10 Categorical Feature Extraction 50
10.1 Merging Datasets 50
10.2 Assigning Categorical Features 52

II Data Analysis 53

11 Overview 54
11.1 General Workflow 54
11.2 Methods 54
11.3 Input 56
11.3.1 Requirements 56
11.3.2 Warning Messages 57
11.3.3 Error Messages 57
11.4 Shared Interactive Widgets 57
11.4.1 Numerical Selection Widgets 57
11.4.2 Filter Widgets 59
11.4.3 Visual Channels Widgets 60
11.4.4 Unique ID Hover 61
11.4.5 Plotting Configuration Widgets 62
11.4.6 Export Workflow as Python Script 62

12 Configuration 63
12.1 Analysis Profiles 63
12.2 Identifiers Config 64
12.3 Categorical Feature Config 64
12.4 Numerical Feature Config 64
12.4.1 Example 65
12.5 Save 65
12.6 Reset 65

III Univariate Analysis **66**

13 Feature Comparison **67**
13.1 Shared Interface Components . . . 67
13.2 Separate by . . . 68
13.3 Comparison Widget . . . 69
13.4 Effect Size . . . 70
13.4.1 Glass's Delta . . . 70
13.4.2 Absolute Cohen's d . . . 71
13.5 Statistical Test . . . 71
13.6 Comparison Annotation . . . 72

14 Feature Histogram **73**
14.1 Shared Interface Components . . . 74
14.2 Histogram Mode . . . 74
14.3 Gaussian Mixture Model Mode . . . 75
14.3.1 Fit Gaussian Mixture Models . . . 76
14.3.2 Heterogeneity index . . . 76
14.3.3 Classification . . . 76
14.3.4 Example . . . 77

IV Bivariate Analysis **80**

15 Feature Distribution **81**
15.1 Shared Interface Components . . . 82
15.2 Marginal Distribution . . . 82
15.3 2D Gaussian Mixture Model . . . 82
15.4 Regression line . . . 84

16 Phasor Analysis **86**
16.1 Shared Interface Components . . . 87
16.2 Select Phasor Coordinate . . . 87
16.3 K-means Clustering . . . 87
16.3.1 Export Clustered Dataset . . . 88

V Multivariate Analysis **89**

17 Dimension Reduction **90**
17.1 Shared Interface Components . . . 91
17.2 Hyperparameter Widget . . . 91
17.2.1 UMAP . . . 92
17.2.2 t-SNE . . . 92

18 Classification 94
18.1 Shared Interface Components . . . 95
18.2 Method-specific Components . . . 95
18.2.1 Classifier Hyperparameters . . . 96
18.2.2 Class Imbalance Handling . . . 100
18.2.3 Confusion Matrix . . . 106
18.2.4 Performance Metrics . . . 106
18.2.5 ROC Curve . . . 107
18.2.6 Feature Importance . . . 108

19 Common Q & A 109

1 Quick Start

Welcome to the FLIM Playground 🥳🎉🥂! This is an interactive graphical user interface (GUI) that allows you to extract single-cell features from fluorescence lifetime imaging microscopy (FLIM) raw data (Data Extraction) and analyze extracted features or datasets extracted via other methods using a built-in repertoire of methods (Data Analysis).

To quickly try out different analysis methods, download this sample dataset and try the online demo. If you prefer to use your own data, read this chapter on data analysis configuration to learn how to configure the system.

Several talks have been given about FLIM Playground:

- FLIM Playground @ SPIE 2026: 13 mins
- FLIM Playground @ the Fluorescence Microscopy Summit by Photonics Media: 31 mins

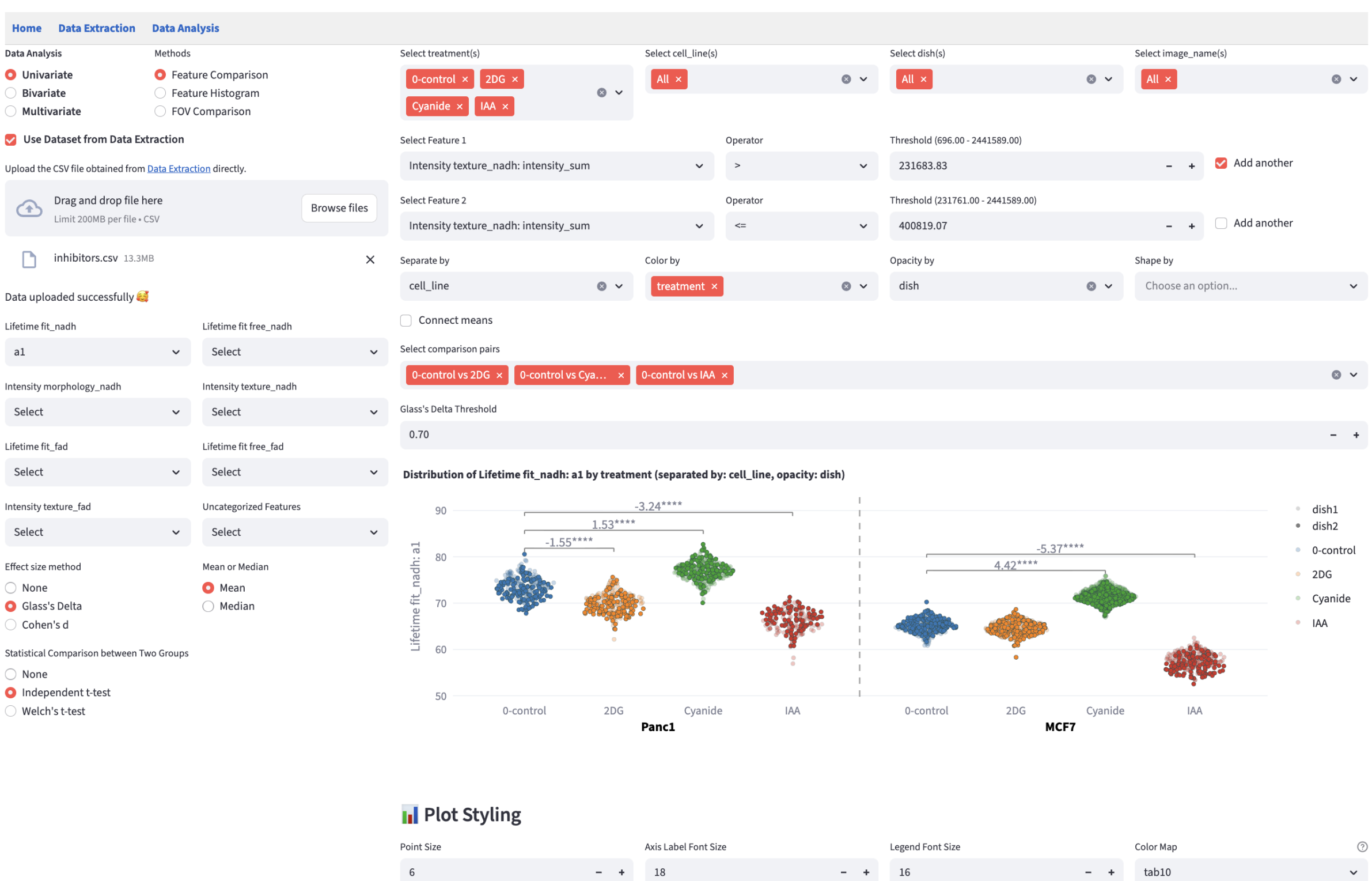

Due to the limitation in local file system access, extracting features from raw data is not available in the online demo. Read on to discover how FLIM Playground transforms raw fluorescence decay data into biological insight through an integrated, interactive workflow.

The latest version of this FLIM Playground manual is available online.

1.1 Installation

FLIM Playground is built entirely in Python and is open-source.

1.1.1 Download

Download the desktop app from GitHub and double-click it to run. Releases are currently available for Windows 11, Mac OS 26, and Ubuntu 24.04. If your operating system is not either of these, you can build it yourself by following the instructions below.

1.1.2 Build it yourself

1. Clone the repo and navigate into the repository once cloned.
2. Install the Python environment
   - Install uv if not yet installed
   - run `uv sync`
3. Build the app
   - run `pyinstaller Flim-Playground.spec --clean`

1.2 Introduction

Fluorescence lifetime imaging microscopy (FLIM) measures the time it takes for a fluorescent molecule to emit light (return to the ground state) after being excited by a pulse of light (enter the excited state). It is sensitive to changes in fluorophore microenvironment including, pH, temperature, and conformational changes due to protein-binding and the presence of quenchers[1]. Coupled with modern automated cell-segmentation methods[2], FLIM enables single-cell analyses that can reveal biological heterogeneity.

Instrumentation

To acquire FLIM data, a light source—typically a pulsed laser for time-domain methods or a modulated continuous-wave source for frequency-domain methods—is used to excite the fluorophore of interest. The emission is detected using instrumentation capable of resolving fluorescence decay, such as time-correlated single-photon counting (TCSPC), time-gated, or phase/modulation-based detection. In time-domain FLIM, the delay between excitation and photon arrival is measured, and often a histogram is built, with the x-axis representing the delay time and the y-axis representing the number of photons falling into each time bin. Compared to intensity images, FLIM has an additional dimension of time (e.g., 256 time bins per 12.5 nanoseconds).
In frequency-domain FLIM, the phase shift and modulation depth of the emission relative to the excitation are determined.

1.3 Challenges

A diverse set of tools — both open-source and commercial, ranging from libraries to code-free graphical user interfaces (GUIs) — is available to extract and analyze FLIM data, providing alternative methods and therefore flexibility to FLIM researchers. Examples include PhasorPy[3], an open-source library for analyzing fluorescence lifetime using the phasor approach; FLUTE[4], an open-source GUI for interactive phasor analysis; FLIMPA[5], an open-source phasor analysis GUI enabling batch processing, ROI-based quantification, and experiment-level comparison through manual assignment; FLIMLib, an open-source generic curve fitting library that can be used to fit fluorescence lifetime decay data; SPCImage[6], a commercial software for fitting and phasor features.

However, while some tools offer code-free interfaces, users still need to write custom code—either to prepare data in the proper format as input, or to further process their outputs for downstream analysis. The fragmentation between tools arises because each focuses on only a subset of data levels: pixel, cell ROI, channel, field of view, and experiment.

1.3.1 Data Levels

- **Pixel**:
  - A single decay curve encoded in vendor-specific file formats (e.g., Becker & Hickl, PicoQuant, etc.)
- **Region of Interest (ROI)**
  - Mask with cell labels
- **Channel**
  - Different fluorophores

  - Fluorophore-specific calibration files
  - Masks focusing on different parts of the cell (e.g., whole cell, cytoplasm, nucleus, stain, etc.)
  - Different feature extraction methods (fitting, phasor, morphology, texture)

- **Field of View (FOV)**

  - Input decay types (e.g., 2D, 3/4D, pixel-level pre-fit lifetime features)

- **Experiment**

  - Different treatments, time points, cell lines, etc., and combinations thereof

An integrated framework should take into account all data levels ■ ■ ■ ■ ■ (•) while maintaining the flexibility to handle various input types (◦). **It should provide a level of abstraction to address fragmentation from data levels and input types**.

Additionally, the use of FLIM is rapidly evolving, and new methods are being developed all the time. An integrated framework should allow users to choose among alternative methods seamlessly, be the backbone of iterative explorations integral to research, and be ready to incorporate new methods: **The provided level of abstraction should address fragmentation from extraction and analysis methods**.

Finally, many of the existing tools, including GUI-based software, are not cross-platform, which limits their accessibility. The Installation addresses this last challenge.

A closer parallel to FLIM Playground's integrated approach is the combination of CellProfiler[7], which extracts per-object morphological and texture features, and CellProfiler Analyst[8], which ingests these outputs or other feature tables for visualization and statistical analysis. However, FLIM Playground stands apart in that it can extract lifetime features from time-resolved data, alongside morphological and texture features from intensity images. Its general Data Analysis section incorporates FLIM-specific methods (e.g., phasor analysis) and provides statistical models that address additional analysis aspects such as data heterogeneity, in addition to training machine learning classifiers.

1.4 Method

1.4.1 Feature Classes

Tabular data columns can be categorized into three feature classes:

- Identifiers: unique row identifier and (optional) field of view identifier that allow us to investigate the biological heterogeneity at the single-cell level
- Categorical features: conceptually help us group the rows (e.g., `treatment` will group the rows into different treatment groups)
- Numerical features: quantify the differences/similarities between data groups

Science, from the data perspective, is about closing the conceptual categorical gaps with quantitative measurements.

At data levels, the data are processed to extract the feature classes in Data Extraction, and the classes are used in the Data Analysis section.

Increasing complexity ↑

Data Levels	Data Processing	Feature Classes
Experiment	Assign category labels (e.g., treatment, cell line, etc.) to each cell; perform data analysis on labeled cell groups.	Categorical features
Field of View	Assign FOV identifier to each cell, obtain unique cell identifiers	Identifiers
Acquisition Channel	Apply **per-channel** ROI mask, calibration and extraction methods to cell-level signals	Numerical features
Cell ROI	Aggregate pixel-level decays to obtain cell-level signals	N/A
Pixel	Read pixel-level decays encoded in different formats	N/A

1.4.2 Design

FLIM Playground has two independent sections:

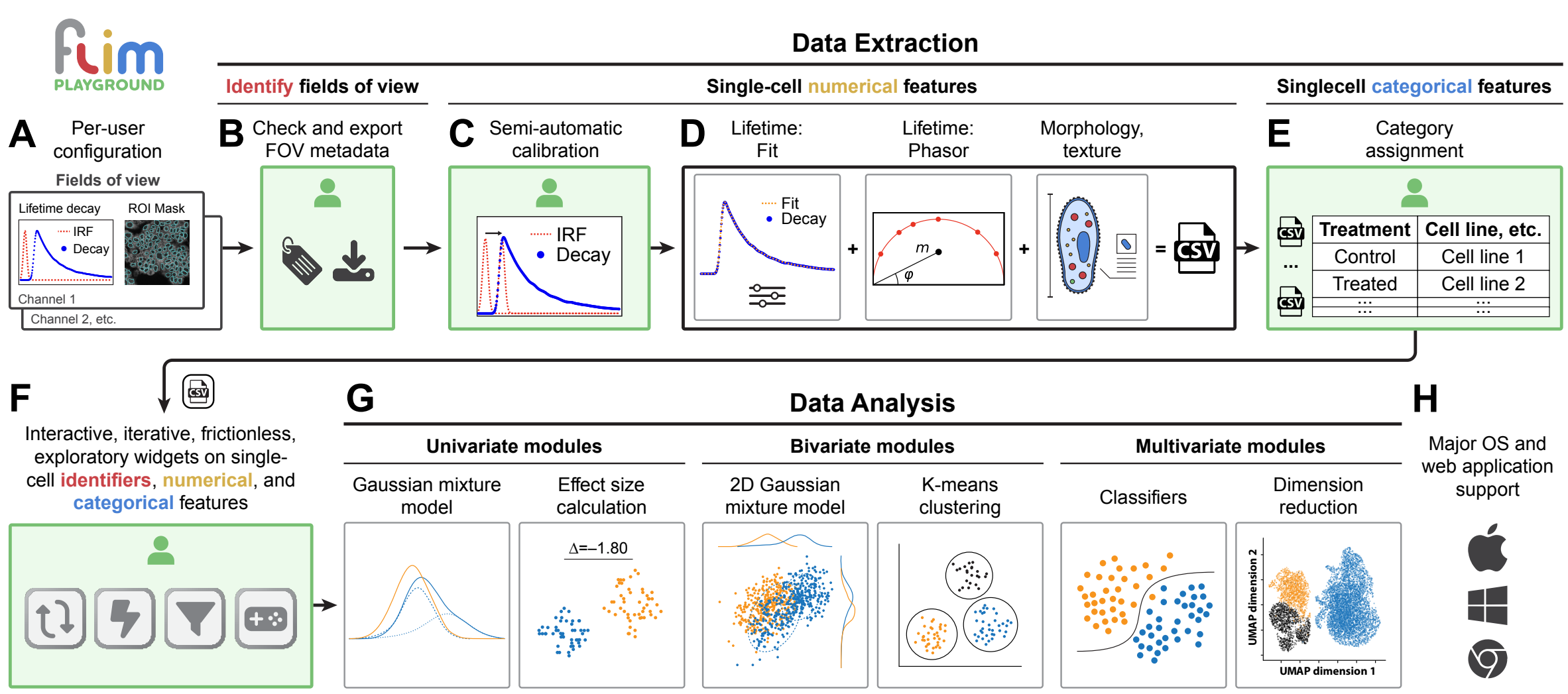

1.4.2.1 Data Extraction

Data Extraction extracts single-cell features from the raw data. It adopts a framework that offers channel-level flexibility in input types and extraction methods without incurring too much overhead for users. Following the above categorization, it is divided into the following steps:

- Data Extraction Configuration (A): allows users to choose among alternative input types and extraction methods (extractors).
- Metadata organization (B): extracts the field of view identifiers and their configurations
- Numerical Feature Extraction: extracts single-cell numerical features based on user-selected extractors. More extractors can be integrated in the future.
  - Calibration (C): calibrate for IRF shift or use fluorescence lifetime standard
  - Alternative lifetime extractors (D):
    - Fitting
    - Phasor
  - Alternative intensity-based extractors (D):
    - Morphology
    - Texture
- Categorical feature extraction (E): extracts single-cell categorical features and combines experiment-level datasets.

1.4.2.2 Data Analysis

Data Analysis analyzes features—whether extracted through Data Extraction or by other methods—using visualizations and statistical modeling. It deploys a shared framework (F) built to handle the feature classes across all analysis methods, enabling the same interactive and frictionless exploration experience and allowing new methods to be integrated in the future easily.

- Data Analysis (G) goes in-depth into how FLIM Playground handles the feature classes and Data Analysis Config goes through how users can configure FLIM Playground to analyze datasets that are not extracted by Data Extraction.

Here is the list of analysis methods incorporated into FLIM Playground, grouped by the number of numerical features they take as inputs:

- Univariate analysis
  - Feature Comparison
  - Feature Histogram
- Bivariate analysis
  - Feature Distribution

    - Phasor Analysis
- Multivariate analysis
    - Dimension Reduction
    - Classification

Both sections are built in Python and as self-contained executables ready to run on major operating systems and in browsers (**H**).

1.4.3 Summary

FLIM Playground resolves these challenges with an interactive code-free graphical user interface (GUI) that spans the full pipeline. It integrates validation checks that guide users at every step, and has a built-in repertoire of analytical methods with interactive widgets that encourage hypothesis-driven, iterative exploration of large datasets. It is built on a modular architecture that enables incorporation of new algorithms in the future.

1.4.4 Citation

FLIM Playground is currently on bioRxiv. If it contributed to your research—whether through Data Extraction for single-cell feature generation or through Data Analysis for data exploration, visualization, selection of analysis methods, or hyperparameter tuning (UMAP, clustering, classification, etc.)—please cite this work in your publication. Your citation directly supports us in maintaining and improving it ✨🎈🍾.

Part I

Data Extraction

2 Overview

FLIM Playground transforms raw FLIM data into single-cell numerical and categorical features, ensuring smooth integration with downstream analysis. All steps are seamlessly connected through interactive widgets, with built-in error checking and reporting to ensure correctness.

A high-level overview of the data extraction process is shown below.

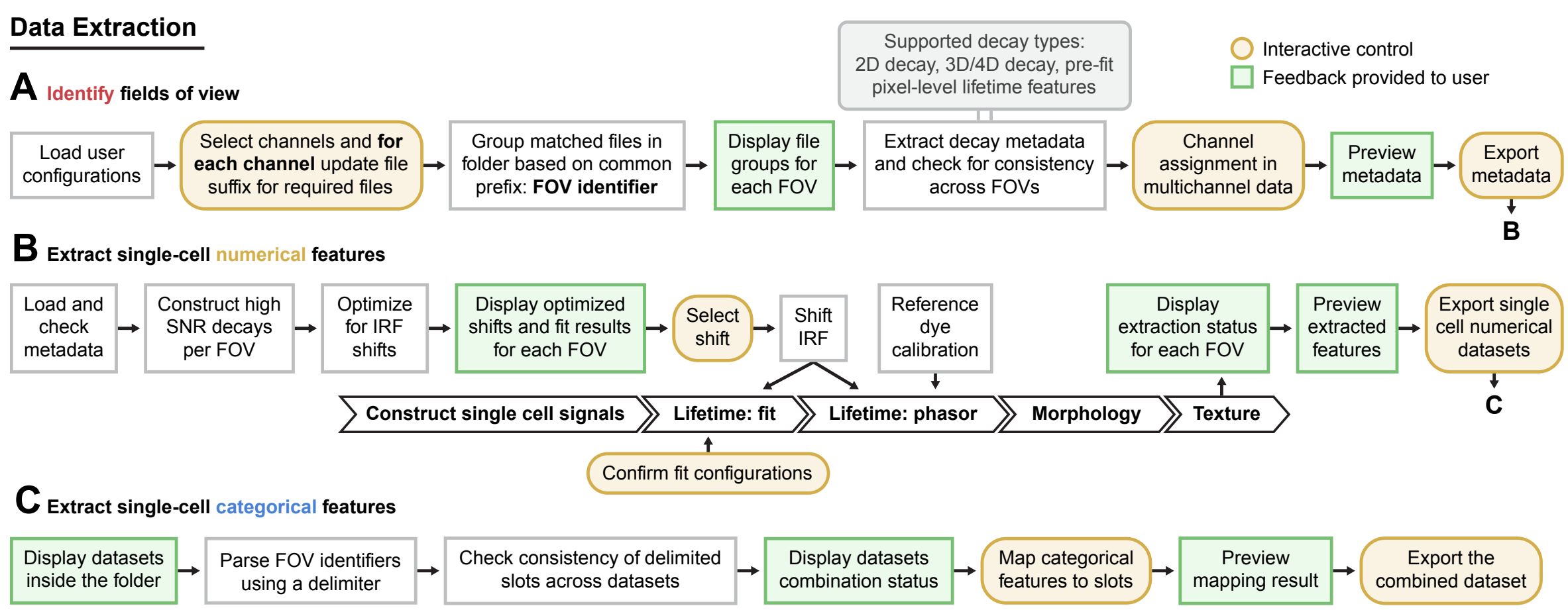

2.1 Stages

Users can configure the system once and apply it to future data. After configuration, users can extract features from raw data using the following steps, inspired by the categorization:

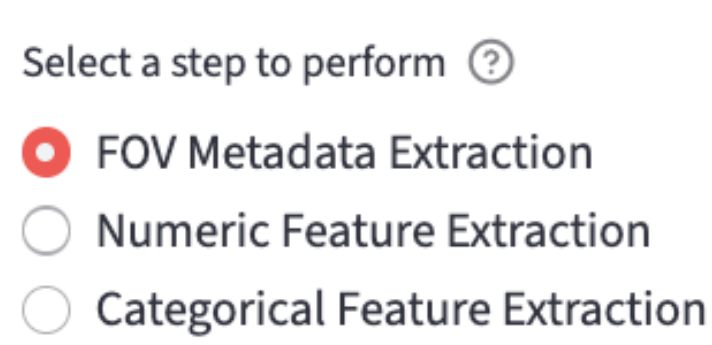

1. FOV Metadata Organization (A): each experiment session consists of multiple fields of view (FOVs). This step helps users organize the metadata of the FOVs.

2. Numerical feature extraction (B): it calibrates and then extracts single-cell numerical features.
   - Calibration
     - Fit Calibration
     - Fit Free Calibration
       * IRF Shift
       * Fluorescence lifetime standard
   - Per-channel feature extraction includes:
     - Lifetime fitting features: fit an exponential decay curve to the measured decay and extract lifetime features per cell ROI.
     - Phasor features: calculate the phasor features such as the phasor coordinates. It also provides phasor analysis.
     - Morphology features: calculate morphology features based on the ROI mask.
     - Texture features: calculate texture features based on the intensity image and the ROI mask.
3. Categorical feature extraction (C): extract the categorical features such as the treatment, time point, etc. based on the FOV name.

- Users can then upload the extracted datasets to perform Data Analysis.

2.2 Input File Types

2.2.1 Decay

See decay types for more details.

- 2D decay:
  - `.csv`: a tabular data sheet with each row representing a cell and each column representing a time bin.
- 3D/4D decay: besides the spatial dimensions and the time dimension, the additional dimension in the 4D array represents acquisition channels.
  - `.sdt`: Becker & Hickl
  - `.ptu`: PicoQuant

2.2.2 Mask

Because of the single-cell focus of FLIM Playground, cell-level masks are needed for each channel. Channels can share the same mask, or use different masks (when they use different masks, the mask ID for each cell should match across masks).

The cell-level mask can focus on different parts of the cell region, such as the whole cell, the cytoplasm, the nucleus, or the stain part.

For an example, see here.

- `.tiff`/`.tif`: a 2D array with background labeled as `0` and each cell ROI region labeled as a unique positive integer. That integer will be part of the unique cell identifier.

2.2.3 IRF

IRF must be a 1D array. Currently, the supported formats are (extendable to more formats in the future):

- `.txt`: it uses `np.loadtxt` to parse a txt file. It returns a 1D array if the IRF numbers are in one row or in one column.

2.2.4 SPCImage t1

If the decay type is 3D/4D pixel pre-fit, users can provide pixel pre-fit lifetime feature files.

- `.asc`: a 2D array in spatial dimensions outputted by SPCImage, with each (row, column) containing the value of the lifetime feature (e.g. `t1`: the first component's lifetime) of that pixel.
- Depending on the chosen number of lifetime components, the other pixel pre-fit feature files are included and their names are inferred from the `t1` file, by replacing `t1` in the file name with `t2`, `t3`, `a1[%]`, etc.

2.2.5 Fluorescence Lifetime Standard

If the fluorescence lifetime standard-based calibration is chosen, users are expected to provide the fluorescence lifetime standard file.

- `.tiff`/`.tif`: a 3D array, with one dimension representing the time axis.

2.2.6 Intensity (2D)

If the channel's imaging modality is `Intensity-only`, users are expected to provide a 2D intensity image in:

- `.tiff`/`.tif`: a 2D array.

2.3 Limitations

- To simplify the workflow and due to the framework chosen, FLIM Playground does not support pixel-level fitting and fit-free analysis, and there are many other open-source tools that can do so (for a comprehensive list, see here). Instead, it does cell-level fitting and fit-free (phasor) feature extraction by summing up all the decays belonging to the same cell ROI to get one cell-level decay as a preprocessing step[9]. It also accepts pre-fit pixel-level lifetime features from SPCImage and aggregates pixel-level lifetime features to cell-level lifetime features.

3 Configuration

> **Note**
>
> Users can configure the system once, and it will be applied to future data.

Constructing a framework that balances flexibility and usability is about choosing the right abstraction level. FLIM Playground chooses acquisition *channel* from data levels as the focus and adopts the **channel-centric framework**. Settings are divided into two categories: cross-channel configurations and per-channel configurations.

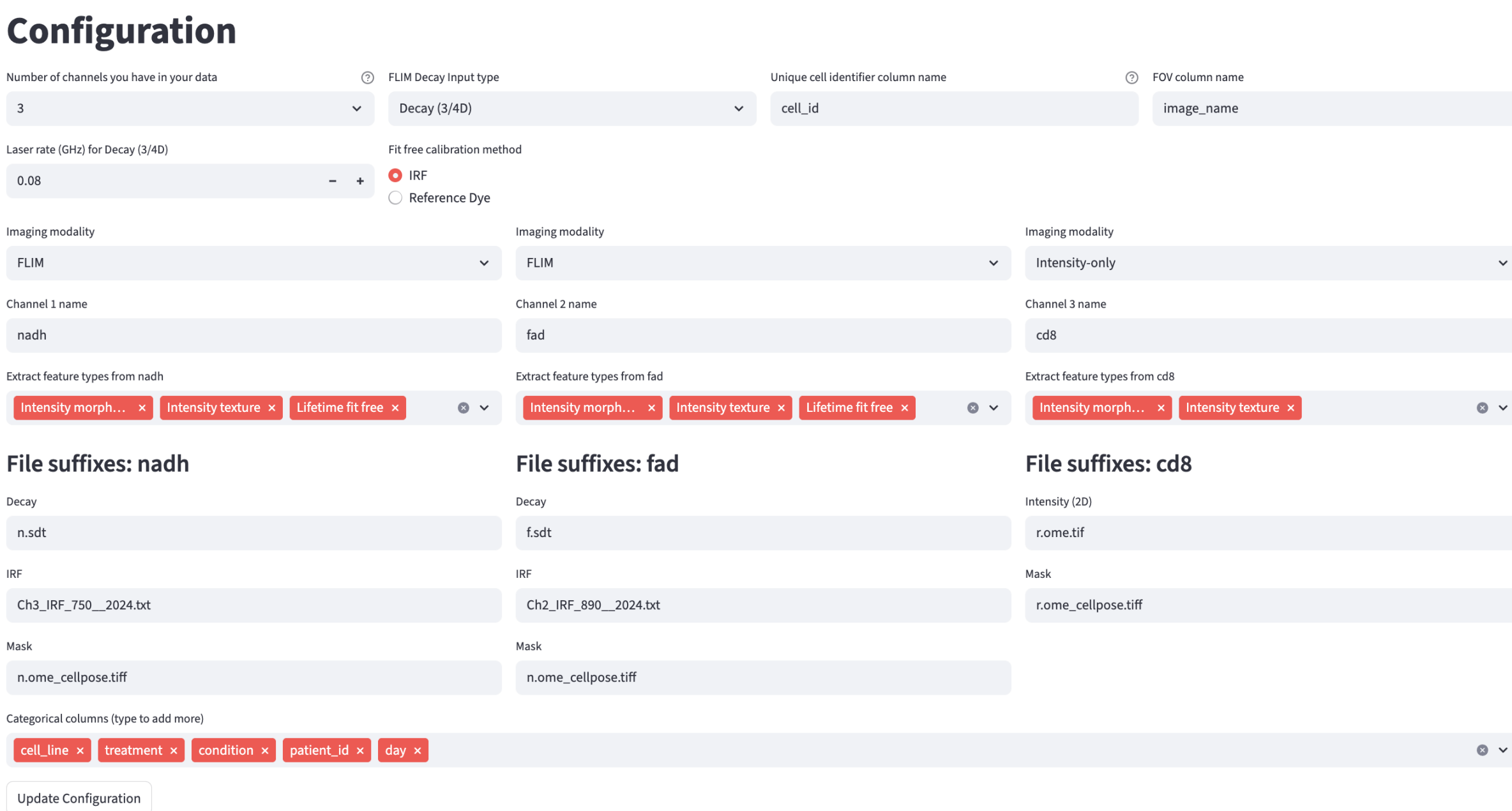

3.1 Cross-Channel Configurations

3.1.1 Categorical Features

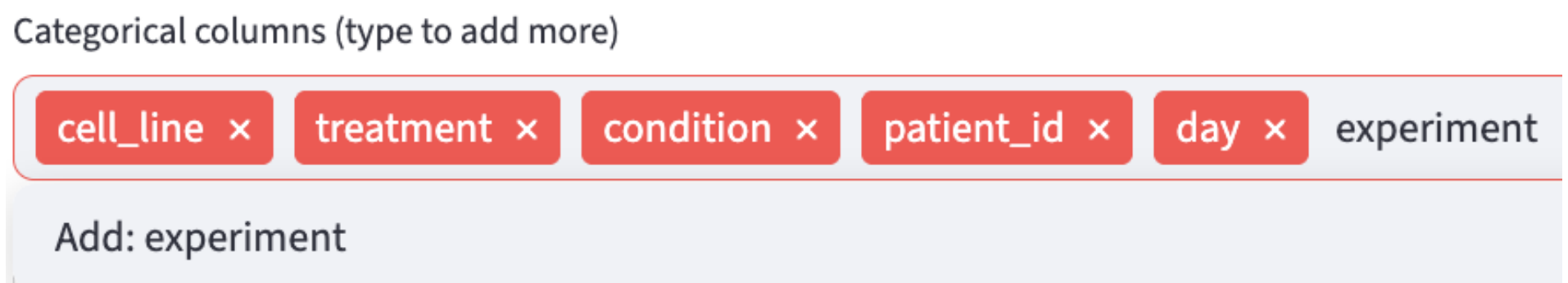

Categorical features are useful to organize the data into groups of interest, and will be used extensively in the Data Analysis section. Users can specify the potential categorical feature names to be extracted in the Categorical Feature Extraction step.

3.1.2 Decay Types

FLIM Playground supports different decay types, including 2D decay, 3D/4D decay, and 3D/4D pixel pre-fit decay, through user specification.

3.1.2.1 2D Decay

In the system developed by Samimi et al.[10], single cells flow through and a decay curve is acquired for each cell by aggregating all the photon arrival time delays deemed to be from the same cell. In exchange for spatial distribution, the acquisition speed is increased enormously. The output of the system is a tabular data sheet with each row representing a cell and each column representing a time bin. **FLIM Playground currently supports 2D decay in the CSV format.**

3.1.2.2 3D/4D Decay

In TCSPC-based FLIM, the data is stored in a 3D/4D array and in special format (e.g. `.sdt`, `.ptu`). In addition to the spatial dimensions and the time dimension (`XYT`), the channel dimension may also be present, making it 4-dimensional (`CXYT`). **FLIM Playground currently supports both `.sdt` and `.ptu` files up to those 4 dimensions.** It reads the `duration` and the number of `time bins` from the decay file metadata. Some systems can also record a time stack, which is not supported yet.

3.1.2.3 3D/4D pixel pre-fit

Since FLIM Playground currently does **not** support pixel-fitting, users have the option to provide pixel pre-fit outputs from SPCImage in the format of `.asc`. See here for how FLIM Playground processes SPCImage outputs to obtain cell-level lifetime fitting features.

3.1.3 Identifiers

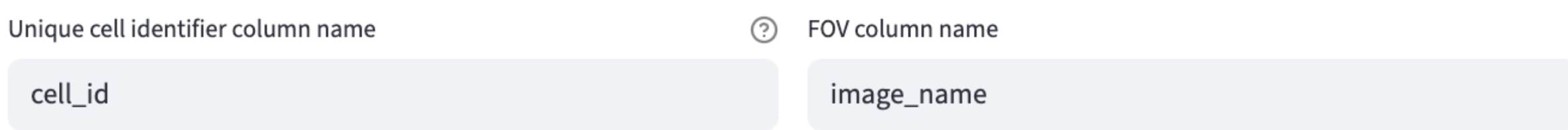

3.1.3.1 Cell Identifier

Users can specify the column name for the unique identifier of the cell to be stored in the output dataset. The cell identifier is constructed as `{FOV Identifier}_{cell_label}`, where the `cell_label` is the unique integer label in the mask or the row number if the decay type is 2D decay.

3.1.3.2 FOV Identifier

Users can specify the column name for the field of view identifier. If the decay type is 2D decay, users may want to specify the column name as `exp_name`, because the FOV is not an image.

3.1.4 Decay Info

3.1.4.1 Laser frequency

Users can specify the laser frequency in GHz. For example, 0.08 GHz equals 80 MHz. It will be used in the phasor analysis. If you do not intend to use the phasor analysis, you can fill in any number.

3.1.4.2 2D Decay-specific

Since the 2D decay file does not provide the `duration` and the number of `time bins` per laser pulse interval, users need to specify them.

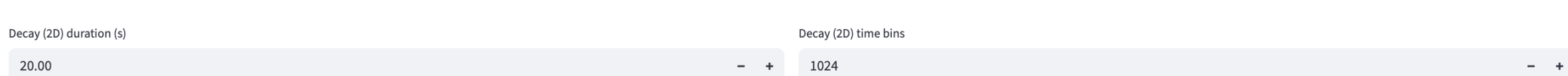

3.1.5 Calibration Method

It supports two calibration methods for phasor analysis:

- IRF shift-based calibration
- fluorescence lifetime standard-based calibration

Since the instrumental response function (IRF) or the fluorescence lifetime standard is measured for each channel, they are set in the per-channel configurations. The fluorescence lifetime standard's lifetime is channel-agnostic.

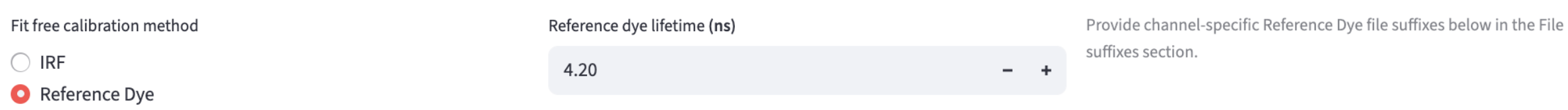

3.1.6 Number of Channels

Each FOV is assumed to have the same number of channels. The configuration will dynamically adjust the per-channel configurations based on the specified number of channels. Currently, the maximum number of channels is 4, and it can be extended to house more channels.

3.2 Per-Channel Configurations

3.2.1 Channel Name

Users can customize the channel name based on, for example, the name of the fluorophore.

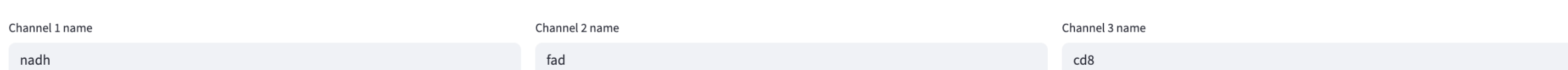

3.2.2 Imaging Modality

Users can specify the imaging modality for each channel. Currently, two modalities are supported:

- `FLIM`: The signal is time-resolved, i.e., has a time dimension, and is expected to be from one of the decay types.
- `Intensity-only`: The signal is a spatial map of intensity values (2 spatial dimensions), i.e., has no time dimension. **Currently, the only supported signal type is a 2D intensity image in the format of `.tiff`/`.tif`.**

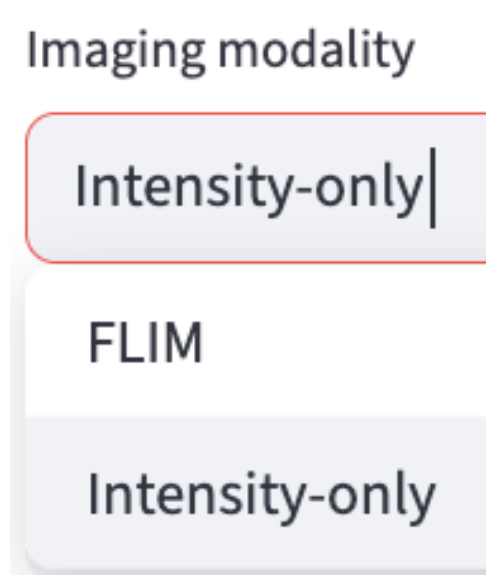

Tip

This setup allows users to image some channels in FLIM mode, and others in non-FLIM mode. It also allows users to extract and analyze their data if they image in non-FLIM microscopic systems such as epifluorescence, confocal, brightfield, etc. by selecting `Intensity-only` for all channels.

Note

If the decay type is 2D decay, the imaging modality for all channels is restricted to `FLIM`.

3.2.3 Feature Extractor

Four types of feature extractors are available if the imaging modality is `FLIM`:

- Lifetime fit
- Lifetime fit free
- Intensity Morphology
- Intensity Texture

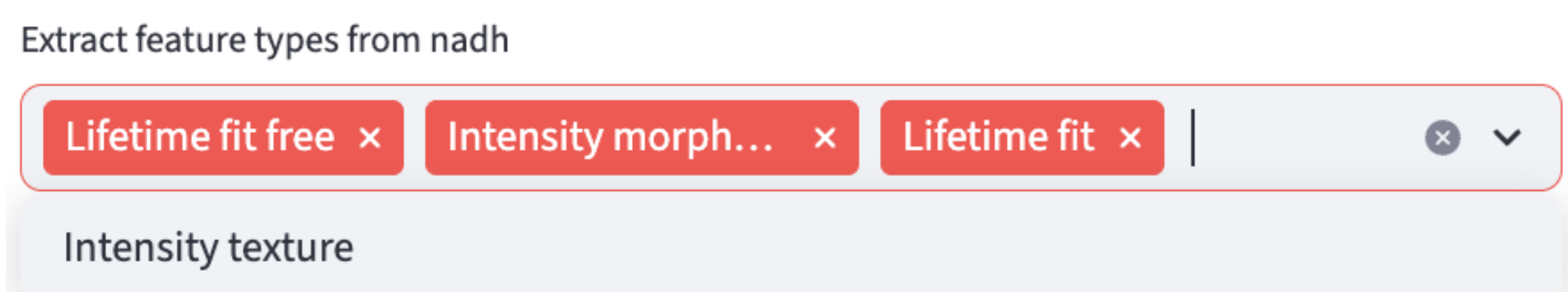

Note

If the decay type is 2D decay, the Intensity Morphology and Intensity Texture feature extractors are **not** applicable.

The Intensity Morphology and Intensity Texture feature extractors are available if the imaging modality is `Intensity-only`.

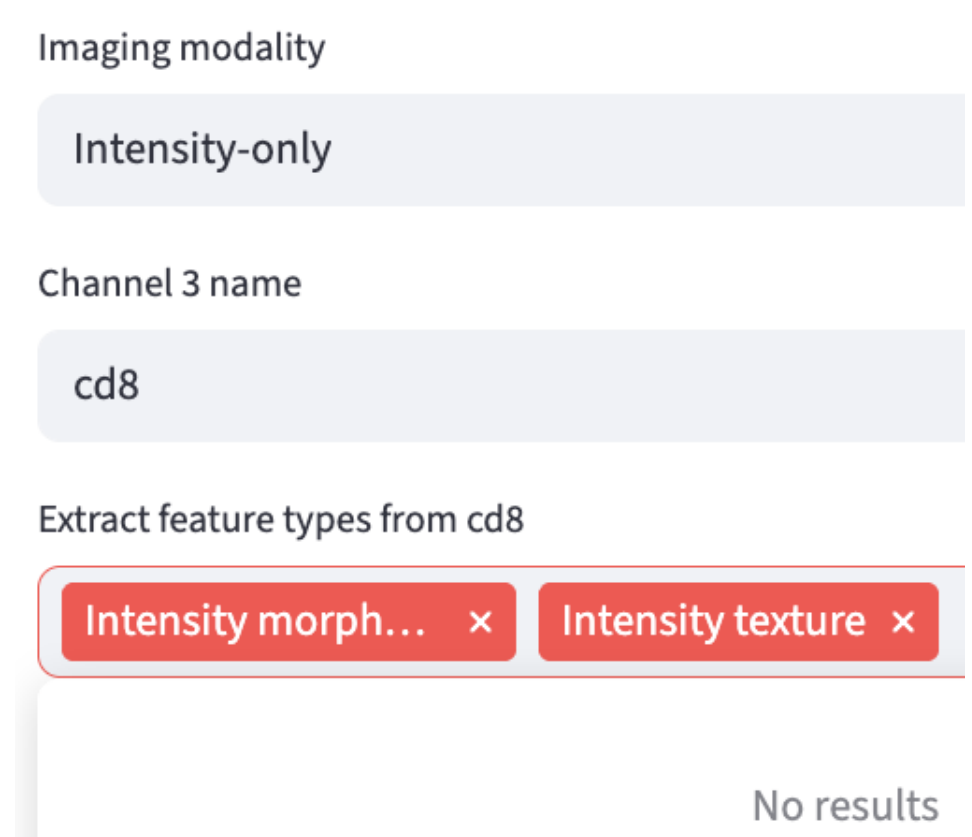

Each channel can be assigned its own set of feature extractors. For example, if users do not intend to do lifetime extraction, they can de-select the `Lifetime fit` and `Lifetime fit free` options.

3.2.4 Number of Components

If users select the `Lifetime fit` under this channel, they can specify the number of components to fit.

3.2.5 Fixed Lifetimes

Users can optionally assign a fixed value (in ns) for specific lifetime components (when $n > 1$, e.g., t_1, t_2). If a value is provided, it serves as the default fixed lifetime during the interactive fitting phase. If left blank (or set to 0), the lifetime acts as a free parameter to be optimized. This is particularly useful when analyzing fluorophores with prior knowledge of their lifetimes.

3.2.6 File Suffix

The FOV Metadata Organization step looks for associated input files for all channels of a given FOV using file suffixes. Based on the decay type, calibration method, and the selected feature extractors, the system automatically generates the file suffixes for required input files.

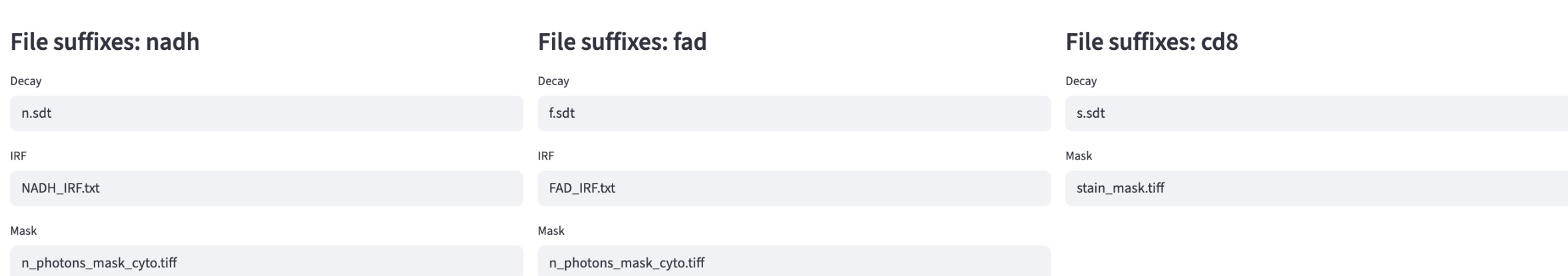

In the example above, channels `nadh` and `fad` were asked to provide an IRF file, while channel `cd8` did not, because `Lifetime fit` was selected for the former two channels and not for the latter. The file suffixes of the IRF files were different for each of the two channels, but they shared the same ROI mask file suffix because they used the same ROI mask. The `cd8` channel used a different ROI mask than the other two channels, indicated by the mask file suffix.

For detailed information on what file formats are supported for each file type (Mask, IRF, etc.), see input file types.

For detailed information on how the file suffixes are used, and what each file type (e.g. `IRF`, `Mask`, etc.) is, see the FOV Metadata Organization page.

3.3 Save

Finally, users can save the configuration for future use by clicking the `Update Configuration` button.

4 Field of View Metadata

It is the first step in the Data Extraction workflow. It organizes the metadata of each field of view (FOV)—including file paths for all required inputs and decay information—into a single metadata file. This file is then used for numerical feature extraction, as well as for users' own bookkeeping and troubleshooting.

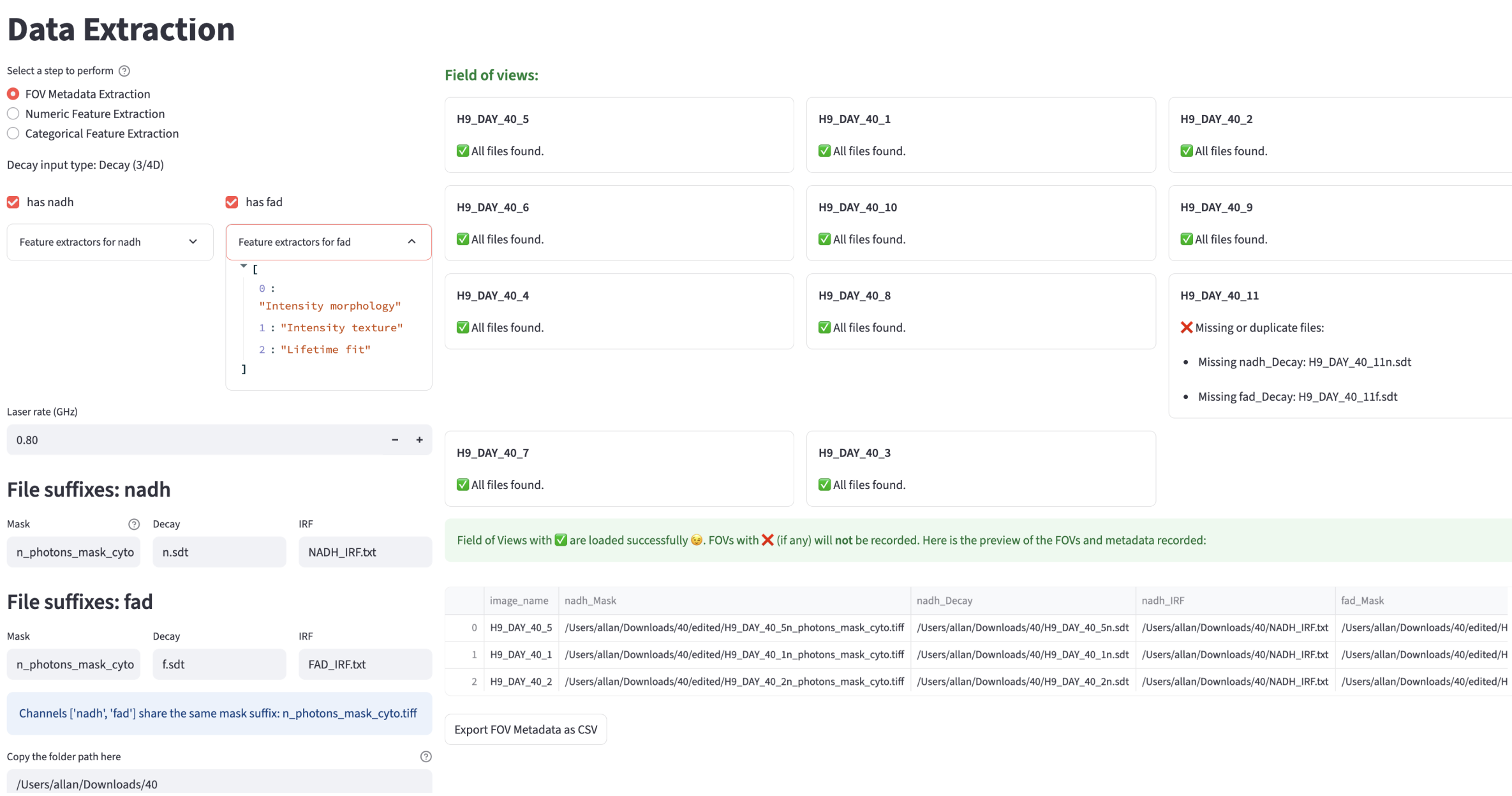

It is divided into two sections:

- The left 1/3 of the screen is the metadata configuration panel, inherited from the Data Extraction Configuration step.
- The right 2/3 shows the extraction status for all FOVs.

4.1 Metadata

Metadata are configured earlier in the Data Extraction Configuration step and copied here. Some fields remain editable for flexibility, while others are fixed to ensure usability. Non-editable fields can be

modified in the Data Extraction Configuration step. After saving your modifications, return to this page and refresh it to see the changes.

4.1.1 Decay Type

See decay types for more details. The decay type is not editable here.

Decay input type: Decay (3/4D)

4.1.2 Channel Names

See channel name config for more details. The names are not editable here, though users can choose which channel to include.

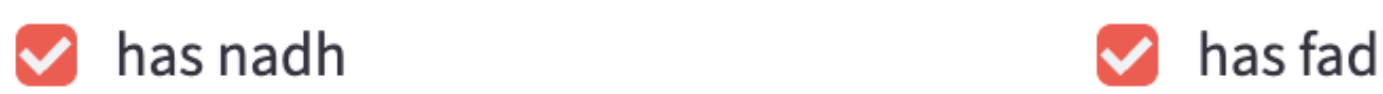

4.1.3 Feature Extractors

See feature extractor config for more details. The feature extractors for each channel are shown but not editable here.

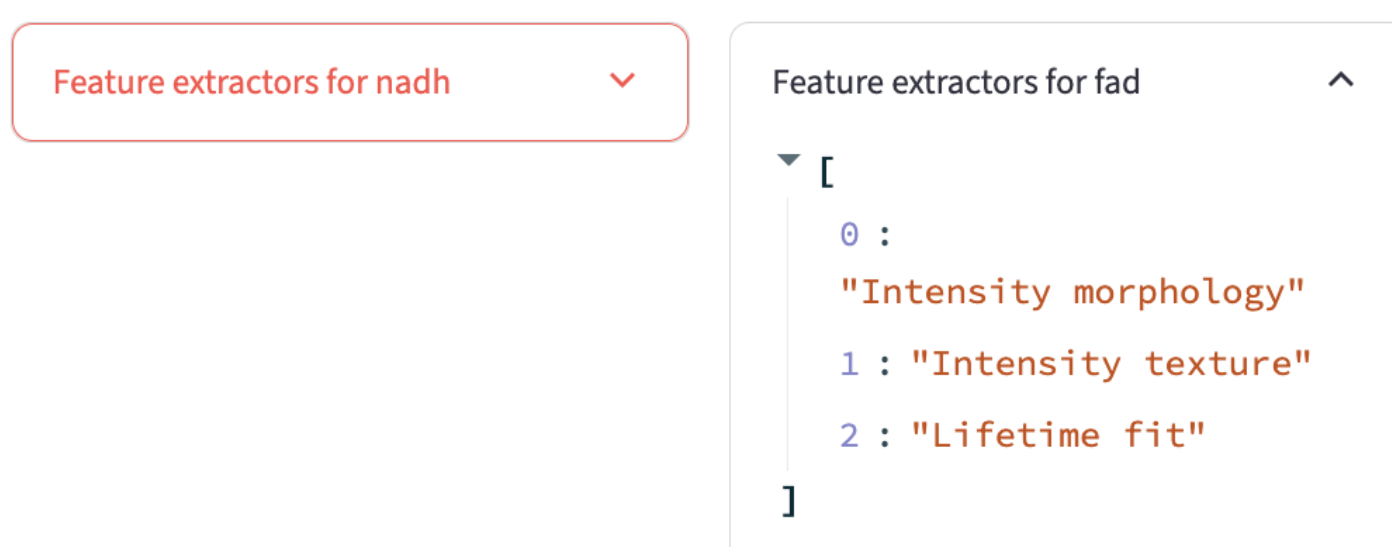

4.1.4 Decay Info

See decay info config for more details. The decay information is editable here.

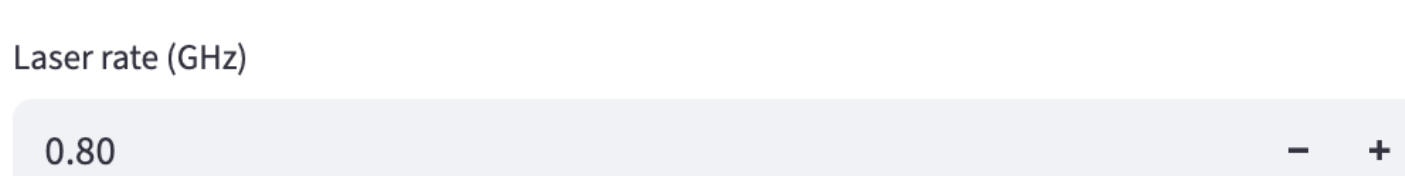

4.1.4.1 2D Decay-specific

Since the 2D decay file does not provide the `duration` and the number of `time bins` per laser pulse interval, users need to specify them.

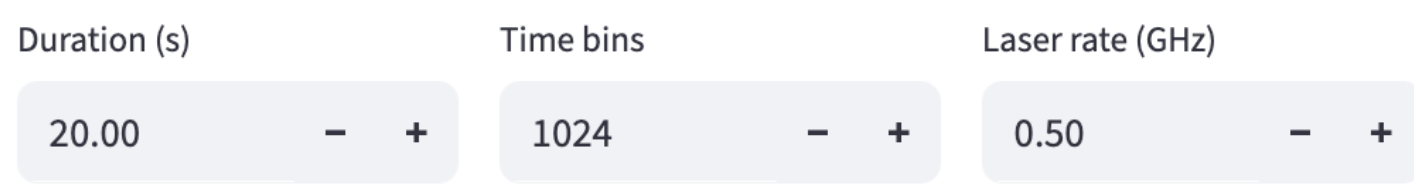

4.1.5 File Suffix

Copied from the file suffix config. Users can edit the file suffixes here.

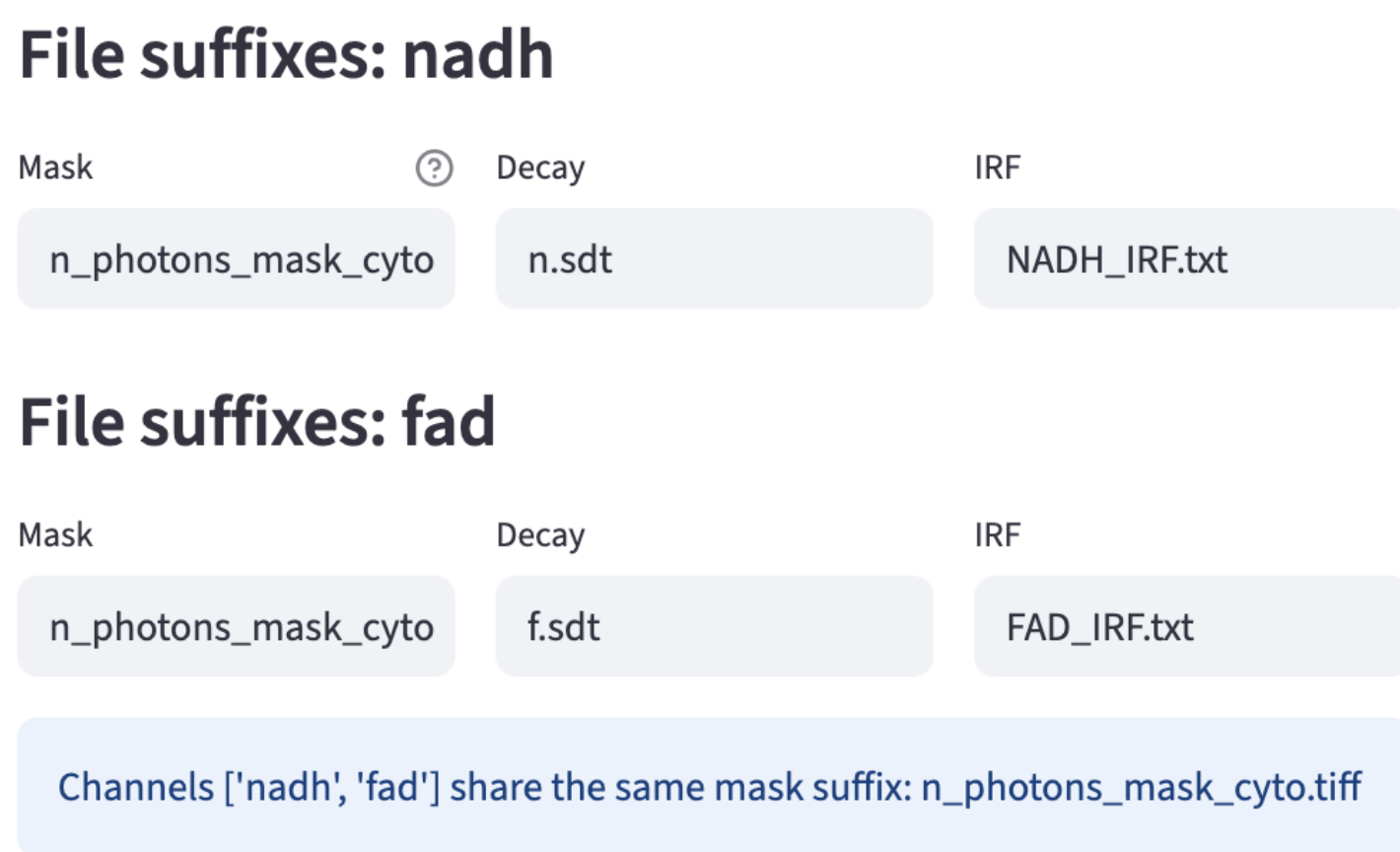

4.1.6 Fluorescence Lifetime Standard

If the selected fit free calibration method is fluorescence lifetime standard, users are asked to specify the fluorescence lifetime standard file path for each channel and the fluorescence lifetime standard's lifetime, which are copied from the data extraction configuration.

4.1.7 Folder Path

Finally, users can specify the folder path that contains all the required input files.

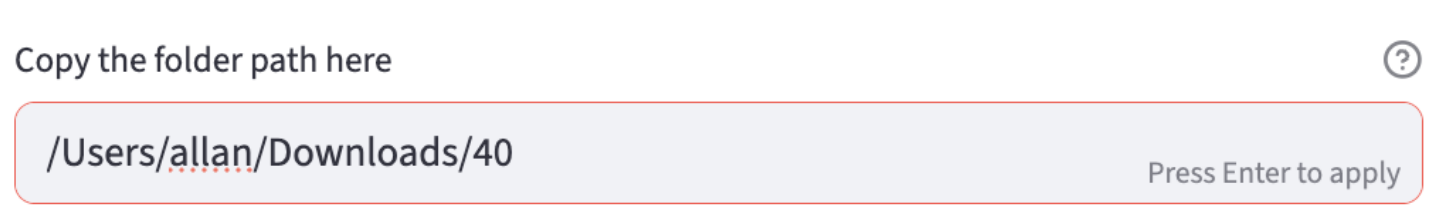

4.2 Metadata Extraction

4.2.1 FOV File Paths

The first step is to find the fields of view:

- It finds all the files recursively in the folder path that ends with the 1st file suffix of the 1st channel.
- The prefix of all the matched files is considered to be the field of view name.
    - FOV name = file name - 1st file suffix

Then, it uses the found FOV names to find all other files:

- The file name to search = FOV name + other file suffixes for each file suffix

The only exceptions are the calibration files, because they are not FOV-specific. They will be searched for based on their file suffixes only.

4.2.1.1 Success

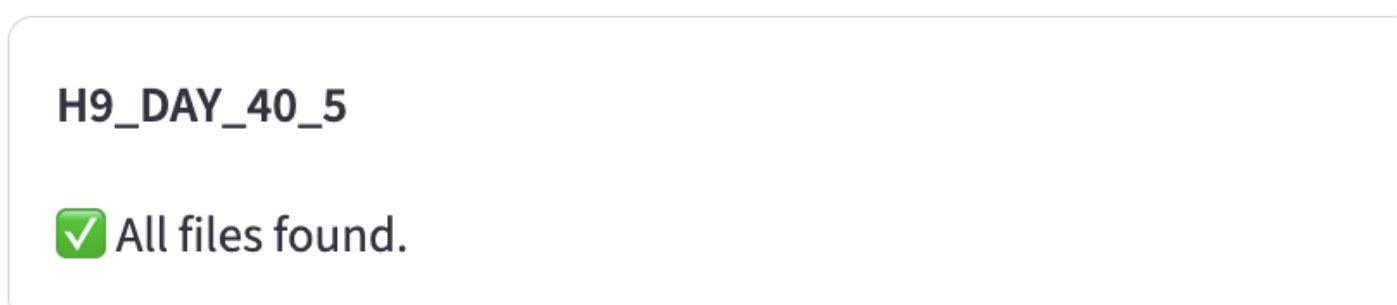

4.2.1.2 Missing

When it cannot find the file based on the file name (FOV name + file suffix for that file type) inside the folder path, it will complain.

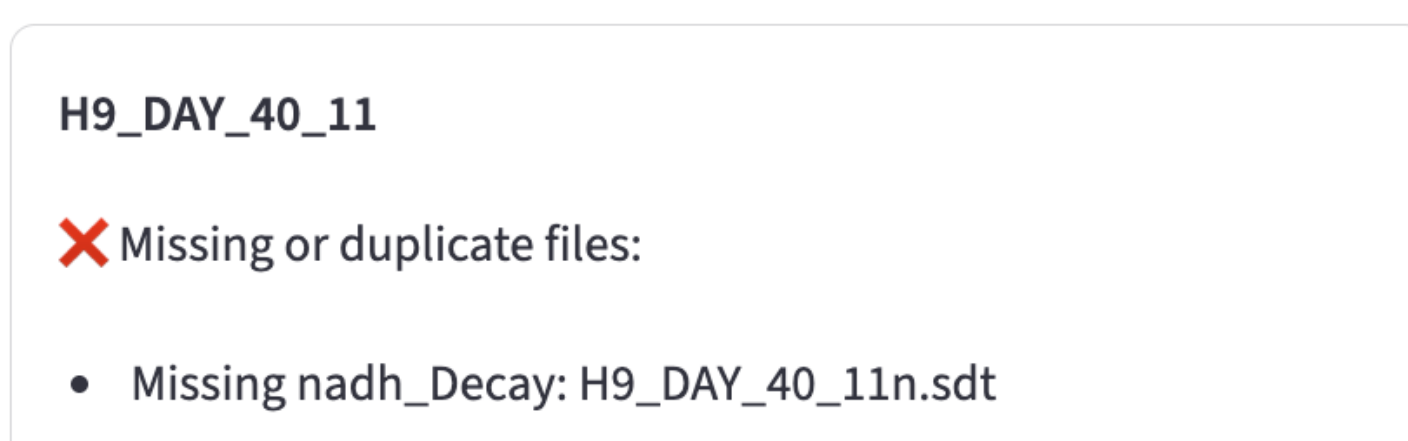

4.2.1.3 Duplicate

Because the calibration file is not FOV-specific, it will be searched based on the file suffixes only. If there are multiple files that match the same file suffix, FLIM Playground will be confused about which one to use.

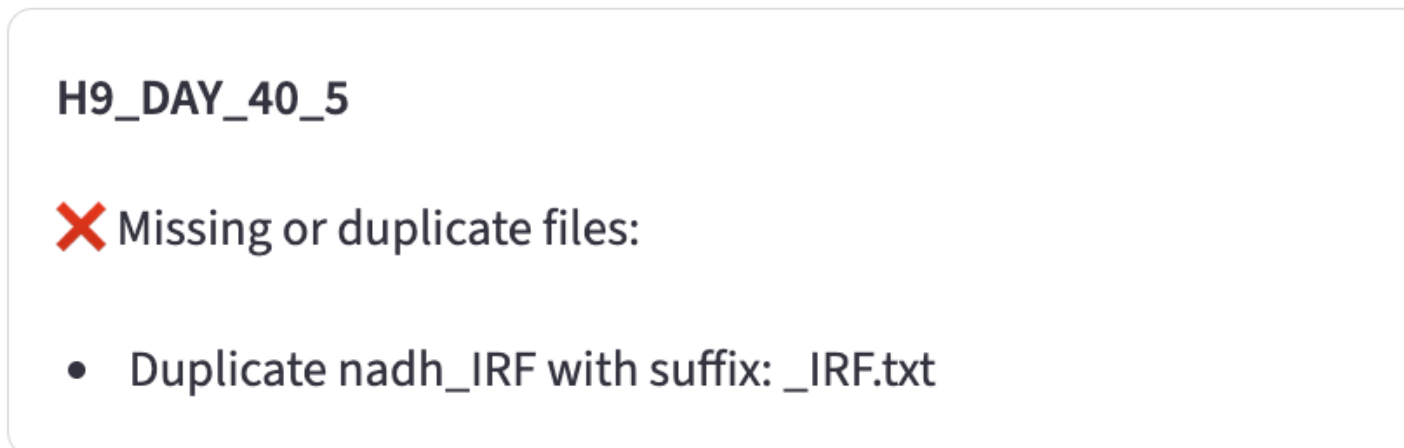

Fields of view with ✅ are loaded successfully 🎇. FOVs with ❌ (if any) will not be recorded.

> 💡 Tip
>
> If the issue persists after renaming the files to match the required prefix, hit the `Rescan Folder` button to force a re-scan of the folder. 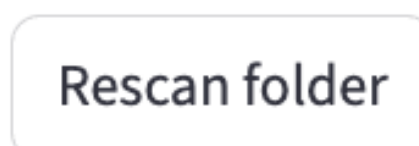
>

4.2.2 Decay Info

If the decay type is 3D/4D decay, FLIM Playground will try to infer the `duration` and the number of `time bins` per laser pulse interval from the decay file metadata. It will also check for inconsistencies across all decay files.

4.2.2.1 Channel Assignment

If the decay file associated with the channel found by the previous step is a 4D array, then FLIM Playground will try to infer the channel number for that channel. For each channel name, it will list all non-empty channels as potential channels to be assigned. If there is only one, the assignment is automatic. If there are multiple, users need to select the channel intended. It will also check for inconsistencies across all decay files about their dimensions and complain if there are any.

4.2.2.2 Fluorescence Lifetime Standard

If the file path to the fluorescence lifetime standard is specified, FLIM Playground will try to look for and read the file. It expects a `tiff` or `tif` file that contains a 3D array, with one of them as the time dimension. It will try to match the time dimension with the time dimension of the decay file. If it cannot find the matching time dimension, it will complain.

> Error: Reference dye file 'test.tif' not found in folder. Please check the file name.

> Error: Cannot find the time axis (256 time bins) in the reference dye file dimensions: (56, 512, 512)

Once the time axis is matched, the file path to the fluorescence lifetime standard, the fluorescence lifetime standard's lifetime, and the time axis will be recorded.

4.2.3 Result Preview and Export

If there is a non-zero number of FOVs with ✅ and channel assignment and fluorescence lifetime standard checks (if applicable) are passed, a preview of the metadata data sheet will be shown for users to check.

	image_name	nadh_Mask	nadh_Decay	nadh_IRF	fad_Mask
0	H9_DAY_40_5	/Users/allan/Downloads/40/edited/H9_DAY_40_5n_photons_mask_cyto.tiff	/Users/allan/Downloads/40/H9_DAY_40_5n.sdt	/Users/allan/Downloads/40/NADH_IRF.txt	/Users/allan/Downloads/40/edited/H
1	H9_DAY_40_1	/Users/allan/Downloads/40/edited/H9_DAY_40_1n_photons_mask_cyto.tiff	/Users/allan/Downloads/40/H9_DAY_40_1n.sdt	/Users/allan/Downloads/40/NADH_IRF.txt	/Users/allan/Downloads/40/edited/H
2	H9_DAY_40_2	/Users/allan/Downloads/40/edited/H9_DAY_40_2n_photons_mask_cyto.tiff	/Users/allan/Downloads/40/H9_DAY_40_2n.sdt	/Users/allan/Downloads/40/NADH_IRF.txt	/Users/allan/Downloads/40/edited/H

Export FOV Metadata as CSV

As an example, the following metadata are extracted:

- `image_name`: The `image_name` column is the name of each FOV, which is specified in the fov identifier config.
- `nadh_Mask`, `nadh_Decay`, `nadh_IRF`, `fad_Mask`, `fad_Decay`, `fad_IRF`: the file paths of the mask, decay, and IRF files for the `nadh` and `fad` channels.
- `nadh_input_type`, `nadh_imaging_modality`, `fad_input_type`, `fad_imaging_modality`: the decay type for each channel. If they share the same imaging modality, the input types should be the same. The only imaging modality supported by FLIM Playground is `FLIM`, therefore the input_type for all channels should be from one of the decay types.
- `nadh_Lifetime fit free`, `nadh_Intensity morphology`, `nadh_Lifetime fit`, `fad_Intensity morphology`, `fad_Intensity texture`, `fad_Lifetime fit`: feature extractors for each channel.

- `nadh_channel`, `duration`, `fad_channel`, `time_bins`, `laser_rate`: the decay info for each channel and info shared by all channels.

The user can click the `Export FOV Metadata as CSV` button to export the metadata as a CSV file.

5 Numerical Feature Extraction

In this step, FLIM Playground extracts *single-cell* numerical features from the raw data in a folder. For each channel, the features to be extracted are specified in the user-selected feature extractors. The available feature extractors are:

- Lifetime fit: fit exponential models to decay curves to estimate lifetimes and their fractional contributions by minimizing an objective function.
- Lifetime fit free: transform decay curves to phasor space using Fourier transform to obtain phasor coordinates.
- Intensity Morphology: morphological features of single-cell ROI masks
- Intensity Texture: texture features of single-cell ROI intensity images

5.1 Input

A CSV file extracted in the fov metadata extraction step that contains the metadata of the FOVs including:

- required file paths
- decay type
- decay info
- selected feature extractors for each channel
- fit free calibration method (if fit free calibration is required)

Users can either upload a previously extracted metadata file (left), or use the cached metadata file just extracted in the fov metadata extraction step (right).

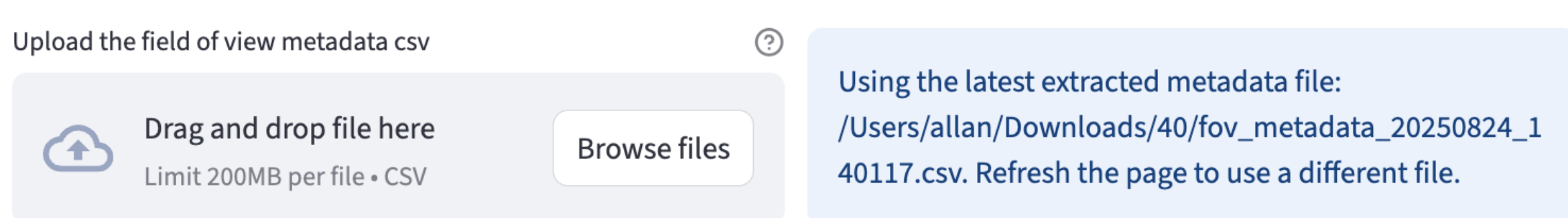

For `Lifetime fit` and `Lifetime fit free` feature extractors, the first step is always calibration.

5.2 Calibration

Calibration in FLIM is essential because raw decays are convolved with the instrument response function (IRF)—the timing profile of the detection system's response to an ultrashort light pulse that broadens and shifts the measured decay—so without correcting for these, fitted lifetimes and phasor positions are biased and not comparable across days, samples, or instruments.

5.2.1 Fit Calibration

During different experiments, the IRF may shift differently with respect to the measured decay. Before applying reconvolution fitting, the IRF shift needs to be estimated for each channel if the `Lifetime fit` feature extractor is selected for the channel.

It is performed in two steps:

1. Gather the high signal-to-noise ratio (SNR) decay curves
2. For each curve, perform reconvolution fitting and set the shift value as the free parameter to be optimized/fitted. The shift search range is centered on the initial shift guess and extends $\pm 2 \times$ FWHM of the IRF in both directions, where FWHM is the full width at half maximum of the IRF measured in time bins. This focused search window prevents the optimizer from getting stuck in suboptimal basins when time gates are restrictive.

The distribution of the shift values is displayed as an interactive scatter plot. When clicking on a point, the corresponding decay curve with the fitted curve and the key statistics are displayed for diagnostics. Based on the distribution or using certain prior knowledge, users can specify the shift value applied to all fields of view (if `Fix the Shift` is selected, see fitting options), or use the fov-specific shift value found by the fitting (if `Fix the Shift` is deselected).

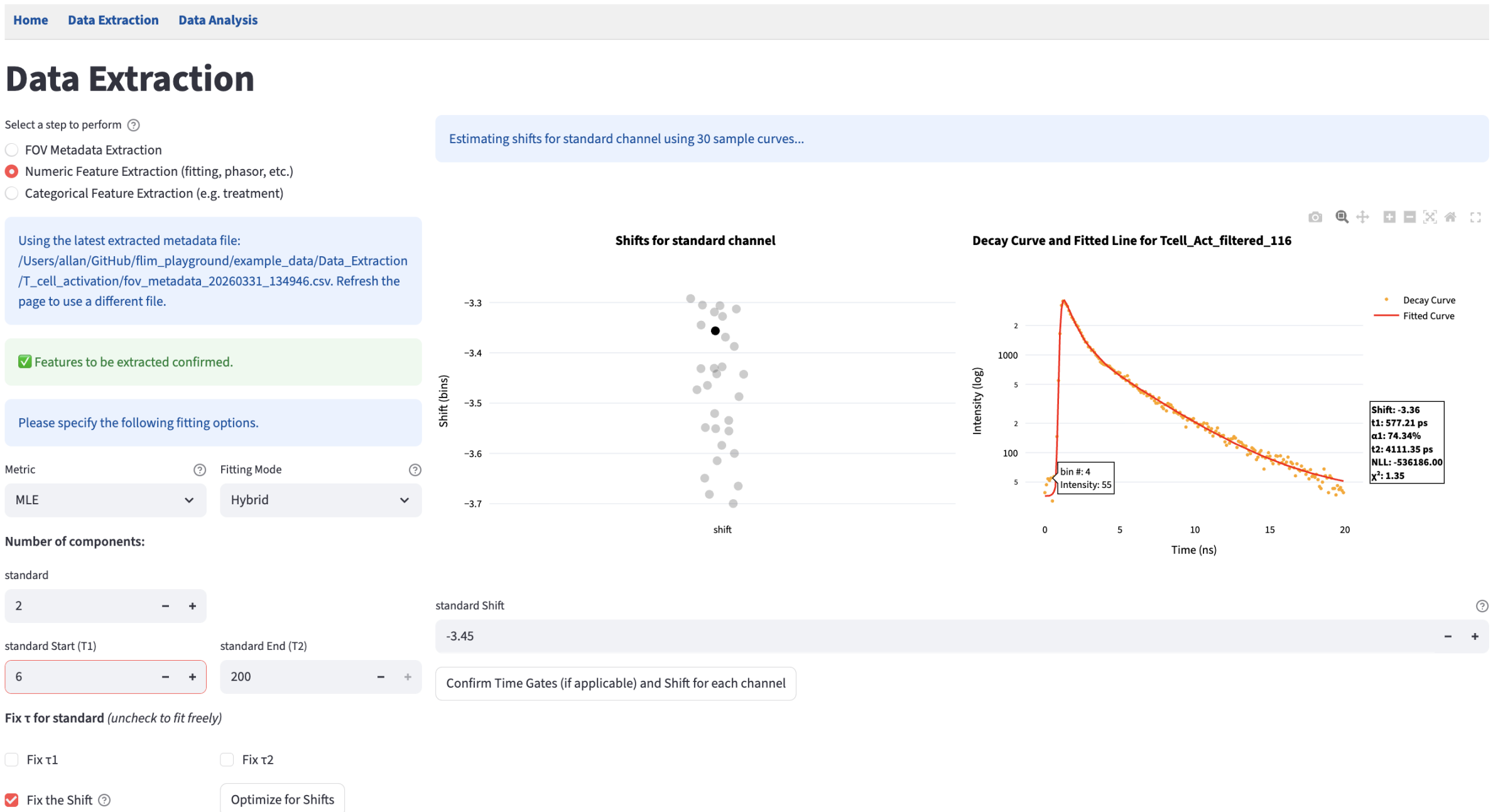

5.2.1.1 Gather High SNR Decay Curves

The high SNR decay curves are constructed automatically based on the decay type. If it is in 2D format, a total of the brightest 30 curves that are below `100000` photons are selected and evenly distributed across all fields of view (one CSV file is considered as one FOV). If it is in 3D/4D format, the fields of view are images, and one curve is constructed for each image that includes all the non-zero pixels within the ROI mask.

5.2.1.2 Reconvolution Fitting

Fitting is essentially an optimization problem: it minimizes the difference between the fitted curve and the measured curve. The fitted curve is modeled by an nth component exponential function convolved with the shifted IRF, and the difference is modeled as an objective metric. Therefore, users are provided with controls over two parts of the fitting process through the fitting options panel:

1. How to construct the objective metric
2. How to perform the optimization

Implementation-wise, FLIM Playground uses the `lmfit` package that takes generic objectives to perform the optimization process that is flexible enough to handle the reconvolution fitting.

Each decay curve is fitted independently. **Fit calibration** (one optimization per high-SNR curve in the IRF shift step) and **Lifetime fit** ROI-level extraction (one optimization per ROI per channel) both

schedule those independent fits **in parallel across CPU cores**, so on a multi-core machine many fits run at once.

If at least one channel has `Lifetime fit` feature extractor selected, the fitting options panel is displayed.

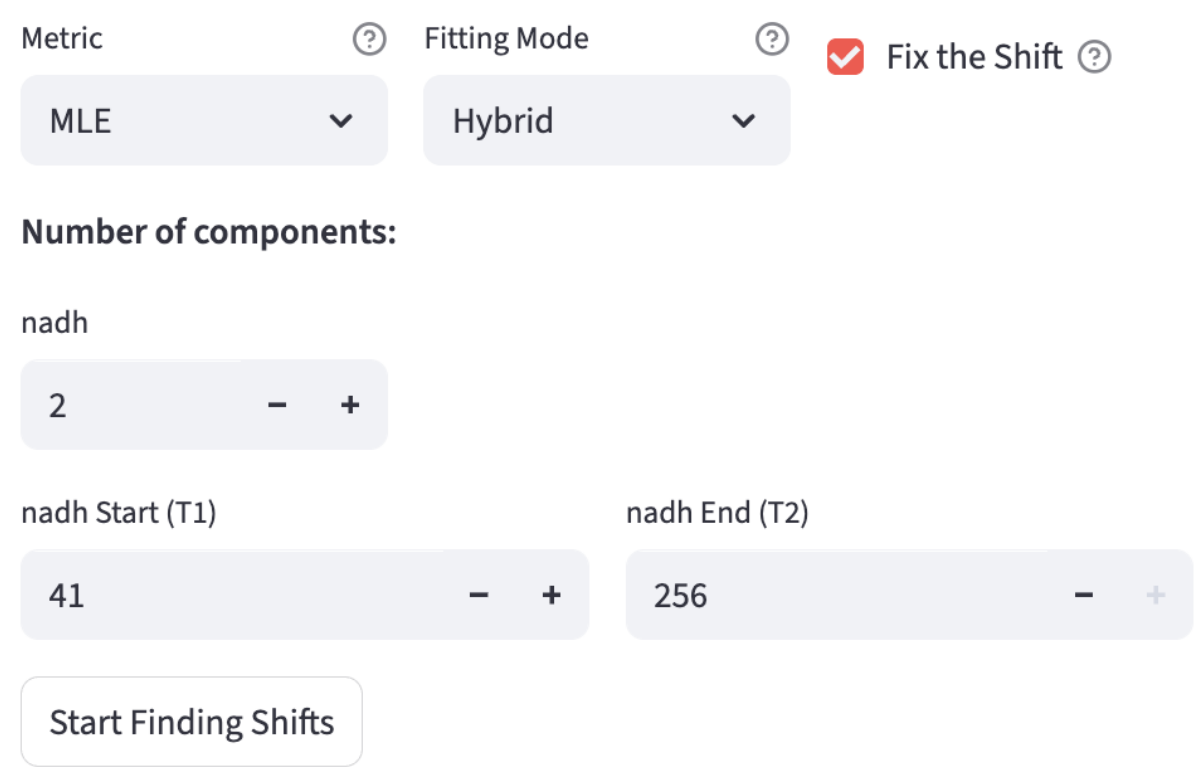

Let's break down the fitting options one by one.

5.2.1.2.1 Number of Components

The number n in the nth component exponential function:

$$I(\mathbf{t}) = \sum_{i=1}^{n} A_i \, e^{-\mathbf{t}/\tau_i}, \qquad \tau_i > 0$$

Therefore, n determines the parameters to be fitted: the amplitudes A_i and the lifetimes τ_i. $\mathbf{t}$ is the time axis of the decay curve calculated by the `duration` and `time bins` from the decay info:

$$\mathbf{t} = [0,\ \Delta t,\ 2\Delta t,\ \ldots,\ (N-1)\Delta t], \quad \text{where } \Delta t = \frac{T}{N}.$$

It supports $n = 1, 2, 3$ components.

Additionally, the constant offset parameter (Z) that represents a time-independent background (i.e., room light) is also optimized[11].

$$\hat{y}(t) = [I(t) + Z] \circledast \mathrm{IRF}(t)$$

where $\circledast$ denotes circular (periodic) convolution, implemented via FFT multiplication (`np.fft.ifft(np.fft.fft(decay) * np.fft.fft(irf))`). Circular convolution correctly accounts for the wrap-around effects inherent in periodic TCSPC decays, where photons arriving after the end of the measurement window appear at the beginning of the next period.

> **Previous Versions (< 1.8.0)**
>
> In versions prior to 1.8.0, the fitting process utilized linear convolution with a cut off:
>
> $$\hat{y}(t) = \left(I(t) * \mathrm{IRF}(t)\right)_{0:N} + Z$$
>
> where $*$ denotes linear convolution and the subscript $(0 : N)$ denotes truncating the result to the length of the original decay curve, effectively ignoring the part of the convolution that extends beyond the measurement window. The difference between this approach and circular convolution is minimal for shorter lifetimes. However, the correct way to fit incomplete decays is formulated above using circular convolution, which is now the standard used in the latest version.

5.2.1.2.2 Fixed Lifetimes

While finding the optimal amplitudes A_i and lifetimes τ_i (when number of components is greater than 1), users can optionally assign fixed values to specific τ_i components during the interactive fitting phase. These explicit constraints remain constant (are not treated as free parameters) while minimizing the cost metric, making the optimization more robust when the true lifetime state is known. The default fixed lifetime values can be initialized in the Data Extraction Configuration.

5.2.1.2.3 Time Gates

Due to the dead time of the system certain time bins from the head and/or tail of the decay curve are not reliable. The T1 (head) and T2 (tail) gates are used to select the time range of the decay curve to be fitted. Users can inspect the decay curve by clicking the full-screen mode button of the plot on the right of the shift result. Hover-based interaction is implemented so users can see the time bin numbers to have a better sense of the time range.

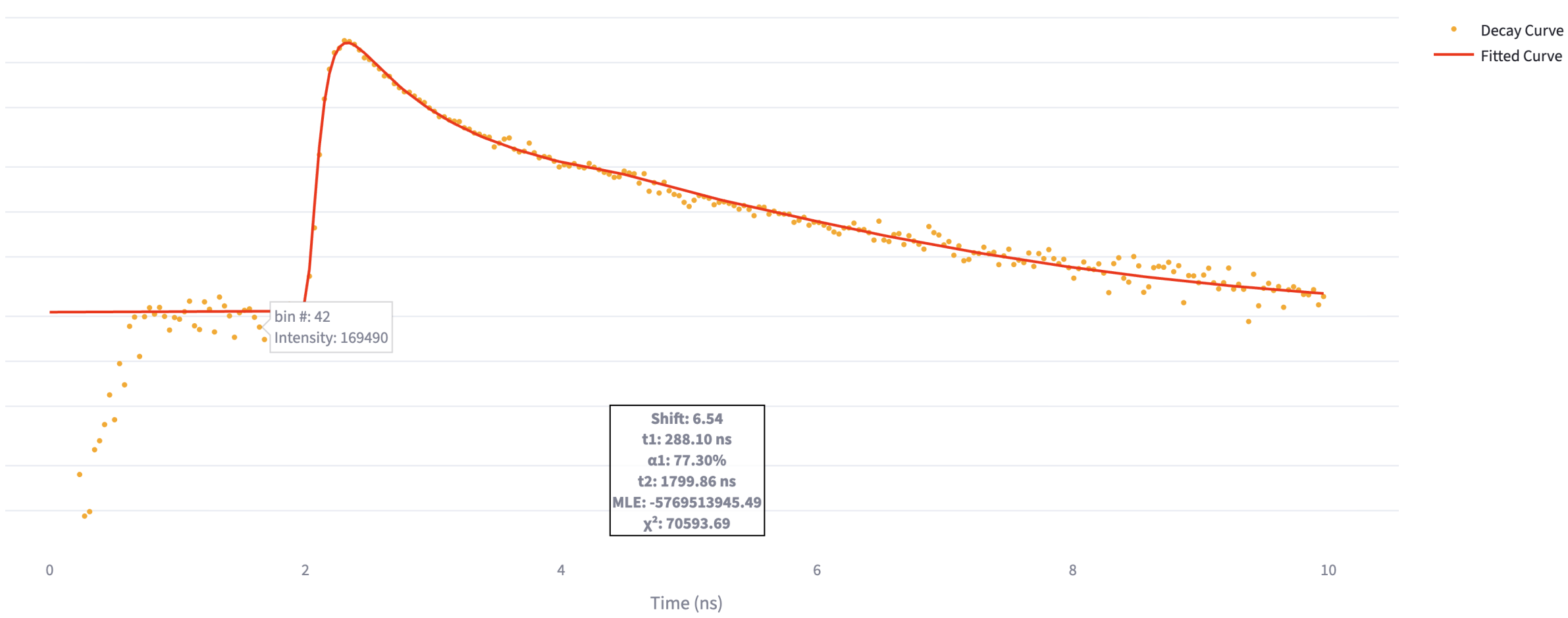

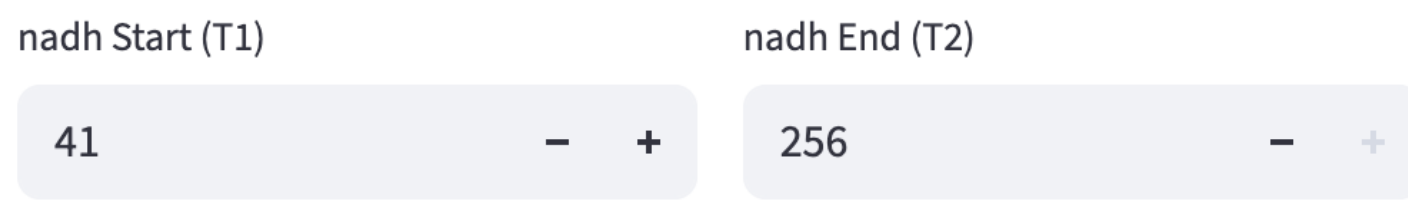

Only the time bins within the T1 and T2 gates are used to calculate the cost metric.

5.2.1.2.4 Metric

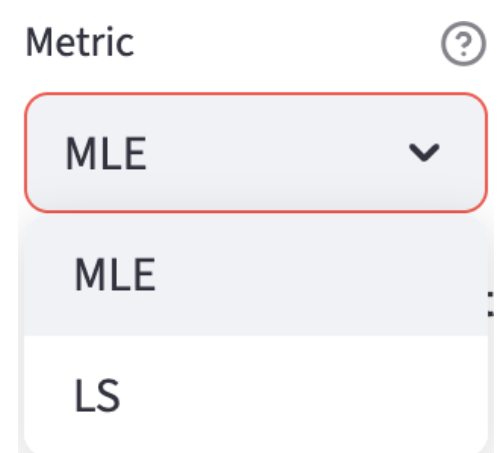

Maximum Likelihood Estimation (`MLE`) estimates the parameters by maximizing the likelihood function, which is the probability of the measured data given the model parameters. A mathematically convenient way to do this is to minimize the negative log-likelihood function:

$$\mathrm{NLL}(\theta; t_s, t_e) = -\sum_{n=0}^{N-1} \mathbf{1}_{[t_s,t_e]}(t_n) \left[y_n \log \hat{y}(t_n) - \hat{y}(t_n) \right].$$

$\mathbf{1}_{[t_s,t_e]}$ is the indicator function that is 1 if t_n is within the time gates $[t_s, t_e]$, and 0 otherwise. y_n is the observed count at time t_n, and $\hat{y}(t_n)$ is the model prediction at t_n.

Least Squares (`LS`) estimates the parameters by minimizing the sum of the squared differences between the measured curve and the model prediction.

5.2.1.2.5 Fitting Mode

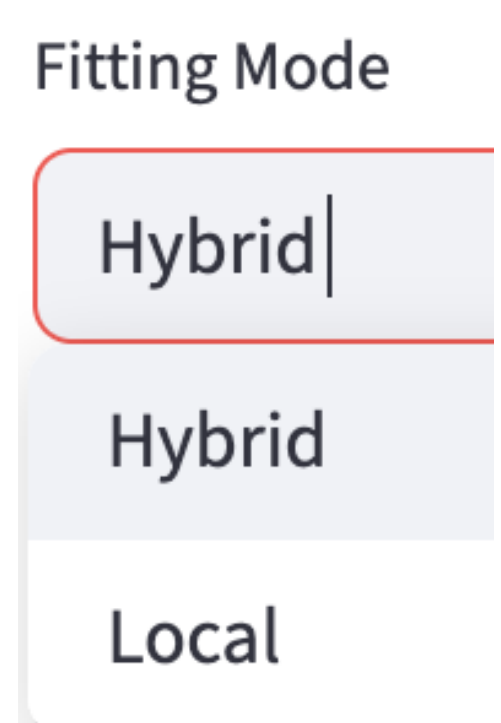

Fit calibration (IRF shift) always runs **Hybrid** optimization to ensure accuracy. For **Lifetime fit** cell-level extraction after calibration, the fitting mode selects the optimization strategy. It is a trade-off between speed and the effort to avoid local minima.

- `Local`: It uses the `leastsq` (least squares with Levenberg-Marquardt) algorithm if the chosen metric is `LS`, otherwise it uses the `nelder` (Nelder-Mead) algorithm.

- `Hybrid`: The slower but more accurate option that combines the `differential evolution` algorithm to obtain a good initial guess for all parameters, followed by the `Local` optimizer to refine the solution.

5.2.2 Fit Free Calibration

Users get to choose between the following two methods to calibrate the IRF shift in the configuration step.

The first step of the calibration is to **subtract an offset value and clip each decay curve to 0 if the time bin is negative**. The offset C is estimated as the mean signal over the final 10% of time bins in each decay ('tail').

Then, depending on the chosen calibration method, the following two methods are available:

5.2.2.1 Shift IRF

It is performed similarly to the fit calibration steps, but in the second step, the shift is not optimized (fitted). It shares the same interface as the fit calibration step, where a scatter plot of the shift values is displayed for each channel, only that the plot is not interactive to show the fit. Instead, it outputs the shift that maximizes the cross-correlation between the IRF and each decay. If `Fix the Shift` is selected, users can specify the shift value that will be applied to all fields of view. Otherwise, the shift value is chosen to be the one that maximizes the cross-correlation between the IRF and the decay.

> **Note**
>
> If both the `Lifetime fit` and `Lifetime fit free` feature extractors are selected for this channel, the optimized IRF shift derived from the fit calibration step is reused for the `Lifetime fit free` feature extractor.

The signals of the shifted IRF are deconvolved from the offset-subtracted decay curves using `phasor.phasor_divide` from the `phasorpy` package.

5.2.2.2 Fluorescence lifetime standard

Because the fluorescence lifetime standard is measured in the same system as the decay curves and we know its lifetime, there is no need to account for the IRF shift and the offset. Therefore, after subtracting the offset, `phasorpy`'s `lifetime.phasor_calibrate` function is used to calibrate all the decay curves behind the scenes when calculating the phasor coordinates.

5.2.3 Confirm Calibration

Once users are satisfied with the calibration settings (fitting options and shift values), they can click the `Confirm Calibration` button at the bottom of the page.

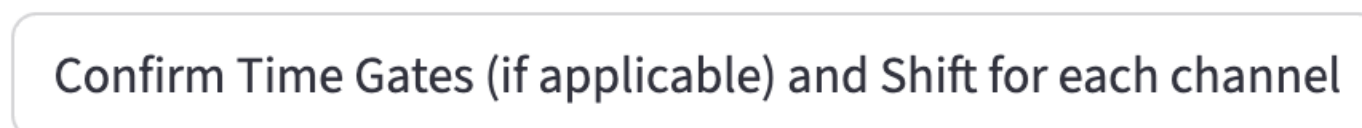

Once confirmed, the left panel prompts users to either apply the calibration settings to the downstream extractors or calibrate again. Users can also save the updated settings (fitting options and shift values) to the metadata file.

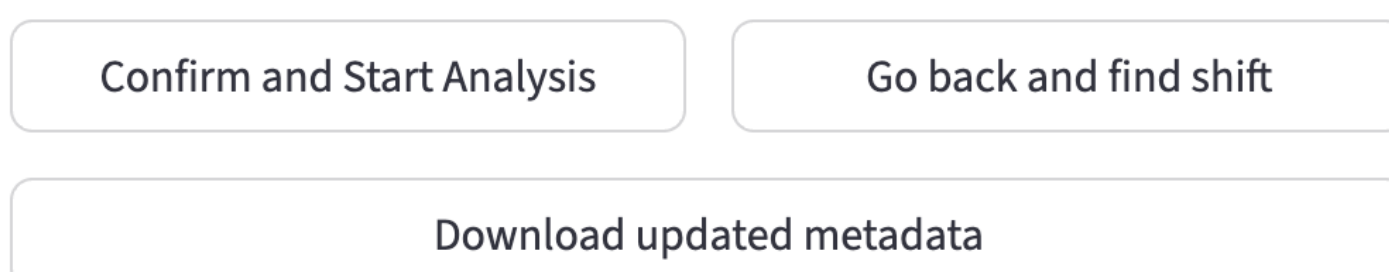

5.3 Feature Extraction

If the selected feature extractors do not require IRF shift calibration, or users have finished the calibration, they can proceed to the feature extraction step by clicking the `Confirm and Start Analysis` button.

Similar to the fov metadata extraction step, FLIM Playground extracts the features and displays the extraction status for each field of view.

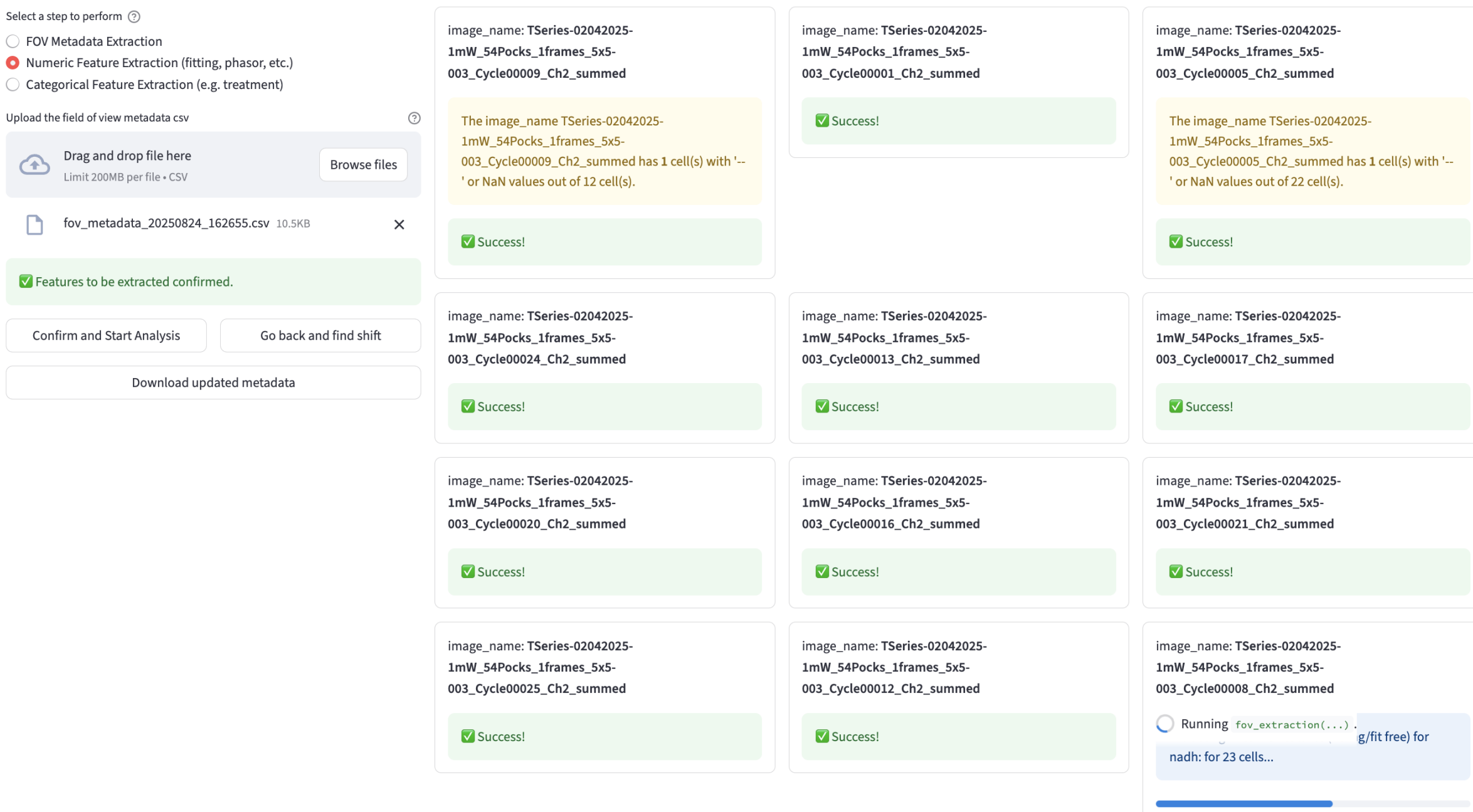

A progress bar is rendered to show the feature extraction progress for each FOV (bottom right).

Warnings are displayed if cells have `NaN` values for some features. They are *not* excluded from the final CSV file.

5.4 Save Results

Once the feature extraction is finished, users can save the results to a CSV file by clicking the `Download` button at the bottom of the page.

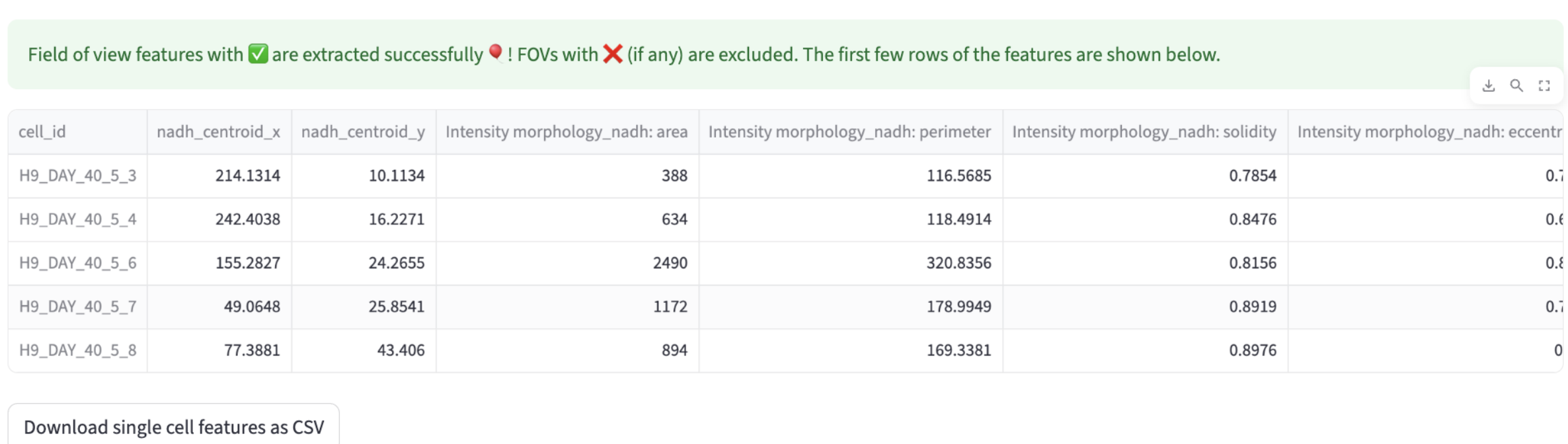

cell_id	nadh_centroid_x	nadh_centroid_y	Intensity morphology_nadh: area	Intensity morphology_nadh: perimeter	Intensity morphology_nadh: solidity	Intensity morphology_nadh: eccentr
H9_DAY_40_5_3	214.1314	10.1134	388	116.5685	0.7854	0.7
H9_DAY_40_5_4	242.4038	16.2271	634	118.4914	0.8476	0.6
H9_DAY_40_5_6	155.2827	24.2655	2490	320.8356	0.8156	0.8
H9_DAY_40_5_7	49.0648	25.8541	1172	178.9949	0.8919	0.7
H9_DAY_40_5_8	77.3881	43.406	894	169.3381	0.8976	0

6 Lifetime Features (Fit)

Biological samples often contain mixtures of fluorescent species, each with its own characteristic lifetime. By modeling the decay as a sum of exponentials, the analysis can separate and quantify these different contributions. For example, NADH in cells exists in both free (short lifetime, ~0.4 ns) and protein-bound (longer lifetime, ~2–3 ns) states; by fitting the fluorescence decay with a bi-exponential model, one can estimate the fraction of each state, which provides direct insight into cellular metabolism and energy production pathways.

FLIM Playground extracts cell-level lifetime fitting features in this feature extractor, including the fraction of each lifetime component (α_i), their lifetimes (τ_i), and mean lifetime (τ_{mean}).

$$I(t) = \sum_{i=1}^{n} A_i \, e^{-t/\tau_i}$$

6.1 Fitting

If the decays have not been pre-fit, FLIM Playground applies the confirmed fitting options (number of components, fixed lifetimes, time gates, metric, and fitting mode) to the same reconvolution fitting process as in the IRF shift calibration step. The IRF shift is no longer a free variable to be optimized. Unlike calibration, which always uses hybrid optimization for shift estimation, users can choose between two fitting modes for cell-level lifetime fitting: **Local** or **Hybrid**. As with calibration, these per-cell fits are parallelized across CPU cores. To recap, the reconvolution fitting minimizes the difference between the measured curve and the fitted curve modeled by a multi-exponential model convolved with the IRF, quantified by the cost metric.

Otherwise, FLIM Playground uses the pre-fit values to calculate the fitting features.

In the final dataset, fitting features are prefixed by the combination of the feature extractor name (i.e. `Lifetime fit`) and the channel name, allowing Data Analysis to group the features. For example, `Lifetime fit_nadh: a1` means the fraction of the first lifetime component for the NAD(P)H channel.

6.1.1 Preprocessing

It sums up all the pixel decays belonging to the same cell ROI labeled by the ROI mask as one decay curve[9]. The ROI summing reduces variability and bias and allows for short integration times at acquisition in exchange for sub-cellular resolution (i.e., pixel-level). Users do not need to specify the bin factor (each pixel sums up the surrounding pixels' decay curves) to account for insufficient photon counts. ROI-level aggregation also keeps the workload manageable; independent ROI fits are then run in parallel across CPU cores.

It also shifts the IRF using the shift values from the IRF shift calibration step. To shift an IRF, FLIM Playground upsamples the IRF 10 times using linear interpolation to fill the gaps. Then it shifts the IRF by the shift values $\times 10$ and downsamples the IRF back to the original size.

6.1.2 Fraction of components

In addition to the absolute amplitudes of each component directly from the fitting result, FLIM Playground calculates the fraction of each component, α_i, as the amplitude of the component divided by the sum of all amplitudes. This normalization allows lifetime to be independent of the absolute intensity of the signal.

6.1.3 Mean lifetime

The mean lifetime, τ_{mean}, is calculated as the weighted average of the lifetimes of all components, where the weights are the fractions of each component.

$$\tau_{mean} = \sum_{i=1}^{n} \alpha_i \tau_i$$

6.2 Pixel pre-fit

Currently, FLIM Playground supports pixel pre-fit lifetime features from SPCImage. The pixel-level lifetime fitting features are expected to be stored in 2D arrays in spatial dimensions, with each row and column having the value of a lifetime feature outputted from SPCImage. SPCImage assigns `0` for pixels that are not fitted (e.g. thresholded out). To avoid them biasing the results, FLIM Playground uses `np.ma.masked_array` to create a masked array and disregards them when calculating the averages using `np.ma.average`.

Then it uses the ROI mask to calculate the cell-level lifetime features by averaging the pixel-level features within each ROI.

7 Lifetime Features (Fit-Free)

Phasor analysis provides a fast, model-free view of lifetimes by transforming fluorescence decays into phasor coordinates through a Fourier transform. This approach is considered “fit-free” because it does not require iterative curve fitting or predefined decay models—each decay is mapped directly to a point on the phasor plot. It has the nice property that mono-exponential decays fall on the universal semicircle while mixtures lie inside as linear combinations, so clusters reveal distinct species and their fractional contributions. FLIM Playground currently extracts cell-level phasor features: `g`, `s`, `Tau_phase`, `Tau_m`, `g_2nd` (2nd harmonic), and `s_2nd`.

Fit-free features in the final dataset are prefixed by the combination of the feature extractor name (i.e. `Lifetime fit free`) and the channel name, allowing Data Analysis to group the features. For example, `Lifetime fit free_nadh: G` means the g coordinate for this cell in the NAD(P)H channel.

7.1 Preprocessing

It sums up all the pixel decays belonging to the same cell ROI labeled by the ROI mask as one decay curve[9]. The phasor features are calculated from the summed decay curve.

If the chosen calibration method is IRF shift calibration, FLIM Playground shifts the IRF using the shift values and subtracts the estimated offset as described in the IRF shift calibration step.

7.2 Phasor features

7.2.1 Step 1: raw phasor coordinates

The reference implementation from phasorpy assumes the decay curve comes from one period and time bins are evenly spaced (untruncated). So, their calculation is frequency (f) and time (T) independent. However, there are cases when the decay curve is truncated due to the deadtime of the system. For example, when the laser frequency is $f = 0.08$ GHz, the theoretical period is $T = 12.5$ ns, but in practice the time window is truncated to $T = 10$ ns. FLIM Playground applies a custom implementation to the truncated decay curve. (The `sample_phase` option can be used to account for the truncation but it does not handle the second or higher harmonics)

7.2.1.1 Untruncated decay curve

It uses the phasor_from_signal function from the phasorpy package to calculate the uncalibrated phasor coordinates g_raw and s_raw, g_raw_2nd and s_raw_2nd, the first and second harmonics' real and imaginary parts, from the cell decay curve.

Either g_irf, s_irf, g_irf_2nd, and s_irf_2nd from the IRF, or ref_mean, ref_real, ref_imag from the fluorescence lifetime standard, depending on the chosen calibration method, are calculated using the same function.

Mathematically, the raw phasor coordinates are calculated as (copied from the phasorpy documentation):

$$F_{\mathrm{DC}} = \frac{1}{K}\sum_{k=0}^{K-1} F_k, \quad g_{\mathrm{raw}} = \frac{1}{F_{\mathrm{DC}}}\frac{1}{K}\sum_{k=0}^{K-1} F_k \cos\left(2\pi h \frac{k}{K}\right), \quad s_{\mathrm{raw}} = \frac{1}{F_{\mathrm{DC}}}\frac{1}{K}\sum_{k=0}^{K-1} F_k \sin\left(2\pi h \frac{k}{K}\right)$$

where K is the number of time bins, F_k is the decay curve, and h is the harmonic number.

7.2.1.2 Truncated decay curve

The phasor coordinates of the IRF and the decay curves of both harmonics are calculated using the time axis $t_k = k\,(T/K)\ \ (k = 0, \dots, K-1)$ and the angular frequency $\omega = 2\pi\, h f$:

$$g_{\mathrm{raw}} = \frac{\sum_{k=0}^{K-1} F_k \cos(\omega\, t_k)}{\sum_{k=0}^{K-1} F_k}, \quad s_{\mathrm{raw}} = \frac{\sum_{k=0}^{K-1} F_k \sin(\omega\, t_k)}{\sum_{k=0}^{K-1} F_k}$$

This formula is equivalent to the untruncated formula when $fT = 1$.

For the fluorescence lifetime standard, the phasor coordinates are calculated using phasorpy's phasor_from_signal and the sample_phase option is set to ωt_k.

7.2.2 Step 2: calibrated phasor coordinates

To get the calibrated phasor coordinates g, s, g_2nd, and s_2nd, FLIM Playground uses either

```
from phasorpy import phasor
g, s = phasor.phasor_divide(g_raw, s_raw, g_irf, s_irf)
g_2nd, s_2nd = phasor.phasor_divide(g_raw_2nd, s_raw_2nd, g_irf_2nd, s_irf_2nd)
```

or

```
from phasorpy import lifetime
g, s = lifetime.phasor_calibrate(g_raw, s_raw, ref_mean, ref_real, ref_imag, frequency=laser_
g_2nd, s_2nd = lifetime.phasor_calibrate(g_raw_2nd, s_raw_2nd, ref_mean, ref_real, ref_imag,
```

The `laser_rate` is specified during the fov metadata extraction and the configuration interface.

7.2.3 Step 3: phasor-derived lifetime

`Tau_phase` and `Tau_m` are calculated using the first harmonic's phasor coordinates `g` and `s`.

```
import numpy as np
w = 2 * np.pi * laser_rate
phi = np.arctan2(s, g)
m = np.sqrt(g**2 + s**2)
tau_phase = 1/w * np.tan(phi)
tau_m = 1/w * np.sqrt(1/m**2 - 1)
```

$$\phi = \text{atan2}(s, g), m = \sqrt{g^2 + s^2}, \tau_\phi = \frac{\tan\phi}{\omega}, \tau_m = \frac{1}{\omega}\sqrt{\frac{1}{m^2} - 1}$$

8 Morphological Features

FLIM Playground extracts single-cell morphological features based on the cell region of interest (ROI) mask of each *channel*. By inputting the ROI mask to `skimage.measure.regionprops`, the following features are extracted:

- `area`: the number of pixels in the ROI.
- `perimeter`: length of the ROI boundary in pixels.
- `solidity`: Solidity $= \frac{\text{\# ROI pixels}}{\text{\# convex hull pixels}}$, where the convex hull is the smallest convex polygon that contains the ROI. A value close to 1 means the shape has few concavities (more solid), while lower values indicate more irregular boundaries.
- `eccentricity`: equals the eccentricity of the ellipse that has the same second moments as the region. It is given by the ratio of the focal distance (the distance between the foci) to the length of the major axis: $e = \frac{c}{a}, \quad e \in [0, 1)$, where a is the semi-major axis length, b is the semi-minor axis length, and $c = \sqrt{a^2 - b^2}$ is the focal distance. When $e = 0$, the ellipse reduces to a circle. It quantifies how elongated the ROI is, with 0 being a perfect circle and 1 being a line.
- `major_axis_length`: the length of the major axis of the ellipse that has the same second moments as the region: $2a$. It represents the longest dimension of the ellipse that approximates the region's shape, essentially describing its maximum elongation.
- `minor_axis_length`: the length of the minor axis of the ellipse that has the same second moments as the region: $2b$. It represents the shortest dimension of that ellipse, capturing the region's minimum elongation.
- `circularity`: describes how close the ROI is to a perfect circle. It is defined as circularity $= \frac{4\pi A_{\text{ROI}}}{P_{\text{ROI}}^2}, \quad \text{circularity} \in (0, 1]$, where A_{ROI} is the area of the region and P_{ROI} is its perimeter. A value of 1 corresponds to a perfect circle.

Feature names in the final dataset are prefixed by the combination of the feature extractor name (i.e. `Intensity morphology`) and the channel name, allowing Data Analysis to group the features. For example, `Intensity morphology_nadh: area` means the area of each cell in the NAD(P)H channel.

9 Textural Features

FLIM Playground extracts single-cell texture features based on the intensity image and the ROI mask from each acquisition *channel*. For an example application, refer to this paper that used CellProfiler to extract the texture features.

The first step is to create a single-cell intensity image for each cell based on the ROI mask and the intensity image. Then a list of texture features (the list may be extended in the future) is calculated:

9.1 Granularity

$Granularity_n$ is the percentage of intensity contributed by bright objects of n pixels in radius. It is computed by performing a morphological opening with a disk of radius n on the cell intensity image and measuring the intensity lost as a percentage of the total intensity of the original cell ROI.

$n = 1, 3, 5, 7, 9$ are computed.

Semantically, higher granularity at smaller scales implies higher fragmentation.

9.2 Radial Distribution

To calculate the radial distribution, each cell intensity image is partitioned into 4 concentric rings, and the intensity fraction over the total intensity for each ring is calculated. For example, a higher fraction in ring 1 implies that signals are more concentrated at the center of the cell, whereas a higher fraction in ring 4 implies that signals are more concentrated at the cell edge.

9.3 Mass Displacement

Mass displacement is the Euclidean distance (in pixels) between the intensity-weighted centroid of the cell ROI and the geometric centroid of the same ROI.

9.4 Intensity Sum

Intensity sum is defined as the sum of pixel intensities within the cell intensity image.

In the final dataset, feature names are prefixed by the combination of the feature extractor name (i.e. `Intensity texture`) and the channel name, allowing Data Analysis to group the features. For example, `Intensity texture_nadh: granularity_1` means the percentage of intensity removed when bright objects of 1-pixel diameter are removed in the NAD(P)H channel.

> **Visual explanation**
>
> This post provides a visual explanation of and code to calculate some of the texture features above.

10 Categorical Feature Extraction

Based on the datasets extracted in the numerical feature extraction step, FLIM Playground:

- merges the datasets into a single dataset
- assigns categorical features to each cell in the merged dataset

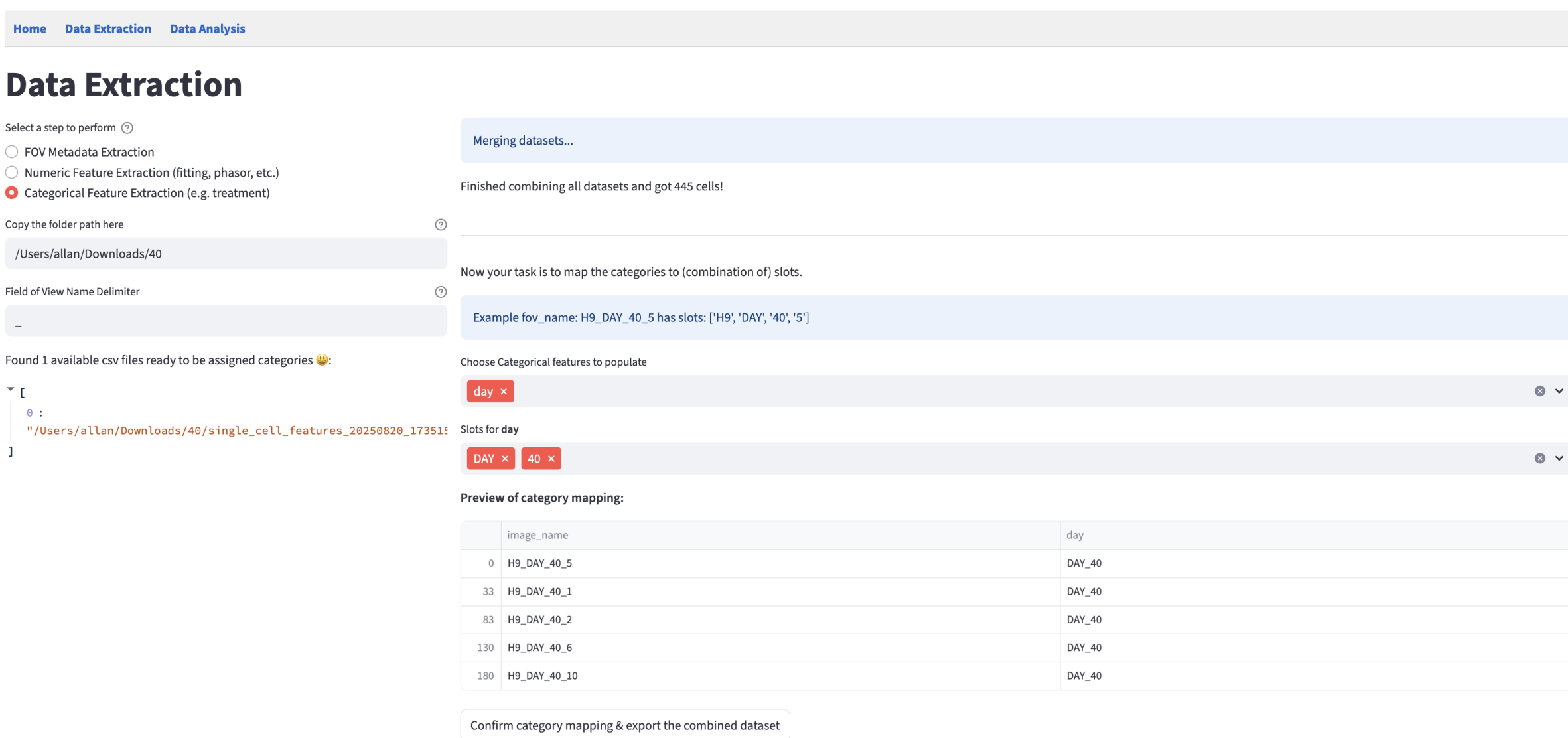

10.1 Merging Datasets

It scans the specified folder path and finds all CSV files. It uses the cell identifier column to check for duplicates both within each dataset and across all datasets.

> **!** Important
>
> **The FOV identifier uniquely identifies a field of view and is assumed to contain all information necessary to extract the categorical features (e.g., treatment, time point, etc.), by including segments separated by a certain delimiter (e.g., _). Categorical features are assigned from individual segments or their combinations.** Example: The FOV `Panc1_Cyanide_dish1`

> denotes that it comes from the Panc1 cell line, under the Cyanide treatment, in the 1st dish.

Therefore, it checks if all field-of-view names include the same number of segments to determine the eligibility of categorical feature assignment.

Field of View Name Delimiter

_

The image_name column in /Users/allan/Downloads/DATA/donor6.csv has different number of parts. For example, check fov_name: HC220113_1n.

The image_name column in /Users/allan/Downloads/DATA/donor5.csv has different number of parts. For example, check fov_name: HC130319_1n.

Found 7 available csv files ready to be assigned categories 😄:

```
[
  0 : "/Users/allan/Downloads/DATA/donor8.csv"
  1 : "/Users/allan/Downloads/DATA/donor9.csv"
  2 : "/Users/allan/Downloads/DATA/donor1.csv"
  3 : "/Users/allan/Downloads/DATA/donor2.csv"
  4 : "/Users/allan/Downloads/DATA/donor3.csv"
  5 : "/Users/allan/Downloads/DATA/donor7.csv"
  6 : "/Users/allan/Downloads/DATA/donor4.csv"
]
```

It merges the datasets that pass the checks by finding common columns and vertically concatenating them.

Merging datasets...

Merging dataset 2 (/Users/allan/Downloads/DATA/donor9.csv) into the combined dataset.

Merging dataset 3 (/Users/allan/Downloads/DATA/donor1.csv) into the combined dataset.

Merging dataset 4 (/Users/allan/Downloads/DATA/donor2.csv) into the combined dataset.

Merging dataset 5 (/Users/allan/Downloads/DATA/donor3.csv) into the combined dataset.

Merging dataset 6 (/Users/allan/Downloads/DATA/donor7.csv) into the combined dataset.

Merging dataset 7 (/Users/allan/Downloads/DATA/donor4.csv) into the combined dataset.

Finished combining all datasets and got 3037 cells!

10.2 Assigning Categorical Features

Available categorical features to be assigned are drawn from the user-specified configuration.

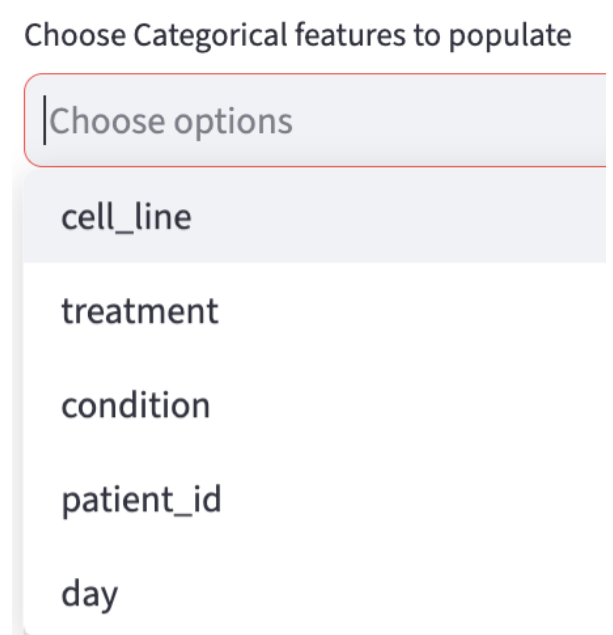

Based on the assumptions, it breaks down the FOV identifier into individual fields and facilitates the assignment.

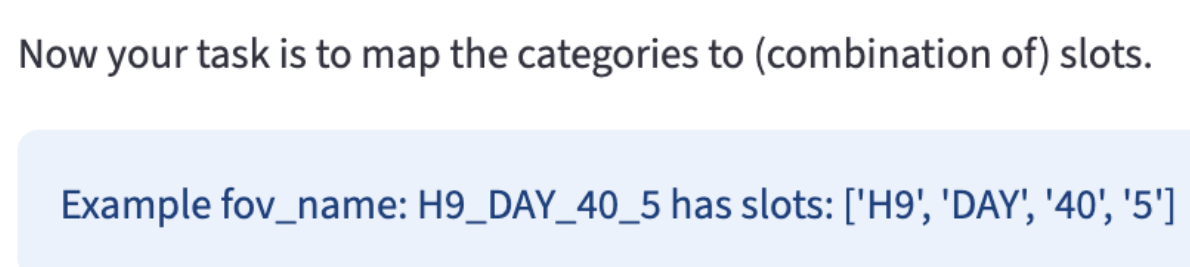

A live preview of the assignment is displayed.

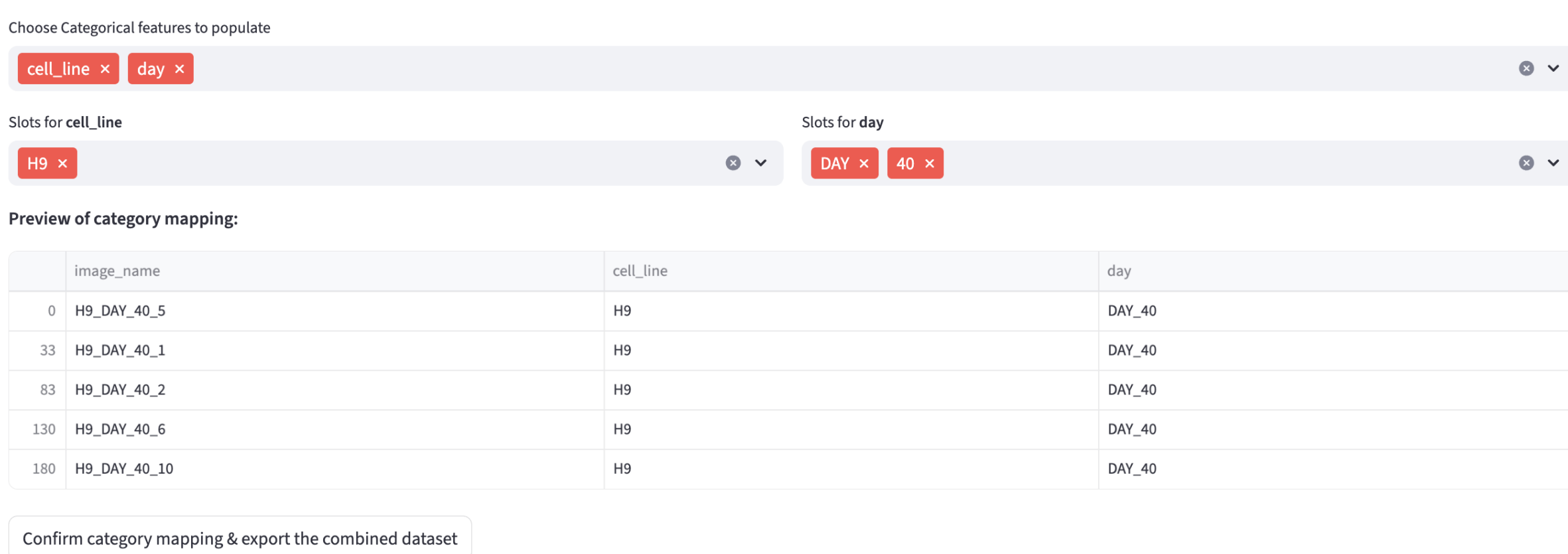

And the merged dataset with categorical features is available for download.

Part II

Data Analysis

11 Overview

We are not done yet 😱! FLIM Playground also provides a suite of methods to analyze and visualize the extracted data (either using the Data Extraction section or in users' own way). They are designed to be interactive and frictionless ⚡ so that users can perform hassle-free exploration of their data with, hopefully, fun 😎. Inspired by the same data categorization in Data Extraction, the Data Analysis section designs an architectural blueprint that all in-house analysis methods build on. It is modularized so that new methods and features can be added in the future easily.

11.1 General Workflow

- Select numerical feature(s) on the left.
- Subset the data to find data of interest on the top. ***All the later interactions are based on this filtered dataset.***
- Use visual channels widgets to look at the data in different ways.
- Change the plot style.
- Hover to find data points of interest.
- Interact with method-specific widgets that typically are associated with a modeling method.
- Once users are satisfied with the analysis (visualizations and statistical modeling results), they can export the entire analysis workflow as a self-contained Python script! 🎉

The visualization and analysis results are updated in real time to reflect users' interactions with any of the above widgets.

11.2 Methods

- Depending on the number of numerical features in the analysis, the methods are categorized into 3 groups:
  - Univariate Analysis
    - Feature Comparison
    - Feature Histogram
  - Bivariate Analysis
    - 2D Feature Distribution

        * Phasor Analysis

    – Multivariate Analysis

        * Dimension Reduction
        * Classification

All the methods share a set of interactive widgets. They also have their own method-specific widgets, the descriptions of which are provided in the corresponding method pages.

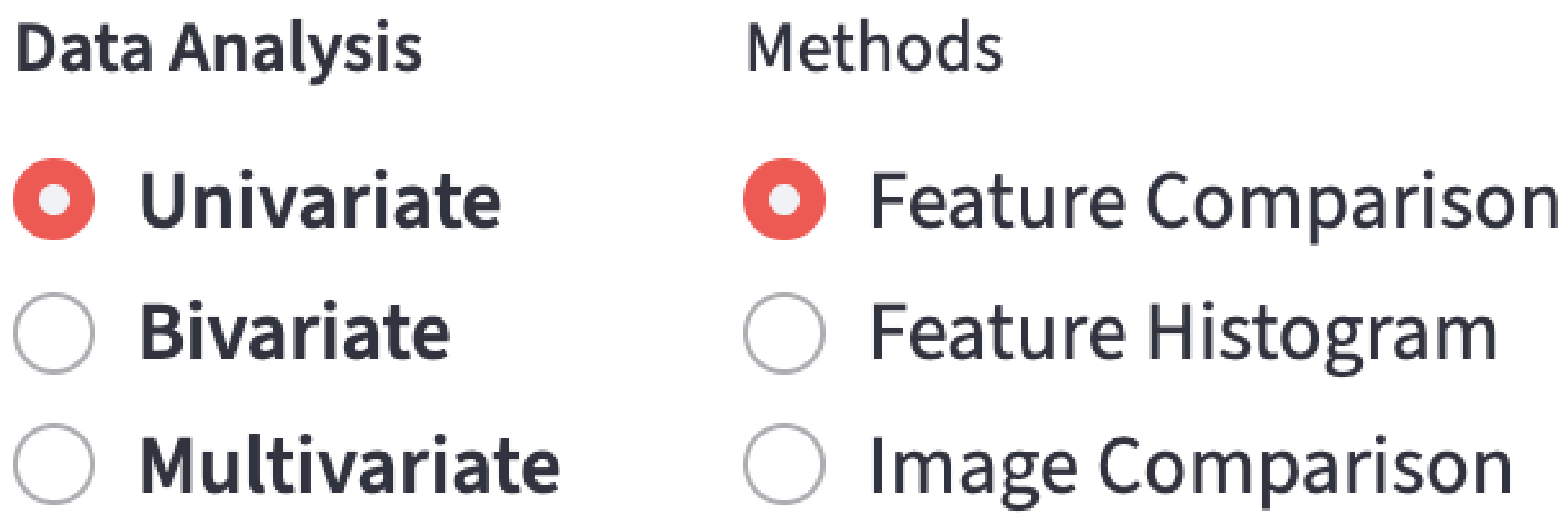

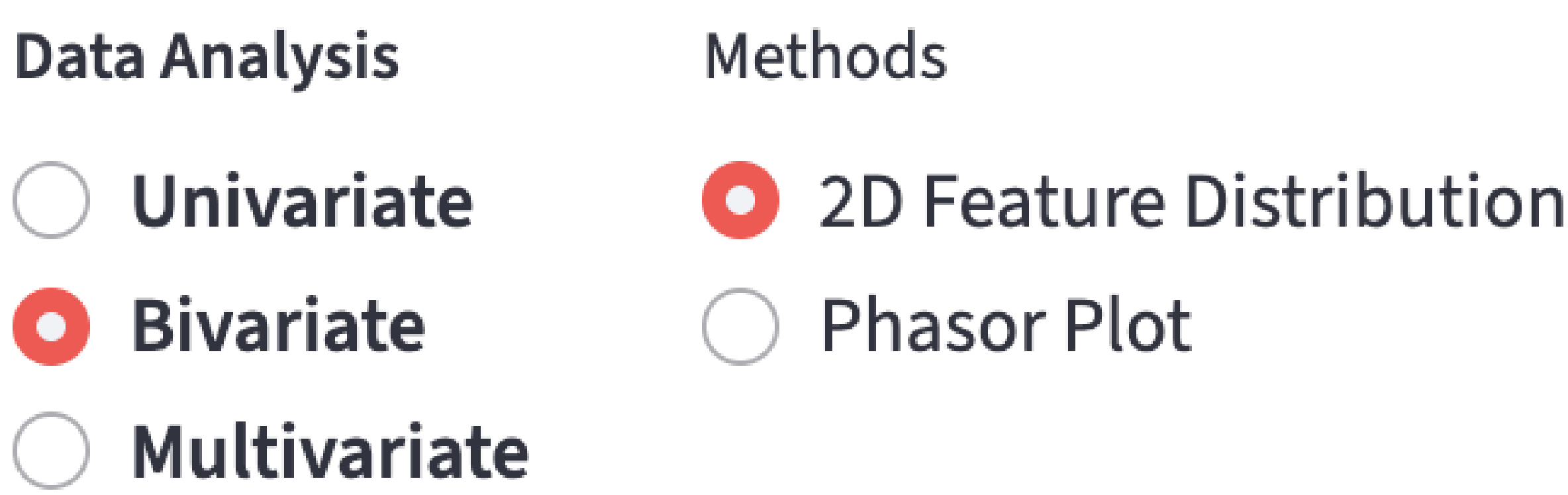

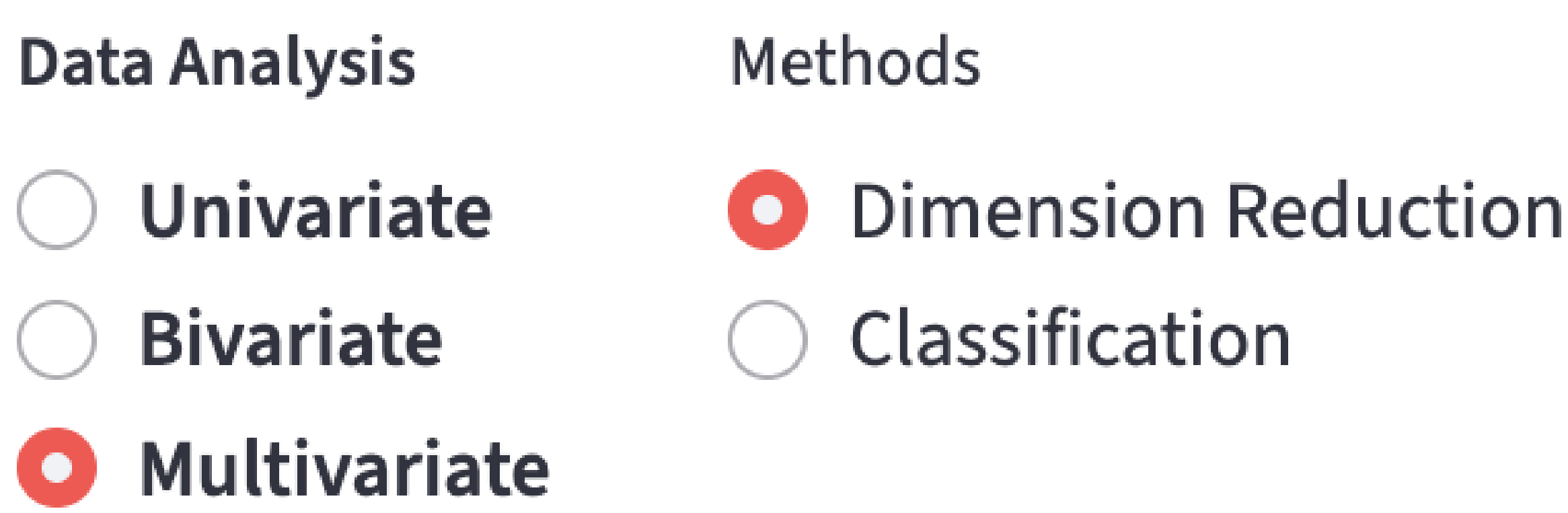

11.3 Input

Users can upload the dataset output from the Data Extraction directly or *their own datasets* in **CSV** (Comma Separated Values) format after finishing the interactive configuration setup.

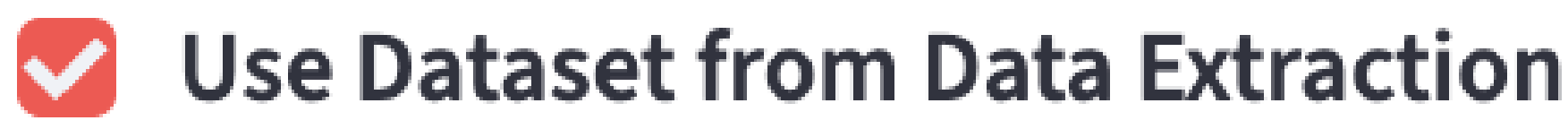

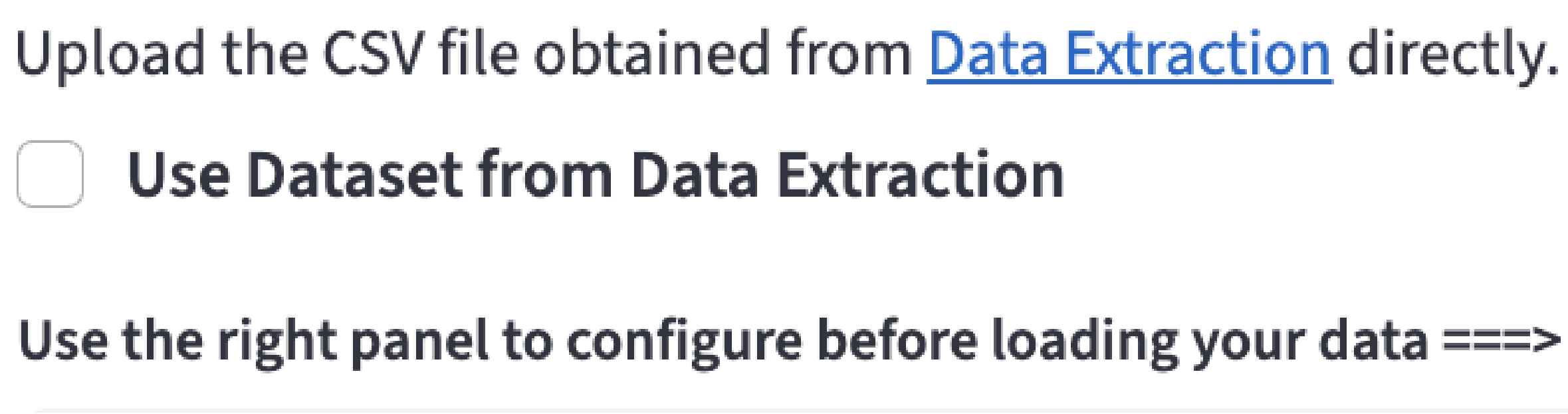

11.3.1 Requirements

> **!** Important
>
> In either case, following the categorization, the dataset should have:
>
> - a column that uniquely identifies each row
> - an (optional) field of view identifier column
>
> - a set of numerical features
> - zero or more categorical features (e.g. treatment, day, patient id, etc.)

The unique row id and field of view id are used to help users identify the row (e.g. a single cell) and field of view (e.g. a single image) of data of interest through the built-in hover-based interaction. Numerical and categorical features are used to render shared widgets. Internally, FLIM Playground will check whether the dataset fulfills the requirements and output meaningful warning or error messages.

> Tip
>
> If you cannot find a certain **numerical feature** under any numerical widgets including `Uncategorized Features`, please inspect the dataset (e.g., using Excel filters) and look for non-numeric values in that column. One non-numeric value (e.g., "–") will prevent it from being recognized.

FLIM Playground searches for **categorical features** in the uploaded dataset based on the user-specified configuration if the dataset is not extracted by Data Extraction. Otherwise, it searches for categorical features specified in the Data Extraction configuration.

11.3.2 Warning Messages

- `Empty columns`: will be dropped.
- `Duplicated columns`: Only the first occurrence of the duplicated column will be kept. Other occurrences will be dropped.
- `Duplicated rows based on the unique row id`: Only the first occurrence of the duplicated row will be kept. Other occurrences will be dropped.
- `Columns with NaN values`: won't be dropped, just a warning message.
  - The analysis will be performed on the rows that are not NaN in the selected numerical features.

11.3.3 Error Messages

- Missing a column that uniquely identifies each row
- Cannot identify any numerical feature column
  - It uses `pd.api.types.is_numeric_dtype` to check if a column is numerical.

> **Note**
>
> Warning messages will not prevent the analysis but error messages will.

11.4 Shared Interactive Widgets

A list of shared widgets is provided to support the general workflow in analysis.

11.4.1 Numerical Selection Widgets

The Data Extraction recognizes numerical features using `pd.api.types.is_numeric_dtype` internally. Then it groups numerical features based on the feature extractor type and the channel it belongs to. For user-provided datasets, the numerical features are grouped based on the user-specified configuration. Ungrouped numerical features are put into the `Uncategorized Features` group. One selection widget is rendered for each numerical feature group.

11.4.1.1 Univariate Analysis

One single-select widget is rendered per group; choosing a feature in any group clears the selections and resets the others to `Select`.

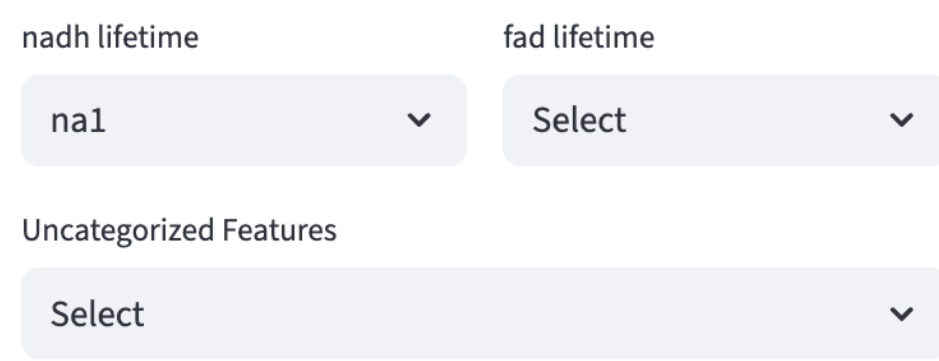

11.4.1.2 Bivariate Analysis

Two sets of selection widgets are rendered to allow maximum flexibility (the two features can be from the same or different feature groups). Each set of selection widgets behaves like the selection widgets in the univariate analysis. The first selected feature will be hidden in the second set of selection widgets.

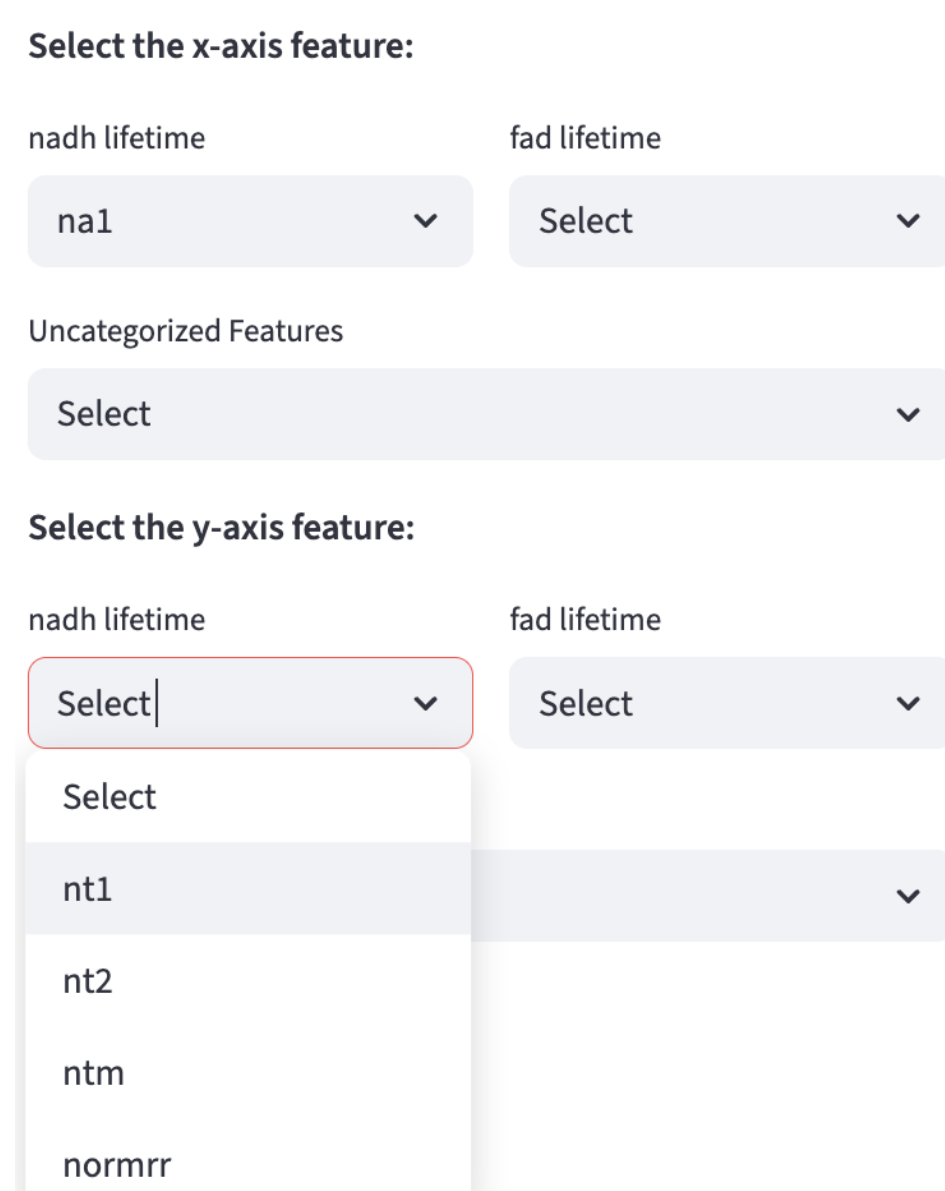

11.4.1.3 Multivariate Analysis

One set of selection widgets, each can select multiple features from a feature group, is rendered. A special value `All` is introduced so that users can conveniently select all features under the feature group. If users select `All`, all the other options will be cleared, and vice versa.

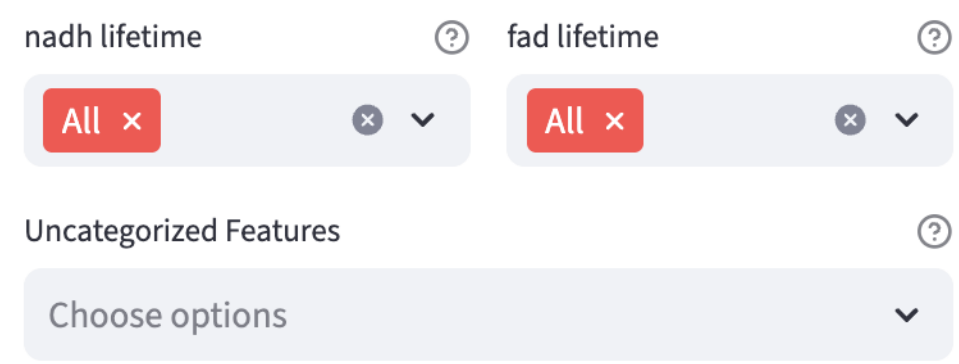

11.4.2 Filter Widgets

11.4.2.1 Categorical Filters

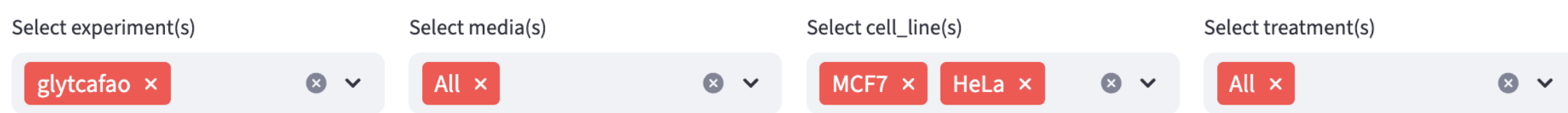

For complex datasets that are collected over multiple days, experiments, treatments, etc., it is useful to filter the data to focus on a subset of the data (data of interest). One filter widget is rendered for each categorical feature so that users have the flexibility to filter the data based on combinations of categorical features. `All` is a special option that include all categories of the selected categorical feature. Once it is selected, all the other options are cleared, and vice versa.

11.4.2.2 Numerical Filters

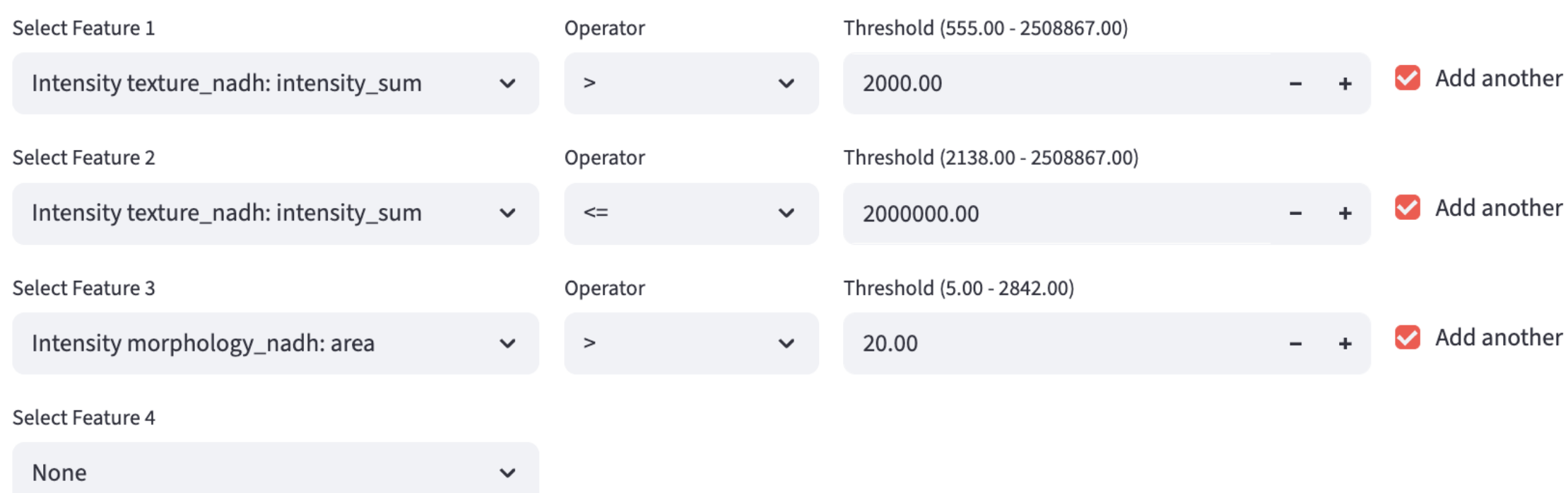

Users can further filter the data based on numerical features. Each row specifies a condition to filter the data:

- `Feature`: the numerical feature to filter on
- `Operator`: > or <=
- `Threshold`: the range of values to filter on, dynamically updated based on previous filters, is displayed for reference. The entered value should be within the range.
- `Add Another`: add a new condition in the next row.

After the two sets of filters are applied, the final filtered data is used for visualization and analysis.

! Important

Make sure you clearly state all numerical filters used.

11.4.3 Visual Channels Widgets

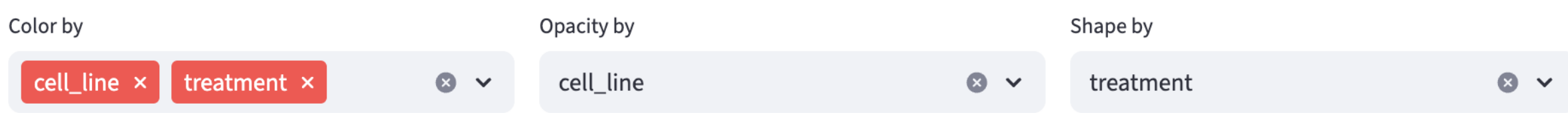

Human vision is wired for rapid, parallel pattern and trend detection; good visual encodings (how to map data to visual elements such as color, shape, opacity, etc.) harness this to surface insights that raw numbers or text obscure[12]. FLIM Playground provides `color`, `opacity`, and `shape` channels for visualizations that support them:

11.4.3.1 Color by

Color is supported in all methods except for Classification. In `Color by`, users can select multiple categorical features, and each unique combination of available categories in selected features is assigned a distinct color. Groups created by `Color by` show up in the x-axis (if multiple features, categories from each feature are delimited by `::`).

Note 1: How to order the groups

The order of the groups in x-axis and in the legend is determined by:

- the order of the selected features: categories of the first feature appear before those of the second, and so on and so forth. For example, the two treatments of `Panc1` are together, because it is sorted first on the cell lines, then on treatments. If you want the treatments to be together, in `Color by` you can select `treatment` then `cell_line`.
- *Numeric-alphabetical sort*: within each feature, the category order is determined by the number inside (e.g. `1` in `Panc1`) first, then the alphabetical order of the category name. Therefore, although `P` is after `M` alphabetically, the `1` is before `7` numerically, making `Panc1` appear before `MCF7`. This may be helpful when you have data from different days or hours. The default string sort will put `Day 100` before `Day 30`, and this is not what we want.

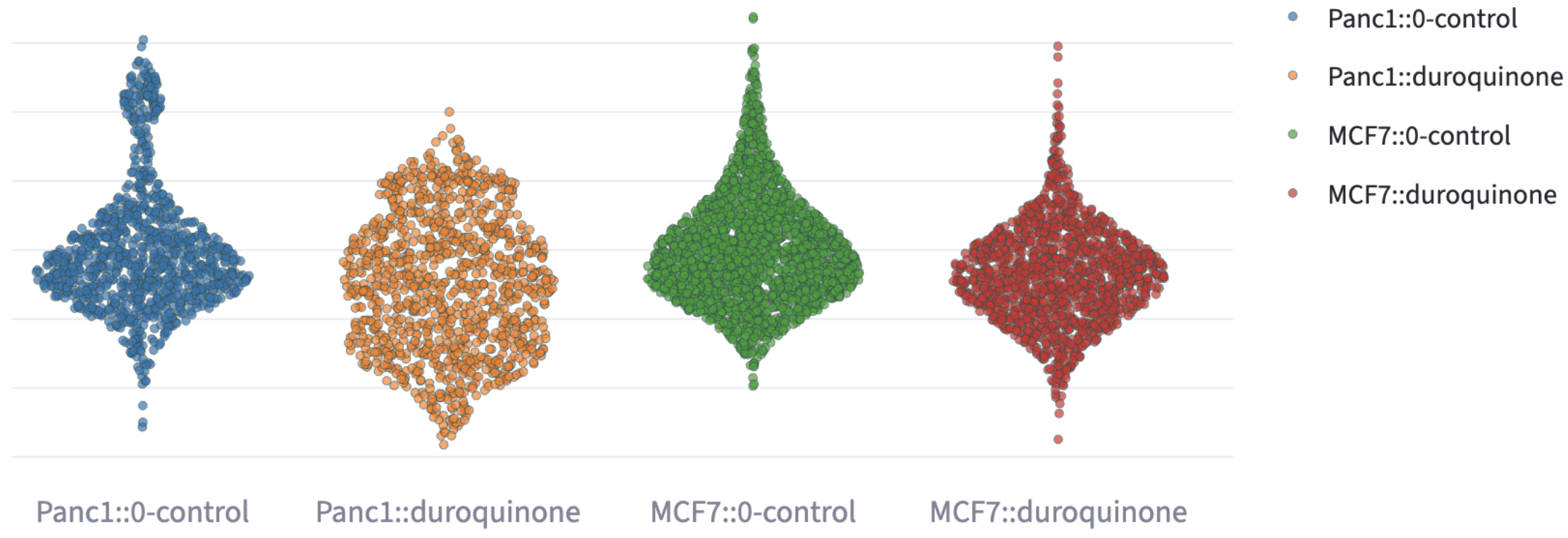

11.4.3.2 Opacity and Shape by

Opacity and shape are supported in all point-based visualizations (e.g. Feature Comparison, 2D Feature Distribution, Phasor Analysis, Dimension Reduction). In `Opacity by` and `Shape by`, users can select one categorical feature, and each unique category is assigned a distinct opacity or shape. The order of the shape and opacity is also sorted numeric-alphabetically.

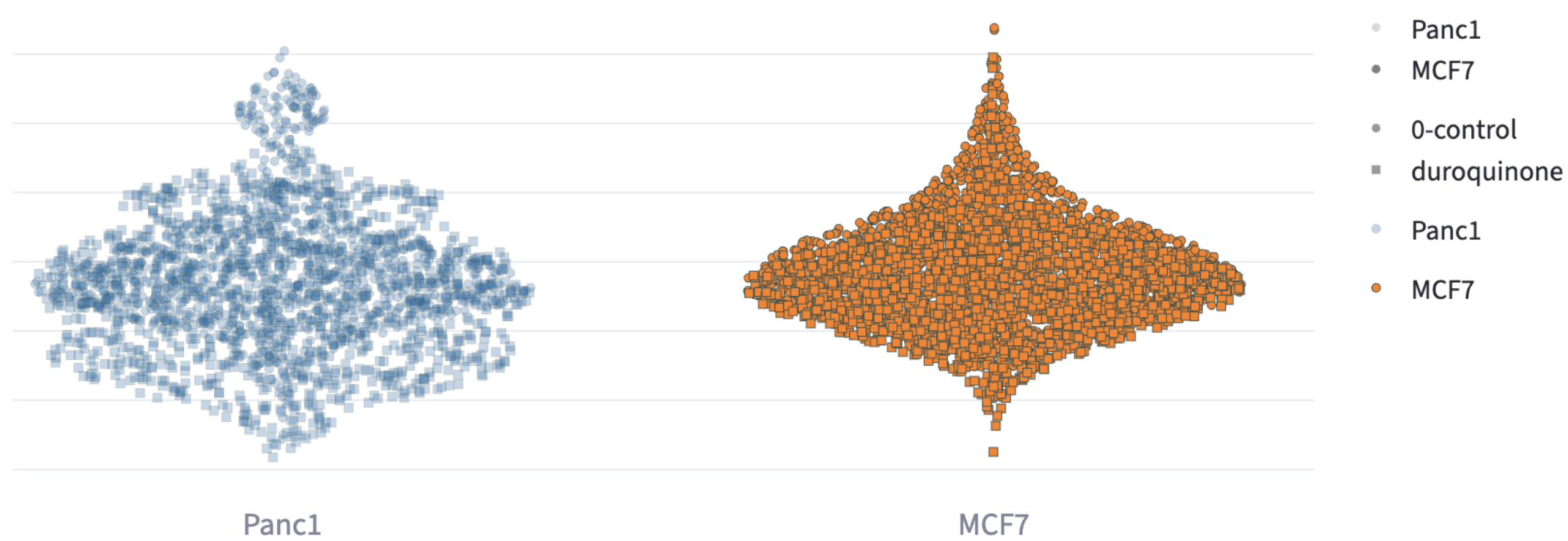

11.4.4 Unique ID Hover

- In all point-based visualizations, when hovering over a point, the unique id of the point will be shown.

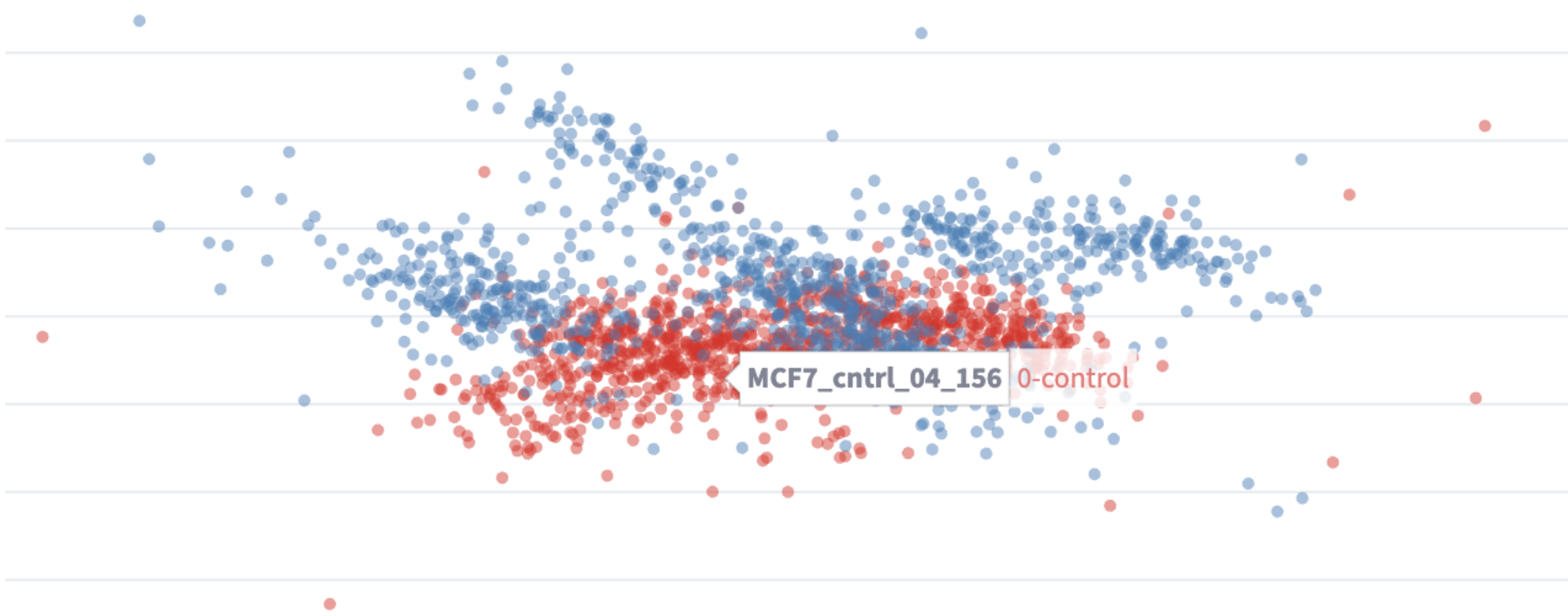

11.4.5 Plotting Configuration Widgets

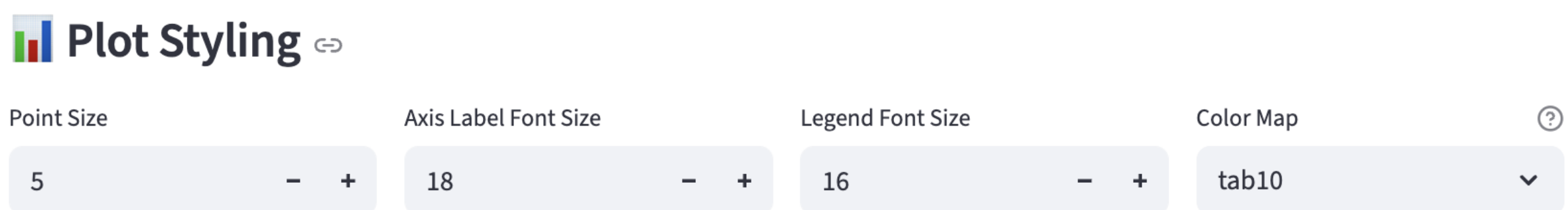

Users can interactively configure the following plot parameters:

- point size (if point-based)
- axis label size
- legend size
- the colormap used to color different groups
  - `colorblind`, `tab10`, `tab20`, `Set1`, `Set2`, `Set3`, `Pastel1`, `Pastel2`, `Accent`, `viridis`, `plasma`, `inferno`, `magma`, `cividis`

11.4.6 Export Workflow as Python Script

At the bottom right of the interface, users can export the entire analysis workflow as a self-contained Python script. This is useful when users want to share their analysis with others, reproduce their analysis later, customize the analysis further, or integrate it into their own workflow. The script will include all the code and analysis configuration (filters, selected numerical features, visual channels, plotting style, module-specific configurations, etc.). After exporting, users need to put the dataset in the same directory as the script and run the script to reproduce the analysis, where the plots are rendered as matplotlib figures and saved as **SVG** files for publication-ready quality.

12 Configuration

FLIM Playground provides an interactive configuration workflow to help users prepare their datasets that are not extracted by Data Extraction so that they fulfill the requirements.

The following sections use the iris dataset as an example: the `flower_id` is added to uniquely identify each row (i.e. a single flower). The other columns are: a categorical feature `species`, and a set of numerical features `sepal_length`, `sepal_width`, `petal_length`, and `petal_width`.

Specifically, the steps are:

- On the left side of the screen, uncheck `Use Dataset from Data Extraction`.

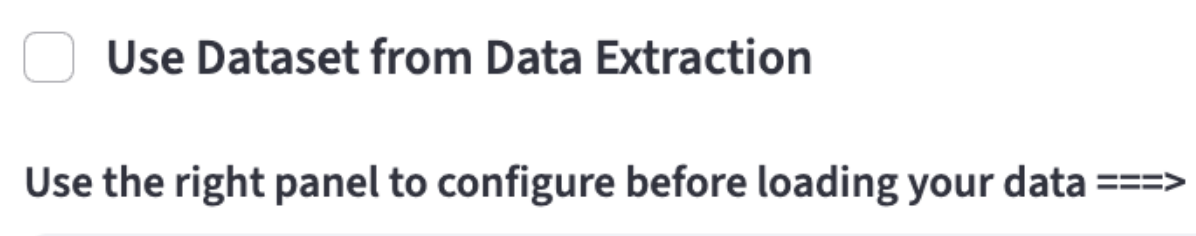

Then the configuration interface will be displayed.

12.1 Analysis Profiles

✨ **NEW** ✨: Users can now create, save, and switch between multiple analysis profiles—each a ready-to-go configuration tailored for different datasets.

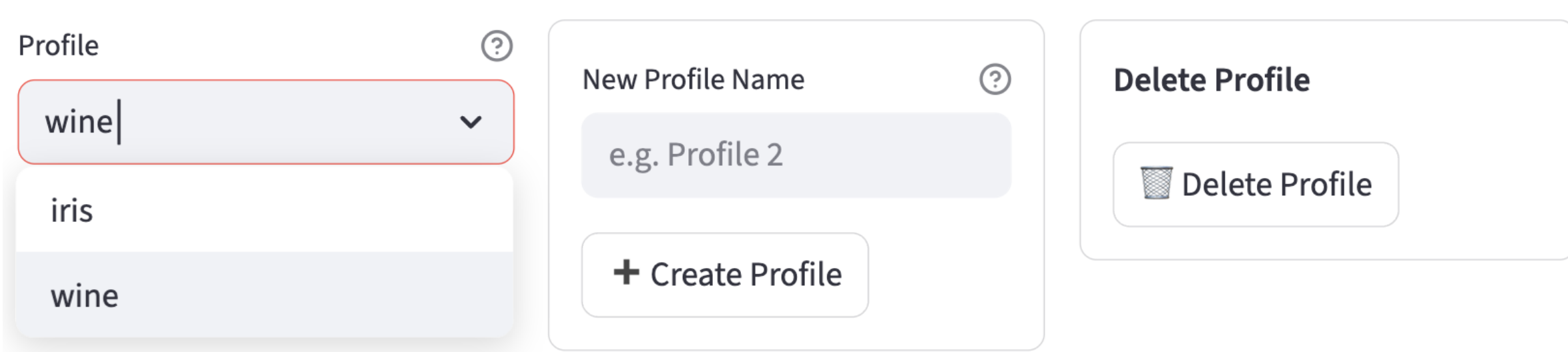

- On the left you can select an existing profile to load.
- In the middle you can create a new profile.
- On the right you can delete an existing profile.

Everything described below is pertained to the current profile.

12.2 Identifiers Config

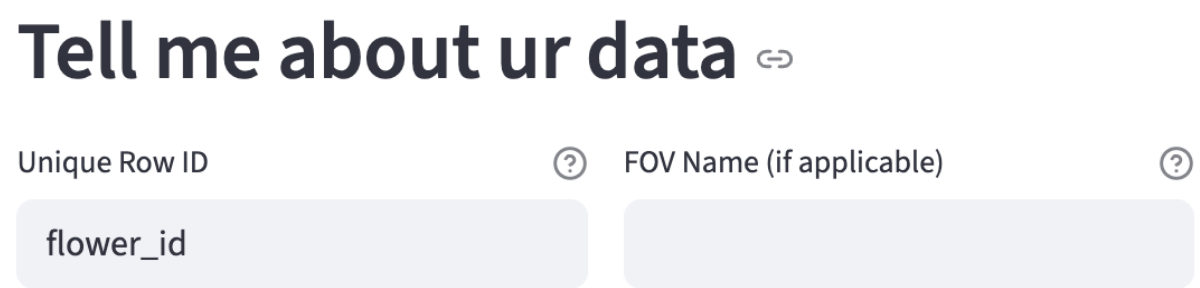

- The dataset needs to have a unique identifier column to identify each row. In this case, `flower_id` is the unique identifier column. This dataset does not have a field of view column, so we leave it blank.

12.3 Categorical Feature Config

- Add categorical columns you want FLIM Playground to recognize here. Those columns are converted to strings internally.

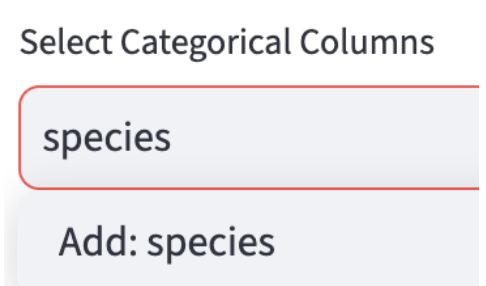

The default categorical feature list does not include `species`, so we add it manually.

12.4 Numerical Feature Config

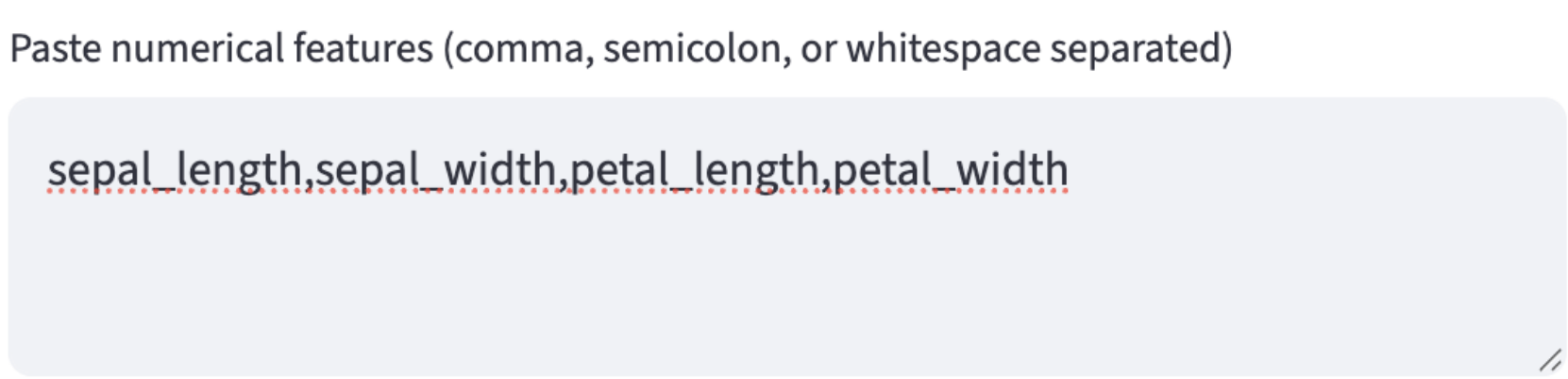

- Users can copy the numerical features from Excel or any text editor that opens their CSV files. The features can be separated by `,`, `;`, or whitespaces including newlines.
- After hitting the `ctrl/cmd + enter` key, the numerical features are parsed and added to `Available Features`. Users can add/delete groups and drag features from the `Available Features` to the groups they've just created. Any ungrouped features are added to a group called `Uncategorized Features`.

12.4.1 Example

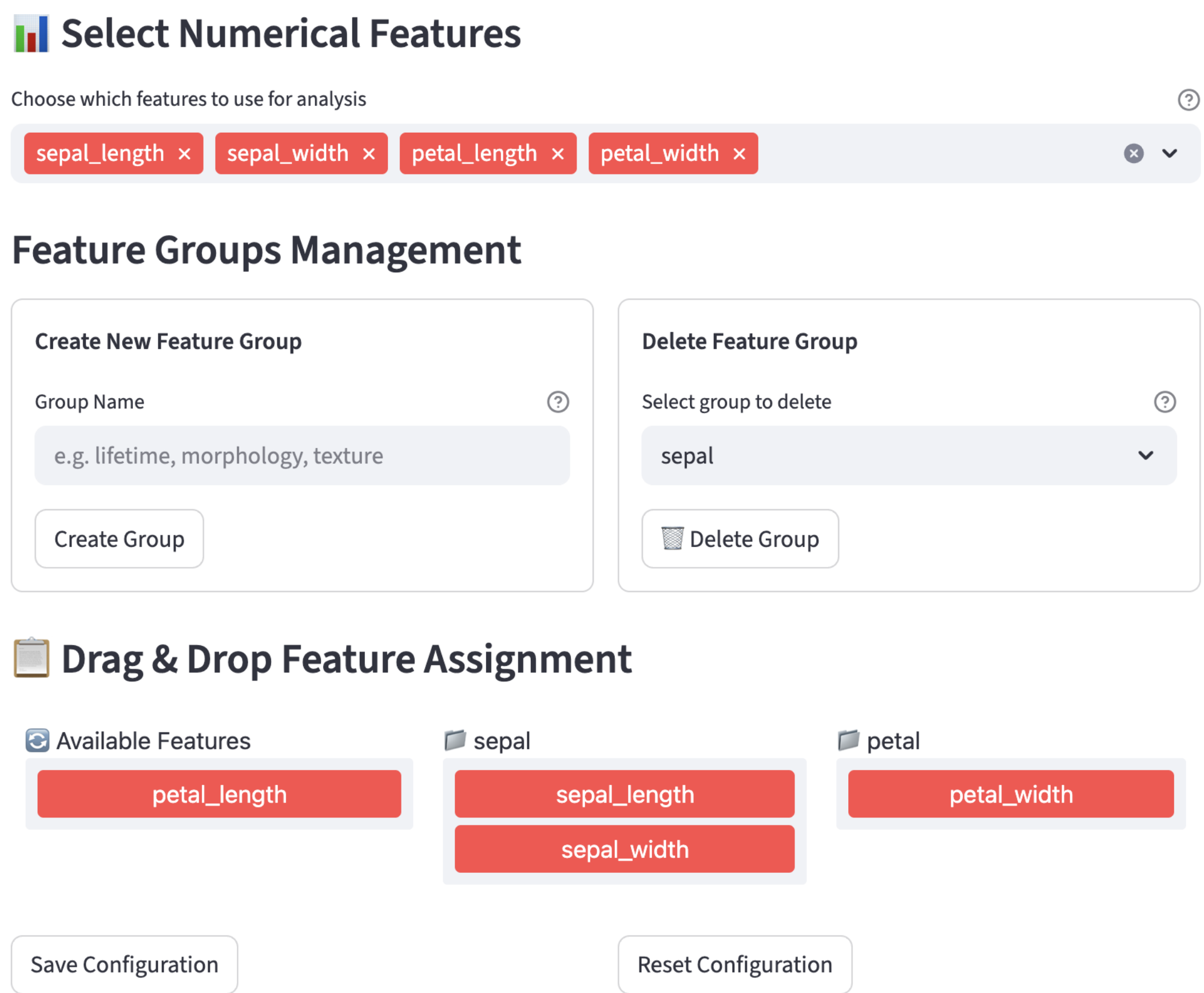

12.5 Save

- After users finish the configuration and click the `Save Configuration` button, FLIM Playground saves configurations into the current profile and loads it for processing current and future datasets.

12.6 Reset

Users can reset the current profile to the default configurations.

Part III

Univariate Analysis

13 Feature Comparison

This method allows users to compare the distributions of a single numerical feature across groups. It can help users spot overarching trends between distributions, identify within-distribution shape-related characteristics such as skewness or bimodality that might signal sub-populations, and diagnose potential outliers.

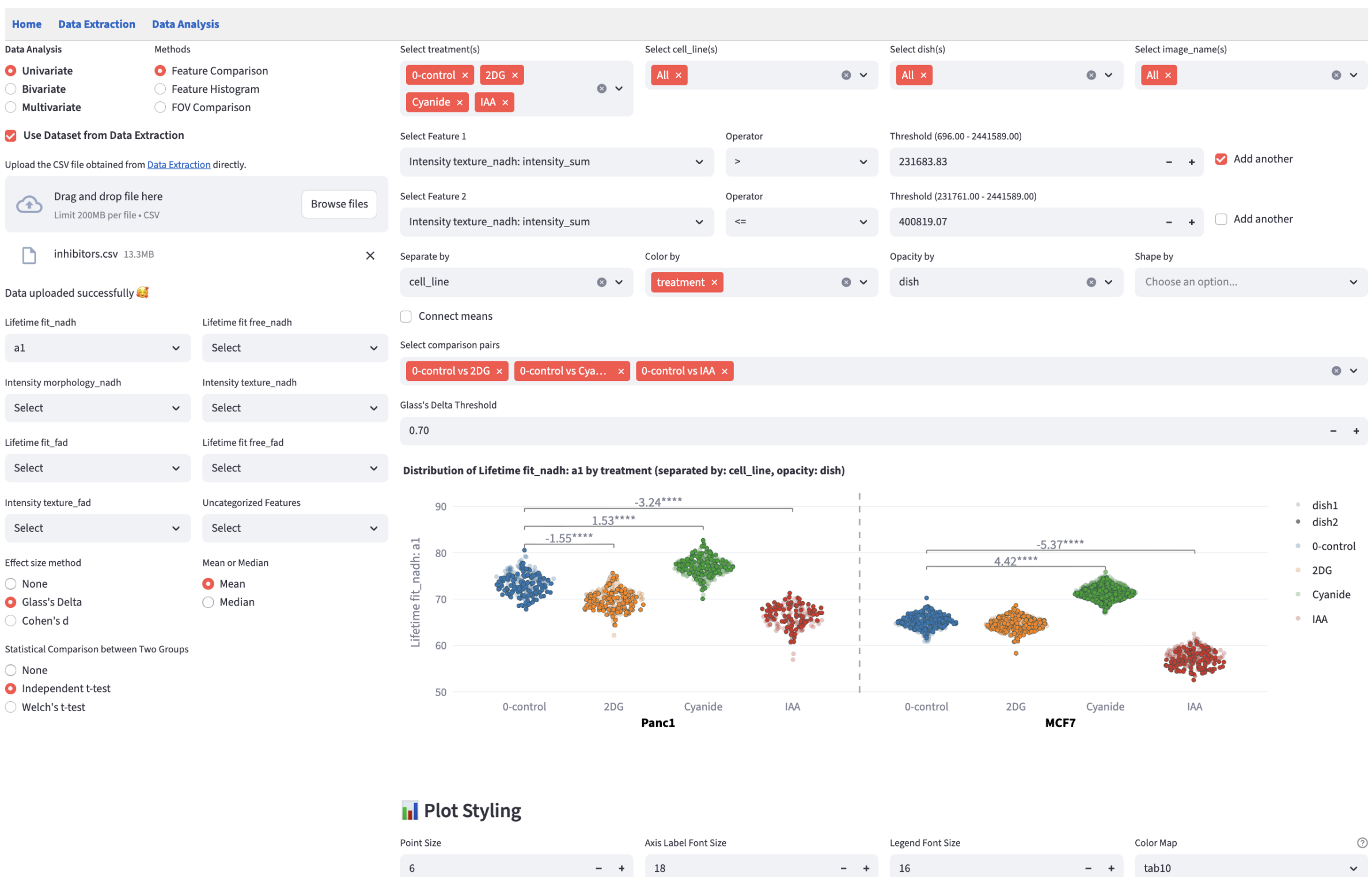

13.1 Shared Interface Components

- On the left, users can use the selection widgets to select the numerical feature to be compared. Exactly one feature can be selected from all feature groups.
- On the top right, users can use the filters to subset the data to find the groups of interest.
- Below the filters, users can apply the visual channels widgets that allow users to `Color by`, `Opacity by`, and `Shape by` categorical features.

- On the bottom right, users can change the plot style using the plot styling widgets.

13.2 Separate by

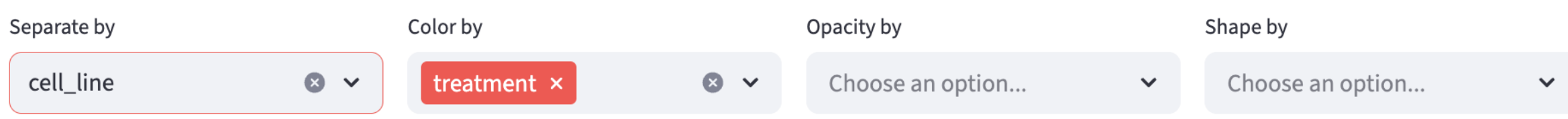

`Separate by` takes effect before `Color by`. `Separate by` divides the plot into sections separated by a dashed line, with each section labeled by one of the available categories of the selected categorical feature after filtering. The selected feature of `Separate by` is automatically removed from available features in `Color by`. The section order is determined by the order of the categories which are sorted numeric-alphabetically.

Then, visual channels (color, shape, opacity) and effect size annotation are applied within each section. For example, the visualization below was separated by `cell_line`, with each section labeled by the bold text of a cell line. Within each section, the x-axis rendered the `Color by` categories (`treatments`) so colors were consistent across sections. Effect size calculation and annotation (only effect size > 0.5 is shown) were performed within each section.

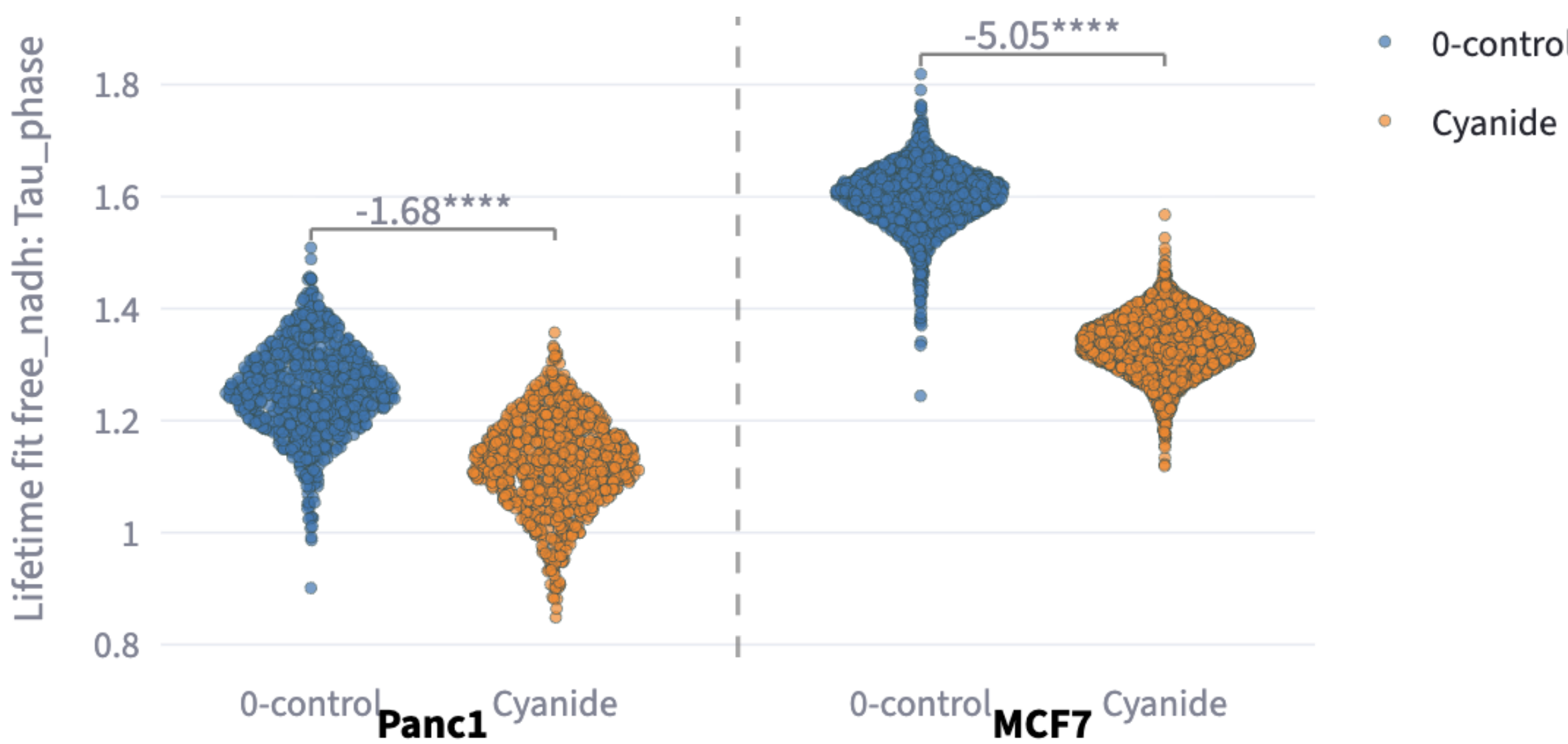

If `Separate by` is left blank and both `cell_line` and `treatment` are selected in `Color by`, then colors are assigned to each cell line and treatment combination, and effect sizes are calculated across all combinations of group pairs (only pairs with effect size > 0.5 are shown).

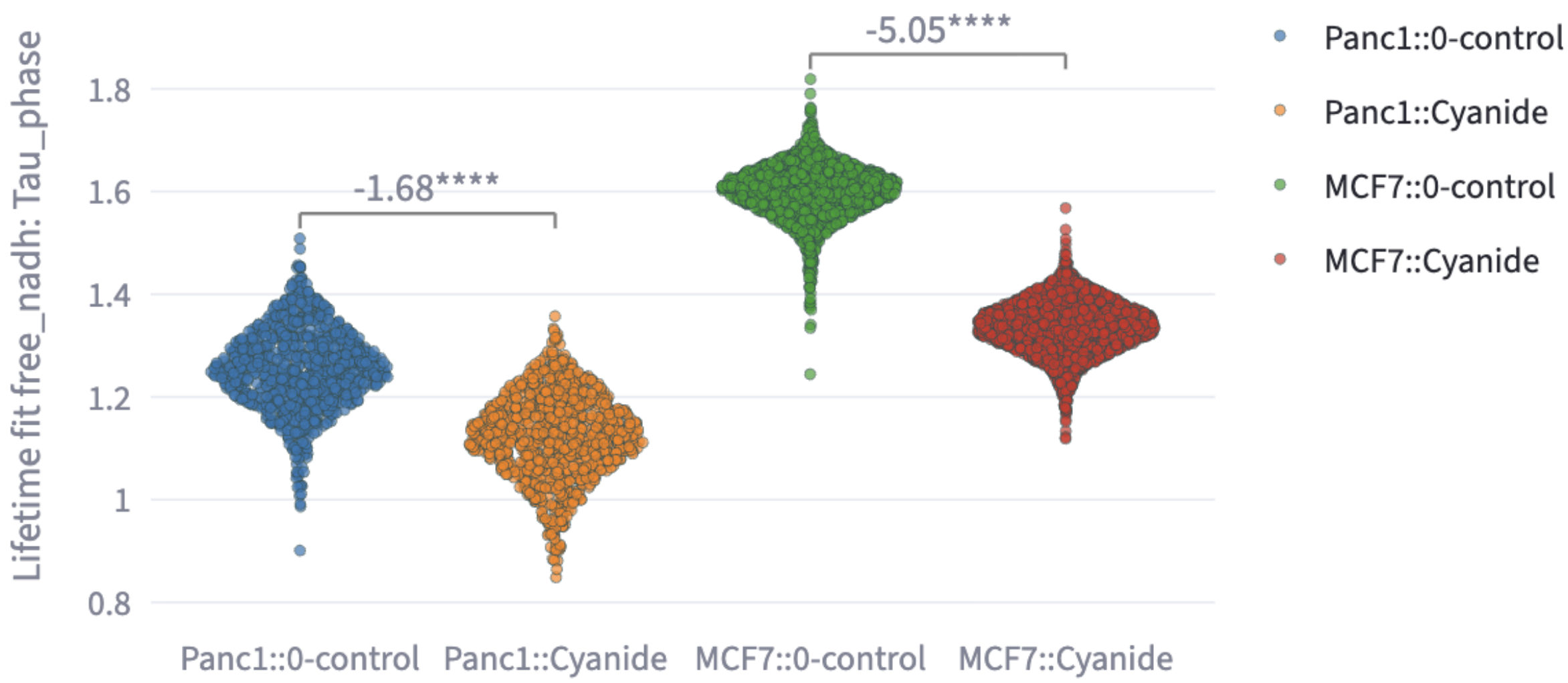

13.3 Comparison Widget

Statistical tests and effect size calculations are performed on the selected comparison pairs. FLIM Playground uses the groups on the x-axis populated by Color by to create comparison groups, starting from the leftmost group. For example, if there are 5 groups, then $\binom{5}{2} = 10$ comparisons will be created. The number of groups can get combinatorially large and many of the comparisons are not meaningful. Therefore, two widgets are provided to filter the comparison groups:

- A selection widget that shows all possible comparisons by default and users can deselect groups they do not need.

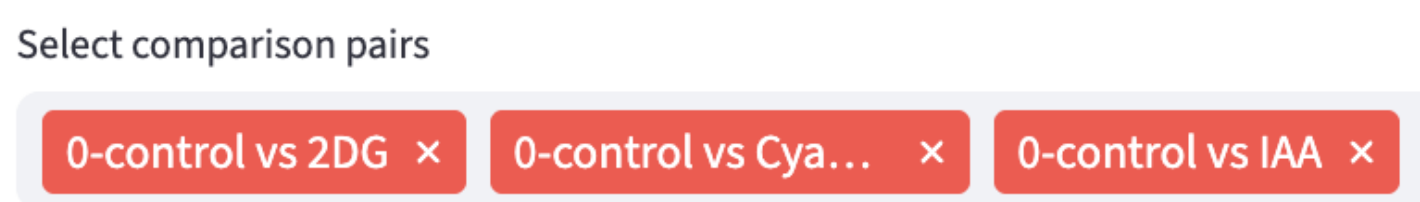

- A threshold by effect size widget that filters out comparisons below the threshold.

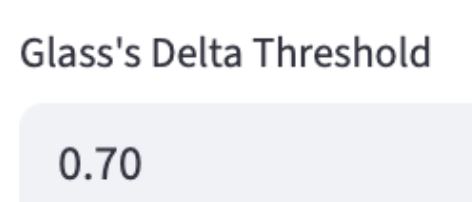

13.4 Effect Size

Complementary to statistical tests, which address "is there a difference?", effect size metrics answer "how large is the difference?", enabling cross-study comparison. FLIM Playground offers two effect size calculation methods, Glass's Delta and Absolute Cohen's d, each can be in mean or median form.

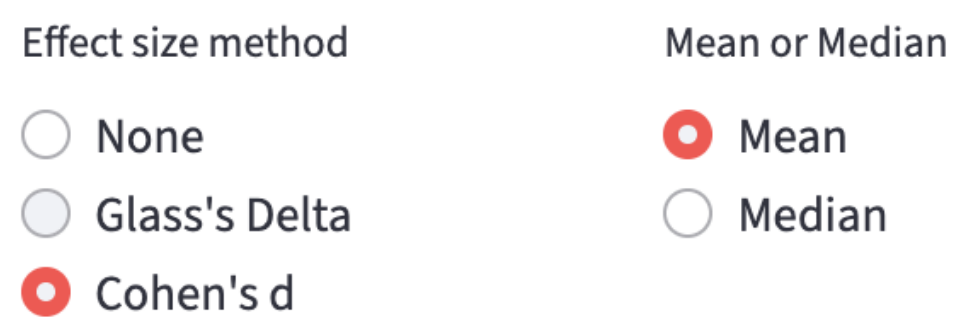

13.4.1 Glass's Delta

Mean ($\bar{X}$) form:

$$\Delta_G = \frac{\bar{X}_{\text{treatment}} - \bar{X}_{\text{control}}}{s_{\text{control}}}, \qquad s_{\text{control}} = \sqrt{\frac{1}{n_c - 1}\sum_{i=1}^{n_c}(X_{control_i} - \bar{X}_{\text{control}})^2}$$

Median ($\tilde{X}$) form:

$$\widetilde{\Delta}_G = \frac{\tilde{X}_{\text{treatment}} - \tilde{X}_{\text{control}}}{\text{MAD}_{\text{control}}}, \qquad \text{MAD}_{\text{control}} = 1.4826 \times \text{median}\left|X_{control_i} - \tilde{X}_{\text{control}}\right|, \qquad i = 1, \dots, n_c$$

Note

MAD stands for median absolute deviation. In order to make MAD asymptotically consistent with the standard deviation under normality, it is scaled by $C = 1/\Phi^{-1}(0.75) \approx 1.4826$. Implementation-wise, it uses `median_abs_deviation` from `scipy.stats`, and `scale` option is set to `"normal"` to account for the constant multiplier C. THE MEDIAN FORM of both Glass's Delta and Absolute Cohen's d IS NOT REPORTED IN THE LITERATURE.

Important

Which group is treatment and which is control matters! From the formula of Δ_G, switching the order not only flips the sign of the result, but also changes the denominator, which is s_{control}, the standard deviation of the *control*. Currently, FLIM Playground treats the group on the left as control and the group on the right as treatment (the negative Δ_G below implies this). FLIM Playground now supports user-customizable x-axis order so that users can arrange the groups to make sure the control group is on the left. Or users can use Absolute Cohen's d that does not

assume the treatment-control structure.

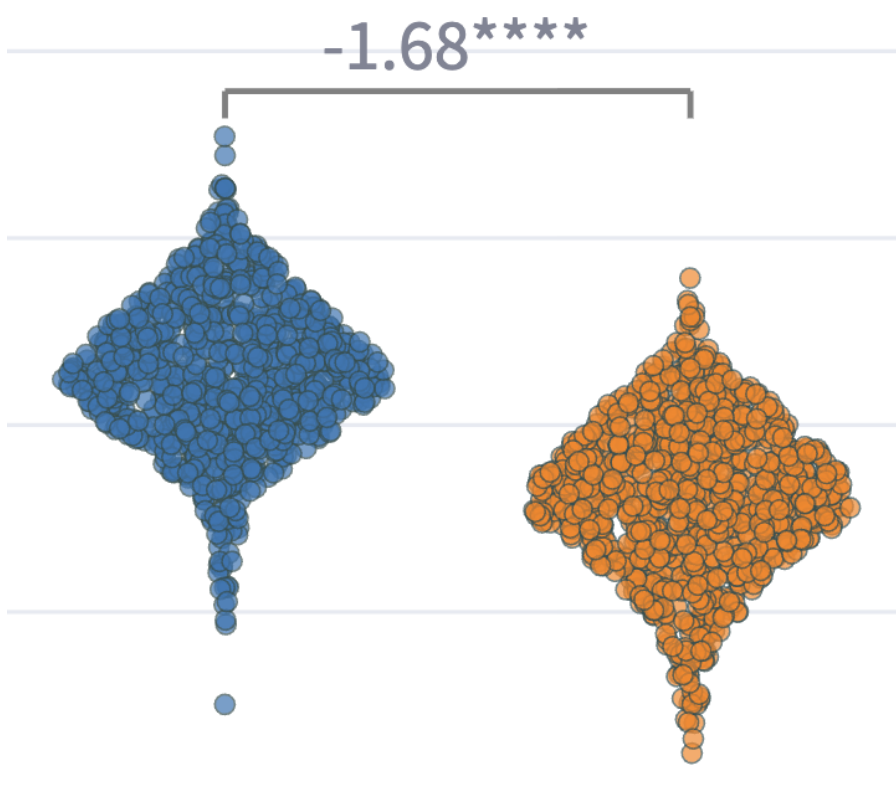

ℹ Note

To keep the point-based visualization style that supports interactive hover while showing the violin-plot-like distribution, Sina plot is used: it uses `gaussian_kde` from `scikit-learn` to make sure the width of the point distribution is proportional to the kernel density.

13.4.2 Absolute Cohen's d

Mean ($\bar{X}$) form:

$$|d| = \left|\frac{\bar{X}_1 - \bar{X}_2}{s_p}\right|, \qquad s_p = \sqrt{\frac{(n_1-1)s_1^2 + (n_2-1)s_2^2}{n_1+n_2-2}}$$

Median ($\tilde{X}$) form:

$$|\tilde{d}| = \left|\frac{\tilde{X}_1 - \tilde{X}_2}{s_{\tilde{p}}}\right|, \qquad s_{\tilde{p}} = \sqrt{\frac{(n_1-1)\,\mathrm{MAD}_1^2 + (n_2-1)\,\mathrm{MAD}_2^2}{n_1+n_2-2}}$$

13.5 Statistical Test

Statistical tests are performed on the selected comparison pairs that exceed the effect size threshold (if users choose to calculate effect sizes). The statistical tests supported are:

- Independent t-test (student's t-test, for equal variances)

- Welch's t-test (for unequal variances)

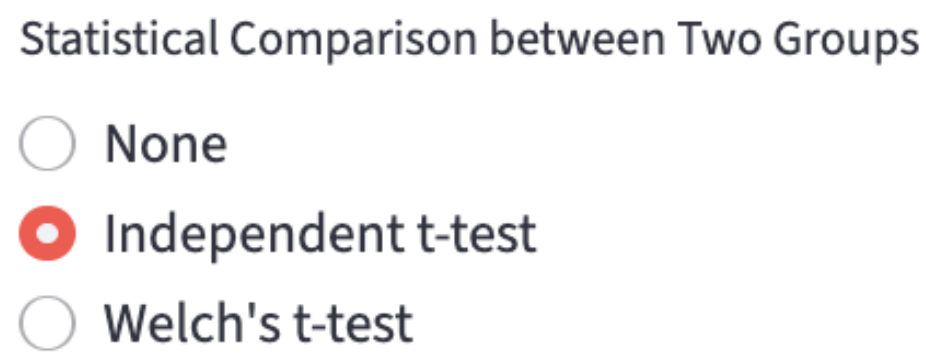

FLIM Playground uses `scipy.stats.ttest_ind` to perform the independent t-test and `scipy.stats.ttest_ind` with `equal_var=False` to perform the Welch's t-test.

P-values are summarized with significance asterisks: if $p \leq 0.0001$, four asterisks (****) are shown; otherwise if $p \leq 0.001$, three (***); otherwise if $p \leq 0.01$, two (**); otherwise if $p \leq 0.05$, one (*). Values with $p > 0.05$ are not given a significance asterisk marker in this convention.

13.6 Comparison Annotation

For each selected comparison pair that exceeds the effect size threshold (if any), the statistical significance, effect size, or both are annotated on the plot. It selects the lowest eligible position (over all points in both groups and over existing annotations) to annotate. If both statistical significance and effect size calculation are performed, the annotation is shown in the format of effect size followed by p-value. If only one is performed, then only the performed method's result is shown.

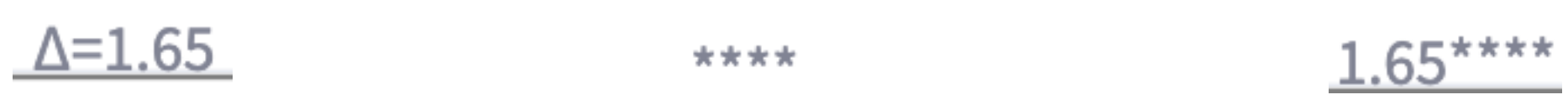

14 Feature Histogram

Feature histograms give an instant readout of each group's center, spread, skew and any multi-peaked shape that raw tables hide. FLIM Playground plots a histogram for each group based on the selected numerical feature. Additionally, the Gaussian Mixture Model mode can help find subcomponents of each histogram in an unsupervised way. To quantify the distribution heterogeneity, normalized H-index is calculated for each group.

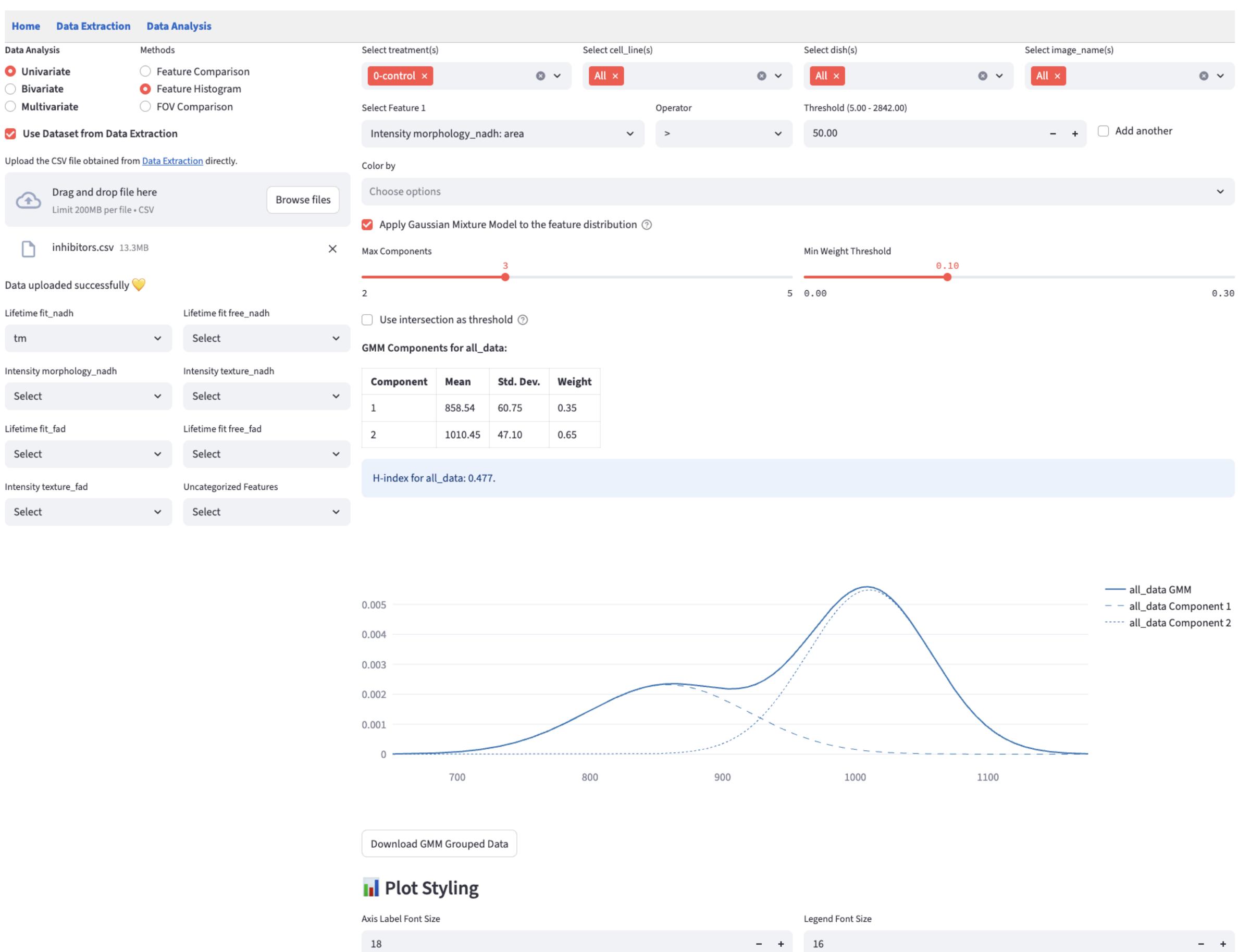

14.1 Shared Interface Components

- On the left, users can use the selection widgets to select the numerical feature to see the histogram/GMM distribution. Exactly one feature can be selected from all feature groups.
- On the top right, users can use the filters to subset the data to find the groups of interest.
- Below the filters, users can apply the visual channels widgets that allow users to `Color by` categorical features. `Opacity by` and `Shape by` are *not* supported because there are no points in the histogram/GMM plot.
- On the bottom right, users can change the plot style using the plot styling widgets.

14.2 Histogram Mode

With a single click, users can switch between the histogram mode and the Gaussian Mixture Model mode. When the checkbox is unchecked, the histogram mode is selected.

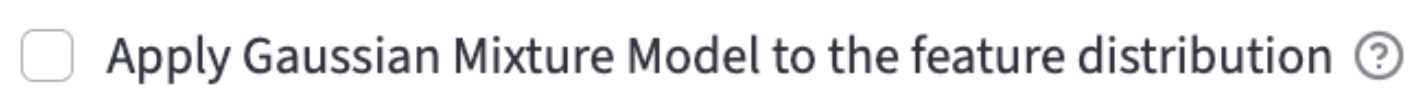

Histogram mode plots a histogram of the selected numerical feature for each group. The histogram bin width is user-controllable, with the default bin width determined automatically as the minimum bin width between the 'sturges' and 'fd' estimators (see here for more details). The maximum bin width is set to one-third of the range of the data. The step size is set to $1/50$ of the range.

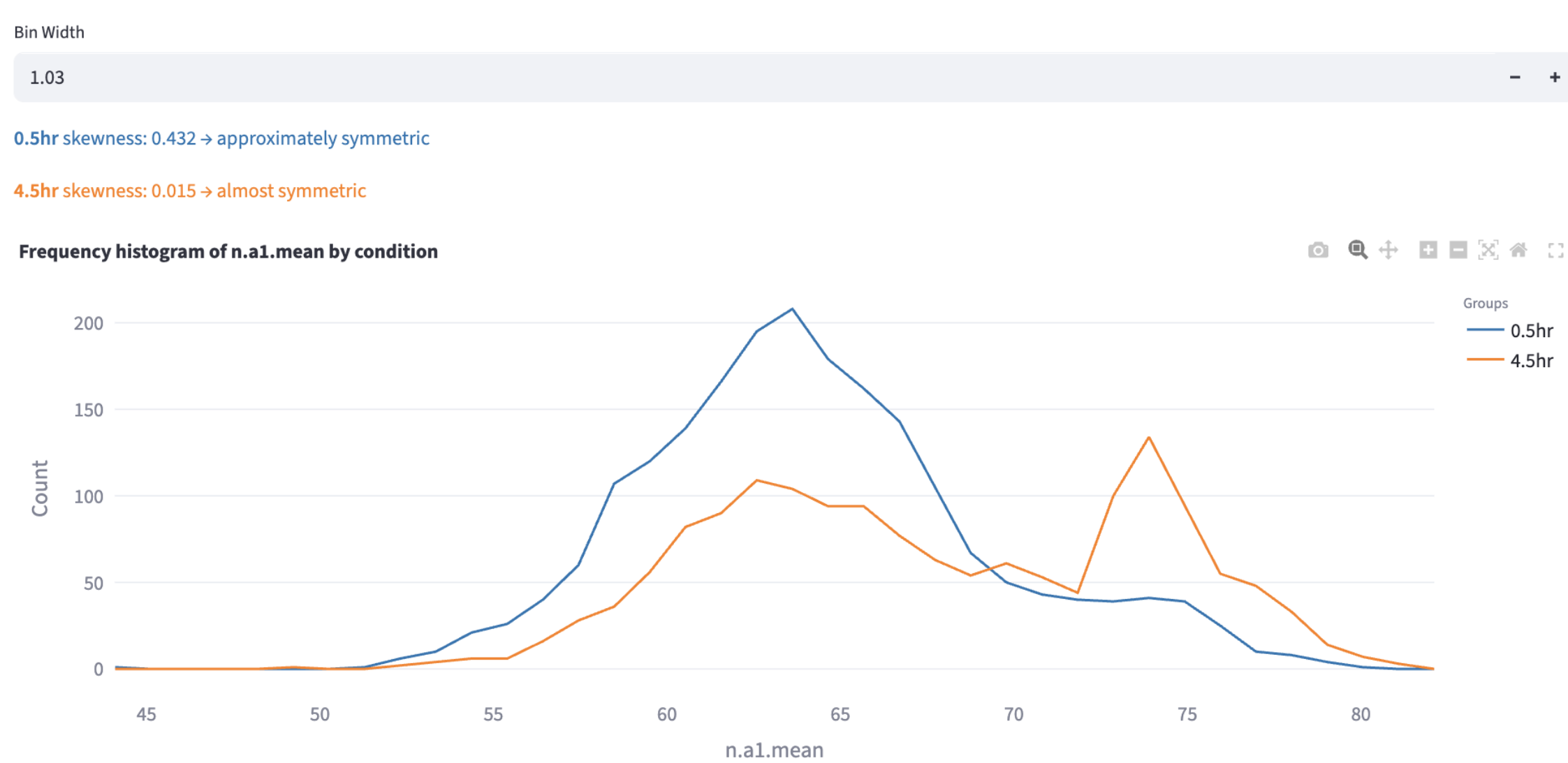

Additionally, the skewness of the distribution is computed and displayed[13].

A rule of thumb categorization of skewness is as follows:

- Strongly left-skewed: < -1
- Moderately left-skewed: -1 to -0.5
- Approximately symmetric: -0.5 to -0.25 and 0.25 to 0.5
- Almost symmetric: -0.25 to 0.25
- Moderately right-skewed: 0.5 to 1
- Strongly right-skewed: > 1

However, the skewness is just one way to quantify the distribution shape. In the example above, the orange distribution was clearly bimodal, but the skewness alone could not capture this. In applications where the distribution is multi-modal, and each mode can represent certain subpopulations (e.g. a cell type, a cell state, a cell cycle phase, etc.), users can use the Gaussian Mixture Model mode to find subcomponents of each distribution in an unsupervised way.

14.3 Gaussian Mixture Model Mode

GMM Components for 0.5hr:

Component	Mean	Std. Dev.	Weight
1	62.18	3.51	0.68
2	68.67	4.66	0.32

GMM Components for 4.5hr:

Component	Mean	Std. Dev.	Weight
1	63.37	3.58	0.59
2	73.84	2.68	0.41

H-index for 0.5hr: 0.468. H-index for 4.5hr: 0.485.

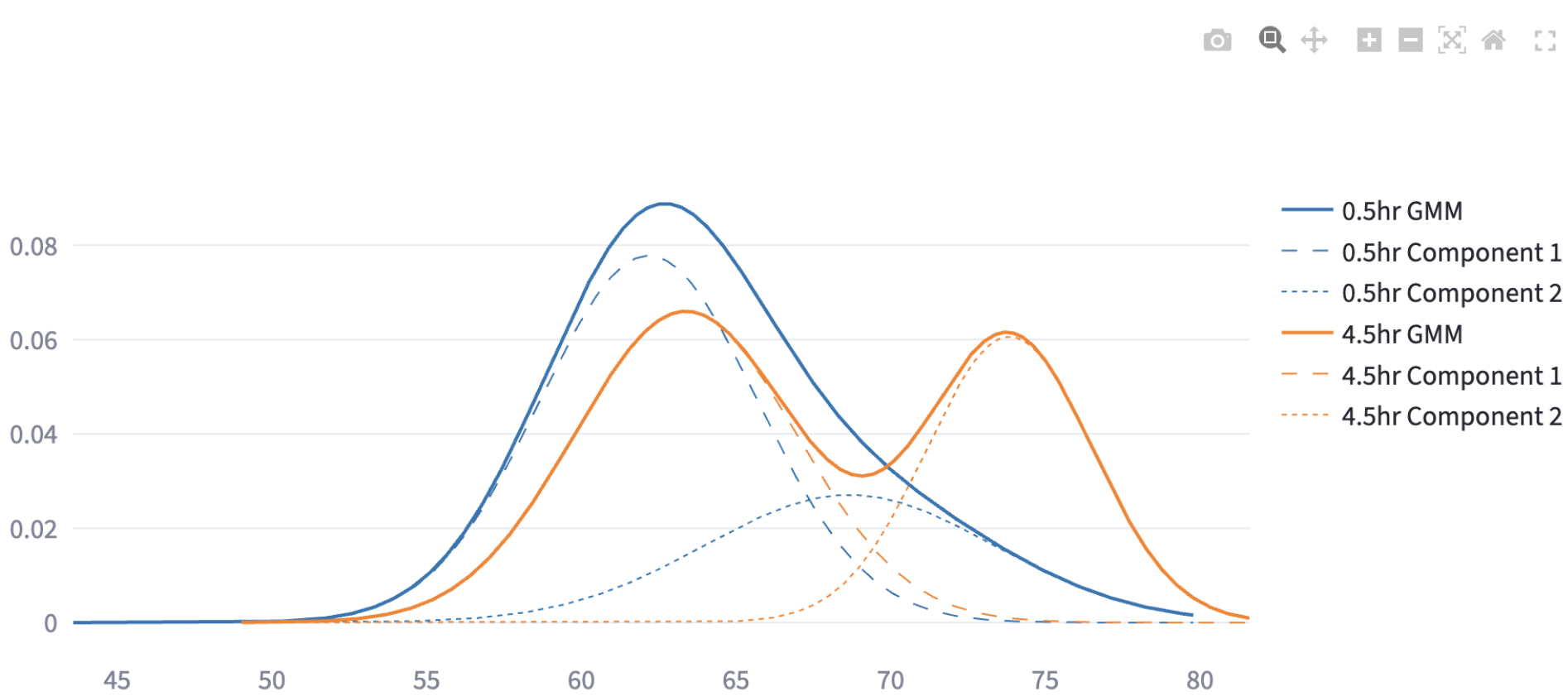

A Gaussian Mixture Model (GMM) represents a dataset as a weighted sum of multiple Gaussian (normal) distributions, each defined by its own mean (μ) and (co)variance (σ^2). By estimating the component

parameters and their weights (mixing coefficients) from the data—commonly using the Expectation-Maximization (EM) algorithm—GMMs can capture complex, multimodal distributions that a single Gaussian cannot.

14.3.1 Fit Gaussian Mixture Models

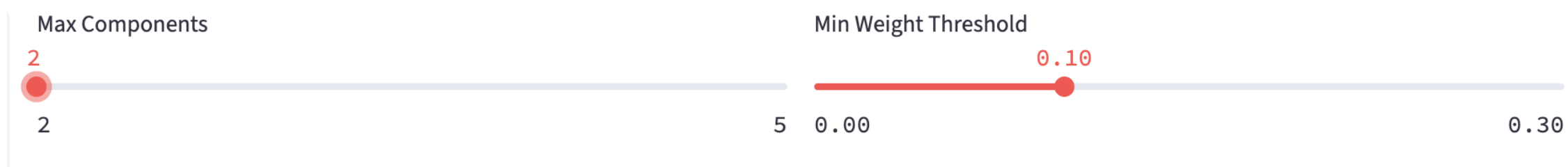

Figure 14.1: Gaussian Mixture Model Configuration

- Fits GMMs on the selected numerical feature up to `Max Components` components, each of which has its component weights (w_i), means (μ_i) and variances (σ_i^2).
- Retains only *valid* models in which every component has weight at least `Min Weight Threshold` (the weights of all components add to 1).
- FLIM Playground will choose the valid model with the lowest BIC score that penalizes the model complexity to avoid overfitting.

14.3.2 Heterogeneity index

To quantify the subpopulation structure, based on the GMM fit, FLIM Playground computes an "H□index" for each group as a weighted entropy□distance: each component contributes according to its weight's uncertainty $(-w_i \log w_i)$[14] scaled by how far its mean μ_i sits from the overall mixture mean $\bar{\mu} = \sum_{i=1}^{k} w_i \, \mu_i$, normalized by the standard deviation of μ_is.

$$H = \sum_{i=1}^{k} (-w_i \log w_i) \, d_i, \qquad d_i \;=\; \frac{|\mu_i - \bar{\mu}|}{\sigma}$$

14.3.3 Classification

- Users can choose to perform intersection thresholding or hard assignment to determine which GMM component each data point belongs to. It appends a new categorical feature column called `GMM_Group` (recognizable by methods in Data Analysis as a categorical feature) to the filtered dataset, the values of which are `{color_group}_1`, `{color_group}_2`, etc. In the example above, the color groups were created by 0.5hr and 4.5hr because the user colored the data by `hour`. The classification result can be saved by clicking:

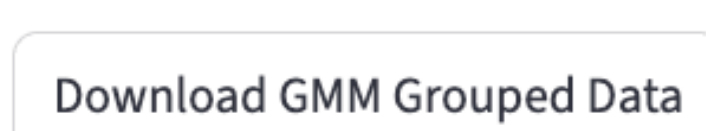

14.3.3.1 Intersection thresholding

- Once the GMM is estimated, thresholds can be obtained by locating the intersection points of adjacent component densities:

$$w_1 \, \mathcal{N}(x; \mu_1, \sigma_1^2) \;=\; w_2 \, \mathcal{N}(x; \mu_2, \sigma_2^2)$$

  where w_1, μ_1, σ_1 are the mixture weight, mean, and standard deviation of the first Gaussian component, respectively, and w_2, μ_2, σ_2 those of the second component.
- The above equation can be formed as a quadratic equation and solved for the unique root lying between μ_1 and μ_2. In practice, we bracket this interval and apply a robust one□dimensional root-finding routine (e.g. Brent's method) to compute intersection points. Data points are then assigned to the class corresponding to the interval in which they fall.

> **! Important**
>
> In rare cases when there is more than one intersection point, the data point is assigned using hard assignment rather than intersection thresholding.

14.3.3.2 Hard assignment

- For each data point, it computes the posterior probability (responsibility) of each component, and assigns the data point to the component with the highest responsibility.

$$\text{label}(x) = \arg\max_i \gamma_i(x)$$

$$\text{where} \quad \gamma_i(x) = \frac{w_i \, \mathcal{N}(x \mid \mu_i, \sigma_i^2)}{\sum_{j=1}^{K} w_j \, \mathcal{N}(x \mid \mu_j, \sigma_j^2)}$$

14.3.4 Example

Pham et al.[15] used GMM with hard thresholding to create gates using NAD(P)H a_1 and optical redox ratio. These gates were used to define metabolically stressed and fit cells in their study of the impact of cryopreservation on T cell metabolism.

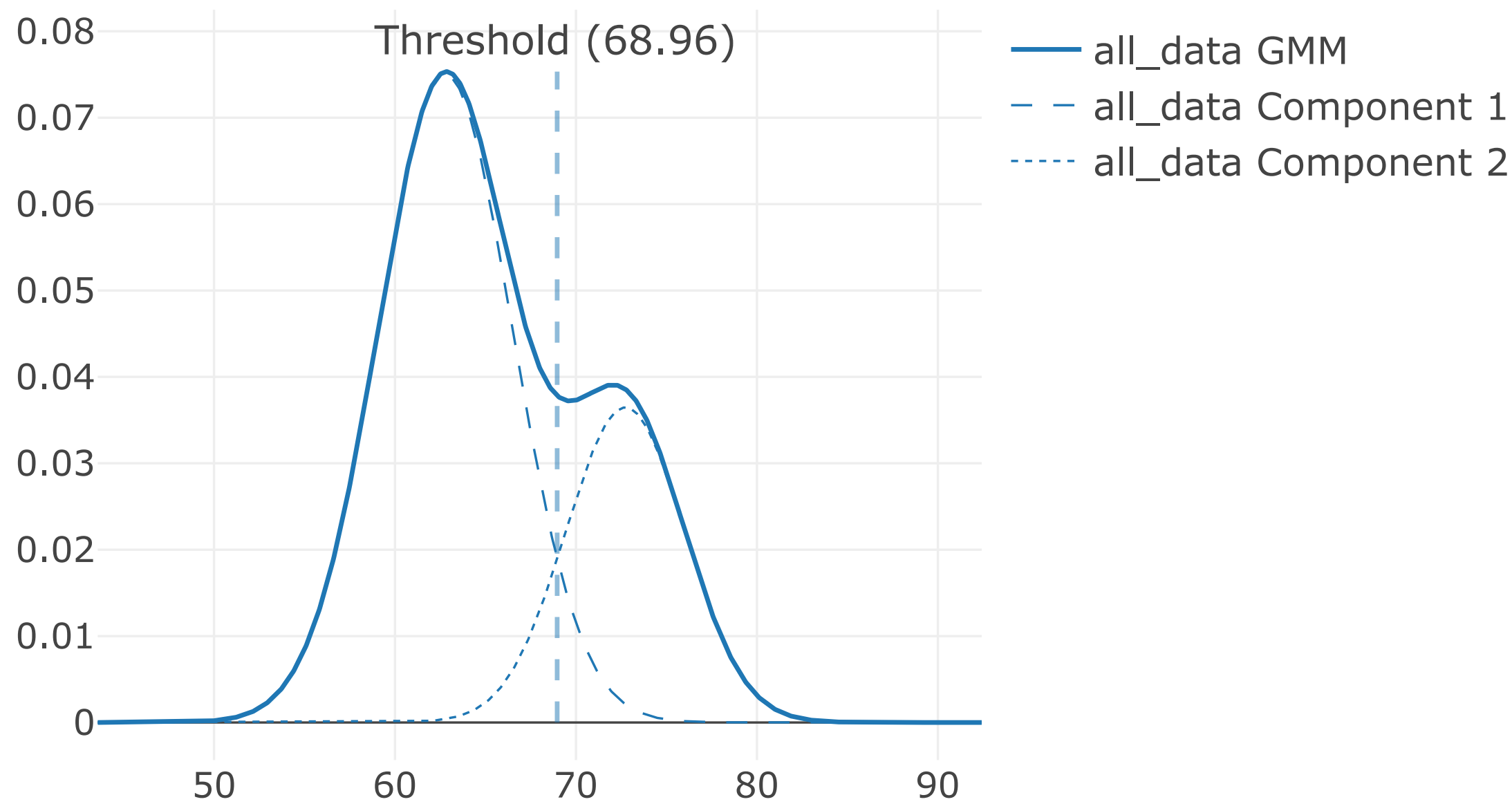

Threshold (68.96)
0.08
0.07
0.06
0.05
0.04
0.03
0.02
0.01
0
50
60
70
80
90
all_data GMM
all_data Component 1
all_data Component 2

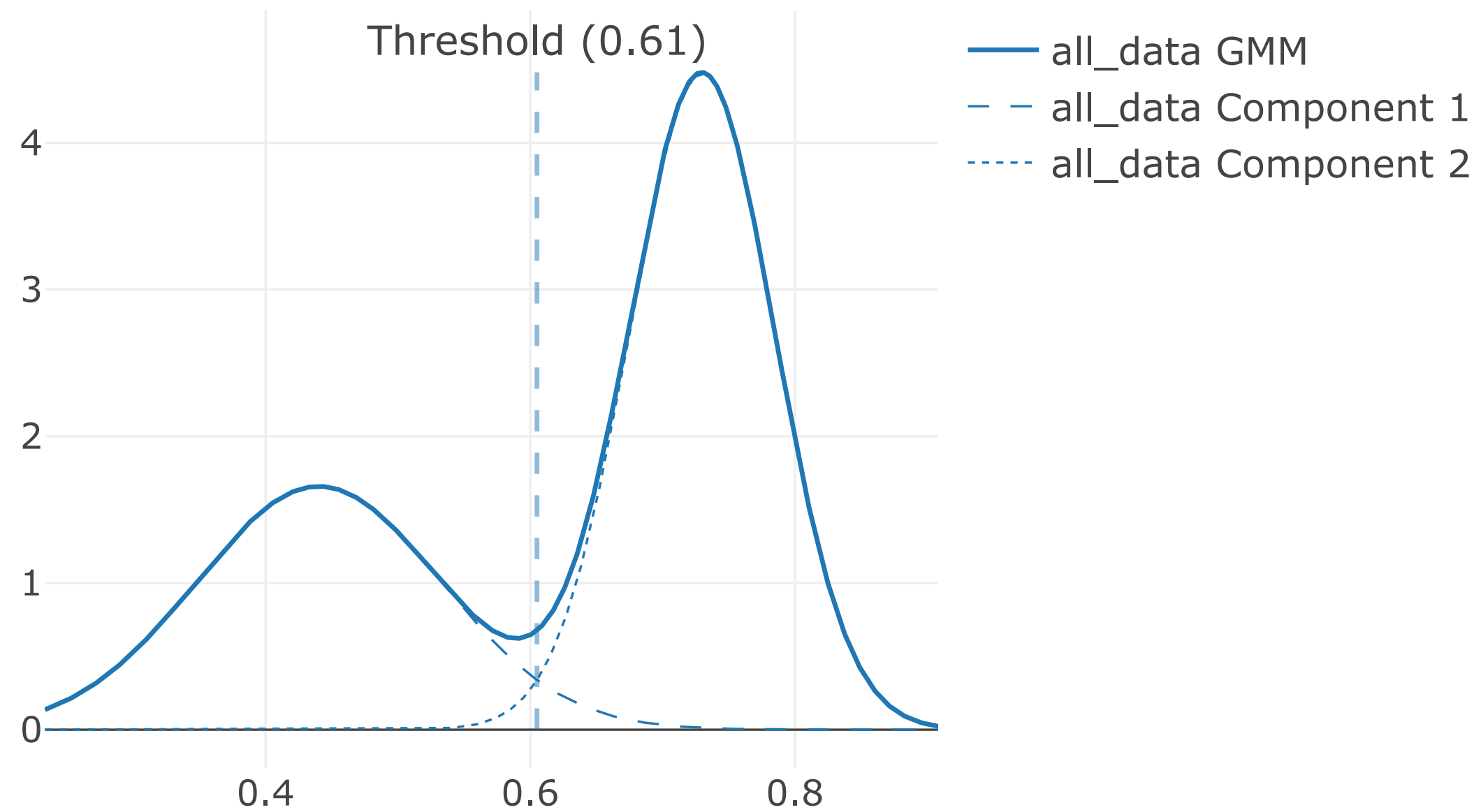

They compared the GMM-derived gate with cell viability by Trypan blue staining:

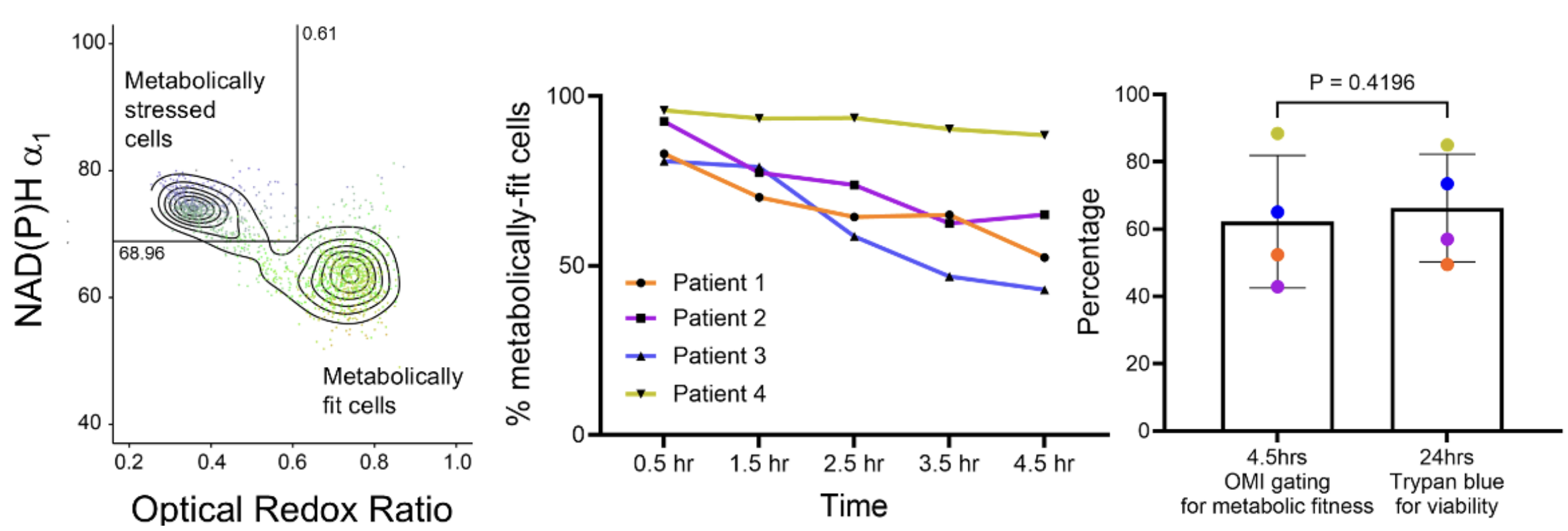

It suggests that optical metabolic imaging (OMI) could serve as an early, non-destructive indicator of T cell viability post-thaw.

Part IV

Bivariate Analysis

15 Feature Distribution

This method extends the univariate Feature Histogram method to visualize the distribution of two numerical features on a scatter plot. It helps reveal patterns such as correlations, clusters, trends, or outliers.

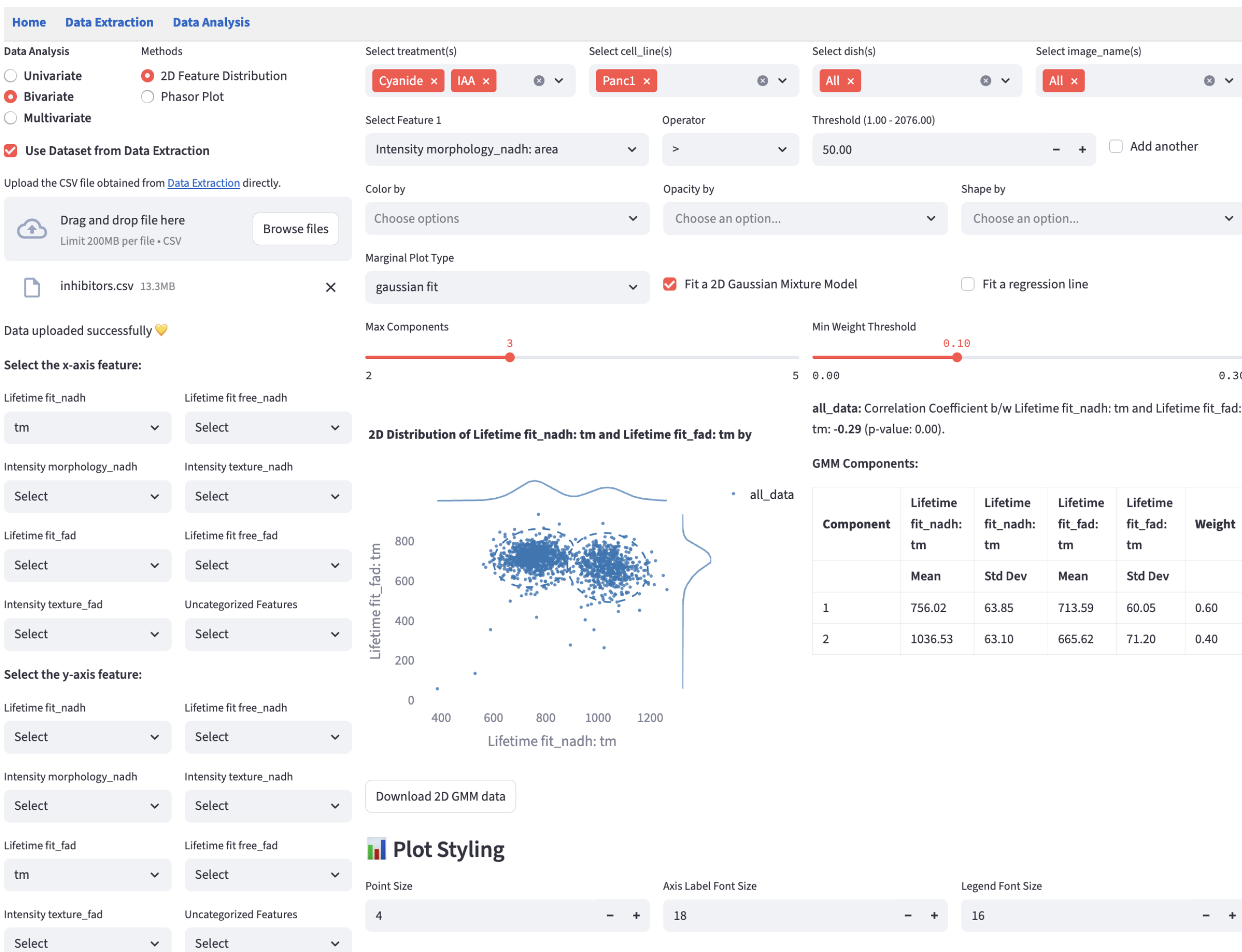

15.1 Shared Interface Components

- On the left, users can use the selection widgets to select the two numerical features on the x-axis and y-axis.
- On the top right, users can use the filters to subset the data to find the groups of interest.
- Below the filters, users can apply the visual channels widgets that allow users to `Color by`, `Opacity by`, and `Shape by` categorical features.
- On the bottom right, users can change the plot style using the plot styling widgets.

15.2 Marginal Distribution

Marginal distributions (the distribution of a single feature) are plotted at the top and the right of the scatter plot. Users can choose the plot type from the dropdown menu.

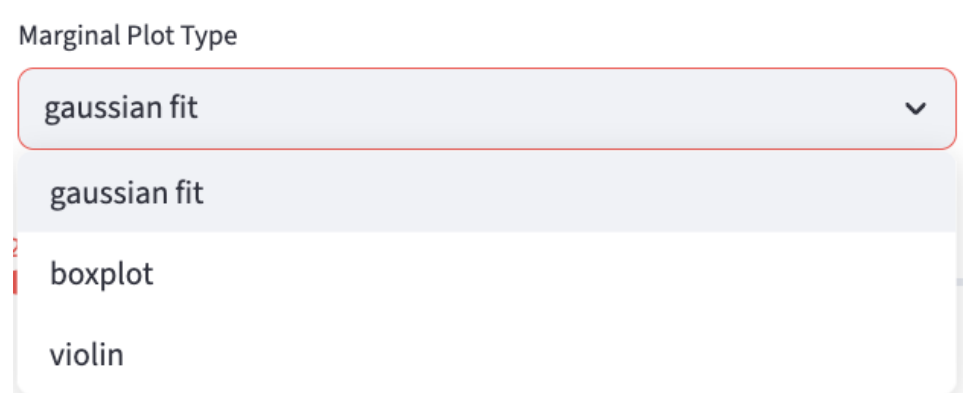

`gaussian fit`: provides a kernel-density estimate using Gaussian kernels. It uses `gaussian_kde` from `scipy.stats` and sets all parameters to default values.

15.3 2D Gaussian Mixture Model

Using the same method as fitting a 1D Gaussian Mixture Model, users can fit a 2D GMM by specifying `Max Components` and `Min Weight Threshold` for each color group.

Ellipses that capture approximately 95% of the points in each subcomponent are drawn. The color of the ellipses is determined by the color group that the subcomponent belongs to.

> **i How ellipses are drawn**
>
> Each ellipse represents a subcomponent of the GMM.
>
> - Center of the ellipse is the mean of the subcomponent (μ_x, μ_y).
> - The ellipse is rotated by $\theta = \arctan 2\big(v_{1y}, v_{1x}\big)$, where $\mathbf{v}_1 = \begin{bmatrix} v_{1x} \\ v_{1y} \end{bmatrix}$ is the first eigenvector of the covariance matrix, so that the major axis of the ellipse is aligned with the eigenvector

corresponding to the largest eigenvalue.

- The semi-axis lengths are $r\sqrt{\lambda_1}$ and $r\sqrt{\lambda_2}$, where λ_1 and λ_2 are the covariance eigenvalues (variances along the principal axes) scaled by $r = \sqrt{\chi^2_{\mathrm{df}=2,\,p=0.95}}$ (Mahalanobis distance) that captures approximately 95% of the points in the subcomponent.

All the components below will be rendered for each color group, which is created by user-chosen categorical features in `Color by`.

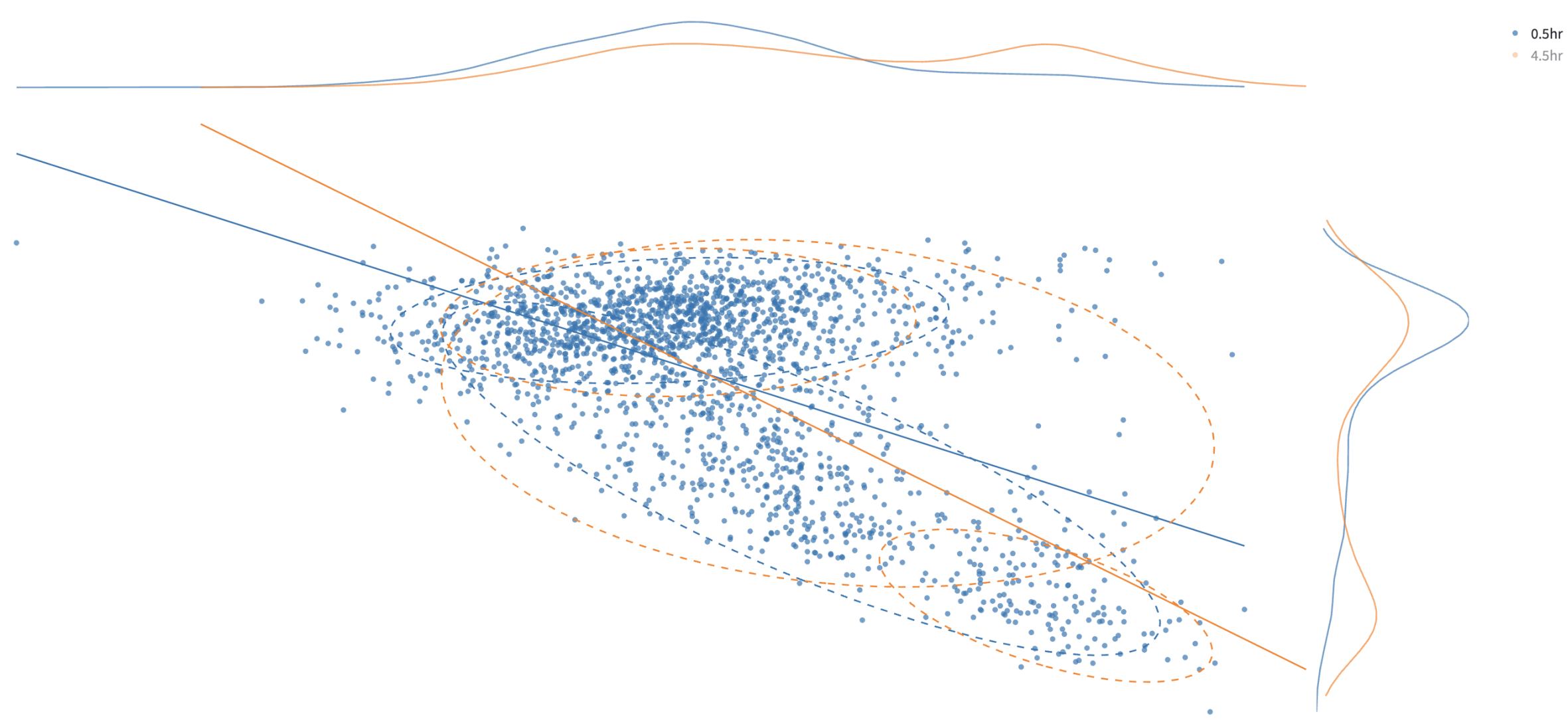

> **Tip**
>
> Users can click on a legend group to hide/show the data points in that group. In the above example, to avoid cluttering the plot, the orange group is hidden.

Tables of subcomponents' means, standard deviations, and weights are reported on the side.

0.5hr: Correlation Coefficient b/w n.a1.mean and rr.mean: **-0.56** (p-value: 0.00).

GMM Components:

Component	n.a1.mean Mean	n.a1.mean Std Dev	rr.mean Mean	rr.mean Std Dev	Weight
1	66.71	5.29	0.54	0.11	0.36
2	62.83	4.12	0.74	0.04	0.64

4.5hr: Correlation Coefficient b/w n.a1.mean and rr.mean: **-0.76** (p-value: 0.00).

GMM Components:

Component	n.a1.mean Mean	n.a1.mean Std Dev	rr.mean Mean	rr.mean Std Dev	Weight
1	73.43	3.12	0.39	0.07	0.39
2	63.87	4.33	0.72	0.07	0.61

The classification result can be saved by clicking:

Download 2D GMM data

The classification is done by hard assignment to determine which GMM component each data point belongs to. It appends a new categorical feature column called `2D_GMM_group` to the filtered dataset, the values of which are `{color_group}_group1`, `{color_group}_group2`, etc. The downloaded dataset keeps all the features plus the new categorical feature column, which is recognized by any method in Data Analysis as a categorical feature like others. The rows that pass the filters are included.

15.4 Regression line

A linear regression line is fitted to the data points in each color group. The key statistics are reported on the side.

Regression R²: 0.311 (Slope: -0.014, Intercept: 1.570)

R^2 tells how much of the variability in y is captured by the model, compared to just predicting the $\bar{y}$.

R^2

- $R^2 = 1 \rightarrow$ perfect prediction.
- $R^2 = 0 \rightarrow$ model predicts no better than the mean.

16 Phasor Analysis

This method focuses on visualizing the distribution of the 1st harmonic or 2nd harmonic phasor coordinates, extracted by the Lifetime fit free extractor, and performs optional clustering to highlight subpopulations or structural patterns.

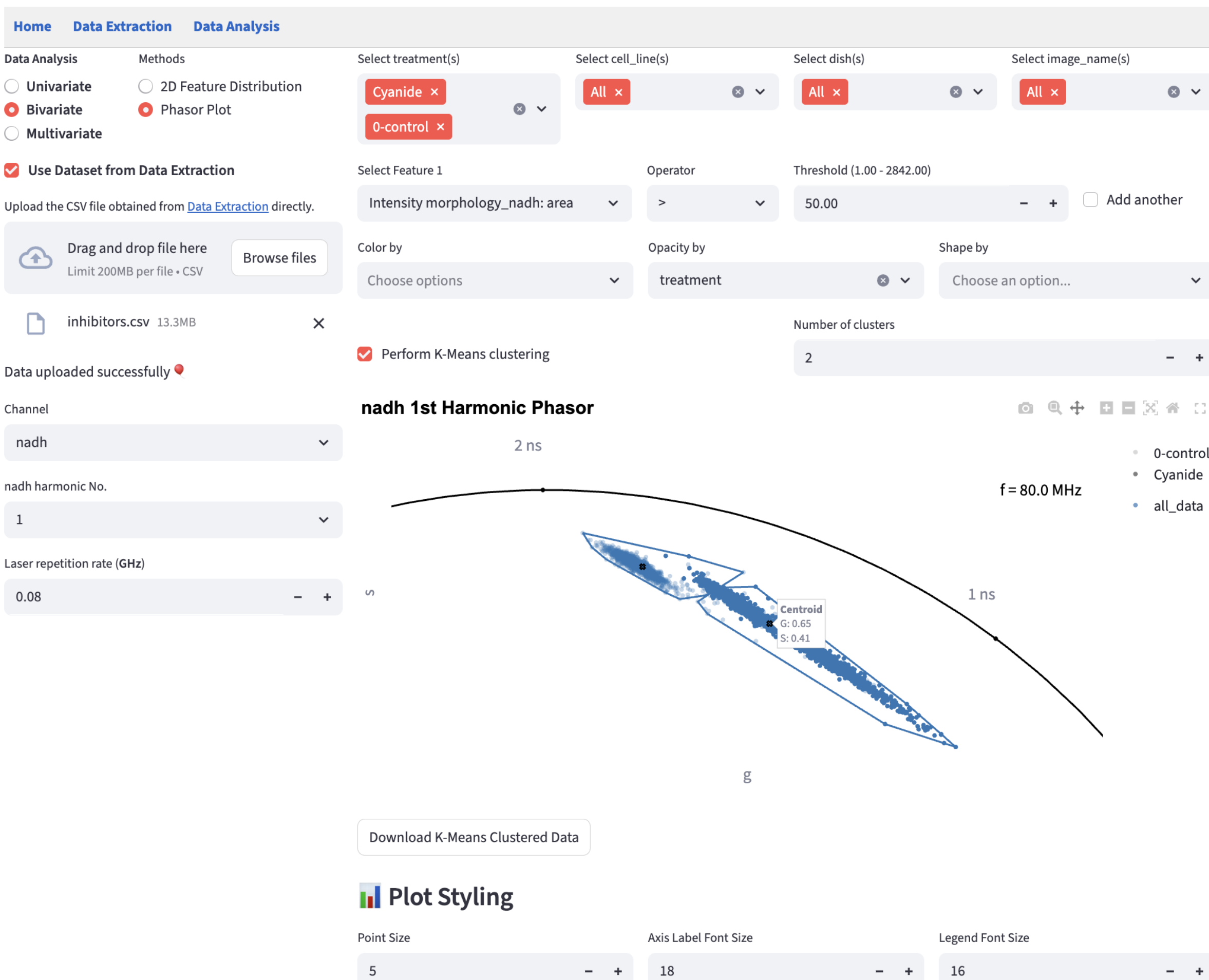

16.1 Shared Interface Components

- On the top right, users can use the filters to subset the data to find the groups of interest.
- Below the filters, users can apply the visual channels widgets that allow users to `Color by`, `Opacity by`, and `Shape by` categorical features.
- On the bottom right, users can change the plot style using the plot styling widgets.

16.2 Select Phasor Coordinate

Instead of using the bivariate selection widgets that select two generic numerical features, this method renders a dropdown menu to select the channel and another to select the harmonic.

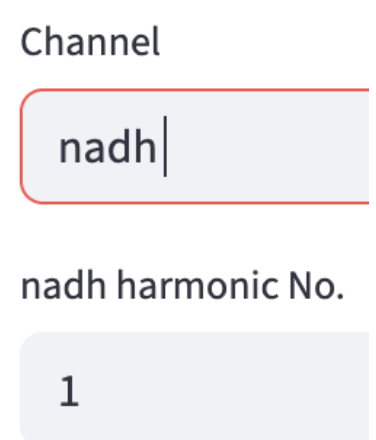

16.3 K-means Clustering

Phasor coordinates of single-cell ROIs from the selected channel and harmonic are plotted in the phasor space. K-means clustering can be performed for each color group (created by `Color by`). If `Perform K-means Clustering` is checked, users can specify the number of clusters to be created based on their prior knowledge. In the future, automatic tuning for this parameter, akin to selecting the number of components of GMM, using silhouette analysis will be added.

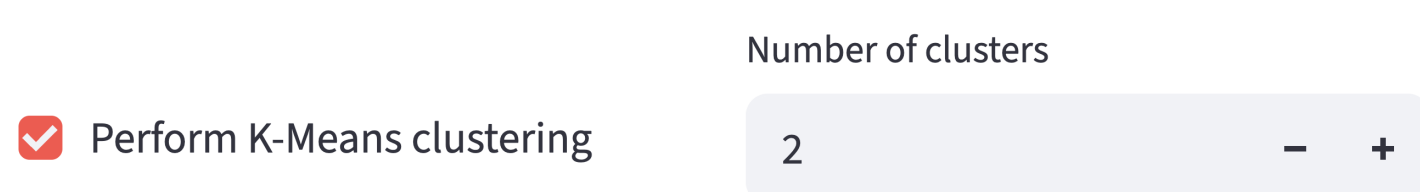

K-means clustering is performed given the number of clusters specified for each color group. Mathematically, it is an iterative algorithm that finds the best cluster assignment with regard to what cluster each point belongs to and what centroid the cluster has. The *best* is defined as the one that minimizes the sum of the squared distances between each point and the center of the cluster it belongs to.

$$\min_{\{C_k\}_{k=1}^{K}} \sum_{k=1}^{K} \sum_{x_i \in C_k} \left\| x_i - \mu(C_k) \right\|_2^2, \qquad \mu(C_k) = \frac{1}{|C_k|} \sum_{x_i \in C_k} x_i.$$

First, FLIM Playground standardizes the phasor coordinates because g (real part) and s (imaginary) are not necessarily on the same scale.

Then, it performs k-means clustering using the `sklearn.cluster.KMeans` function. All the other hyperparameters control the optimization process (e.g. `init` controls the initialization of the centroids), and they are set to their default values. `42` is used as the random seed to ensure reproducibility.

To visualize the result of clustering, FLIM Playground draws a polygon (convex hull) that contains all the points of each cluster, the color of which is determined by the color group the cluster belongs to. It uses the `scipy.spatial.ConvexHull` function to calculate the convex hull. The center of each cluster is marked as a black bordered cross and users can hover over it to see its coordinates.

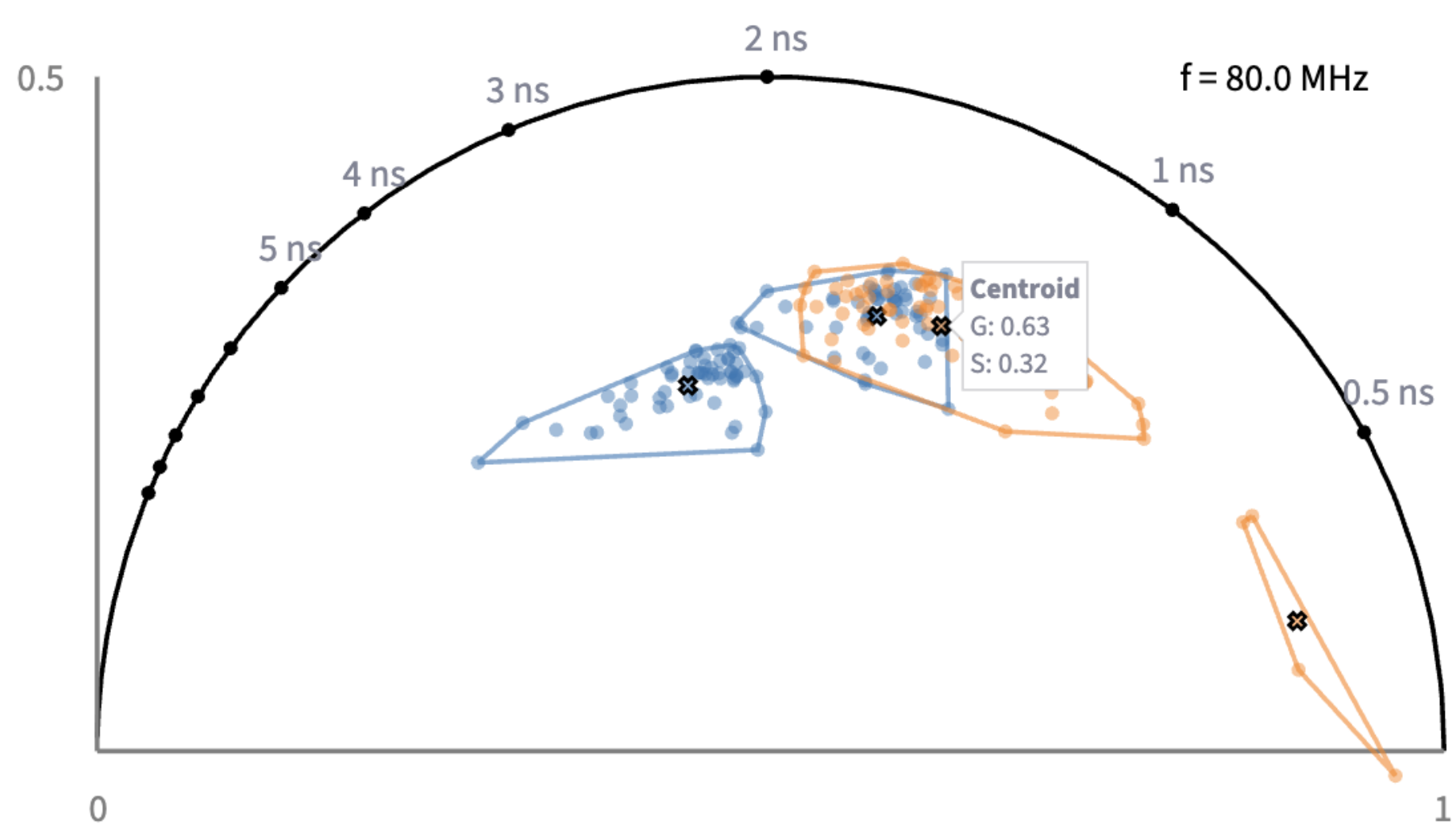

16.3.1 Export Clustered Dataset

Users can download the clustered dataset by clicking:

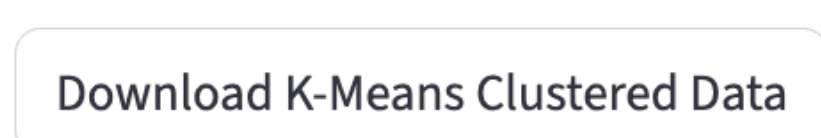

The downloaded dataset keeps all the features plus the new categorical feature column (`k_means_cluster`), which is recognized by any method in Data Analysis as a categorical feature like others. The rows that pass the filters are included.

Part V

Multivariate Analysis

17 Dimension Reduction

To represent and visualize high-dimensional data in a meaningful way and help users interpret the data, dimension reduction methods project the data into a lower-dimensional space. Three popular dimension reduction methods are often used: UMAP, PCA, and t-SNE.

Principal Component Analysis (PCA) is a linear dimension reduction method that projects the data onto the directions of the largest variance. It is fast, deterministic, and easier to interpret (PCA loadings are provided), but it is limited by the linear nature of the projection. For a detailed discussion of PCA, please refer to this post.

Uniform Manifold Approximation and Projection (UMAP) and t-Distributed Stochastic Neighbor Embedding (t-SNE) are non-linear dimension reduction methods that can capture more complex patterns in the data. They are more flexible and can handle non-linear relationships between features, but they are more computationally expensive, less interpretable, not deterministic (FLIM Playground uses the random seed 42 to ensure reproducibility), and dependent on the hyperparameters. Here is a post that explains how UMAP and t-SNE work at a high level and the meaning of some of the hyperparameters.

The numerical features are standardized before the dimension reduction.

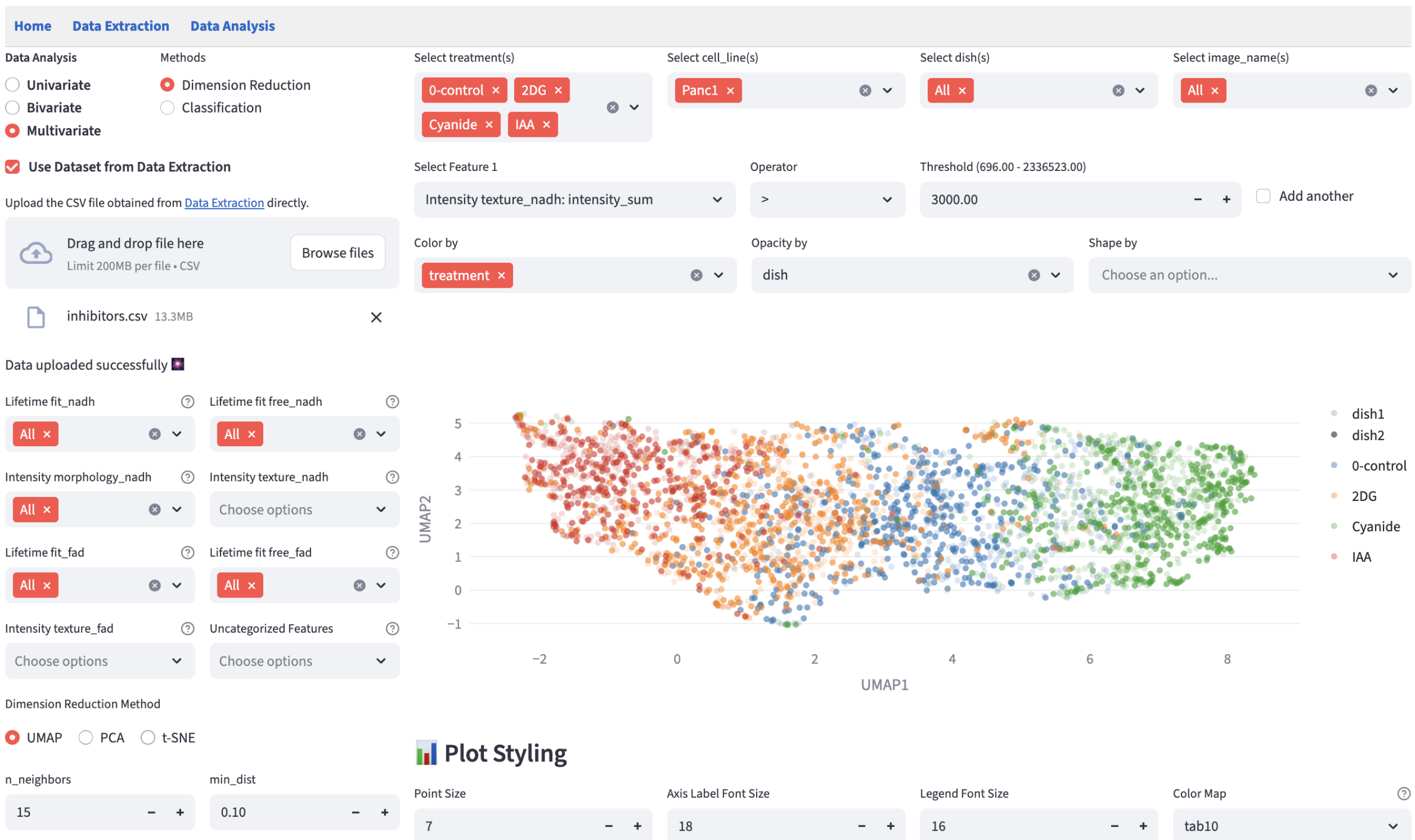

17.1 Shared Interface Components

- On the left, users can use the selection widgets to select multiple numerical features from each feature group.

- On the top right, users can use the filters to subset the data to find the groups of interest.
- Below the filters, users can apply the visual channels widgets that allow users to `Color by`, `Opacity by`, and `Shape by` categorical features.
- On the bottom right, users can change the plot style using the plot styling widgets.

17.2 Hyperparameter Widget

Hyperparameters really matter, so we provide a set of interactive widgets to help users change the hyperparameters and see their effects in real time.

17.2.1 UMAP

FLIM Playground uses the `umap-learn` implementation of UMAP. It chooses to expose `n_neighbors` and `min_dist` for users to tweak. Other hyperparameters are set to default values.

17.2.2 t-SNE

FLIM Playground uses the `sklearn.manifold.TSNE` implementation of t-SNE. It chooses to expose `perplexity` and `early_exaggeration` for users to tweak. Other hyperparameters are set to default values.

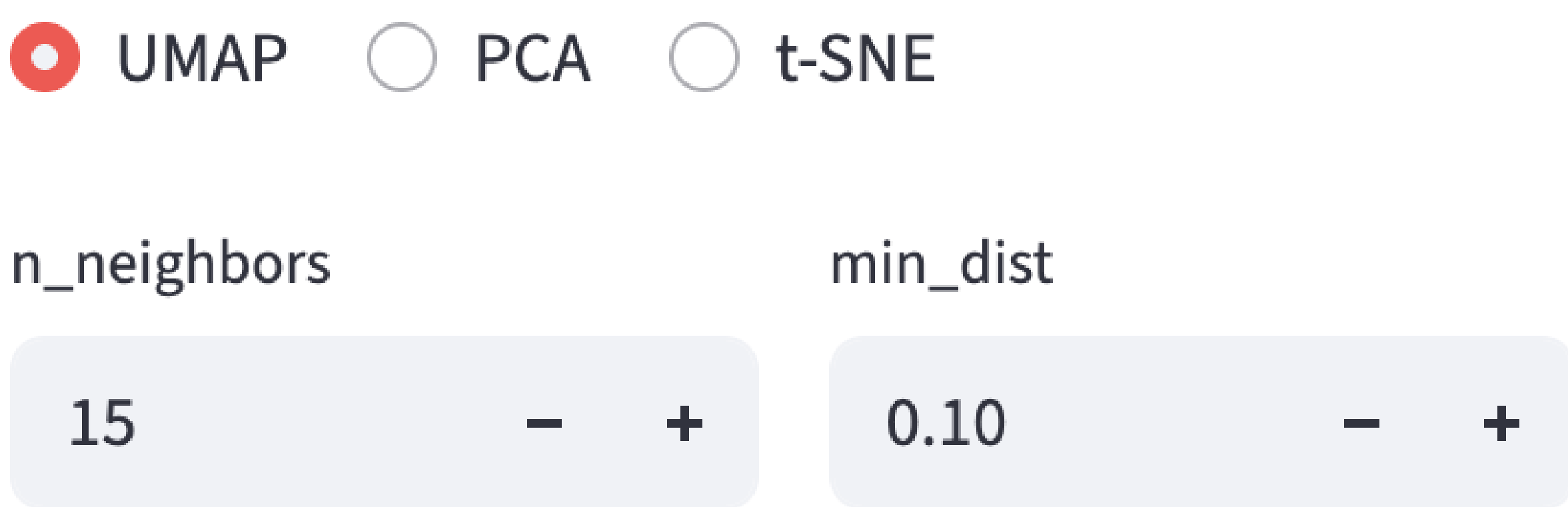

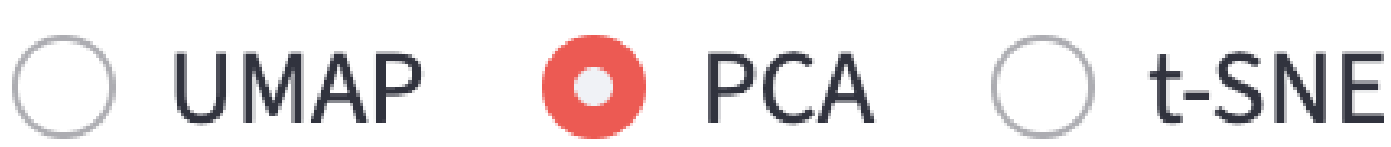

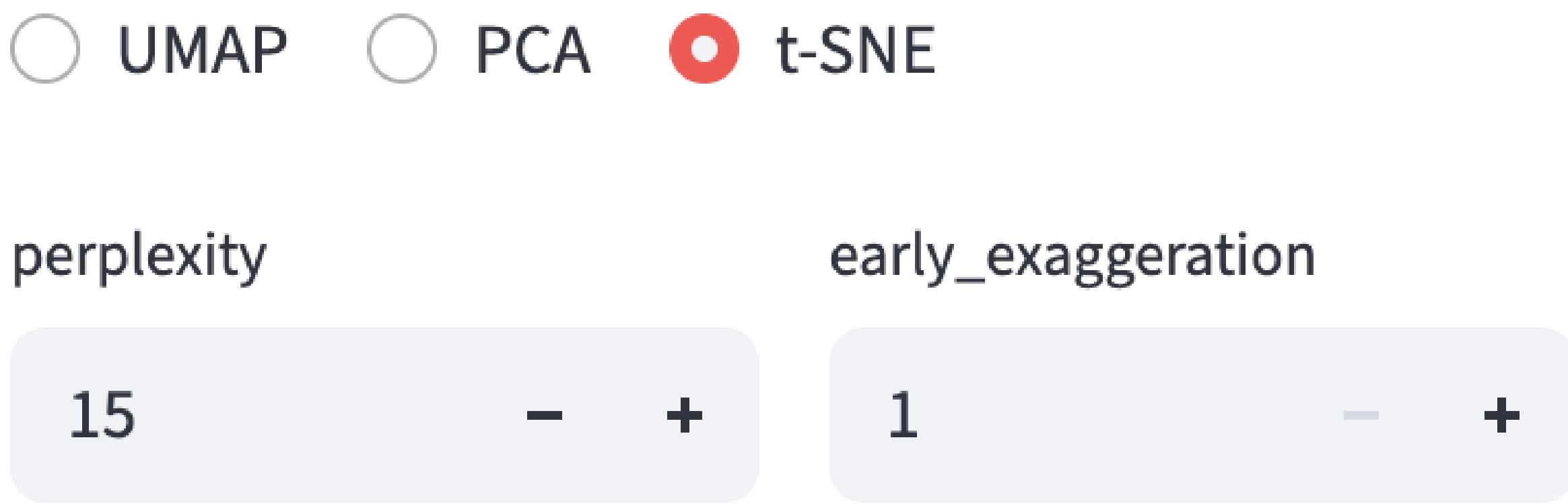
UMAP
PCA
t-SNE
perplexity
15
early_exaggeration
1

18 Classification

A machine learning classifier learns patterns from categorized data and then predicts the category of new, unseen data. This module provides a set of machine learning classifiers and interactive widgets to help users perform classification.

All the classifiers are implemented using `scikit-learn` and the performance metrics are calculated using `sklearn.metrics`. A fixed random seed (42) is used for all the classifiers to ensure reproducibility.

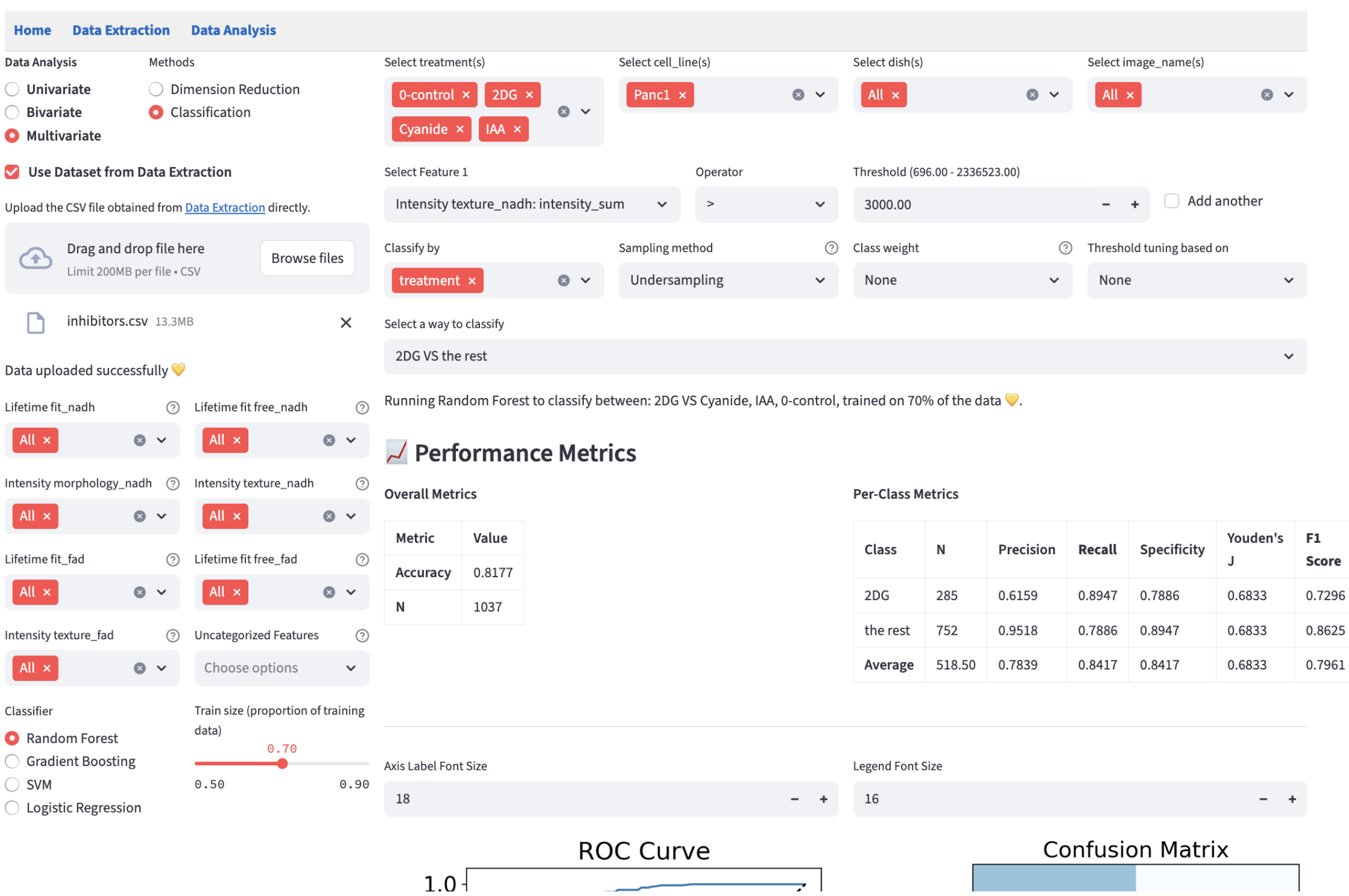

The performance metric plots are cut off in the screenshot. See the performance metrics section for the complete view.

An overview of the pipeline:

- Choose the numerical features on the left.
  - The z-score normalization is appliedto the features before training if the chosen classifier is `SVM` or `Logistic Regression`, because they are sensitive to feature magnitudes, while `Random Forest` and `Gradient Boosting` are not.
- Choose the classifier and train-test split.
- Filter the dataset.
- Choose categorical features to form classes.
- Choose any of the methods to handle class imbalance.
- Choose the classes to be classified.
- Look at the performance metrics, confusion matrix, and ROC curve.
- Iterate over the previous options to improve the performance with regard to the metrics you care about.

18.1 Shared Interface Components

- On the left, users can use the selection widgets to select numerical features for the classifier. Multiple features can be selected from each feature group.
- On the top right, users can use the filters to subset the data to find the groups of interest.
- Above the performance metrics plots, users can change the plot style using the plot styling widgets.

18.2 Method-specific Components

- Choose the classifier and train-test split: the slider controls the proportion of the data used to train the classifier and the rest is used as unseen data to test its generalizability. The following classifiers are available:
  - `Random Forest`
  - `Gradient Boosting`
  - Support Vector Machine (`SVM`)
  - `Logistic Regression`

The train-test split is used to evaluate the generalizability of the classifier. The Validation set is used in hyperparameter tuning. Since the classifiers above do not have a lot of hyperparameters to tune, it does not partition a separate validation set. Though the k-fold cross-validation is used in tuning the threshold.

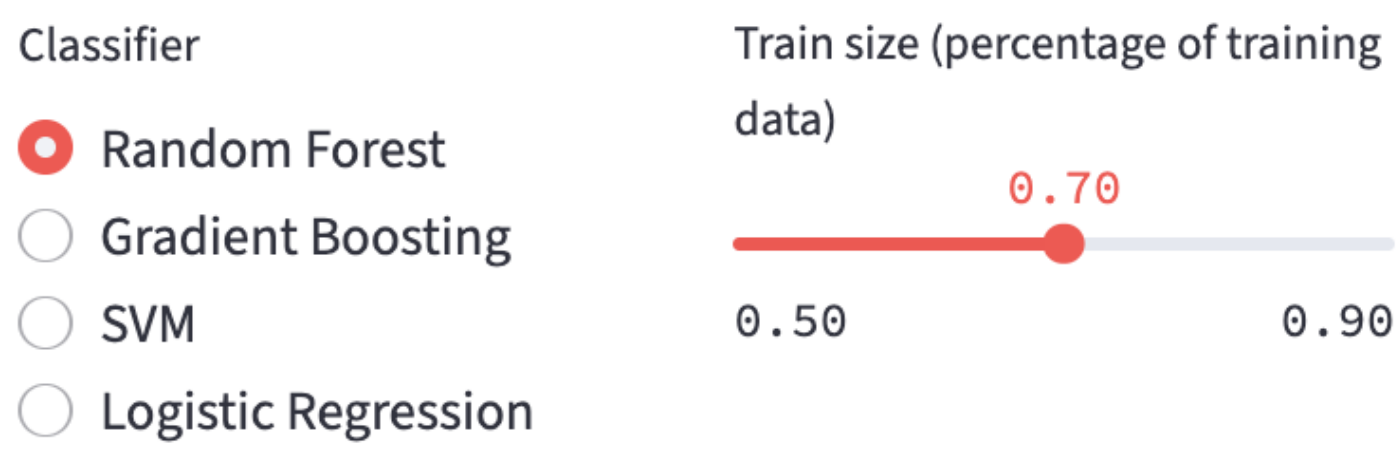

- `Classify by`: The `Color by` from the visual channels widgets is renamed to `Classify by` and it is used to form classes based on (a combination of) selected categorical features. Other visual channels are not supported in this method.

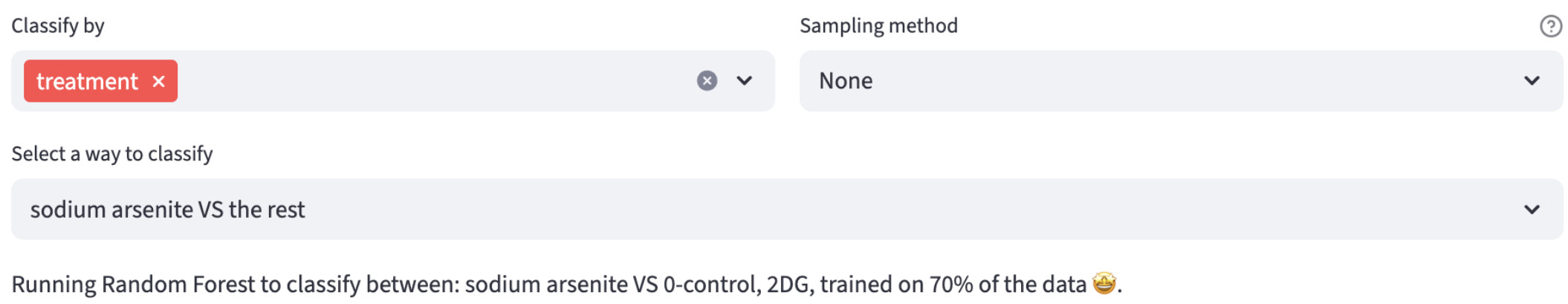

- Choose the classes to be classified: classes are formed based on the combinations of categories of the selected categorical features. In general, it supports binary classification and in general n-way classification where n is the number of classes. If one categorical feature is selected and after filtering has 3 categories, then all possible ways to classify are produced including the three-way classification. Additionally, the `class x vs the rest` option is provided for each class.

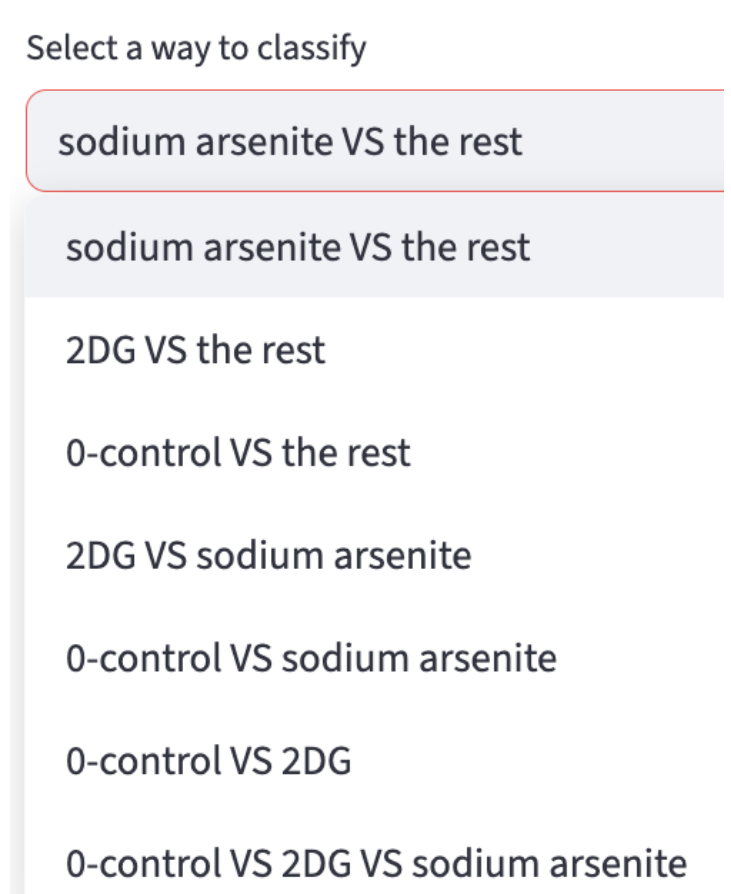

18.2.1 Classifier Hyperparameters

The classifier hyperparameter panel is organized per classifier with three roles:

- `Model-Form/Capacity`: defines the hypothesis family or expressive power.
- `Regularization`: constrains model complexity to reduce overfitting.
- `Optimization`: affects training convergence and runtime behavior.

18.2.1.1 Random Forest

Parameter table

Param	Role	Effect	Priority
`n_estimators`	Model-Form/Capacity	More trees increase ensemble capacity; gains usually show diminishing returns after enough trees.	High
`max_depth`	Regularization	Caps tree depth to reduce overfitting; also directly limits capacity.	High
`min_samples_split`	Regularization	Requires more samples before splitting, reducing fragmentation on small partitions.	Medium
`min_samples_leaf`	Regularization	Enforces minimum leaf size, smoothing predictions.	Medium
`max_features`	Regularization	Fewer features per split increase tree diversity and reduce variance/overfitting.	Medium-High

18.2.1.2 Gradient Boosting

Parameter table

Param	Role	Effect	Priority
`n_estimators`	Model-Form/Capacity	More stages increase additive model capacity; strongly interacts with `learning_rate`.	High
`learning_rate`	Regularization	Shrinks each stage contribution; lower values typically need more trees and often generalize better (also affects optimization dynamics).	High
`max_depth`	Regularization	Limits base learner complexity; also limits capacity.	High
`subsample`	Regularization	`< 1.0` introduces stochastic boosting, reducing variance and overfitting risk.	High

18.2.1.3 Support Vector Machine (SVM)

Parameter table

Param	Role	Effect	Priority
`C`	Regularization	Higher `C` penalizes training errors more, which usually weakens regularization.	High

`kernel`	Model-Form/Capacity	Chooses hypothesis family (`linear` vs non-linear kernels).	High
`gamma`	Model-Form/Capacity	Controls influence radius of samples for `rbf`/`poly`/`sigmoid`; higher values create tighter boundaries.	High
`degree`	Model-Form/Capacity	Polynomial order for `poly` kernel; higher degree increases flexibility.	Medium-High
`coef0`	Model-Form/Capacity	Kernel offset for `poly`/`sigmoid`, shifting boundary behavior.	Medium
`tol`	Optimization	Convergence tolerance; mostly affects runtime/termination behavior.	Low

18.2.1.4 Logistic Regression

Parameter table

Param	Role	Effect	Priority
`regularization`	Regularization	Selects regularization type; available options depend on `solver` (see table below). Maps to `l1_ratio` internally.	High
`C`	Regularization	Inverse regularization strength; higher `C` means weaker regularization.	High

l1_ratio	Regularization	Balances l1 vs l2 when solver='saga' and regularization='elasticnet'.	Medium
fit_intercept	Regularization	Disabling intercept is a mild structural constraint.	Low
solver	Optimization	Optimization algorithm; affects convergence speed and which regularization options are available.	Low-Medium
max_iter	Optimization	Iteration budget; too low can stop before convergence.	Low-Medium
tol	Optimization	Convergence threshold; mainly runtime-sensitive.	Low

Regularization options by solver:

Solver	Available regularization
lbfgs	l2, none
liblinear	l1, l2
newton-cg	l2, none
newton-cholesky	l2, none
sag	l2, none
saga	l1, l2, elasticnet, none

18.2.2 Class Imbalance Handling

We provide three options for handling class imbalance (i.e., one or more classes in a dataset have significantly fewer samples than others, which would otherwise cause a classifier to bias toward the majority class). Note: to prevent data leakage, all the methods below are applied to the training set only.

- **Sampling methods**:
  - None: stratified random sampling of the data on classification classes, regardless of the class size. It is implemented using train_test_split from sklearn.model_selection.

  - `Undersampling`: All classes are downsampled to the size of the smallest class. It is implemented using `RandomUnderSampler` from `imbalanced-learn`.
  - `Oversampling`: All classes are upsampled to the size of the largest class by randomly duplicating samples from minority classes. It is implemented using `RandomOverSampler` from `imbalanced-learn`.

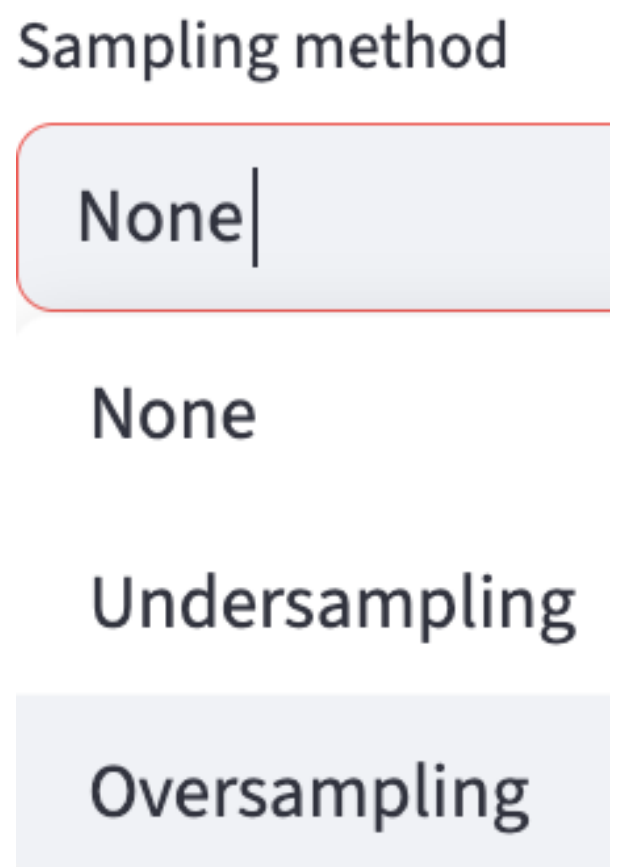

- **Class weights**:
  - `None`: no class weights.
  - `Balanced`: the class weights are inversely proportional to the class size (support). Note: Gradient Boosting does not support class weights in the scikit-learn implementation, and thus this option is not available for it.

- **Threshold tuning**: Whereas the previous two options influence the probablity estimation part of the classifier, this option adjusts only the decision boundary used to convert predicted probabilities into class labels.
  - `None`: no threshold tuning. The default decision is always the class that has the highest predicted probability (in binary, anything above 0.5; in multi-class, the class with the highest predicted probability).
  - `Balanced Accuracy`: the threshold is set to maximize the balanced accuracy score, which is the unweighted average of each class's recall (i.e., the minority class's recall is as important as the majority class's recall).
  - `F1 Score`: the threshold is set to maximize the unweighted average of each class's F1 score.

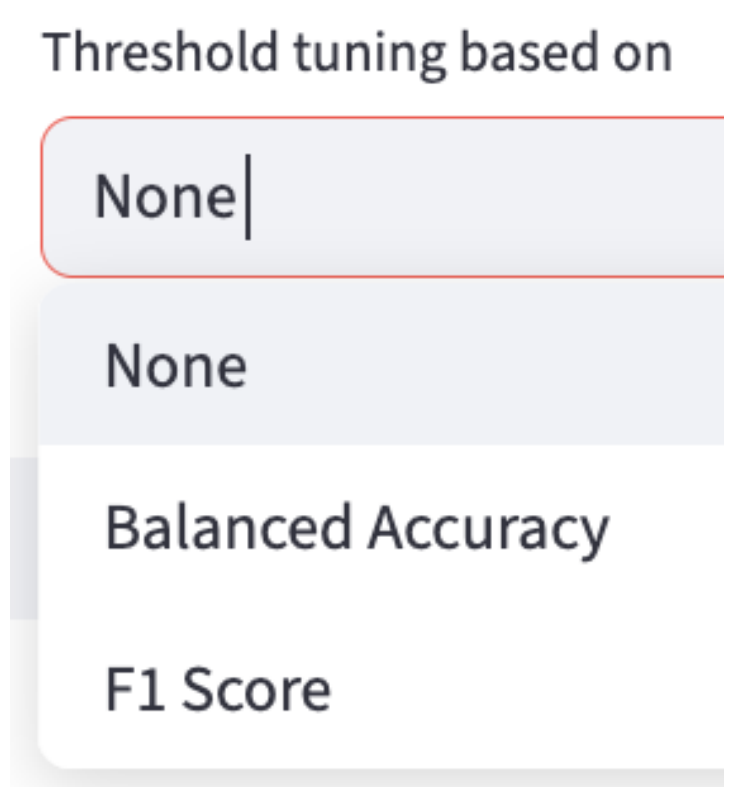

How does threshold tuning work?

It treats threshold selection as a separate optimization problem from model training, allowing thresholds to be tuned independently of the underlying classifier's probability estimates.
K-fold cross-validation for the probability model: The procedure begins by establishing a stratified k-fold cross-validation scheme (default k=5): the dataset is divided into k mutually exclusive subsets, and for each fold, one subset serves as the validation set while the remaining k-1 subsets form the training set.
The probability models are trained separately for each cross-validation fold. This process yields k distinct probability models, each trained on a different subset of the data, along with their corresponding validation set probability predictions that collectively cover all samples exactly once.
Threshold optimization: A single threshold optimization procedure is performed once using all cross-validation folds. The threshold optimization process differs for binary and multi-class classification problems. For binary classification, a single threshold parameter $\theta \in [0, 1]$ is optimized, where class 1 is predicted when $P(\text{class } 1 \mid x) \geq \theta$, and class 0 otherwise. For multi-class problems, a threshold vector $\theta = [\theta_1, \theta_2, \ldots, \theta_k]$ is optimized simultaneously for all k classes, where thresholds are applied through a normalized probability scheme: predictions are made by selecting the class with the highest normalized probability $P(\text{class } i \mid x)/(\theta_i + \epsilon)$, where $\epsilon = 10^{-10}$ prevents division by zero.
The optimization objective function evaluates candidate thresholds by: (1) applying the same threshold(s) on top of the fold-specific probability models to generate prediction classes for the fold-specific validation set, (2) computing the specified evaluation metric (e.g., balanced accuracy, F1-score) between predicted and true labels for each fold, and (3) returning the negative mean cross-validated score across all folds (for minimization using the Nelder-Mead simplex algorithm). Finally, the optimized threshold(s) are applied on top of the probablity model trained on the entire training set to the test set to generate the final predictions.

Threshold Tuning Example

Before threshold tuning:

Overall Metrics

Metric	Value
Accuracy	0.8854
N	4231

Per-Class Metrics

Class	N	Precision	Recall	Specificity	Youden's J	F1 Score
2DG	757	0.7259	0.5773	0.9525	0.5298	0.6431
the rest	3474	0.9118	0.9525	0.5773	0.5298	0.9317
Average	2115.50	0.8189	0.7649	0.7649	0.5298	0.7874

Axis Label Font Size

18 – +

Legend Font Size

16 – +

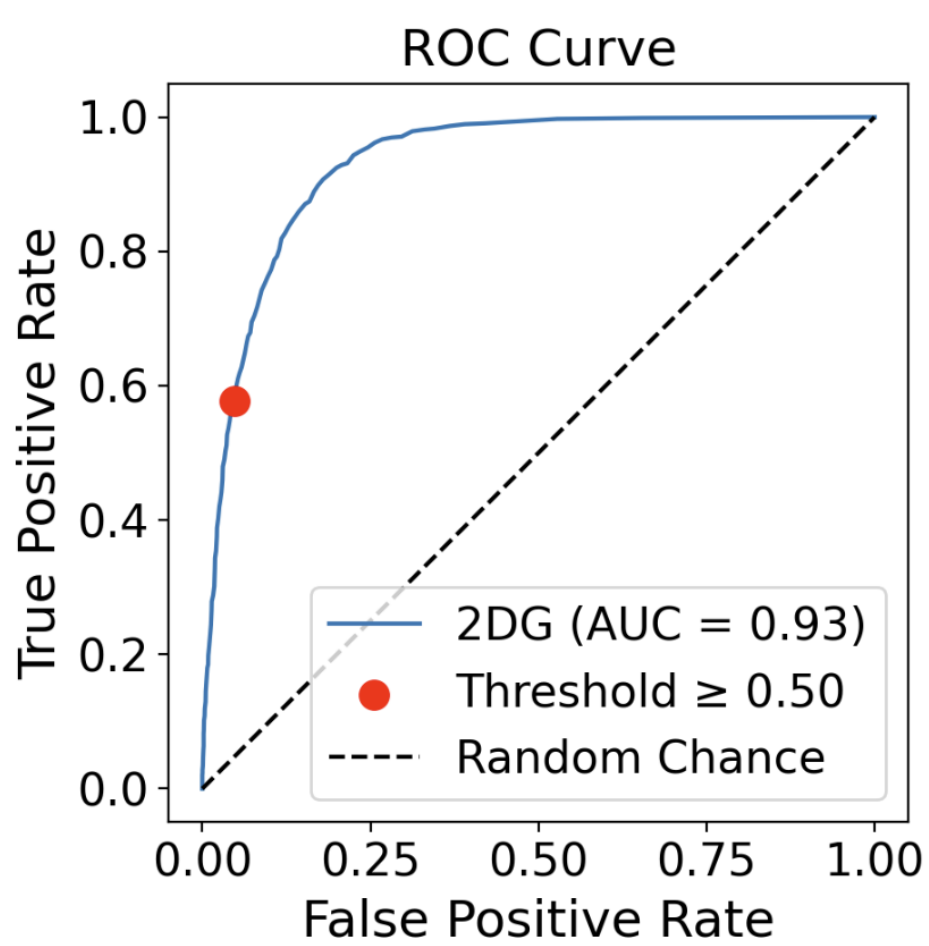

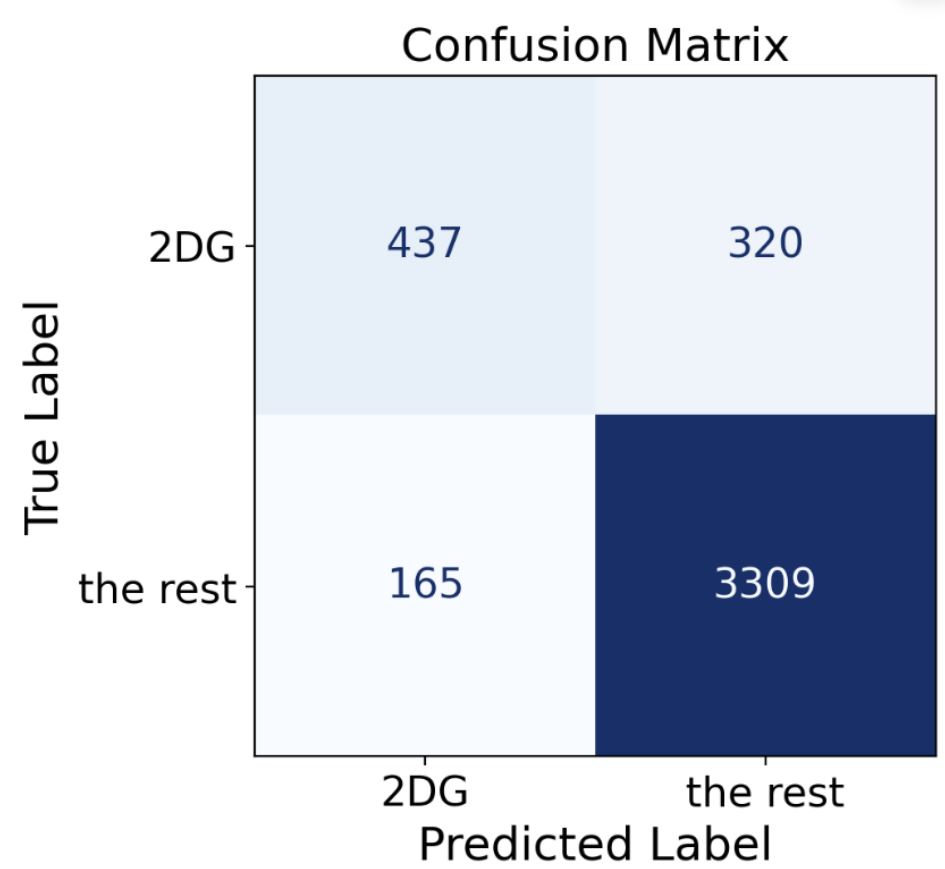

After threshold tuning based on the balanced accuracy metric:

📈 Performance Metrics

Overall Metrics

Metric	Value
Accuracy	0.8225
N	4231

Per-Class Metrics

Class	N	Precision	Recall	Specificity	Youden's J	F1 Score
2DG	757	0.5022	0.9247	0.8002	0.7249	0.6509
the rest	3474	0.9799	0.8002	0.9247	0.7249	0.8810
**Average**	2115.50	0.7410	0.8625	0.8625	0.7249	0.7659

Axis Label Font Size

18 − +

Legend Font Size

16 − +

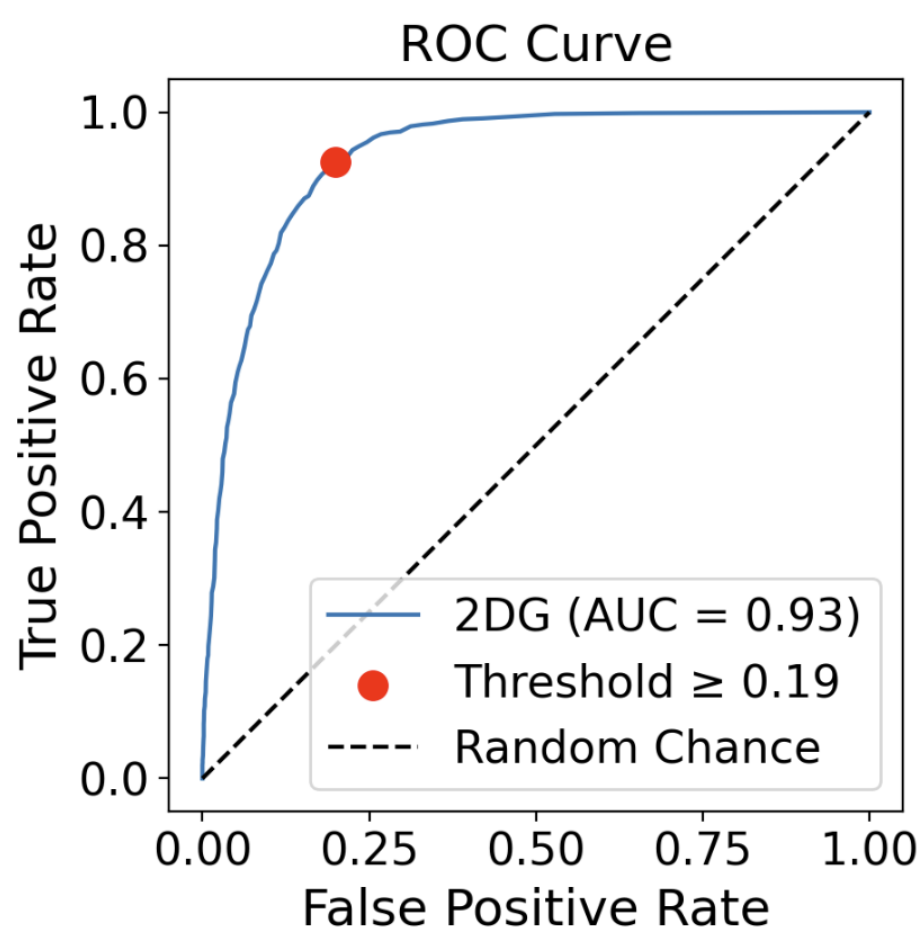

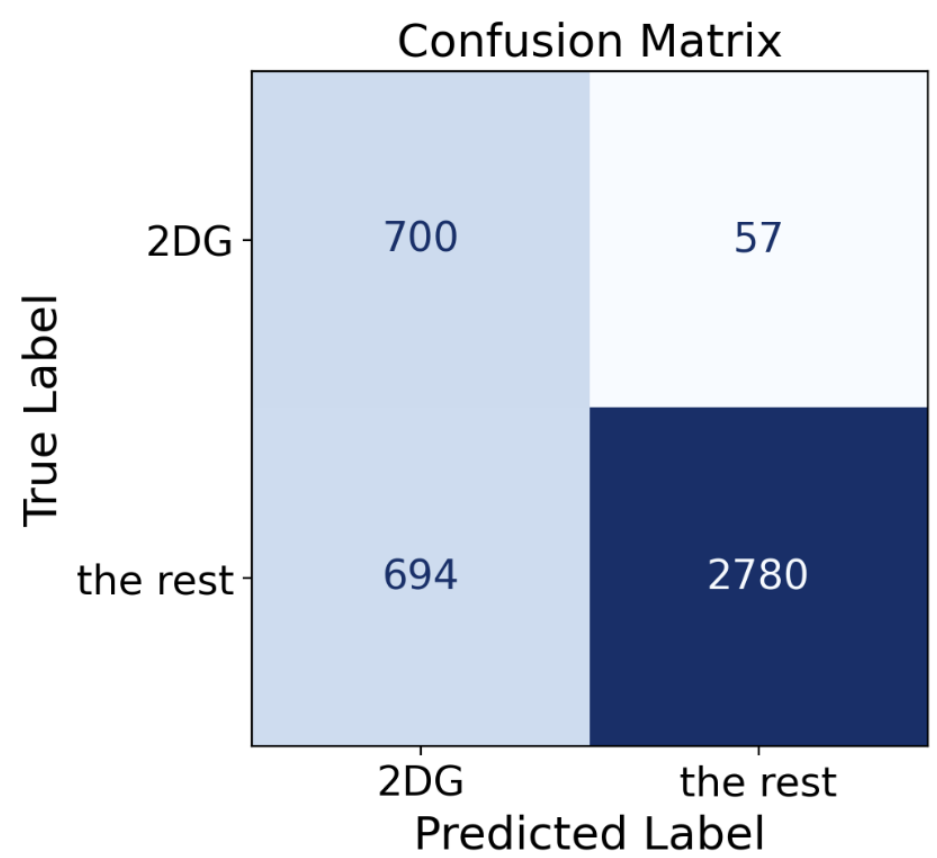

Things to **highlight**:

- The two ROC curves are the same, because the underlying probability models are the same.
- The annotated operating point in red is "better" *after* the threshold tuning because it achieves higher Recall (TRP) + Specificity (TNR), as can be seen in the metrics table (the optimized metrics are highlighted in red). The threshold before was 0.5, the default value, and after tuning it was optimized to 0.19. The reduction in thresholds means that the classifier is tuned to be more "confident" at predicting the positive class (2DG), which is the minority class. The effect of this threshold optimization is also visible in the confusion matrix.
- However, all of the above come at the cost of other metrics, such as the overall accuracy (0.89 to 0.82) and average precision (0.82 to 0.74). **Select the class imbalance methods/options that maximize the metrics most important to you.**

Sampling Example

Before sampling:

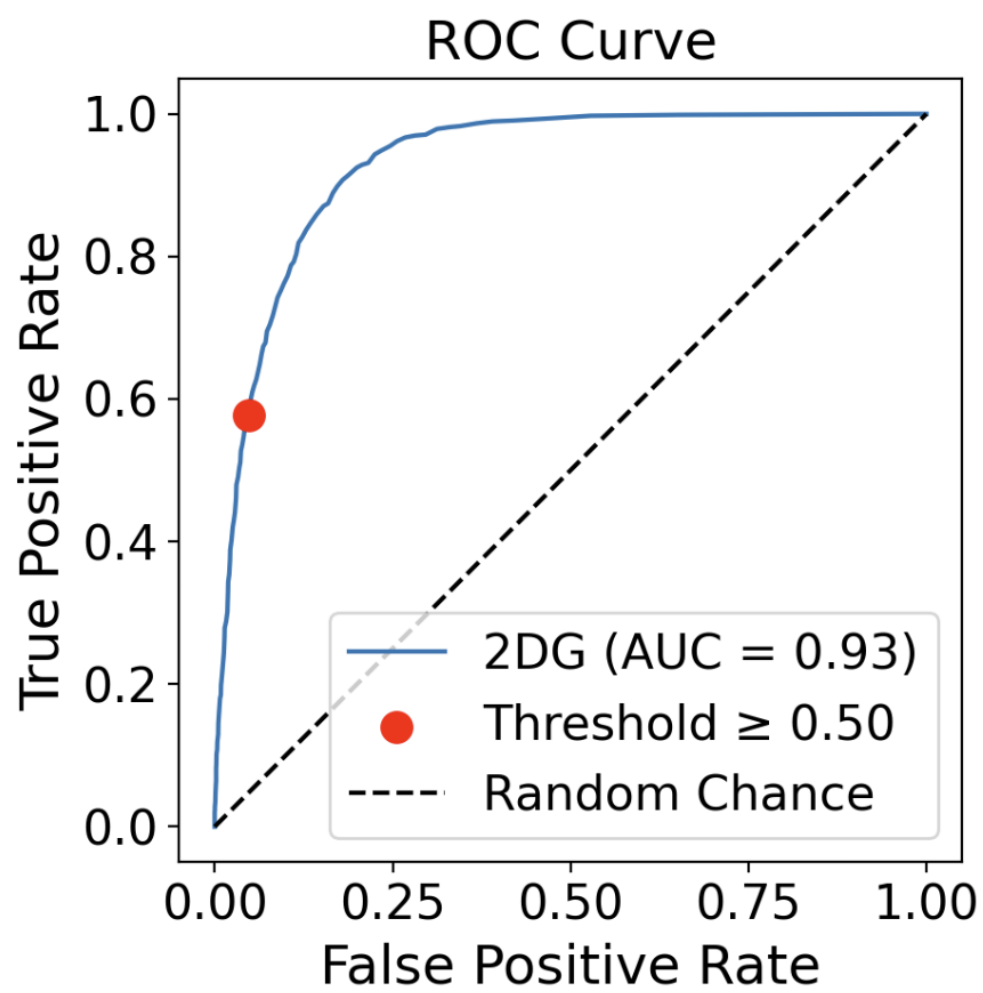

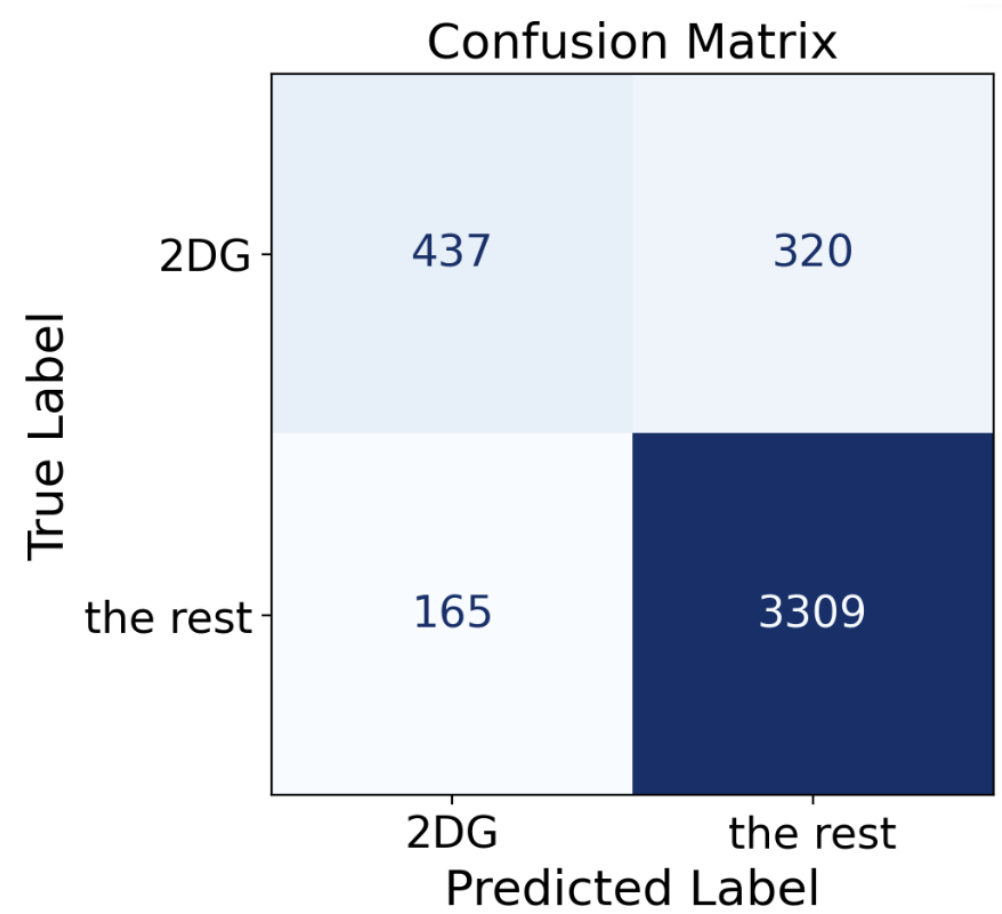

After sampling (undersampling was used in this case):

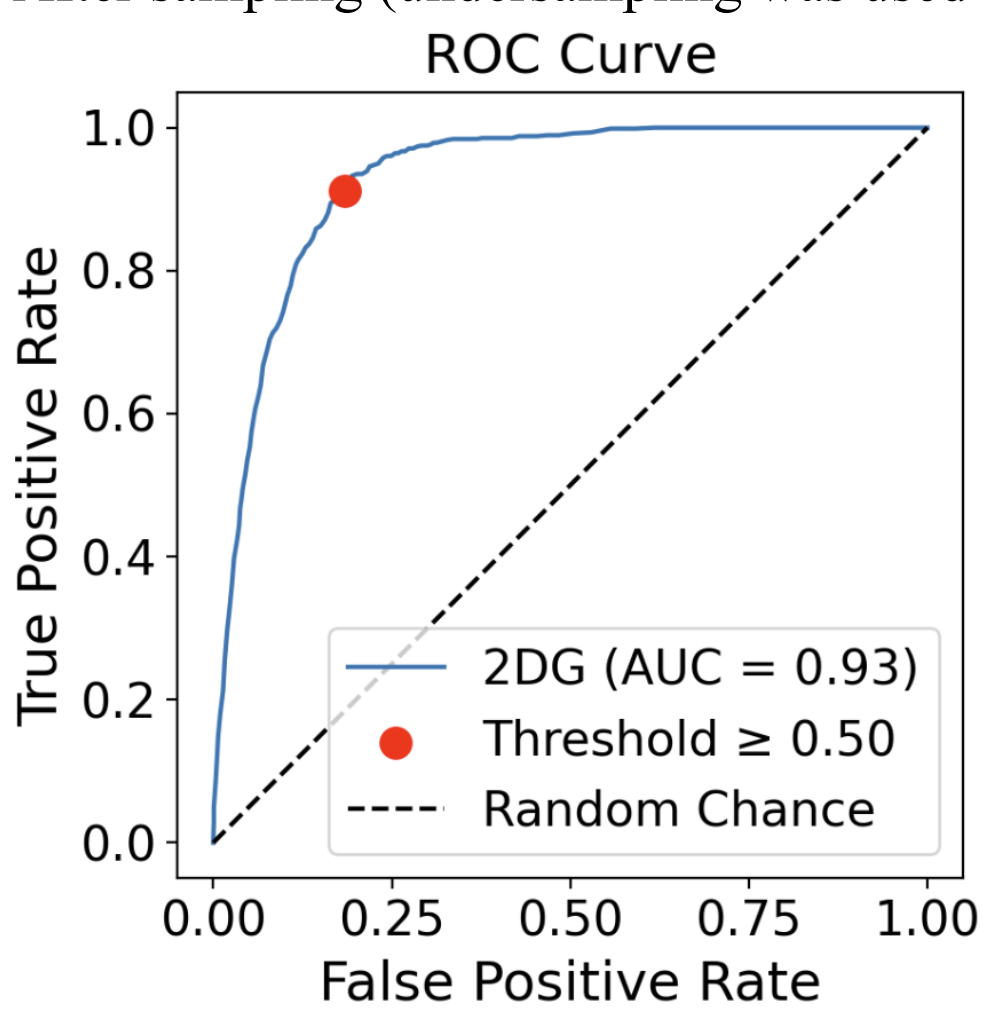

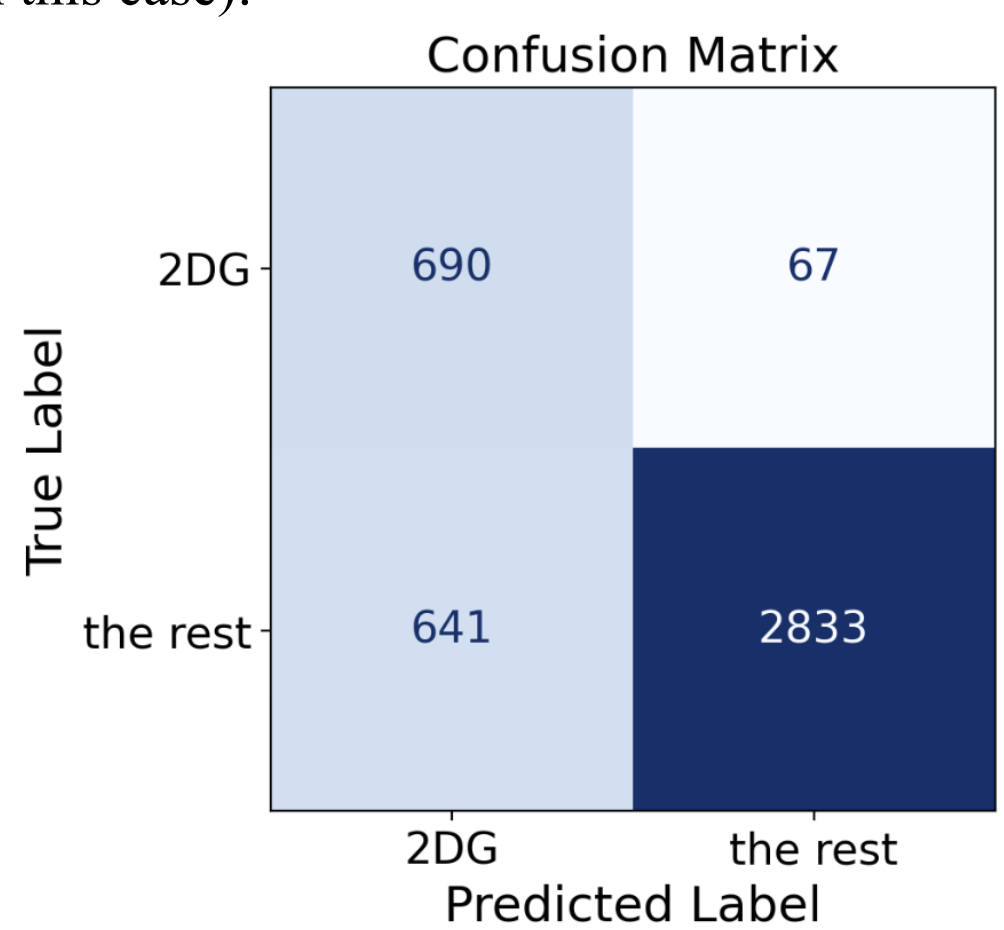

Things to **highlight**:

- The ROC curve is different, because the underlying probability models are different.
- The thresholds before and after are the same (the default value of 0.5), but the operating points are at different locations and their associated confusion matrices are different.

18.2.3 Confusion Matrix

FLIM Playground evaluates the trained classifier on the held-out test set by converting the predicted probabilities into hard labels using the chosen threshold or by selecting the class with the highest predicted probability. The predictions are then summarized using sklearn.metrics.confusion_matrix. In the binary case, it is represented as:

	Predicted Positive	Predicted Negative
**Actual Positive**	**True Positive (TP)** – correct hits	**False Negative (FN)** – missed positives
**Actual Negative**	**False Positive (FP)** – false alarms	**True Negative (TN)** – correct rejections

18.2.4 Performance Metrics

The overall accuracy is calculated as the ratio of the sum of the true positives and true negatives to the sum of all the elements in the confusion matrix (the sum of the diagonal over the sum of all the elements).

- Accuracy: $\frac{TP+TN}{TP+FP+TN+FN}$

Other metrics are calculated for each class based on the confusion matrix:

- Precision: $\frac{TP}{TP+FP}$
- Recall (Sensitivity): $\frac{TP}{TP+FN}$
- Specificity: $\frac{TN}{TN+FP}$
- Youden's Index: Sensitivity $+$ Specificity $-$ 1
- F1 Score: $\frac{2\times\text{Precision}\times\text{Recall}}{\text{Precision}+\text{Recall}}$

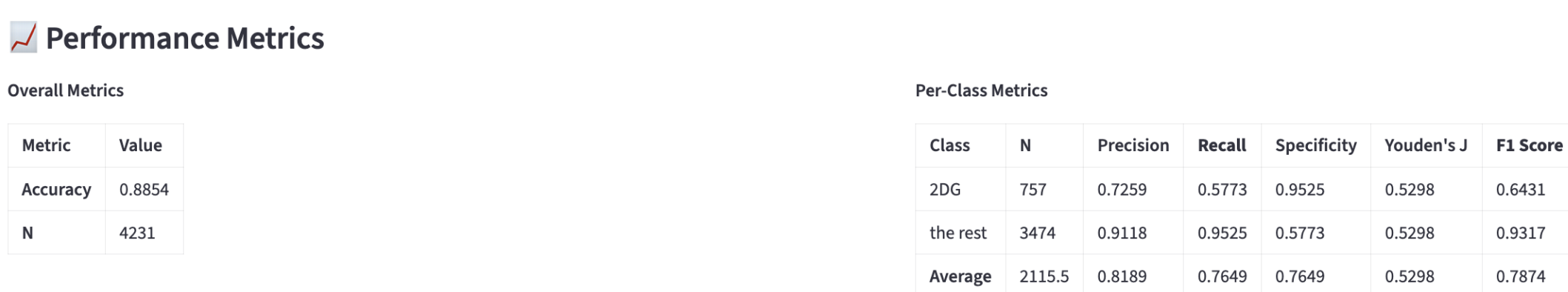

Performance Metrics

Overall Metrics

Metric	Value
Accuracy	0.8854
N	4231

Per-Class Metrics

Class	N	Precision	Recall	Specificity	Youden's J	F1 Score
2DG	757	0.7259	0.5773	0.9525	0.5298	0.6431
the rest	3474	0.9118	0.9525	0.5773	0.5298	0.9317
Average	2115.5	0.8189	0.7649	0.7649	0.5298	0.7874

> Performance Metrics Example
>
> The reason that we need metrics in addition to accuracy is because *Accuracy is Not Enough*. For a visual explantion of metrics, see here. In short, it is all about trade-off.

18.2.5 ROC Curve

Another way to look at the performance of the classifier is to plot the Receiver Operating Characteristic (ROC) curve and its Area Under the Curve (AUC).

Confusion matrix looks at the performance of the classifier at a single threshold, while ROC curve looks at the performance of the classifier at all thresholds (i.e., from 0 to 1).

At the origin (0, 0), the threshold is 1, so the classifier predicts all samples as negative, making the false positive rate $\text{FPR} = \frac{\text{FP}}{\text{FP+TN}} = 0$ and the true positive rate $\text{TPR} = \frac{\text{TP}}{\text{TP+FN}} = 0$, i.e. the origin. At the point (1, 1), the threshold is 0, so the classifier predicts all samples as positive, making the false positive rate 1 and the true positive rate 1, i.e. the point (1, 1).

AUC summarizes the ROC curve by integrating it from 0 to 1, so it is threshold-free and has a nice interpretation: it is the probability that the classifier assigns a higher score to a randomly chosen positive sample X^+ than to a randomly chosen negative sample X^-.

The ROC curve displays the operating point (FPR, TPR) associated with the confusion matrix on the right.

For a visual explanation, see here.

18.2.5.1 Multi-class ROC Curve

In multi-class classification, each class's ROC curve is computed as a one-vs-rest binary classification problem. The operating point (FPR, TPR) associated with the confusion matrix is displayed for each class.

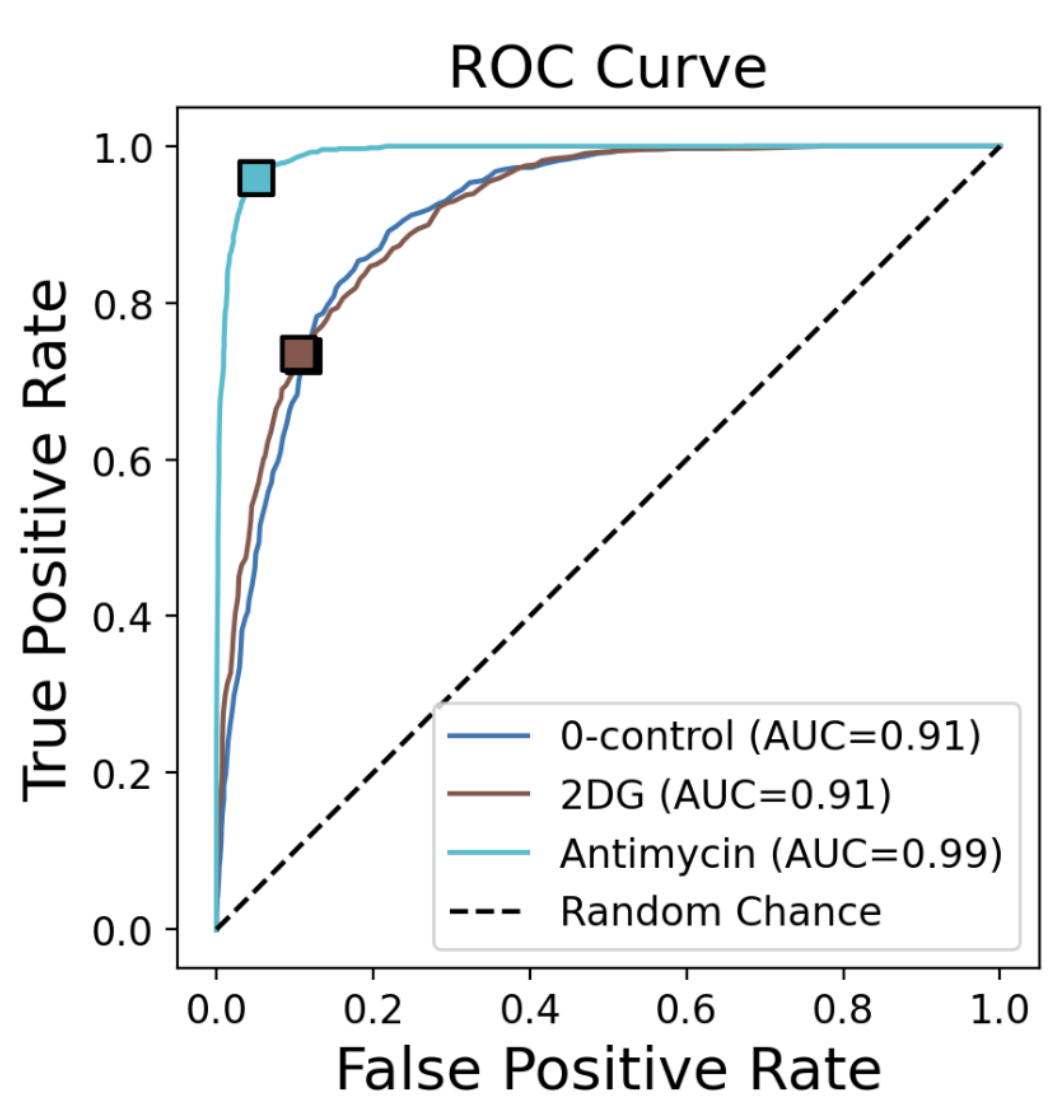

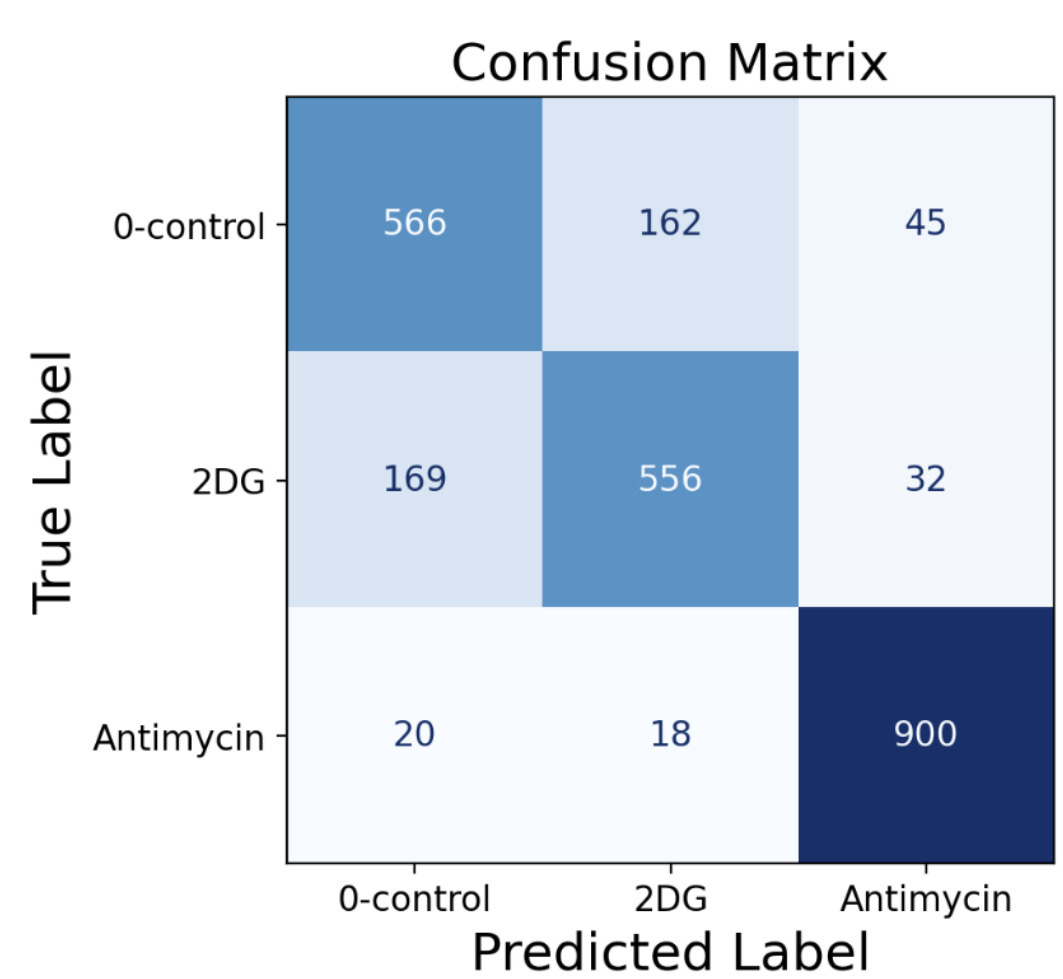

18.2.6 Feature Importance

`Random Forest` and `Gradient Boosting` classifiers provide feature importance scores.

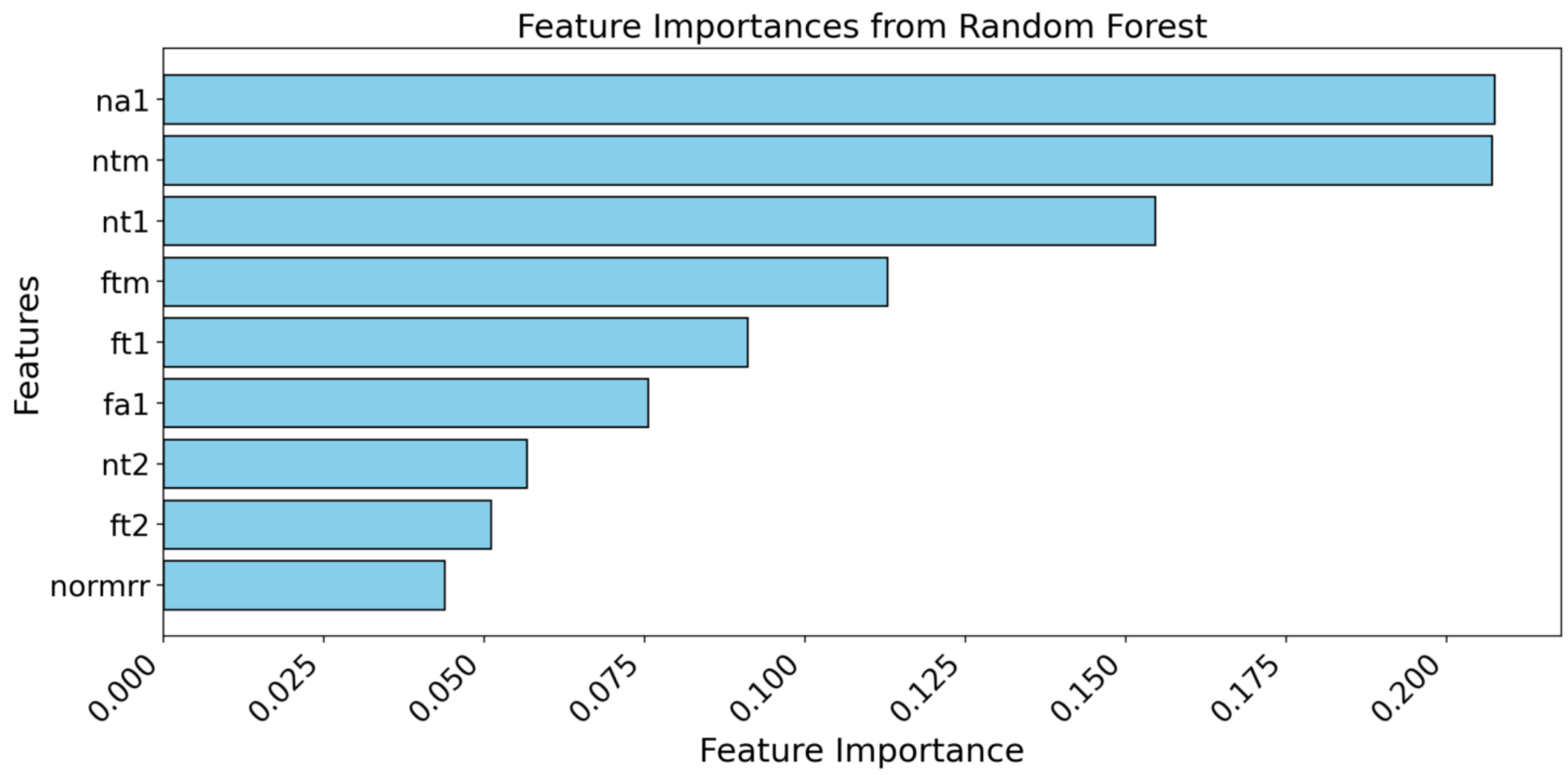

19 Common Q & A

This chapter addresses frequently asked questions about FLIM Playground.

Q: Why I cannot find a certain **numerical feature** under numerical selection/filter widgets?

A: If you cannot find a certain **numerical feature**, try the following:

- look inside the `Uncategorized Features` feature group
- inspect the dataset (e.g., using Excel filters) and look for non-numeric values in that column. One non-numeric value (e.g., "–") will prevent it from being recognized. After clearing those cells (Excel cell not real cells), saving the file, and uploading it again, the feature should be recognized.

Q: Why I cannot find a certain **categorical feature** under categorical filter/visual channel widgets?

A: - If the uploaded dataset was extracted by Data Extraction, FLIM Playground will only match categorical features specified in the Data Extraction configuration. Otherwise, FLIM Playground will only match categorical features specified in the configuration. - If the categorical feature was in the corresponding configuration, and it is still not visible: - If you just added it, FLIM Playground may not have picked it up yet. Try refreshing the page. - If you have already added it, then there is only one unique value in that categorical feature column. FLIM Playground will not show it.

- It is ok if your dataset does not have all of the categorical features specified in either of the configurations.

Q: How do I change the FLIM Playground's display mode (i.e., dark mode or light mode)?

A: FLIM Playground uses the browser's current display mode. You can change it by going to the browser's settings and changing the display mode.

1. Datta, R., Heaster, T. M., Sharick, J. T., Gillette, A. A. & Skala, M. C. Fluorescence lifetime imaging microscopy: fundamentals and advances in instrumentation, analysis, and applications. *Journal of Biomedical Optics* **25**, 071203 (2020).
2. Stringer, C., Wang, T., Michaelos, M. & Pachitariu, M. Cellpose: A generalist algorithm for cellular segmentation. *Nature methods* **18**, 100–106 (2021).
3. Gohlke, C., Pannunzio, B., Schüty, B. & Blanco, R. Phasorpy/phasorpy: v0.7. Zenodo https://doi.org/10.5281/zenodo.16923774 (2025).
4. Gottlieb, D., Asadipour, B., Kostina, P., Ung, T. P. L. & Stringari, C. FLUTE: A python GUI for interactive phasor analysis of FLIM data. *Biological Imaging* **3**, e21 (2023).

5. Kapsiani, S. *et al.* FLIMPA: A versatile software for fluorescence lifetime imaging microscopy phasor analysis. *Analytical Chemistry* (2025).
6. Becker, W. *The Bh TCSPC Handbook*. (Becker & Hickl GmbH, 2021).
7. Stirling, D. R. *et al.* CellProfiler 4: Improvements in speed, utility and usability. *BMC Bioinformatics* **22**, 433 (2021).
8. Stirling, D. R., Carpenter, A. E. & Cimini, B. A. CellProfiler analyst 3.0: Accessible data exploration and machine learning for image analysis. *Bioinformatics* **37**, 3992–3994 (2021).
9. Samimi, K. *et al.* Segmentation-guided photon pooling enables robust single cell analysis and fast fluorescence lifetime imaging microscopy. *bioRxiv* https://doi.org/10.1101/2025.09.30.679660 (2025) doi:10.1101/2025.09.30.679660.
10. Samimi, K. *et al.* Autofluorescence lifetime flow cytometry with time-correlated single photon counting. *Cytometry Part A* **105**, 607–620 (2024).
11. Warren, S. C. *et al.* Rapid global fitting of large fluorescence lifetime imaging microscopy datasets. *PLoS ONE* **8**, e70687 (2013).
12. Cleveland, W. S. & McGill, R. Graphical perception: Theory, experimentation, and application to the development of graphical methods. *Journal of the American Statistical Association* **79**, 531–554 (1984).
13. Virtanen, P. *et al.* SciPy 1.0: Fundamental Algorithms for Scientific Computing in Python. *Nature Methods* **17**, 261–272 (2020).
14. Shannon, C. E. A mathematical theory of communication. *The Bell System Technical Journal* **27**, 379–423 (1948).
15. Pham, D. L. *et al.* Autofluorescence imaging reveals the impact of cryopreservation on t cell metabolism and activation response. *bioRxiv* https://doi.org/10.1101/2025.11.14.688556 (2025) doi:10.1101/2025.11.14.688556.
